# Anatomy-driven automated subsegmentation of pelvic and proximal femoral CT into clinically relevant subregions and landmarks

**DOI:** 10.1186/s12880-026-02753-x

**Published:** 2026-09-08

**Authors:** Mohammed Rashed, Hatem Alabdulrahman, Simon D. Westfechtel, Frank Hildebrand, Daniel Truhn

**Affiliations:** 1 Department of Orthopaedic Surgery, University Hospital Aachen, Aachen, Germany; 2 Department of Diagnostic and Interventional Radiology, University Hospital Aachen, Aachen, Germany

**Keywords:** Pelvic CT, Automated segmentation, Proximal femur, Acetabulum, Anatomical landmarks, 3D Slicer

## Abstract

**Background:**

The pelvis is among the most demanding regions in musculoskeletal imaging, and CT is its reference standard. Available automated tools, however, largely produce whole-bone or single-task masks, leaving the finer surgical subregions to slow, operator-dependent manual work. We developed a rule-based pipeline that converts pelvic and proximal femoral masks into a structured map of surgically meaningful subregions and landmarks, quantifying the manual correction burden required to achieve anatomically acceptable outputs.

**Methods:**

Anonymized pelvic CT scans were collected retrospectively; after predefined quality criteria, 757 of 1,316 screened normal adult cases were processed by a one-click pipeline in 3D Slicer. Parent structures from TotalSegmentator were divided by fixed anatomical rules into columns, acetabular regions, and proximal femoral regions. In 100 randomly selected cases, every segment was expert-reviewed and corrected where needed; correction frequency was the primary endpoint, with accuracy computed for corrected segments only. Pipeline performance was also assessed in a 60-case pathological-anatomy cohort for affected segments against manually segmented ground truth.

**Results:**

Of 8,100 segment observations, 93.8% were accepted without correction, and 51 of 81 segments required no correction in any case. Correction clustered in a few boundary regions, mainly the ischium and femoral neck (median 4.5 segments per case). Computed only on the corrected segments, the median Dice was 0.94, indicating that errors were modest where they occurred. A secondary symmetry analysis showed median surface agreement of 90% within 2 mm and 99.9% within 5 mm. In the pathological-anatomy cohort, pooled median Dice was 0.970 across affected components. Pooled landmark error was 5.37 mm median, with 91.9% of manual clicks inside the automated landmark.

**Conclusions:**

Validated in normal adult anatomy, this rule-based pipeline converts pelvic and proximal femoral CT into a structured hierarchy of clinically relevant subregions, needing only limited, predictable correction. It substantially reduces manual workload while preserving the anatomical detail needed for morphometry and dataset generation. Performance held up in an initial pathological-anatomy sample. Extension to fracture, deformity, or clinical decision-making requires dedicated validation in abnormal anatomy.

**Supplementary Information:**

The online version contains supplementary material available at https://doi.org/10.1186/s12880-026-02753-x.

## Introduction

Pelvic anatomy poses a distinct three-dimensional analytical challenge [1]. CT remains the gold standard for detailed evaluation of the pelvis [2]. Its assessment requires integration of diverse findings, including acetabular measurements, pelvic alignment, bone stock, and symmetry analysis [3–5]. Therefore, segmentation is particularly valuable in pelvic CT, as it converts complex anatomy into structures that can be consistently visualized, localized, and measured [6].

Automated pelvic CT segmentation has been achieved using atlas-based, statistical shape-model-based, and graph-based approaches [7–9]. These methods primarily produced segmentation of whole pelvic bones or limited hip structures [7, 8, 10, 11]. Deep-learning models have enabled multi-class segmentation of pelvic structures including the sacrum and bilateral hip bones [12, 13].

Normal-anatomy segmentation also has standalone value: it underpins statistical shape modeling of the pelvis for surgical planning and shape-variation reference [14]. Moreover, it supports contralateral mirrored-templating techniques already established for pelvic trauma reduction and reconstruction, in which the segmented healthy side serves as the surgical reference for the affected side [15].

Recent advances in CT-based pelvic segmentation have produced task-specific outputs such as coarse pelvic bone masks, fracture-fragment masks, and radiotherapy-oriented pelvic bone contours [16–20]. However, these approaches do not provide a detailed anatomical hierarchy of pelvic and proximal femoral subsegments. Such structured regional mapping could support subregion-specific morphometry, symmetry-based pelvic assessment, and bone-quality mapping [21–24].

The current study addresses this gap by developing a rule-based automated pipeline that transforms parent pelvic and proximal femoral CT masks into a structured subsegmentation of clinically relevant anatomical subregions and landmarks. Validation proceeded in deliberate stages, beginning with normal anatomy: a pipeline’s rule-based logic must first prove reliable under standard conditions before its behavior on pathology-disrupted anatomy can be meaningfully interrogated, since each pathology alters a distinct anatomical region in its own characteristic way. The primary aim was therefore to quantify the manual correction burden required to achieve anatomically acceptable outputs across a large normal adult dataset, complemented by a targeted evaluation of pipeline performance on a pathological-anatomy subset.

## Methods

### Patient selection and CT acquisition

Pelvic CT datasets were retrospectively collected from fully anonymized imaging archives affiliated with the Directorate of Health Affairs in Sharqia, Egypt, under formal authorization for research and healthcare innovation. The datasets were collected between July 2025 and March 2026, using two different CT scanner models: Toshiba Aquilion Lightning and Philips Ingenuity CT (120 kVp; 30–50 mA; slice thickness 0.625–0.789 mm). All data were received anonymized and converted to NIfTI format prior to processing. Demographic stratification was not possible with the available data. All cases were confirmed as adult by radiological absence of open physes.

### Normal-anatomy cohort

**Fig. 1:**
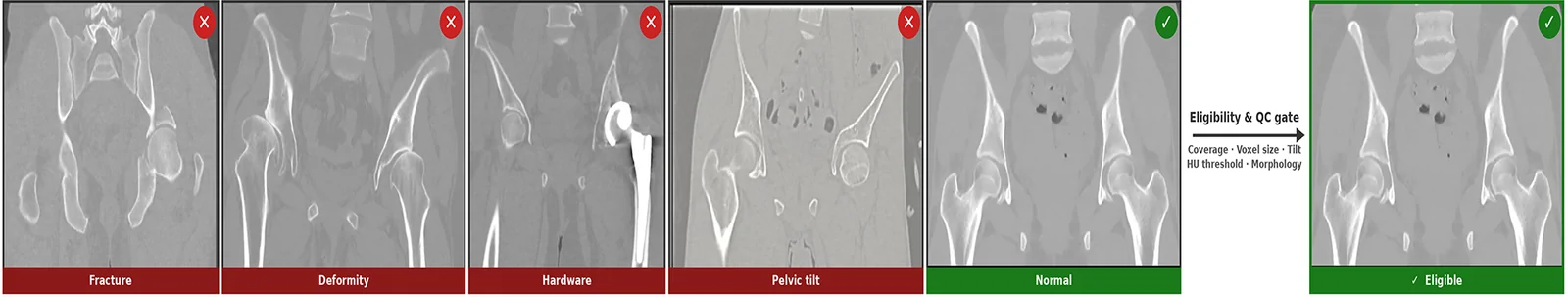
Pelvic CT eligibility and quality-control screening. Representative CT examples show excluded cases and an eligible normal case after assessment of coverage, resolution, bone density, pelvic orientation, and morphology

**Inclusion criteria.** Adult CT normal scans were included if they encompassed the entire pelvis and proximal femora, extending from at least the L5 vertebral level superiorly to below the lesser trochanters inferiorly. Native CT quality required near-isotropic acquisition, with in-plane voxel size ≤ 1.0 mm and slice spacing ≤ 1.25 mm, predefined to preserve reliable 3D reconstruction and segmentation. Cases with marked pelvic malposition on CT were excluded [25]. Malposition was defined as > 5° coronal tilt or > 5° axial rotation and was applied as a study-defined quality criterion, as pelvic orientation can affect CT-based measurements [3]. Bone quality was additionally screened at L5, where mean trabecular attenuation ≥ 160 HU was required as an indicator of sufficient bone density for reliable segmentation. Voxel spacing, pelvic positioning, and L5 attenuation were assessed algorithmically using a custom Python-based workflow implemented in 3D Slicer. Morphological abnormalities, fractures, implants, major imaging artifacts, and eligibility decisions were reviewed by an experienced orthopaedic surgeon (Fig. 1).

**Exclusion criteria.** CT scans were excluded if they were obtained in paediatric patients or showed fracture, major deformity, post-surgical change, metallic implants, severe imaging artifacts, incomplete anatomical extent, or failure to satisfy the predefined technical criteria.

### Pathological-anatomy cohort

Cases excluded from the normal-anatomy cohort for fracture, coxarthrosis, or deformity formed this cohort and were organized into four categories: femoral fracture, pelvic-ring fracture, acetabular fracture, and coxarthrosis/deformity. Cases were not screened against the malposition or L5 bone-density criteria applied above, and were selected solely on pelvic coverage and category membership; the cohort may therefore incidentally include cases with pelvic malposition or tilt. Fifteen cases were randomly selected from each category (60 cases total) to assess pipeline behavior on pathological anatomy.

### Segmentation workflow

**Fig. 2:**
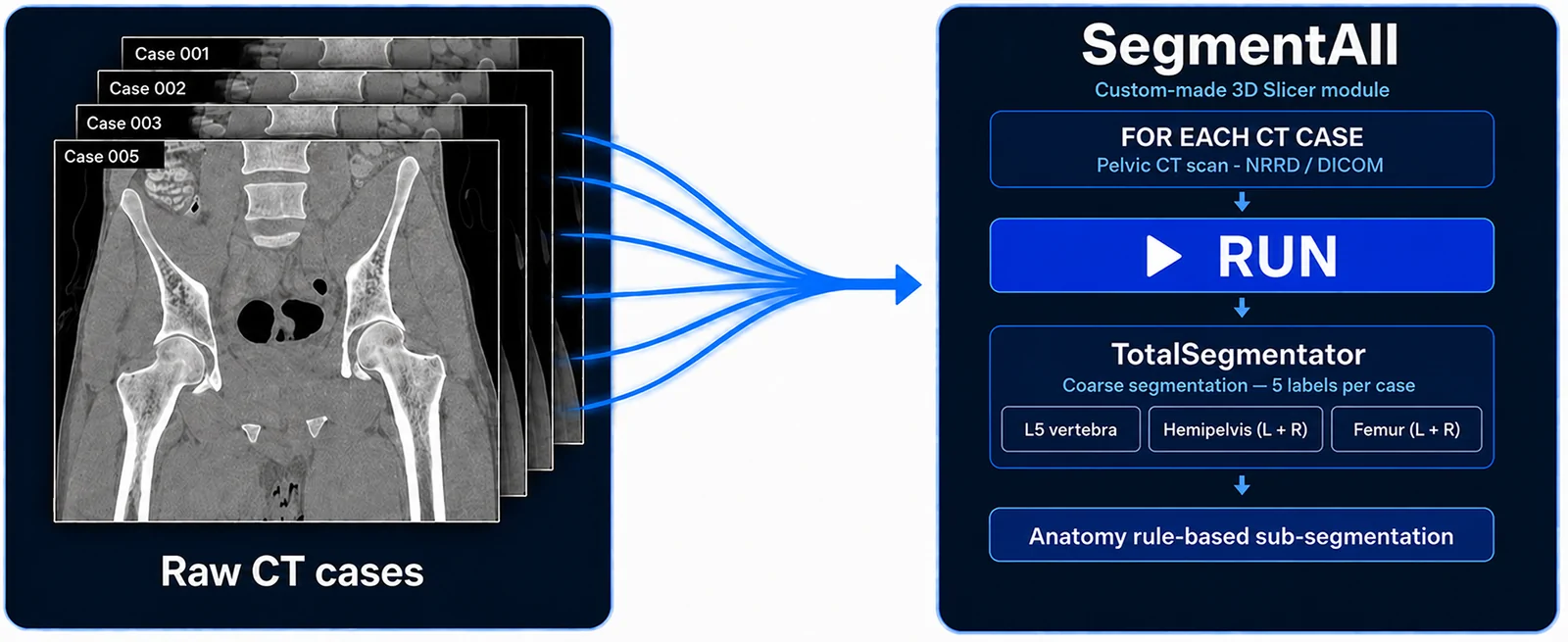
SegmentAll workflow from raw pelvic CTs to the final pelvic and proximal femoral segmentation tree

An end-to-end segmentation pipeline for the pelvis and proximal femur was implemented as a custom 3D Slicer module [26], designed for one-click batch processing of large datasets (Fig. 2). The Slicer environment was chosen to leverage its native CT volume handling, segmentation infrastructure, and spatial processing tools within a single workflow. Manual correction was performed exclusively during reference mask creation for validation and forms no part of the routine processing workflow.

**Fig. 3:**
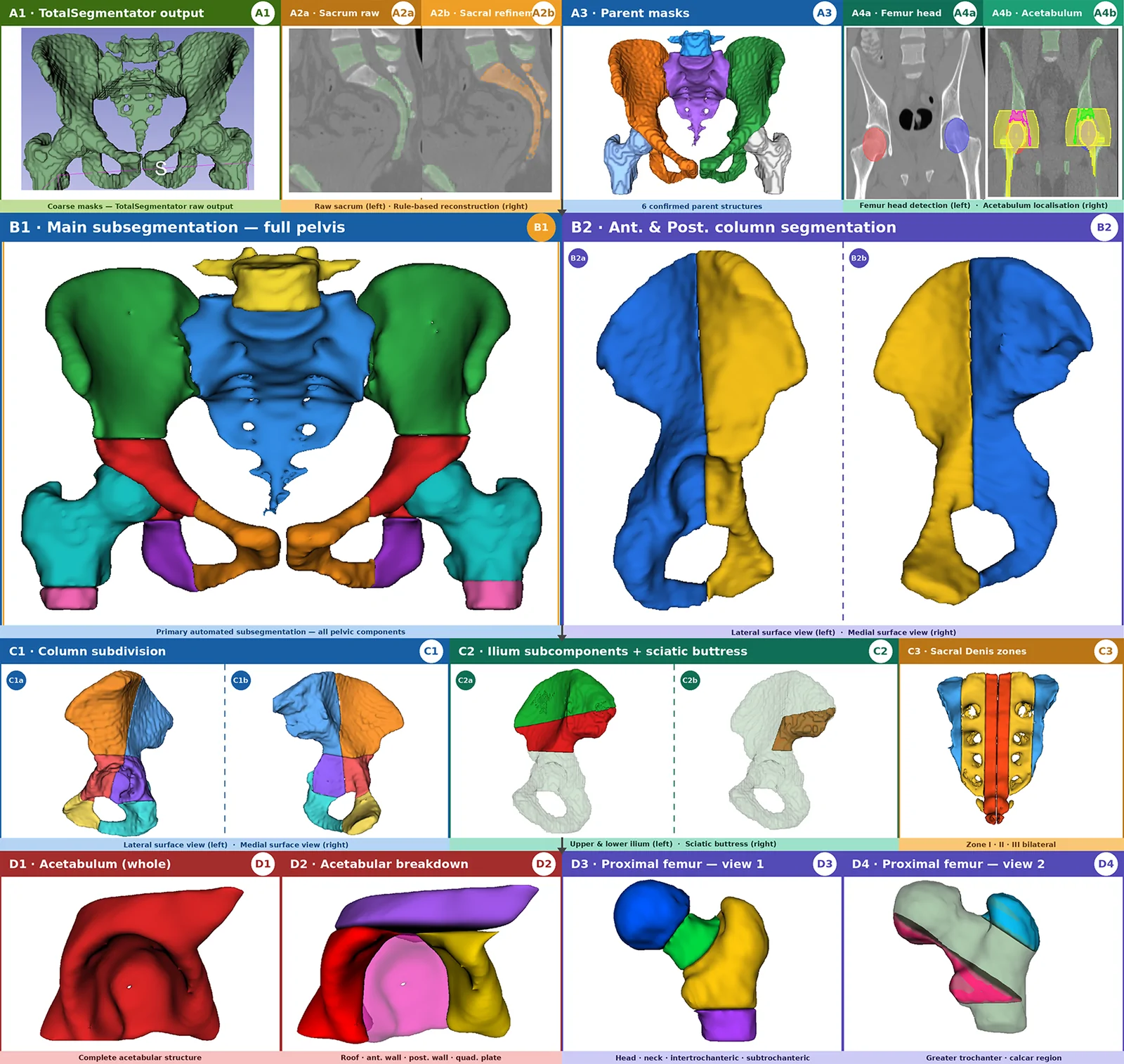
Automated rule-based pelvic CT subsegmentation workflow. (**A1**) TotalSegmentator coarse masks. (**A2**) Sacrum raw output (left) and rule-based reconstruction (right). (**A3**) Six parent structures. (A4) Bilateral femoral head detection (left) and acetabulum localisation (right) on coronal CT. (**B1**) Full-pelvis automated subsegmentation result. (**B2**) Anterior and posterior column segmentation; lateral (left) and medial (right) surface views. (**C1**) Column subdivision; lateral (left) and medial (right) views. (**C2**) Ilium subcomponents — upper and lower ilium (left) and sciatic buttress (right). (**C3**) Sacral Denis zones I–III, bilateral. (**D1**) Complete acetabulum. (**D2**) Acetabular breakdown: roof, anterior wall, posterior wall, and quadrilateral plate. (**D3**) Proximal femur: head, neck, intertrochanteric, and subtrochanteric regions. (**D4**) Proximal femur: greater trochanter and calcar region

**Fig. 4:**
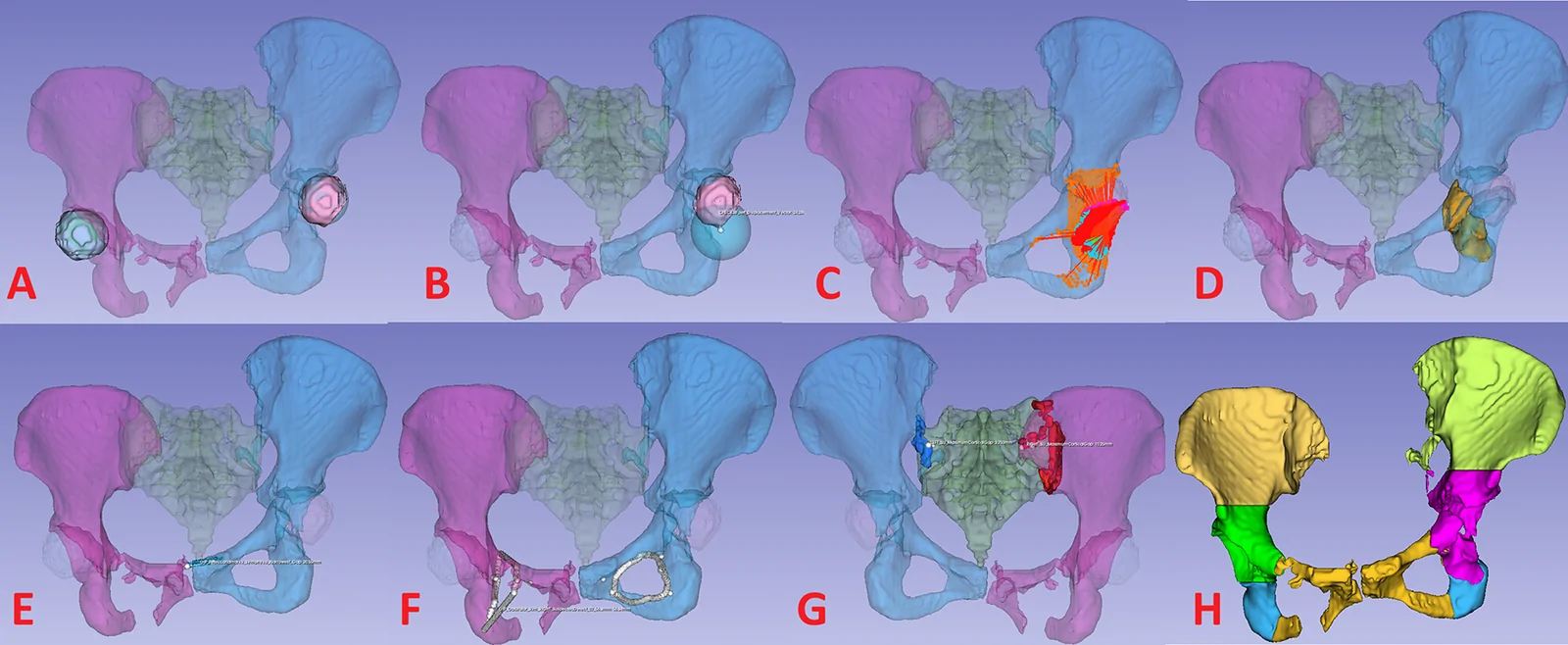
Automated quantitative analysis on a representative acetabular-fracture case (left-sided). (**A**) Bilateral femoral-head sphere fitting. (**B**) Head-socket congruity; left shows an incongruent gap. (**C**) Registered expected socket with displacement vectors, quantifying 24.3 mm superior-posterior head displacement. (**D**) Template defect geometry over the fractured region. (**E**) Pubic symphysis gap measurement. (**F**) Obturator ring discontinuity tracking. (**G**) Sacroiliac joint asymmetry assessment. (**H**) Final bilateral innominate partition into acetabulum, ilium, pubis, and ischium

TotalSegmentator was integrated to identify the major parent structures required for downstream processing, including L5, the sacrum, both hemipelves, and both femora [12]. The initial sacral mask frequently showed incomplete coverage and was therefore refined using a dedicated rule-based reconstruction step. Parent masks were passed automatically into the rule-based subsegmentation workflow without per-case manual inspection; deterministic anatomy-based rules were then applied across the batch to generate the final subsegmented anatomy (Fig. 3). Parent-mask quality was assessed only within the validation subsets, to detect any error that could affect subsequent segmentation results. The pipeline was further adapted to accommodate positional variability introduced by disturbed anatomy, segmenting major affected subregions while flagging apparent abnormalities (Fig. 4). Detailed segmentation logic for individual regions and the anatomical definitions of framework-defined labels are provided in the Supplementary Material.

### Computational performance

Automated processing time was assessed on a subsample of 10 normal-anatomy and 10 pathological-anatomy cases, re-run on a laptop with an Intel Core i7-1265U CPU (10 cores, 12 threads), 32 GB RAM, and integrated Intel Iris Xe graphics (no discrete GPU acceleration used).

### Anatomical design of the segmentation tree

The segmentation hierarchy was designed to reflect orthopedic surgical anatomy rather than generic bone parcellation. The acetabulum is the osseous convergence of the ilium, ischium, and pubis—the three fused components of the hemipelvis [27]. Accordingly, these four structures were defined as the principal pelvic components and then organized into anterior and posterior column divisions, reflecting the structural logic of pelvic surgical anatomy [28]. The hierarchy was then extended to finer surgically relevant regions, for instance, the acetabular walls and quadrilateral plate support assessment of acetabular containment and column integrity [29, 30]. The sciatic buttress and femoral calcar provide pelvic and proximal femoral reference regions for hip load transfer and regional bone stock [31–33]. Denis-based sacral zones convert the sacrum from a single mask into a fracture-oriented map of alar, foraminal, and central involvement [34].

### Segmentation pipeline architecture

The pipeline operates through sequential parent-to-child geometric derivation. Parent masks, the sacrum, bilateral hemipelves, and femora, served as the geometric foundation for all downstream subsegmentation. The femoral head was localized using a best-fit circular reference derived from axial and coronal proximal femoral core masks; its detected center and radius then guided acetabular confinement and extraction. Acetabular subsegmentation was performed within a standardized roof-centered reference frame, partitioning the socket into anterior wall, posterior wall, and quadrilateral plate by positional assignment relative to reproducible anatomical axes. The hemipelvis was divided into anterior and posterior column domains by a deterministic plane connecting the iliac crest apex to the mid-roof point. Each downstream subregion was derived by Boolean operations on parent masks, geometric thresholding, or proportional mediolateral partitioning, with no region boundary drawn by manual delineation. Full definitions and segmentation logic for each region are provided in the Supplementary Methods.

### Landmark generation

Anatomical landmarks were generated automatically within the same subsegmentation pipeline and required no manual input. Each landmark was derived directly from the relevant parent mask using extremal geometry, prominence detection, or midline inference. The ASIS and PSIS were identified as the most anterior and most posterior points of the iliac mask, respectively. The iliac crest apex was extracted as the superolateral extremal point of the iliac mask. The AIIS was localized from the anterior iliac prominence below the crest level. The symphysis pubis was defined as the most anterior midline point between the bilateral hemipelvis masks. The lesser trochanter was identified from the posteromedial prominence of the proximal femoral mask. All landmarks were generated as named segment masks or point-sphere regions and are integral outputs of the segmentation tree, not externally placed fiducials.

### Normal anatomy validation

A randomly selected subset of 100 eligible cases from the final cohort was used for validation. The automated pipeline generated the complete segmentation tree for each case. Segments requiring correction were independently identified by two experienced pelvic surgeons (the authors) across all 100 cases, each blind to the other’s judgment; case-level and segment-level agreement were quantified using percent agreement and Cohen’s kappa. Corrections were then performed by one surgeon and confirmed by the second. Segments were initialized from pipeline output and manually corrected; these corrected working masks reflect expert-audited correction burden rather than an independently generated reference standard.

This validation design constitutes expert-audited correction-burden validation, in which the primary practical endpoint is correction frequency, the proportion of segments requiring expert correction per case. Accepted segments are reported as counts and percentages. Among segments requiring correction, the Dice similarity coefficient (DSC), center-of-mass distance (CMD), average symmetric surface distance (ASSD), and 95th-percentile Hausdorff distance (HD95) were computed from corrected observations only and quantify the actual magnitude of pipeline error.

Secondary validation assessed anatomical plausibility by left–right symmetry analysis after contralateral mirroring and rigid surface registration, summarized by root-mean-square surface distance (RMS) and the proportions of corresponding surface points within 1, 2, 3, 4, and 5 mm.

### Pathological-anatomy performance assessment

To evaluate pipeline performance in the presence of anatomical disruption, four pathological categories were validated: acetabular fracture, pelvic-ring fracture, femoral fracture, and coxarthrosis/deformity, which also included cases of pelvic tumor. For each category, consecutive eligible cases were processed until 15 valid cases were obtained (60 cases total).

For each case, the anatomically affected main component(s), acetabulum, ilium, ischium, pubis, proximal femur/femoral head, or sacrum, were identified from a predefined per-case diagnosis checklist, and a ground truth mask for each affected component was created by manual segmentation performed by the reviewing surgeon.

The automated pipeline output for each affected component was compared against this ground truth using the Dice similarity coefficient (DSC), absolute percentage volume difference (APVD), center-of-mass distance (CMD), average symmetric surface distance (ASSD), and 95th-percentile Hausdorff distance (HD95), computed per category and pooled overall.

Maximum fracture gap was measured for each fracture case using a two-point ruler placed at the site of greatest visible separation between opposing fracture margins on multiplanar CT, and categorized into predefined strata (mild < 5 mm, moderate 5–10 mm, high > 10 mm). A 5 mm gap threshold has been shown to be clinically significant for acetabular fracture outcome, associated with increased risk of conversion to total hip arthroplasty [35], while displacement > 10 mm has been independently associated with worse long-term pelvic-ring outcomes [36].

Landmark accuracy was evaluated within the pathological-anatomy cohort for five landmarks — ASIS, PSIS, lesser trochanter, sacroiliac joint, and symphysis pubis — restricted to landmarks located within or adjacent to the affected component per case. For each landmark, an automated reference point was computed once per case from the corresponding automated segment: the segment centroid for ASIS, PSIS, and the sacroiliac joint, and the most medial extremal point of the segment surface for the lesser trochanter, reflecting its anatomical definition as a projecting bony prominence rather than a volumetric center. A manual point was then placed at the visually identified anatomical landmark position, and deviation was recorded as the Euclidean distance between the manual point and the automated reference. For the symphysis pubis, the surgeon placed a single manual point at the visually identified narrowest point of the symphyseal gap; this point was assessed against two automated references: whether it fell inside the automated gap segment (0 mm if inside, else distance to the nearest gap boundary), and its distance to the pipeline’s own automatically computed narrowest-gap reference point.

### Statistical analysis

Exported validation outputs were consolidated and analyzed using a custom Python script incorporating predefined integrity checks for case count, segment count, primary observations, and secondary symmetry observations. Accepted segments, those retained without expert correction, were reported as counts and percentages at the segment and case levels; no accuracy metrics were computed for these observations. Segments requiring correction were identified by expert visual review (see Validation); only flagged segments were subsequently edited. Correction frequency was calculated as the proportion of segments flagged for correction, at the segment and case levels. Among corrected segments, DSC, CMD, ASSD, and HD95 were computed to quantify the magnitude of correction. Correction burden is therefore characterized jointly by frequency (proportion of segments corrected) and magnitude (severity metrics among corrected segments), rather than by frequency alone. Right and left observations were pooled within predefined bilateral anatomical categories where appropriate. Secondary symmetry results were summarized by RMS and threshold-based surface agreement proportions at the regional level.

For the pathological-anatomy cohort, DSC, APVD, CMD, ASSD, and HD95 were computed by comparing automated output directly against the manually segmented ground truth for each affected component, since no correction step was involved in this cohort; metrics are reported per category and pooled overall. Case-level mean Dice, averaged across all validated targets within a case, was used as the primary summary observation for between-category comparison. Differences in case-level mean Dice across the four pathological categories were assessed using the Kruskal-Wallis test, with pairwise comparisons performed using Mann-Whitney U tests and Holm correction for multiple comparisons. The association between maximum fracture gap and case-level mean Dice was assessed using Spearman correlation; this analysis is reported as exploratory given that it pools cases across heterogeneous anatomical targets.

## Results

A total of 1,316 pelvic CT cases were screened for eligibility. Of these, 559 cases were excluded: 72 pediatric cases, 218 fracture cases (98 femoral, 69 pelvic-ring, and 51 acetabular fractures), 61 cases with metallic implants or hardware, 32 cases with advanced coxarthrosis or relevant pelvic/hip deformity, and 176 cases with technical or eligibility-related imaging limitations caused by incomplete L5-to-lesser-trochanter coverage of required segmentation components, marked osteoporosis, pelvic malposition/rotation, or inadequate image quality. The final dataset comprised 757 normal adult pelvic CT cases, all processed successfully by the automated segmentation pipeline; 100 cases were randomly selected for detailed primary and secondary validation. From the excluded fracture and coxarthrosis/deformity cases, 60 cases were selected across four pathological categories (femoral fracture, pelvic-ring fracture, acetabular fracture, and coxarthrosis/deformity, 15 per category) to assess pipeline performance under pathological anatomy.

Parent-mask quality was inspected across both validation cohorts. No errors were identified in the 100-case normal-anatomy subset. In the 60-case pathological cohort, one case with acetabular dislocation and femoral head–acetabulum overlap required manual correction of the parent mask before automated subsegmentation.

### Expert-audited validation overview — normal-anatomy cases

Across all 100 normal-anatomy validation cases, correction was confined to a minority of observations, concentrated within a predictable anatomical subset rather than distributed across the segmentation tree. Inter-rater agreement between the two independently reviewing surgeons was 91% at the case level and 97.85% at the segment level (Cohen’s κ = 0.781, substantial agreement), supporting the reliability of the underlying correction classifications. The overall acceptance, correction, and reliability profile across all 8,100 case-segment observations is summarized in Table 1.

**Table 1:** Expert-audited validation overview across the 100-case validation dataset

Item	*n*	%
**Total segment observations**	8,100	—
Accepted without expert correction	7,595	93.8
Required expert correction	505	6.2
Segments never requiring correction across all 100 cases	51 of 81	63.0
Segments requiring correction in at least one case	30 of 81	37.0
Cases requiring zero corrections	4 of 100	4.0
Cases requiring correction in ≤ 5 segments	52 of 100	52.0
Cases requiring correction in ≤ 10 segments	96 of 100	96.0
Case-level inter-rater agreement (any correction needed)	91 of 100	91.0
Segment-level inter-rater agreement (Cohen’s κ = 0.781)	7,928 of 8,100	97.85

Validation overview across 8,100 case-segment observations in the 100-case validation dataset. Median corrected segments per case: 4.5 (range 0–19). Values are reported as counts and percentages.

Among segments requiring correction, error magnitude remained moderate across all geometric metrics. Median Dice was 0.94 and remained within clinically acceptable limits (Table 2) [37, 38].

**Table 2:** Accuracy metrics among corrected observations (*n* = 505 of 8,100)

Metric	Mean ± SD	Median [Q1, Q3]
**Corrected observations**,** n**	505 of 8,100	—
Dice	0.8821 ± 0.1471	0.9353 [0.8457, 0.9751]
HD95 (mm)	6.8961 ± 10.0434	5.0000 [2.0254, 8.5701]
ASSD (mm)	1.5769 ± 4.6458	0.6673 [0.2991, 1.6453]
CMD (mm)	3.7943 ± 6.8007	2.1145 [0.8712, 4.5543]

Metrics were calculated exclusively from corrected observations (Dice < 1.0); accepted observations contribute no values to this table. Abbreviations: Dice, Dice similarity coefficient; HD95, 95th-percentile Hausdorff distance; ASSD, average symmetric surface distance; CMD, center-of-mass distance. Values are reported as mean ± SD and median [Q1, Q3].

### Segment-level expert-correction frequency

Stable anatomical reference structures, including the sacrum, bilateral ilium, acetabulum, anterior and posterior columns, sciatic buttress, and all column subcomponents, were accepted without correction in every case. Correction was concentrated in boundary-dependent regions, with the ischium and femoral neck requiring intervention most frequently and showing the greatest geometric error among corrected observations. Acetabular subsegments, sacral zones, and iliac subregions required only occasional or isolated correction (Table 3).

**Table 3:** Segment-level expert-correction frequency across the 100-case validation dataset

Segment	Corrected, *n*	Corrected, %	Mean Dice	Mean HD95 (mm)	Mean ASSD (mm)
Ischium	135	67.5	0.9344	3.5542	0.5188
Femoral neck	82	41.0	0.8235	10.2144	2.5116
Intertrochanteric region	64	32.0	0.9431	6.1140	0.9318
Femoral head	45	22.5	0.9441	5.8172	0.8332
Proximal femur	41	20.5	0.9615	5.1871	0.5651
Subtrochanteric region	37	18.5	0.7665	9.4384	2.5444
Pubis	27	13.5	0.7294	16.6918	4.2516
Greater trochanter	22	11.0	0.9868	1.0942	0.2286
Calcar region	17	8.5	0.6621	11.0916	2.8749
Anterior wall	11	5.5	0.9014	4.2802	0.7032
Posterior wall	10	5.0	0.9151	3.8701	0.7370
Acetabular roof	4	2.0	0.7194	6.1836	1.9282
Lesser trochanter	4	2.0	0.4166	18.9365	13.4503
Quadrilateral plate	4	2.0	0.9776	1.4578	0.2735

A segment was classified as corrected when its Dice coefficient was < 1.0; right and left observations were pooled within each bilateral category. Dice, HD95, and ASSD values reflect corrected observations only. Abbreviations: HD95, 95th-percentile Hausdorff distance; ASSD, average symmetric surface distance. Values are reported as counts, percentages, mean Dice, mean HD95, and mean ASSD.

### Case-level expert-correction burden during validation

The distribution of correction burden across individual cases confirmed that workload remained manageable throughout the validation dataset. The majority of cases required correction in fewer than seven segments, and a small proportion required no correction at all. No case exceeded 19 corrected segments out of 81 analyzed (Table 4).

**Table 4:** Case-level expert-correction burden during validation

Corrected segments per case, *n*	0	1	2	3	4	5	6	7	8	9	10	11	14	19
**Cases**,** n**	4	7	16	13	10	2	18	6	11	6	3	2	1	1

Case-level correction burden was defined as the number of analyzed segments with Dice < 1.0 within an individual validation case, out of a total of 100 validation cases. Corrected-segment counts not shown (12, 13, and 15–18) had zero cases. Values are reported as counts.

### Overall secondary symmetry validation

Secondary symmetry analysis confirmed excellent bilateral agreement. The median RMS was 1.2494 mm [1.0456, 1.5772]. Threshold-based agreement increased progressively with wider tolerance levels, reaching a median of 99.8537% within 5 mm (Table 5).

**Table 5:** Overall secondary symmetry validation metrics

Metric	Mean ± SD	Median [Q1, Q3]
RMS (mm)	1.4840 ± 1.5039	1.2494 [1.0456, 1.5772]
Within 1 mm (%)	63.4942 ± 12.0345	63.0662 [56.0684, 72.4334]
Within 2 mm (%)	88.5457 ± 8.5802	90.4710 [85.2930, 94.2608]
Within 3 mm (%)	95.8609 ± 5.8051	97.7556 [94.9489, 98.9801]
Within 4 mm (%)	98.0124 ± 4.3905	99.3767 [97.7988, 99.8698]
Within 5 mm (%)	98.8264 ± 3.6654	99.8537 [99.0414, 99.9933]

Secondary symmetry metrics were calculated across all regional bilateral comparisons in the 100-case validation dataset; threshold values indicate the percentage of surface points within each distance tolerance. Abbreviation: RMS, root-mean-square surface distance. Values are reported as mean ± SD and median [Q1, Q3].

### Pathological-anatomy segmentation assessment

Segmentation accuracy was broadly consistent across pathological categories and anatomical targets, with pooled median Dice of 0.970 across all 183 evaluations; individual targets ranged from 0.921 to 0.991. Ischium consistently showed the lowest accuracy and widest spread of any target, reflecting its comparatively small size and thin cortical margins. Case-level mean Dice differed significantly across the four pathological categories, driven by lower accuracy in pelvic-ring fracture relative to femoral fracture (Table 6).

**Table 6:** Segmentation performance by anatomical target, pooled across the 60-case pathological-anatomy cohort

Target	Evaluations, *n*	Dice, median (range across categories)	ASSD, mm (range)	HD95, mm (range)	APVD, % (range)
Acetabulum	45	0.967–0.973	0.35–0.39	2.15–2.40	3.85–4.29
Ilium	21	0.985–0.991	0.09–0.21	0.00–0.50	0.79–2.93
Ischium	12	0.921–0.927	0.72–1.02	3.20–4.00	9.19–10.45
Pubis	32	0.935–0.962	0.22–0.71	1.26–4.48	2.33–8.99
Femoral head	31	0.957–0.960	0.44–0.52	2.45–2.91	4.59–5.83
Proximal femur	33	0.984–0.990	0.14–0.16	0.00–1.18	1.94–3.18
Sacrum	9	0.984–0.989	0.15	0.00–0.40	1.68–3.12
**All targets**	**183**	**0.970**	**0.34**	**2.26**	**3.65**

Dice, ASSD, HD95, and APVD are reported as the median value observed in each of the four pathological categories (acetabular fracture, coxarthrosis/deformity, femoral fracture, pelvic-ring fracture), shown here as the lowest–highest value across those four categories. “All targets (pooled)” combines every evaluation across all targets and categories (*n* = 183). n = number of target evaluations for that structure, pooled across the categories in which it was assessed.

#### Between-category comparison

Case-level mean Dice differed significantly across the four pathological categories (Kruskal-Wallis, *p* = 0.036). Pairwise comparison with Holm correction identified femoral fracture versus pelvic-ring fracture as the only significant pair (*p* = 0.048); no other pairwise comparison reached significance. Full pairwise comparison matrix is provided in Supplementary Material.

Landmark error was consistent across categories for each landmark (pooled median 5.37 mm, 91.9% within target across 480 evaluations), indicating that pathology type did not materially affect localization accuracy. PSIS showed markedly lower variance than all other landmarks (SD 0.08 mm versus 2.60–6.05 mm elsewhere), consistent with its sharp, well-defined posterior bony apex, which both the percentile-based automated detection and manual identification reliably converge on (Table 7).

**Table 7:** Landmark accuracy, pooled across pathological categories

Landmark	*n*	Median error, mm	Mean ± SD, mm	Inside target, %
ASIS	120	4.19 (4.14–4.26)	4.98 ± 2.60 (4.77–5.09)	84.2 (73.3–90.0)
PSIS	120	6.88 (6.87–6.89)	6.87 ± 0.08 (6.86–6.88)	100.0
Lesser Trochanter	120	4.84 (4.39–5.50)	6.31 ± 6.05 (5.10–7.45)	91.6 (86.7–93.3)
Sacroiliac Joint	120	5.58 (4.81–6.62)	6.34 ± 3.17 (6.01–6.72)	91.7 (86.7–96.7)
Symphysis Pubis†	60	7.99 (6.78–8.89)	7.17 ± 2.72 (6.90–7.45)	n/a
All landmarks (pooled)*	**480**	**5.37**	**6.13 ± 3.71**	**91.9**

Table 7 Values are median or mean ± SD, pooled across all four categories, with the range across category-level values shown in parentheses; n = evaluations pooled across all four categories (30 per category per landmark, except Symphysis Pubis, 15 per category). “Inside target, %” = the proportion of manual clicks that landed within the automated landmark’s own segment boundary (not the affected-component segment). *“All landmarks (pooled)” combines ASIS, PSIS, Lesser Trochanter, and Sacroiliac Joint (480 evaluations); pooled mean ± SD computed by total-variance decomposition across categories. †Symphysis Pubis is assessed by two independent metrics (gap-segment containment and narrowest-point distance; see Methods) rather than a single inside/outside check, so it is not combined into the pooled row above and “inside target” does not apply to it. Per-category breakdown is provided in Supplementary Material

Maximum fracture gap was measured across the three fracture categories (acetabular, femoral, and pelvic-ring fracture; coxarthrosis/deformity is a non-fracture category and was not assessed for fracture displacement). Mean displacement was 16.3 ± 19.2 mm (acetabular fracture), 15.3 ± 5.8 mm (femoral fracture), and 15.4 ± 11.4 mm (pelvic-ring fracture). Across all fracture cases pooled, displacement magnitude was not significantly correlated with case-level mean Dice (Spearman ρ = − 0.168, *p* = 0.26; exploratory), indicating that correction burden did not scale predictably with fracture gap size within the range evaluated.

### Computational performance

Automated processing time, assessed on random subsamples of 10 normal-anatomy and 10 pathological-anatomy cases, was 8.82 ± 1.36 min per case for normal anatomy (mean ± SD; median 9.12 min; range 6.50–10.02 min) and 8.11 ± 2.44 min per case for pathological anatomy (median 7.13 min; range 4.69–13.20 min), on the hardware specified in Methods.

## Discussion

Automated pelvic CT segmentation has matured to reliable bone extraction, but not yet to the anatomical detail surgeons actually use in practice. In this study, we developed and tested an automated pipeline for detailed pelvic and proximal femoral subsegmentation. Key results were as follows.

1. In normal anatomy, the pipeline produced stable segmentations with a low correction burden, indicating that most structures were captured reliably without expert input.
2. Where correction was needed, it concentrated at small boundary regions between adjacent bones, reflecting the genuine anatomical difficulty of separating continuous bone rather than a general weakness in the pipeline.
3. In pathological anatomy, we evaluated the pipeline’s behavior on the affected component and on automated landmark detection under fracture- or deformity-related disruption, and found performance held up well, suggesting the approach could extend beyond ideal, undisturbed cases.

Manual workflows remain a major barrier in pelvic CT analysis [39, 40]. Previous reports showed that CT-based 3D model segmentation took 90–120 min for each hip joint, while manual CT-based THA planning averaged 185.40 ± 21.76 min per case [39, 40]. Additionally, prior data showed that some pelvic landmarks were frequently mislabeled by 10 mm or more [41]. CT-based pelvic analysis is also challenging in several clinically important and research-oriented tasks [27, 42]. These include pelvimetric extraction, fracture classification, fixation planning, deformity correction, and large-scale generation of structured anatomical data for research use [42, 43]. Together, these limitations underscore the value of automated segmentation for structured and reproducible pelvic CT analysis [44].

Earlier pelvic CT studies established the feasibility of atlas-based segmentation, landmark transfer, and targeted hip-joint extraction [1, 7–10, 45]. Recent CT-based pelvic segmentation work has remained task-specific. Coarse bone-segmentation approaches produced lumbar spine, sacrum, bilateral hemipelves, femoral, or whole-pelvis labels [11, 12, 16, 17]. Trauma-focused pipelines targeted pelvic fracture masks, fragment segmentation, or fracture-reduction planning [4, 13, 19]. Measurement- and landmark-based frameworks automated selected outputs such as 3D pelvimetry, acetabular lunate-surface analysis, or landmark detection in defective pelvis CTs [5, 6, 42]. Therefore, current literature supports automated pelvic CT analysis for defined tasks, but a unified fully automated subsegmentation framework of normal pelvic and proximal femoral subregions remains insufficiently established.

In the current study, subsegmentation was reframed from coarse bone-mask extraction into an orthopaedic anatomical mapping system. Each output region was selected and defined according to anatomical units with direct clinical applicability in pelvic assessment and surgical planning. This segmentation design is particularly relevant for future fracture mapping, regional morphometry, and planning-oriented CT analysis, where interpretation depends on precise anatomical location rather than whole-bone volume alone.

This study’s validation strategy was designed to match each cohort’s clinical question. Correction-burden classification for the normal-anatomy cohort clarifies pipeline quality broadly, exposing strengths and weaknesses across the full anatomical tree, for example identifying that small junctional segments require manual control, which flags these segments as candidates for direct comparison against a manual reference mask; this approach is more scalable for fine subsegmentation than constructing such a reference for all 8,100 segment-observations. In the pathological-anatomy cohort, comparing the affected component directly against an independent ground truth was the more reliable approach, since disruption manifests primarily at the site of pathology, also the structure most relevant to surgical planning. Pooling unaffected segments would dilute this comparison, as their performance mirrors the normal-anatomy cohort and would mask genuine accuracy differences at the affected site. Landmark accuracy was evaluated using the same logic: surgical planning applications of these landmarks, including fixation-trajectory and contralateral-reference planning, apply mainly to trauma and deformity cases, so accuracy was assessed in the pathological-anatomy cohort, where robustness under anatomical disruption is the clinically relevant test.

Pelvic CT segmentation remains boundary-sensitive even before fine subsegmentation. Recent work has described challenges related to small targets, thin boundaries, and separation of adjacent hip bone segments [13]. In the present study, stable reference structures, including the sacrum, ilium, acetabulum, anterior and posterior columns, sciatic buttress, and all column subcomponents, required no expert correction in any of the 100 normal-anatomy validation cases. Among femoral and acetabular subsegments, the femoral head, greater trochanter, acetabular walls, and dense sacral compartments were accepted without correction in most cases. Correction was confined to boundary-dependent or bridging regions, principally the femoral neck, ischium, pubis, subtrochanteric region, calcar region, and intertrochanteric region, where anatomical continuity makes discrete voxel-level separation inherently less deterministic. This boundary profile explains why correction frequency was concentrated in a small subset of transition-related segments rather than distributed uniformly across the anatomical tree. Several boundaries in this framework are defined by anatomical rules of inherent geometric precision, applied identically across every case. The acetabular roof, for instance, corresponds to the superior weight-bearing portion of the lunate articular surface [46] identified in the pipeline as the superior acetabular surface at the level where femoral head contact first disappears. Here the anatomical rule is the definition, and its consistent execution across cases represents a standard of reproducibility that freehand delineation does not achieve.

The primary question this study addresses is practical: to what extent does the pipeline reduce manual segmentation workload in pelvic CT analysis. Correction frequency is therefore the central endpoint, reflecting how many segments required expert intervention and how large the remaining errors were. Across 100 validation cases, 93.8% of segments were accepted without correction, and correction where required was moderate in magnitude and confined to a predictable anatomical subset. Median Dice among corrected segments in this study (0.94) meets or exceeds Dice values reported as indicating good-to-excellent performance in pelvic and general bone CT segmentation, ranging between 0.86 and 0.93 [37, 38]. Comparable studies at this level of anatomical detail are limited in the current literature, so direct segment-level comparison is not possible. Available literature reports Dice at the coarser level of whole-bone or fracture-fragment segmentation. Under that coarser definition, the PENGWIN 2024 challenge reported a top fragment-wise Intersection over Union of 0.930 across 150 multi-center CT scans [47], and Zeng et al. reported Dice of 0.935–0.955 for pelvic fracture fragments [48]. Correction here was concentrated in small, boundary-dependent junctions (femoral neck, ischium, pubis, intertrochanteric region) rather than large, discretely separable fragments — a harder comparison basis, since fine internal boundaries lack the natural discontinuities that ease fracture- or whole-bone-level segmentation, where fragment identification relies primarily on dislocation rather than subtle image cues [48]. These findings indicate that the pipeline generates anatomically acceptable pelvic and proximal femoral subsegmentations in most cases, with a limited and predictable correction burden.

The acetabulum and femoral head are among the most challenging structures in automated CT segmentation, due to narrow inter-bone joint spaces, deformed shapes, and weak, diffused boundaries [45]. Reported acetabulum-segmentation targets and metrics vary widely: Hopkins et al. used neural-network hemipelvis segmentation with statistical shape modeling to derive bone-loss volume, reporting Dice 0.827 for the defect region and 0.948 for the hemipelvis [49]; Liu et al. isolated the acetabulum by removing the femoral head slice-by-slice, reporting pixel error rates (1.0–5.9% missing, 1.2–8.7% faulting) and surface deviation (± 1.9–2.4 mm) but no Dice [50]; Cheng et al. segmented femoral head and acetabulum as one combined mask, reporting Dice 91.55% ± 4.82 (81.83–95.09% across pathology severity, 110 hips) [45].

In the present study, the acetabular zone was defined as the socket level inclusive of the anterior and posterior walls with their columnar extension, to support downstream trauma applications such as column/wall fracture mapping. Across the 60-case pathological-anatomy assessment, median Dice was 0.967–0.973 for the acetabulum and 0.957–0.960 for the femoral head. Among the studies reviewed here, none reports Dice for this same anatomical target, so these figures are not directly comparable to the values above.

Landmark localization in pelvic CT is similarly affected by anatomical variability and, in pathological cases, by fracture- or deformity-related disruption. Hêches et al. used reference-model registration to automatically label 12 pelvic landmarks in normal-anatomy CT, comparing against radiologist manual labeling; median error was 2.3–11.6 mm manually and 1.9–6.3 mm automatically [41].

In the present study, pooled error across five landmarks in the 60-case pathological-anatomy assessment was 5.37 mm median, with 91.9% of manual clicks inside the automated landmark’s segment — within Hêches et al.‘s automatic-method range despite pathological anatomy. The comparison is approximate, given differing landmark sets and anatomy type.

Symmetry analysis served as a secondary plausibility check for segmentation consistency. In the present study, contralateral mirroring and rigid surface registration were applied across 100 CT cases and paired regions including the acetabulum, femoral head, ilium, innominate, ischium, proximal femur, pubis, and anterior and posterior columns; median surface agreement reached 90.47% within 2 mm and 99.85% within 5 mm. These findings are consistent with previous 3D studies of intact pelvic symmetry [51–53]. Ead et al. analyzed 14 pelvic CT scans using Mimics-based 3D reconstruction and Geomagic deviation analysis, reporting an RMS deviation of 1.14 ± 0.26 mm and 91.9 ± 5.55% of surface points within ± 2 mm [51]. Zheng et al. analyzed 50 pelvic CT scans using Mimics, Geomagic, and closest-point alignment, reporting a mean surface deviation of 1.15 ± 0.16 mm and 90.82 ± 4.67% of points within the permissible ± 2 mm range [52]. Bakhshayesh et al. analyzed ten healthy adult CT scans using STL reconstruction, hemipelvis mirroring, volume fusion, and CT motion analysis, showing that right–left differences remained within approximately 3 mm translation and 2° rotation [53]. These studies establish bilateral pelvic symmetry as a reliable reference concept; the current study extends this concept from whole-pelvis comparison to region-level segmentation, supporting its use for side-to-side quality control, normal-variation assessment, and detection of abnormal asymmetry.

Beyond validation, the segmentation framework was integrated into a custom one-click 3D Slicer workflow to assess its feasibility for quantitative analysis and clinically relevant secondary applications. These included automated regional bone-quality assessment in predefined load-bearing areas and selected pelvic and proximal femoral morphometric measurements. The pathological-anatomy results reported here support extending this framework toward fracture- and deformity-related applications, including fixation and arthroplasty planning. Dedicated validation at the level of these specific downstream tasks is still required. The same framework may also support structured annotation of abnormal or excluded cases. Automated segmentation followed by selective manual refinement, using AI-assisted interactive labeling frameworks [54], could accelerate the creation of curated datasets for future deep-learning models. These outputs remain extensions of the core pipeline and represent planned directions for follow-up studies; each requires application-specific validation before clinical use. Technical details for these secondary applications are provided in the Supplementary Material.

### Strengths and limitations

This automated pelvic CT framework moves beyond coarse bone segmentation or single-task measurement, providing an anatomy-based orthopedic subsegmentation map of the pelvis and proximal femur, validated through expert-audited correction-burden analysis across 100 normal-anatomy cases, with an initial extension to a 60-case pathological-anatomy assessment. Pelvic CT segmentation is treated as a structured anatomical framework, preserving clinically meaningful regions and landmarks rather than generating isolated masks.

The primary limitation of the validation design is that reference masks were initialized from pipeline output and corrected where needed, rather than generated independently from scratch; results should therefore be interpreted as expert-audited anatomical acceptability and correction burden, not conventional independent agreement. Correction execution was performed by one surgeon and confirmed, not independently repeated, by the second, so residual variability in the corrected mask itself cannot be fully excluded. In the routine 757-case pipeline, parent masks passed automatically into subsegmentation without per-case manual inspection; quality was assessed only within the 100- and 60-case validation subsets.

A further limitation is that the pipeline’s full 81-segment tree was validated exclusively in normal adult CT scans; performance in fractures, deformity, severe degeneration, implants, or marked morphological alteration remains unproven at that level of granularity and requires dedicated evaluation before application to abnormal cohorts or clinical decision-making. The 60-case pathological-anatomy assessment validated only the major affected component(s) per case against manual ground truth, acetabulum, ilium, ischium, pubis, proximal femur/femoral head, or sacrum, not the full fine subsegmentation tree. Pipeline behavior at the level of fine boundary-dependent subsegments under pathological disruption remains unvalidated and is a direction for future work. Age and sex were not available in the anonymized dataset, and generalizability beyond the two identified scanner models is unconfirmed.

## Conclusion

This study presents a rule-based automated pipeline that transforms pelvic and proximal femoral CT into a structured, orthopaedically meaningful map of anatomical subregions and landmarks. Correction burden was low and concentrated in a predictable, boundary-dependent subset, rather than distributed across the anatomical tree. Performance held up on an initial pathological-anatomy sample, extending beyond ideal, undisturbed cases. The framework offers a reusable foundation for morphometric analysis and structured dataset generation. Larger-scale, dedicated validation in abnormal anatomy is required before extension to fracture, deformity, or clinical decision-making workflows.

## Supplementary Information

Below is the link to the electronic supplementary material.

Supplementary Material 1

Supplementary Material 2

Supplementary Material 3

Supplementary Material 4

Supplementary Material 5

## Data Availability

The datasets analyzed and generated during the current study are not publicly available because they consist of retrospectively collected fully anonymized medical imaging data obtained under local authorization and subject to institutional data-governance and data-protection requirements. The datasets will be stored securely at the authors’ institution for 10 years in accordance with institutional data-retention policy. Data may be made available from the corresponding author upon reasonable request and subject to applicable ethical, institutional, and data-protection approvals.

## Abbreviations

AIIS: Anterior inferior iliac spine
APVD: Absolute percentage volume difference
ASSD: Average symmetric surface distance
ASIS: Anterior superior iliac spine
CMD: Center—of—mass distance
DSC: Dice similarity coefficient
HD95: 95th—percentile Hausdorff distance
HU: Hounsfield unit
PSIS: Posterior superior iliac spine
RMS: Root—mean—square surface distance
THA: Total hip arthroplasty
