# Supplementary material for: Anatomy-driven automated subsegmentation of pelvic and proximal femoral CT into clinically relevant subregions and landmarks
Source: BMC Med Imaging. 2026 Sep 8;26:454. doi: 10.1186/s12880-026-02753-x (PMC13584298; doi:10.1186/s12880-026-02753-x)
Supplement: Supplementary file 1 [file 12880_2026_2753_MOESM5_ESM.pdf]

Pelvic CT Segmentation and Quantitative Anatomical Analysis

Clinical Findings Summary

Case ID: ACETFX_0004_0000 | Age: Not supplied | Status: VALID

CONCLUSIVE FINDING

Automated CT analysis flags Left head-socket suspected dislocation; low acetabular coverage on Left; acetabular discontinuity of at least 5 mm; and 4 additional flagged finding(s). Femoral parameter assessment is flagged; see the dedicated evidence page.

OVERALL STATION ASSESSMENT

Flagged finding of at least 5 mm in the hip/acetabular, anterior-ring station group(s).

Clinical station checklist

HIP JOINT AND ACETABULUM

Femoral-head morphology — NO FLAG
R VALID HEAD CENTRE — MORPHOLOGY CAUTION / L VALID HEAD CENTRE — MORPHOLOGY CAUTION

Head-socket congruity — FLAGGED
R CONGRUENT / L SUSPECTED DISLOCATION (SUPERIOR + POSTERIOR), 24.3 mm

Acetabular coverage — FLAGGED
R 40.8% / L 22.1%; low coverage LEFT

Acetabular continuity — FLAGGED
3 accepted cluster(s); maximum 3-D span 9.2 mm; 5 mm or larger discontinuity finding

Head position / dislocation — FLAGGED
LEFT 24.3 mm; SUPERIOR + POSTERIOR

Joint-space / arthrosis screen — NOT ASSESSED
True joint-space status not published by the applicable route

Femur parameters — FLAGGED
Automated femoral analysis flags right 3D NSA above Hartel true-3D CT IQR; left 3D NSA below Hartel true-3D CT IQR; bilateral femoral-offset asymmetry ≥5 mm; right head-centre-to-lesser L/D below the

ANTERIOR PELVIC RING

Overall anterior ring screen — FLAGGED
Discontinuity finding of at least 5 mm at an anterior-ring station

Pubic symphysis — FLAGGED
Gap 21.3 mm; SUSPECTED TRAUMATIC SYMPHYSEAL DIASTASIS

Right obturator ring — FLAGGED
2 candidate(s); maximum bridge chord 56.3 mm; 5 mm or larger discontinuity

Left obturator ring — NO FLAG
0 reportable rim discontinuity candidates

POSTERIOR PELVIC RING

Right SIJ integrity — NO FLAG
median 3.2 mm; P95 7.8 mm; WIDER THAN AGE REFERENCE

Left SIJ integrity — NO FLAG
median 0.2 mm; P95 0.2 mm; NARROWER THAN AGE REFERENCE

SCOPE: Joint-space narrowing is an arthrosis/coxarthrosis screen, not a stand-alone diagnosis. These station tests do not exclude isolated or minimally displaced iliac or sacral fractures; review the source CT.

SIJ references: normal CT mean 2.3 +/- 0.4 mm (<40 y) and 1.9 +/- 0.2 mm (>40 y) [8]; traumatic axial/coronal distance context [9]; anatomical extent/search-corridor context [17].

Detailed anatomical evidence and quantitative measurements follow on the next pages.

Pelvic CT Segmentation and Quantitative Anatomical Analysis

1. Femoral-Head Morphology and Centre

RIGHT FEMORAL HEAD: VALID HEAD CENTRE — MORPHOLOGY CAUTION; CENTRE MODERATE CONFIDENCE. LEFT FEMORAL HEAD: VALID HEAD CENTRE — MORPHOLOGY CAUTION; CENTRE MODERATE CONFIDENCE.

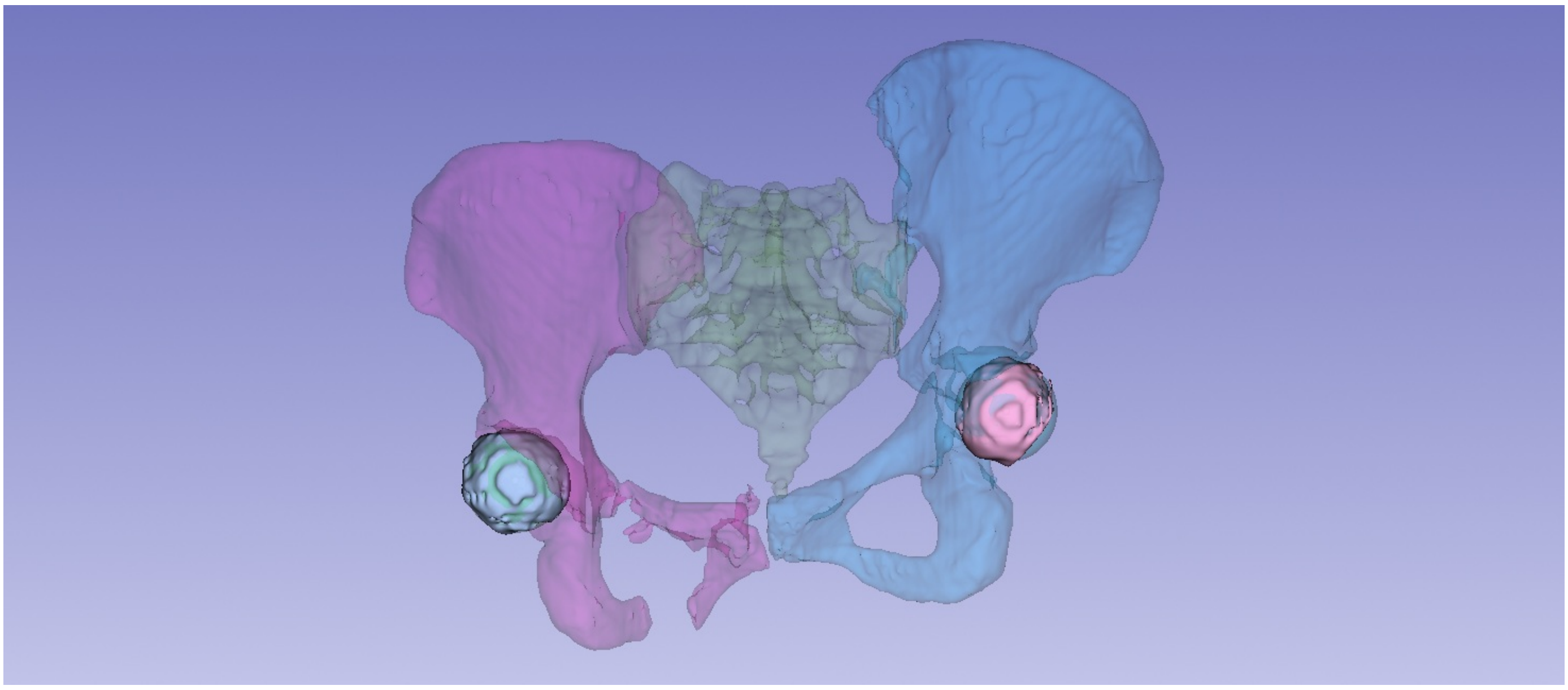

MEASUREMENT	RESULT	REFERENCE / COMPARATOR	INTERPRETATION
Right fitted radius	**20.3 mm**	Adolescent radial-CT comparator: male 24.53 +/- 1.74 mm; female 21.94 +/- 1.13 mm [10]	VALID HEAD CENTRE — MORPHOLOGY CAUTION
Left fitted radius	**20.0 mm**	Adolescent radial-CT comparator: male 24.53 +/- 1.74 mm; female 21.94 +/- 1.13 mm [10]	VALID HEAD CENTRE — MORPHOLOGY CAUTION
Sphere-fit residual	**R/L P95 2.1 mm/2.6 mm**	Published 3-D sphere/asphericity analysis supports the measurement family; these residual limits are study-specific [11]	Quantitative morphology QC

Best-fit sphere radius and residuals are reported from the segmented femoral head. Published 3-D CT/asphericity work supports this measurement family, while the residual QC limits are study-specific [10,11].

Pelvic CT Segmentation and Quantitative Anatomical Analysis

Femur Subsegmentation and Quantitative Parameters

Automated femoral analysis flags right 3D NSA above Hartel true-3D CT IQR; left 3D NSA below Hartel true-3D CT IQR; bilateral femoral-offset asymmetry ≥5 mm; right head-centre-to-lesser L/D below the published interval; left head-centre-to-lesser L/D above the published interval.

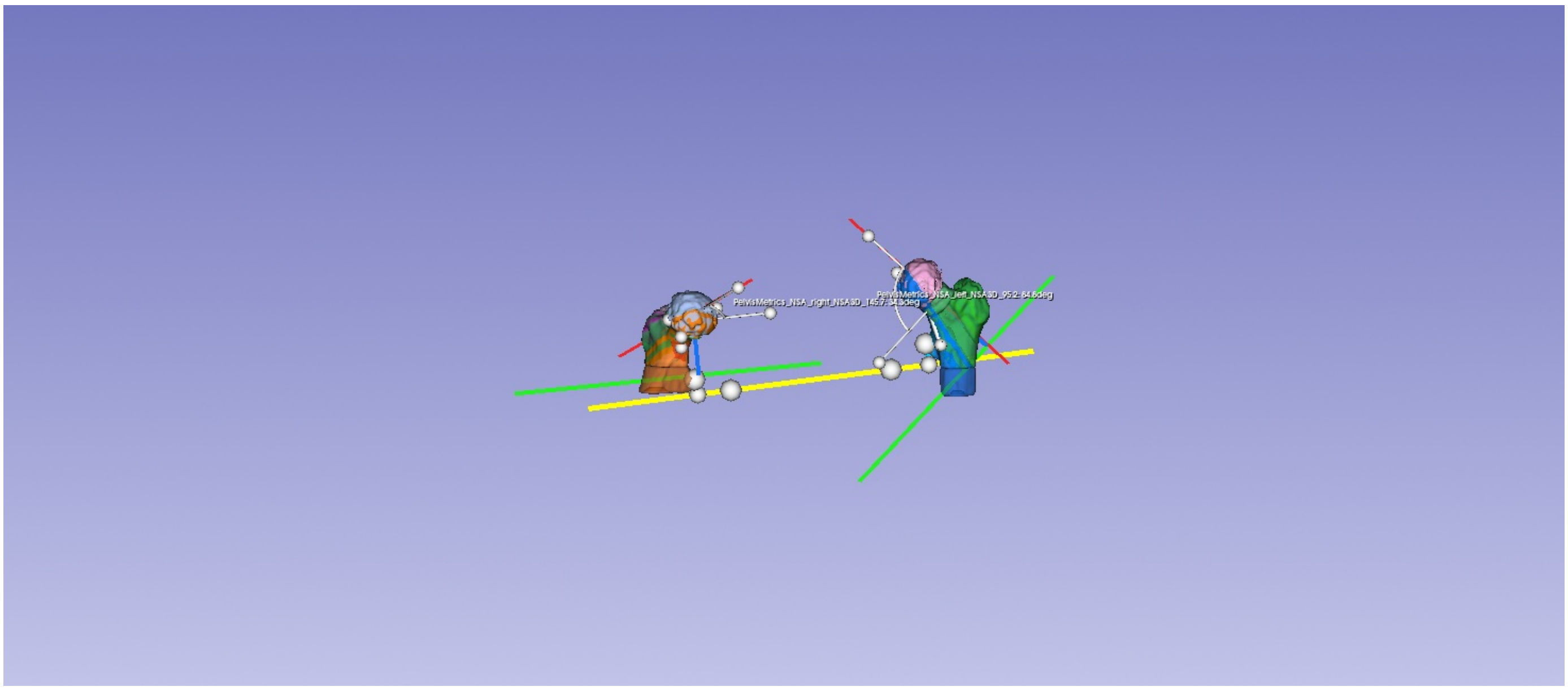

MEASUREMENT	RESULT	REFERENCE / COMPARATOR	INTERPRETATION
True-3D neck-shaft angle (R / L)	**145.75 / 95.23 deg**	Hartel CT IQR 117.9-125.6 deg [18]	FLAGGED HIGH / FLAGGED LOW
NSA absolute bilateral difference	**50.5 deg**	Quantitative side comparison; not a stand-alone diagnosis [18]	FLAGGED
Femoral offset (R / L)	**51.15 / 77.05 mm**	Absolute native CT values are quantitative only [19]	FLAGGED ASYMMETRY
Offset absolute side difference	**25.9 mm**	>=5 mm reconstruction-asymmetry comparator [19]	FLAGGED
Limb-length discrepancy	**0.5 mm**	>=10 mm comparator [20]	WITHIN 10MM COMPARATOR; Left shorter
Head-centre-to-lesser L/D — Right	**0.561**	AP-radiographic mean 0.84 +/- 0.08; screening interval 0.68-1.00 [21]	L/D is below the published screening interval; this alone does not diagnose proximal-femoral collapse.
Head-centre-to-lesser L/D — Left	**1.330**	AP-radiographic mean 0.84 +/- 0.08; screening interval 0.68-1.00 [21]	L/D is above the published screening interval; review the lesser point and head geometry.
Integrated femur assessment	**FLAGGED**	Automated quantitative screening; CT/source-segmentation confirmation required	Automated femoral analysis flags right 3D NSA above Hartel true-3D CT IQR; left 3D NSA below Hartel true-3D CT IQR; bilateral femoral-offset asymmetry ≥5 mm; right head-centre-to-lesser L/D below the published interval; left head-centre-to-lesser L/D above the published interval.

The displayed femur subsegments preserve the locked RAS geometry and existing lesser-trochanter result. NSA and offset use the corrected medullary-centre shaft axis; LLD uses bilateral lower-innominate reference geometry. L/D uses the femur-local AP projection (posterior component excluded). Published comparators are screening references, not stand-alone diagnoses [18-21].

Pelvic CT Segmentation and Quantitative Anatomical Analysis

2. Native Head-Socket Shell and Congruity

RIGHT MARGIN CONTINUOUS; INTACT CONTACT MARGIN; CONGRUENT. LEFT MARGIN SPLIT; SPLIT FRACTURE; SUSPECTED DISLOCATION, SUPERIOR + POSTERIOR -- CT-confirmed 24.3 mm displacement relative to the registered expected socket (see section 6).

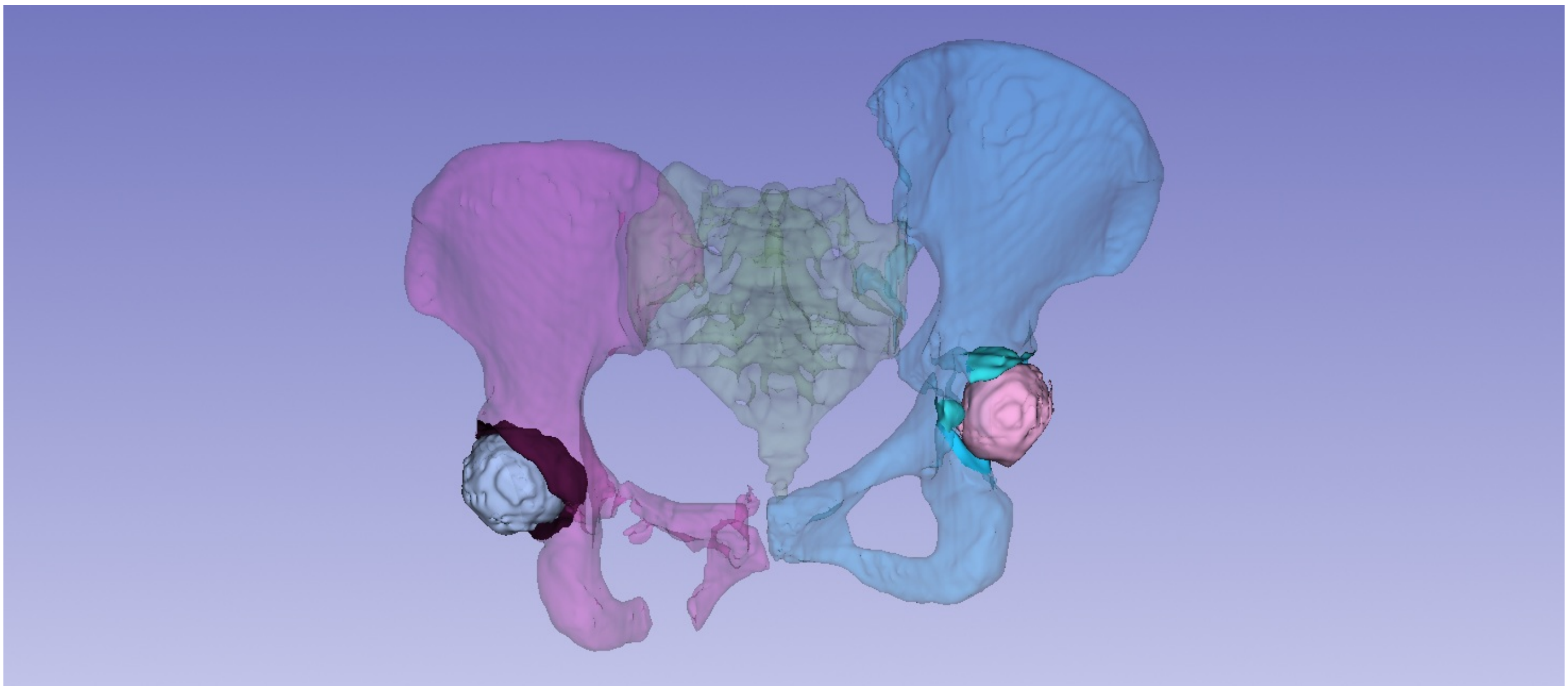

MEASUREMENT	RESULT	REFERENCE / COMPARATOR	INTERPRETATION
Right local gap (median / P95)	**2.7 mm / 6.0 mm**	Healthy-control whole-joint WBCT JSW 3.99 +/- 0.53 mm; different acquisition and geometry [12]	CONGRUENT
Left local gap (median / P95)	**5.5 mm / 6.8 mm**	Healthy-control whole-joint WBCT JSW 3.99 +/- 0.53 mm; different acquisition and geometry [12]	SUSPECTED DISLOCATION, SUPERIOR + POSTERIOR (24.3 mm CT-confirmed)

Head-centred rays assess acetabular-shell continuity and local gap. The cited weight-bearing CT joint-space value is shown only as context because the acquisition and geometry differ [12].

Pelvic CT Segmentation and Quantitative Anatomical Analysis

3. Midsagittal Plane and Central-Bone QC

MIDSAGITTAL PLANE QC: PASS — BOTH CENTRAL BONES ARE NEAR-EQUAL; L5 5.59%; SACRUM 3.61%.

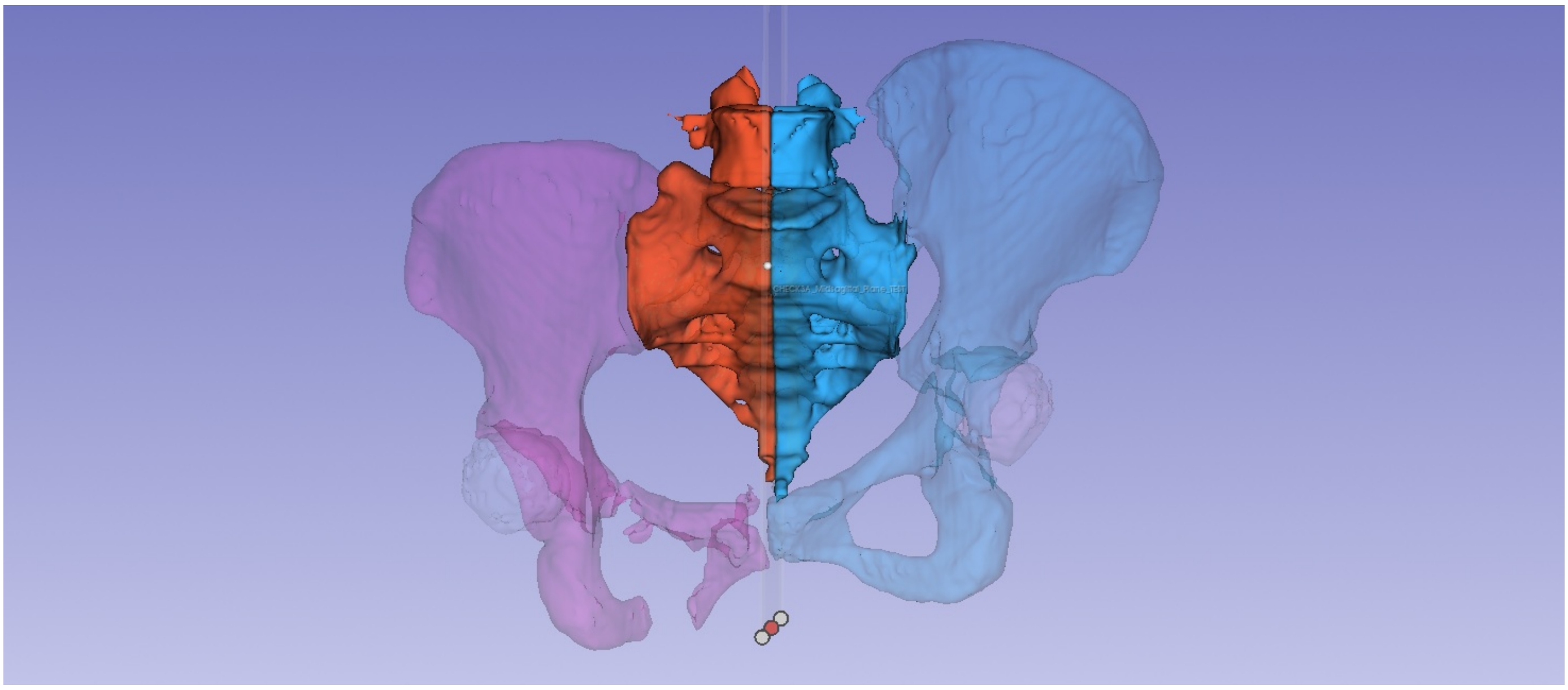

MEASUREMENT	RESULT	REFERENCE / COMPARATOR	INTERPRETATION
L5 bilateral half-volume difference	**5.59%**	Study-internal geometric QC	NEAR-EQUAL
Sacral bilateral half-volume difference	**3.61%**	Study-internal geometric QC	NEAR-EQUAL
Overall plane QC	**PASS — BOTH CENTRAL BONES ARE NEAR-EQUAL**	10% bilateral difference is a study-internal QC rule	Technical plane validation; not a clinical diagnostic cutoff

The pelvic midsagittal plane is accepted only after bilateral L5/sacral half-volume QC. The 10% difference rule is a study-internal geometric QC criterion, not a clinical threshold.

Pelvic CT Segmentation and Quantitative Anatomical Analysis

4. Global Femoral-Head Coverage

GLOBAL FEMORAL-HEAD COVERAGE: RIGHT 40.8%; LEFT 22.1%.

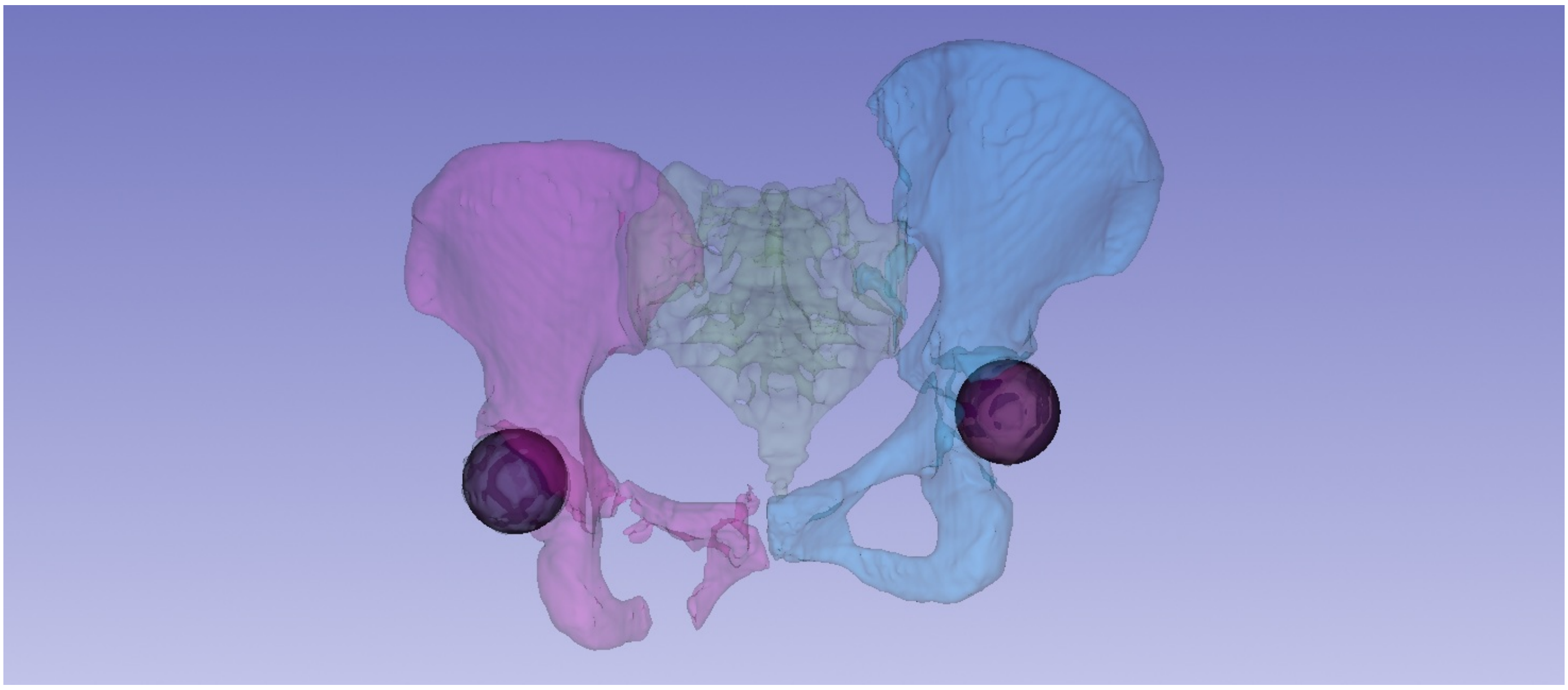

MEASUREMENT	RESULT	REFERENCE / COMPARATOR	INTERPRETATION
Right global coverage	**40.8%**	Asymptomatic CT mean 40 +/- 2%; calculated mean +/-2SD approximately 36-44% [5]	Z=+0.38 | WITHIN APPROXIMATE ASYMPTOMATIC MEAN+/-2SD
Left global coverage	**22.1%**	Asymptomatic CT mean 40 +/- 2%; calculated mean +/-2SD approximately 36-44% [5]	Z=-8.96 | BELOW APPROXIMATE ASYMPTOMATIC MEAN-2SD

Global curved-surface coverage is measured on a best-fit femoral-head sphere using radial intersection with the acetabular shell. This is an automated adaptation of Larson's method; 40 +/- 2% is the published comparator [5].

Pelvic CT Segmentation and Quantitative Anatomical Analysis

6. Registered Expected Native Socket and Head Displacement

LEFT FEMORAL-HEAD DISPLACEMENT: 24.3 mm, SUPERIOR + POSTERIOR; CT-CONFIRMED DISPLACED RELATIVE TO REGISTERED EXPECTED SOCKET.

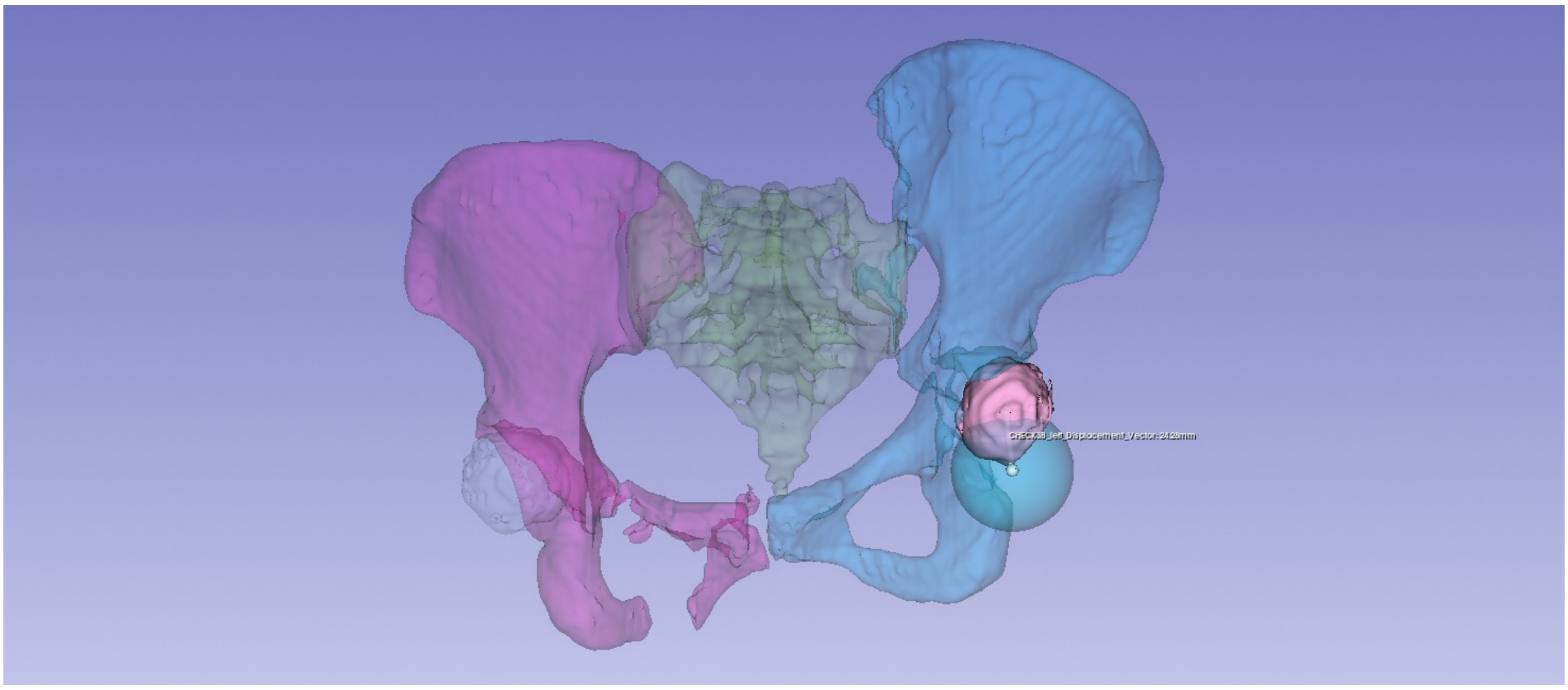

MEASUREMENT	RESULT	REFERENCE / COMPARATOR	INTERPRETATION
3-D displacement	**24.3 mm**	Healthy controls: 1.3 +/- 0.6 mm head-centre translation during external-rotation stress WBCT; not equivalent to routine trauma CT [12]	DISPLACED RELATIVE TO REGISTERED EXPECTED SOCKET
Dominant direction	**SUPERIOR + POSTERIOR**	Direction is reported directly from locked Check3B; no ReportBuilder RAS recomputation.	INCONGRUENT

Displacement is reported from the actual head centre relative to the registered expected native socket centre. Direction is taken directly from the locked source analysis.

Pelvic CT Segmentation and Quantitative Anatomical Analysis

7. Registered Template Defect Geometry

LARGE SUPERIOR–POSTERIOR SPLIT FRACTURE; MEDIAL-WALL DEFECT.

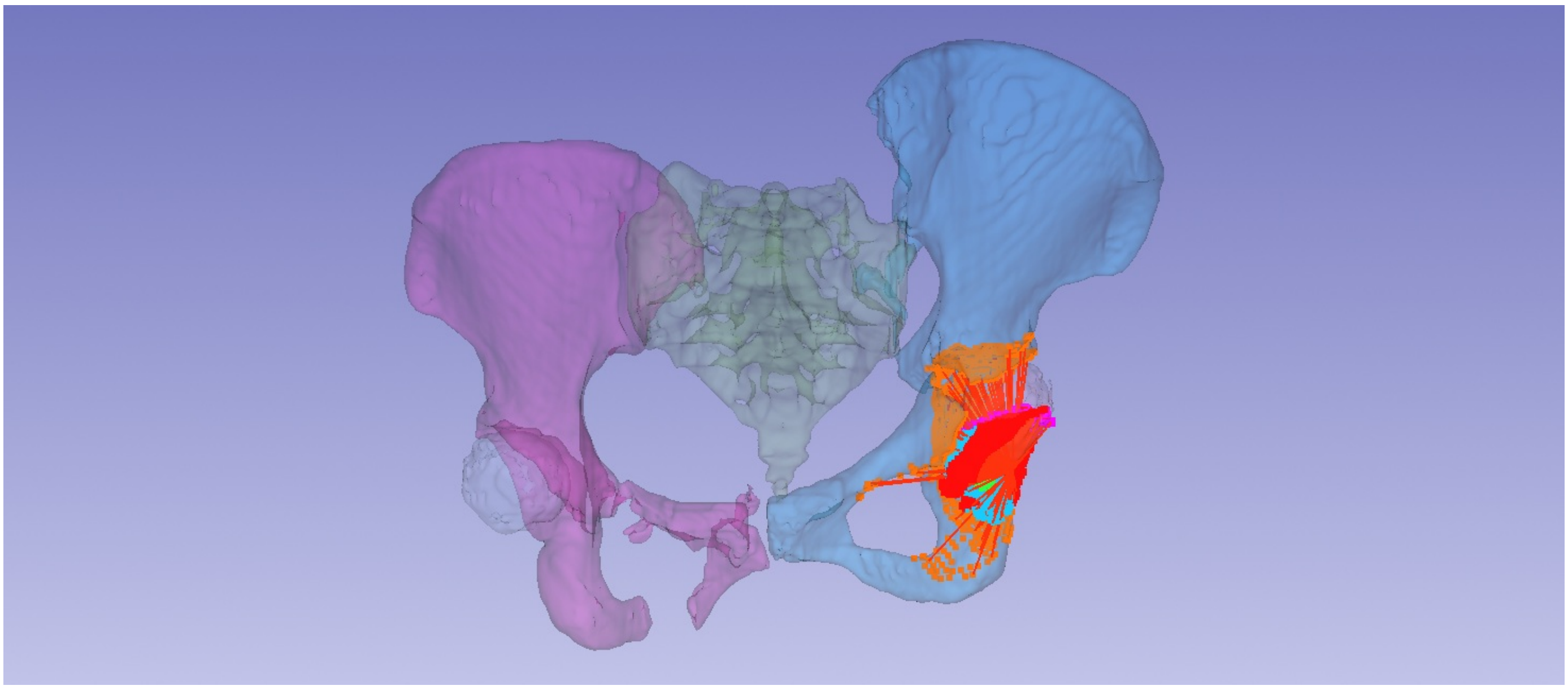

MEASUREMENT	RESULT	REFERENCE / COMPARATOR	INTERPRETATION
Defect class / severity	**SPLIT FRACTURE / LARGE**	Letournel/Judet provides the anatomical fracture framework, not percentage cutoffs [13]	SUPERIOR–POSTERIOR
Defected support	**92.0%**	No validated normal population range for this registered-template metric.	Study-internal technical rule.
Central / medial status	**MEDIAL-WALL DEFECT**	central support/displacement cutoffs are internal trial thresholds.	Study-internal technical rule.

Expected, present, displaced, and missing acetabular support are reported by anatomical direction. Judet-Letournel is used only as fracture-anatomy background [13].

Pelvic CT Segmentation and Quantitative Anatomical Analysis

8. Original-CT Support Confirmation

ORIGINAL-CT CONFIRMATION: STATUS CONFIRMED; ACETABULAR DEFECT CONFIRMED; HEAD POSITION CONFIRMED.

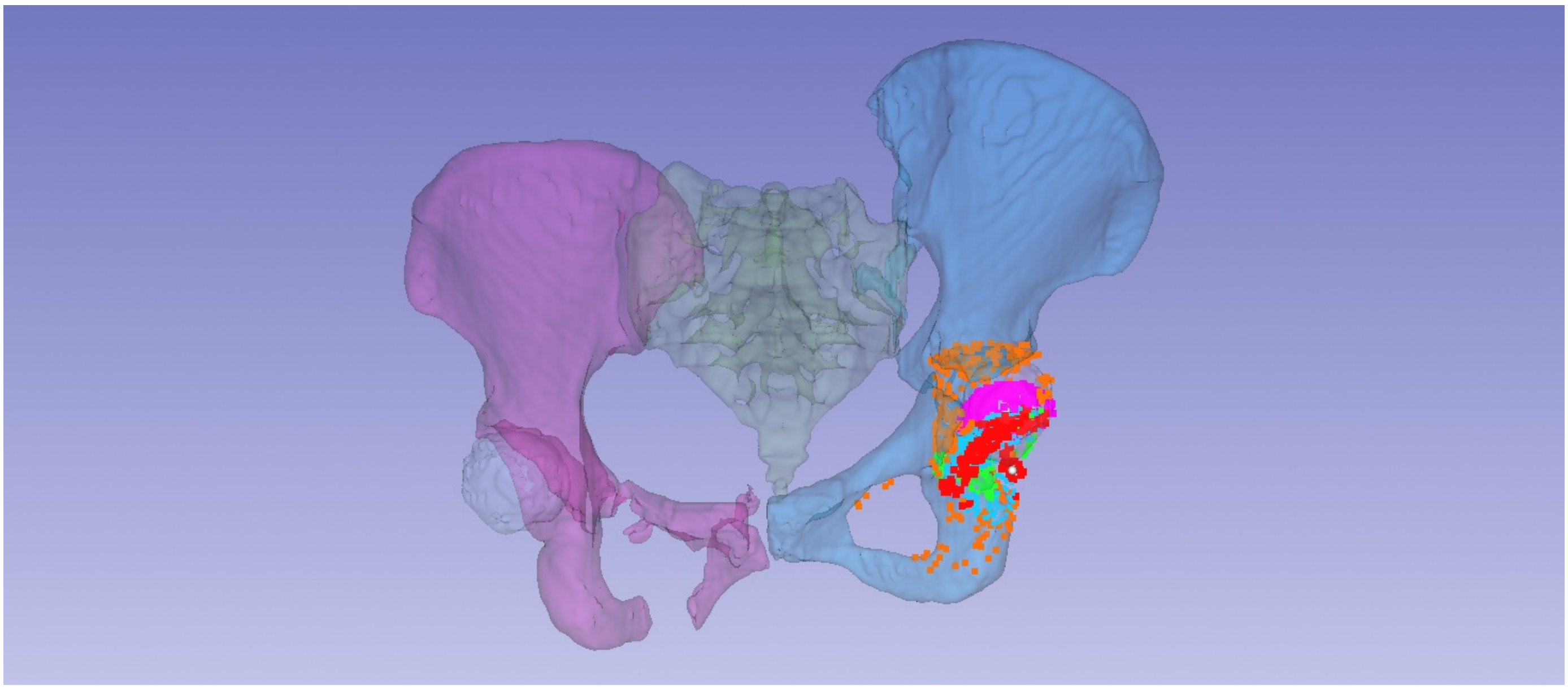

MEASUREMENT	RESULT	REFERENCE / COMPARATOR	INTERPRETATION
CT defect confirmation	**CONFIRMED**	Cortical >300 HU and cancellous 150-300 HU provide tissue context, but ray gates are algorithm-specific [14]	CONFIRMED
CT defected support	**81.1%**	No validated normal range for this CT-support fraction.	Study-internal technical rule.
Median support offset	**9.8 mm**	No validated clinical cutoff for the registered-template offset metric.	Study-internal technical rule.

Original scalar CT is sampled along the registered expected socket to assess actual bony support. HU and percentage gates are technical analysis parameters; published HU ranges are tissue context only [14].

Pelvic CT Segmentation and Quantitative Anatomical Analysis

9. Registered Expected-Socket Original-CT Ray Confirmation

FLAGGED: Left 3 accepted acetabular discontinuity cluster(s); maximum 3-D span 9.2 mm; 5 mm or larger.

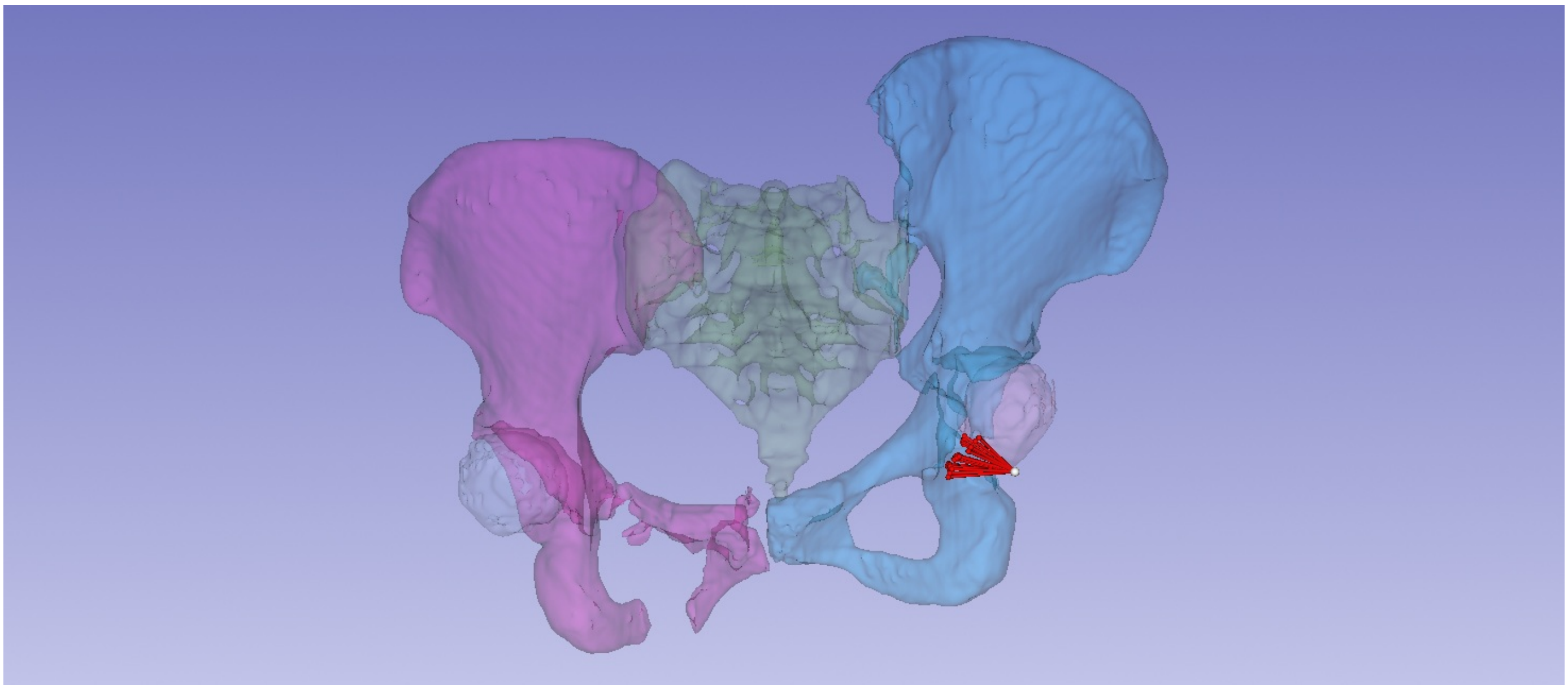

MEASUREMENT	RESULT	REFERENCE / COMPARATOR	INTERPRETATION
Abnormal side	**LEFT**	Registered expected native socket geometry	Original CT remains the bone-evidence source
Accepted CT discontinuity clusters	**3**	Expected/template geometry is never counted as bone	CT-CONFIRMED INTERNAL ACETABULAR DISCONTINUITY: ANTERIOR-SUPERIOR + CENTRAL
Cluster 1 radial opening	**NA**	Measured against the registered expected native socket using original CT	ANTERIOR-SUPERIOR; GAP / NO CT-SUPPORTED BONE
Cluster 2 radial opening	**NA**	Measured against the registered expected native socket using original CT	CENTRAL; GAP / NO CT-SUPPORTED BONE
Cluster 3 radial opening	**4.80 mm**	Measured against the registered expected native socket using original CT	ANTERIOR-SUPERIOR; MILD WITH PARTIAL NO-HIT GAP

For the registered abnormal reconstruction route, the contralateral socket is only an initial geometric prior and is rigidly registered to stable ipsilateral extra-acetabular anatomy. The registered expected socket defines where bone is expected; only original CT support inside the actual abnormal-side innominate can satisfy a ray. Template geometry is never counted as bone.

Pelvic CT Segmentation and Quantitative Anatomical Analysis

10. CT-Supported Socket Reconstruction

CT-supported socket reconstruction completed on LEFT; 1 CT-matched displaced fragment candidate(s) recovered.

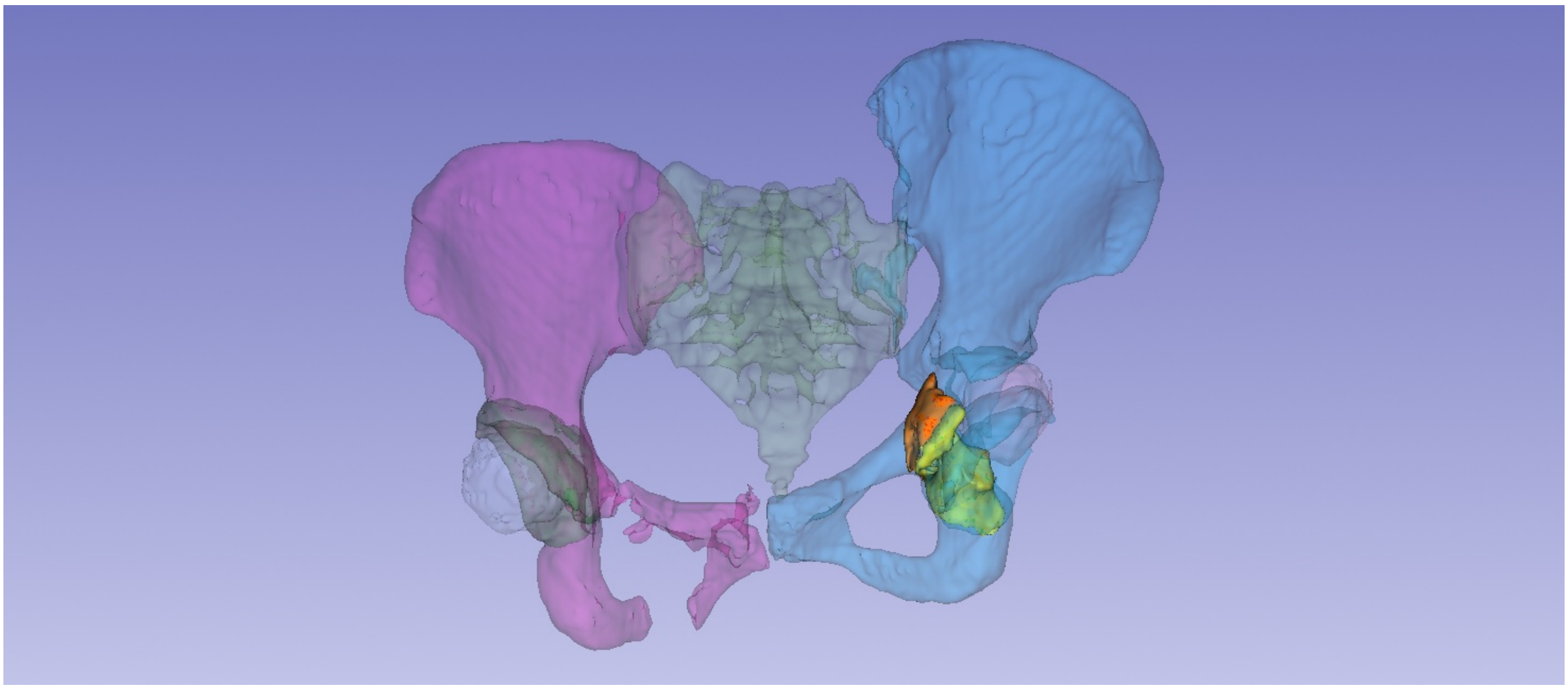

MEASUREMENT	RESULT	REFERENCE / COMPARATOR	INTERPRETATION
Adaptive status	**CREATED**	No population normal range; reconstruction step only.	Technical reconstruction
Recovered fragments	**1**	No validated clinical cutoff.	CT-matched candidate fragments

CT-supported displaced acetabular material is evaluated within the abnormal reconstruction route. This page reports reconstruction output rather than a population diagnostic cutoff.

Pelvic CT Segmentation and Quantitative Anatomical Analysis

11. Final Acetabular Socket

Final acetabular socket reconstruction completed on LEFT; original-CT acetabular defect confirmation: CONFIRMED; MEDIAL-WALL DEFECT.

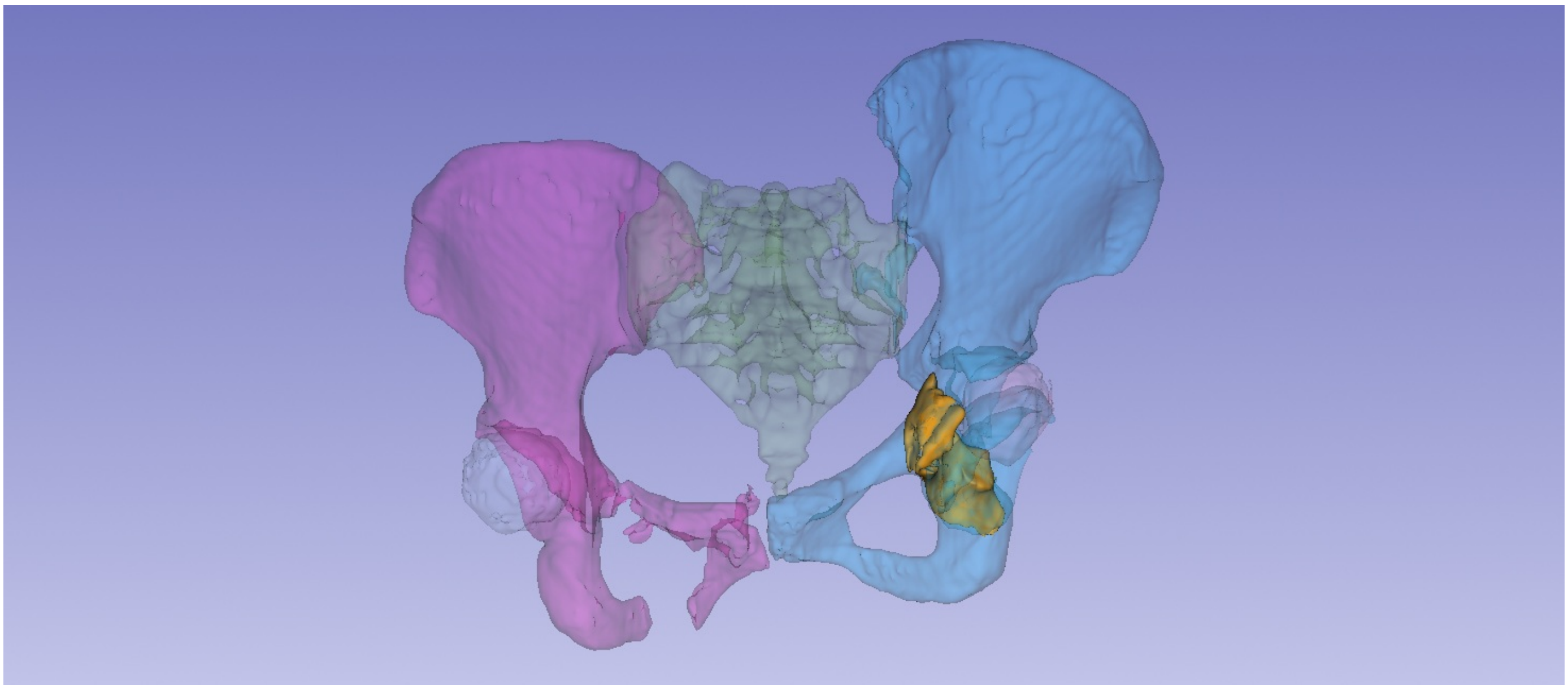

MEASUREMENT	RESULT	REFERENCE / COMPARATOR	INTERPRETATION
Reconstruction status	**COMPLETED**	Final patient-specific socket reconstruction stage	LEFT acetabulum
Original-CT defect confirmation	**CONFIRMED**	Original CT support confirmation from the completed reconstruction chain	MEDIAL-WALL DEFECT
CT-matched displaced fragments	**1**	Candidate fragments recovered by the CT-supported reconstruction step	Patient-specific reconstruction output

Final patient-specific socket support is summarized against the registered expected anatomy. Contralateral anatomy is used as a patient-specific geometric prior, not perfect ground truth [15].

Pelvic CT Segmentation and Quantitative Anatomical Analysis

Pubic Symphysis

PUBIC SYMPHYSIS: SUSPECTED TRAUMATIC SYMPHYSEAL DIASTASIS; GAP 21.3 mm, AP/SI SHEAR 0.1 mm/0.7 mm.

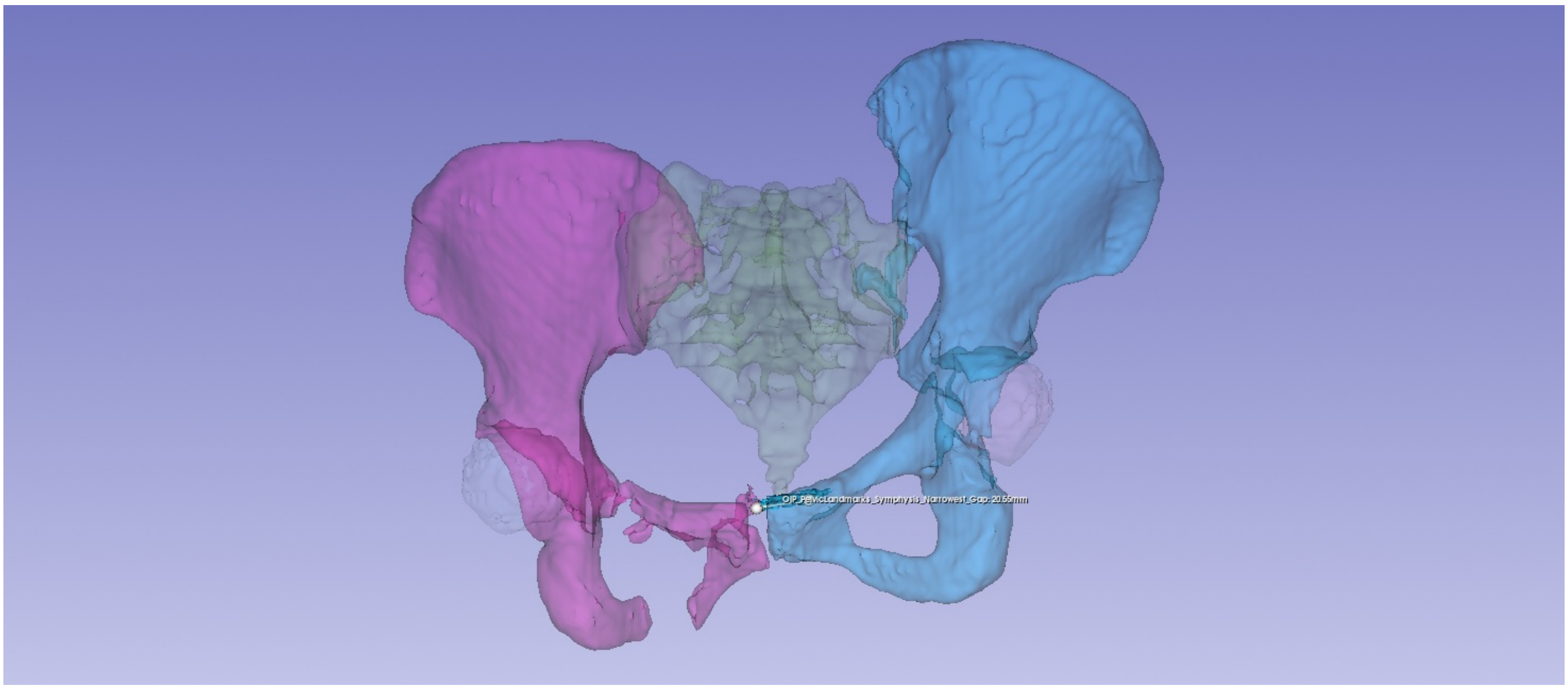

MEASUREMENT	RESULT	REFERENCE / COMPARATOR	INTERPRETATION
Narrowest symphyseal gap	**21.3 mm**	Manual axial-CT mean 3.1 +/- 1.5 mm; calculated mean +/-2SD approximately 0.1-6.1 mm [1]	SUSPECTED TRAUMATIC SYMPHYSEAL DIASTASIS
AP / SI shear	**0.1 mm / 0.7 mm**	No validated static-CT cutoff for this automated measurement; the published 5-mm value refers to dynamic flamingo radiographs [4]	Descriptive only

Narrowest stable left-right symphyseal gap. Hwang et al. used a manual axial-CT measurement; the calculated 6.1-mm mean+2SD value is descriptive rather than diagnostic [1].

Pelvic CT Segmentation and Quantitative Anatomical Analysis

Obturator Ring

OBTURATOR RING: FLAGGED: Right 2 rim candidate(s), maximum bridge chord 56.3 mm; 5 mm or larger; Left no reportable rim discontinuity.

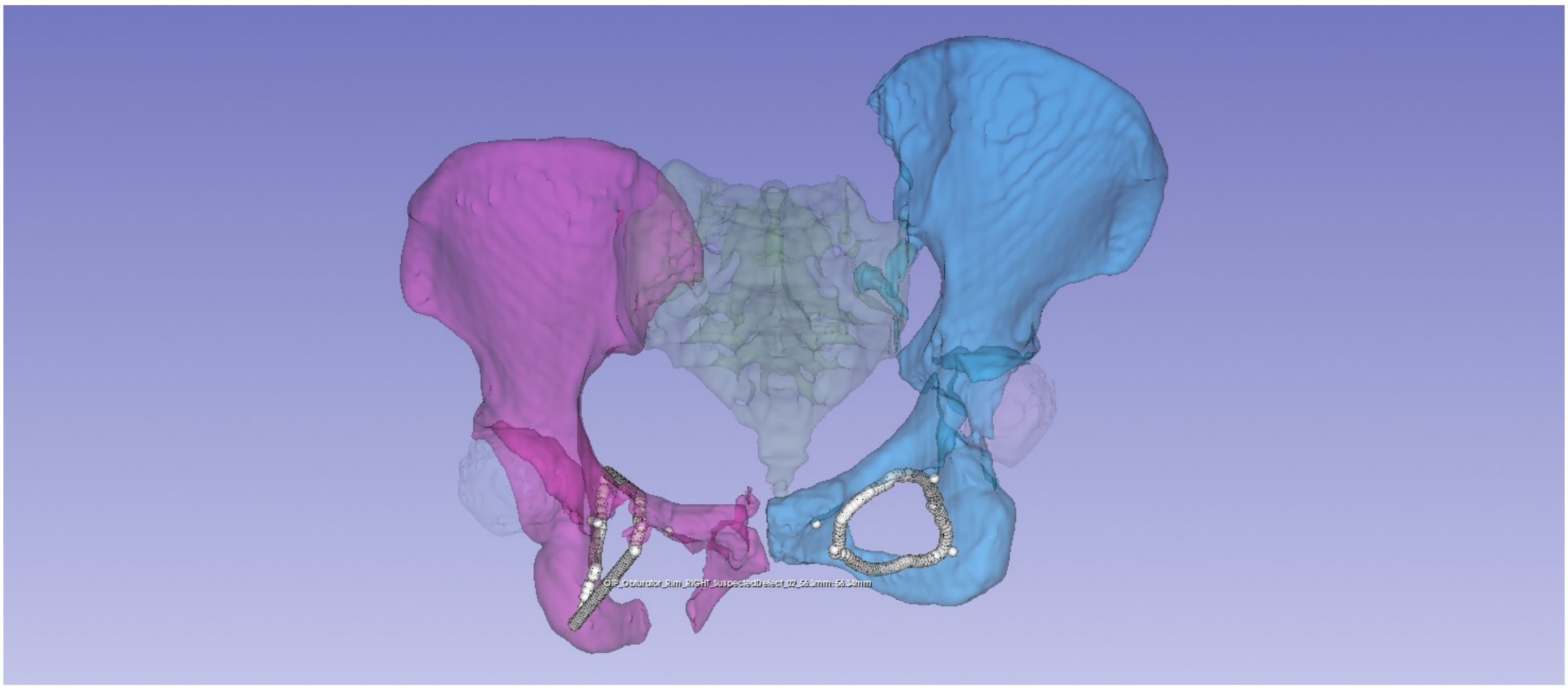

MEASUREMENT	RESULT	REFERENCE / COMPARATOR	INTERPRETATION
Right AP / SI extent	**63.8 mm / 58.3 mm**	Dry-bone mean AP 52.4 +/- 8.0 mm; SI 46.9 +/- 18.3 mm [7]	Descriptive dry-bone comparator only
Right rim discontinuity	**2**	4-mm registered pelvic asymmetry level is an alert comparator, not a fracture cutoff [6]	Visual confirmation required
Left AP / SI extent	**40.9 mm / 36.7 mm**	Dry-bone mean AP 51.7 +/- 8.7 mm; SI 46.6 +/- 16.9 mm [7]	Descriptive dry-bone comparator only
Left rim discontinuity	**0**	4-mm registered pelvic asymmetry level is an alert comparator, not a fracture cutoff [6]	Visual confirmation required

The inner obturator rim is tracked through 360 degrees. The 4-mm literature value is a registered pelvic-asymmetry comparator, not a fracture cutoff; dry-bone AP/SI dimensions are descriptive only [6,7].

Pelvic CT Segmentation and Quantitative Anatomical Analysis

Sacroiliac Joints

SACROILIAC JOINTS ASSESSED: SUSPECTED SIJ DIASTASIS ABSOLUTE AND ASYMMETRIC; P95 side difference axial 7.2 mm / coronal 4.4 mm; width wider than age reference/narrower than age reference.

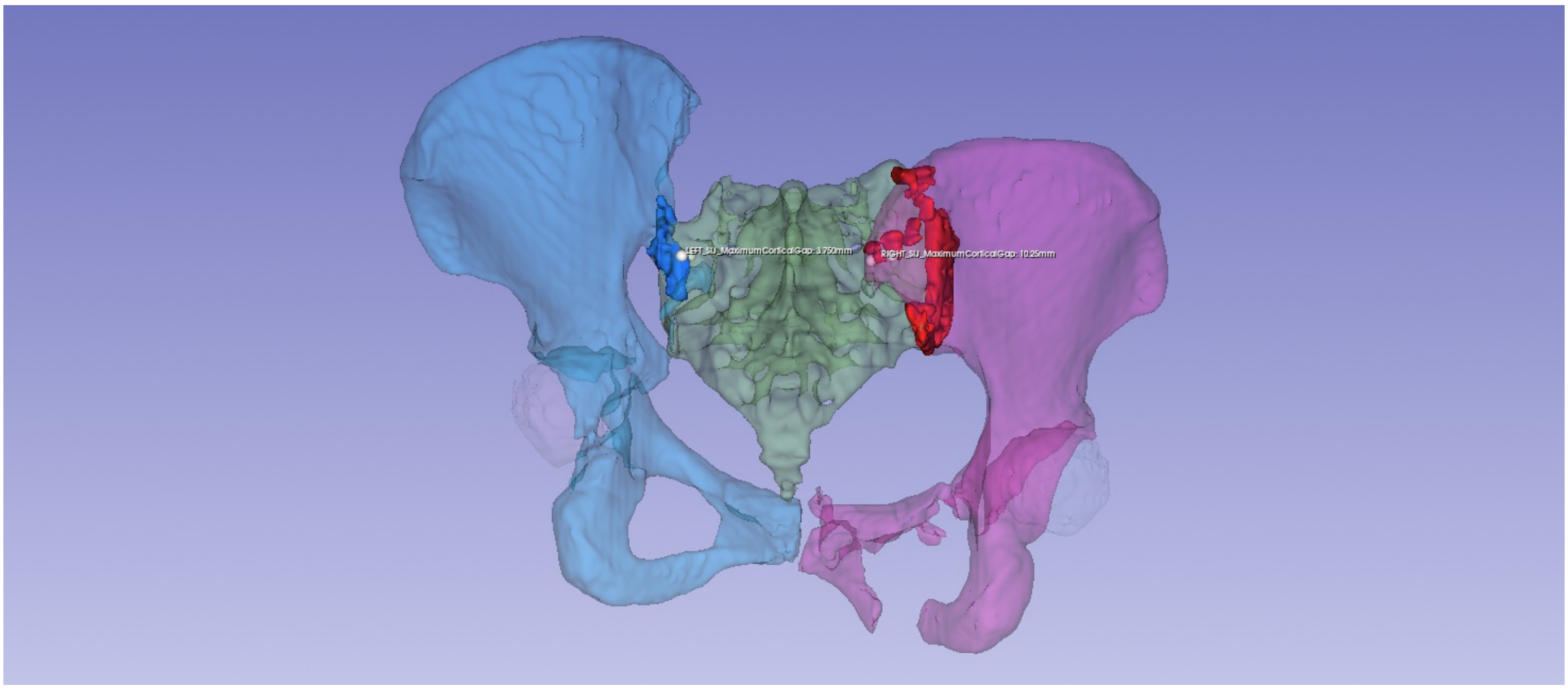

MEASUREMENT	RESULT	REFERENCE / COMPARATOR	INTERPRETATION
Right median cortical gap	**3.2 mm**	Published CT reference: 2.3 +/- 0.4 mm for patients <40 y and 1.9 +/- 0.2 mm for patients >40 y; patient age not supplied [8]	WIDER THAN AGE REFERENCE
Left median cortical gap	**0.2 mm**	Published CT reference: 2.3 +/- 0.4 mm for patients <40 y and 1.9 +/- 0.2 mm for patients >40 y; patient age not supplied [8]	NARROWER THAN AGE REFERENCE
Axial / coronal side difference	**7.2 mm / 4.4 mm**	Study-specific open-reduction context: 3.39 / 3.71 mm; absolute 6.52 / 7.26 mm [9]	SUSPECTED SIJ DIASTASIS ABSOLUTE AND ASYMMETRIC

Opposing sacral and iliac cortices localize the SI joint, and original CT is sampled along local normals. Published normal widths and trauma cut-points are comparators because the automated 3-D construction differs from manual MPR measurements [8,9].

Pelvic CT Segmentation and Quantitative Anatomical Analysis

Final Innominate Partition

FINAL INNOMINATE PARTITION: EXACT BILATERAL RECONSTRUCTION. Current method: ROOT10 single-plane pubis/ischium split.

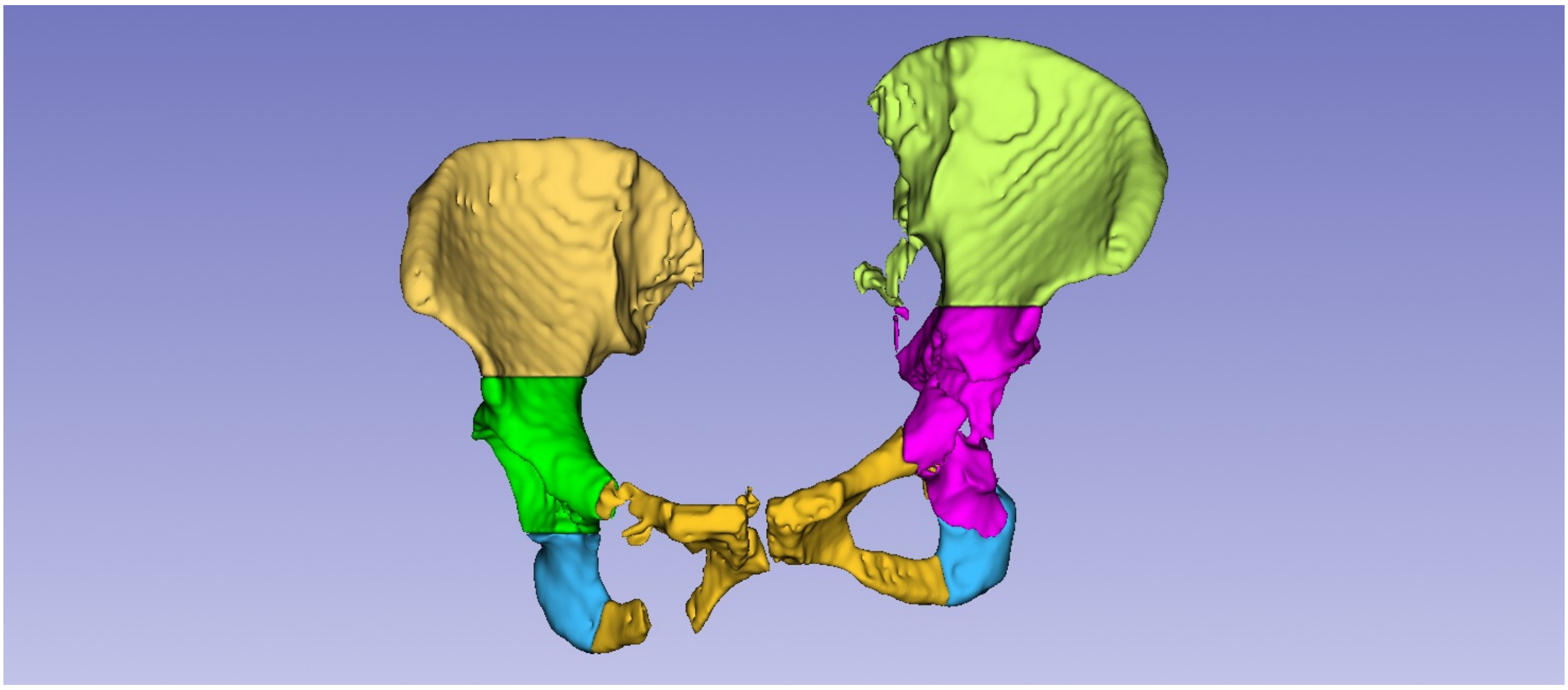

MEASUREMENT	RESULT	REFERENCE / COMPARATOR	INTERPRETATION
Right reconstruction	**PASS**	Technical requirement: zero overlap/unassigned/outside voxels.	ROOT10 single-plane pubis/ischium split
Left reconstruction	**PASS**	Technical requirement: zero overlap/unassigned/outside voxels.	ROOT10 single-plane pubis/ischium split

The innominate is reconstructed as mutually exclusive acetabulum, ilium, pubis, and ischium. Zero overlap, unassigned, and outside voxels are technical consistency requirements.

Pelvic CT Segmentation and Quantitative Anatomical Analysis

Technical Measurements - Page 1

**ROUTE / QUALITY CONTROL**	
Report status	VALID
Initial pathway	ABNORMAL
Final state	ABNORMAL
Midsagittal-plane QC	PASS — BOTH CENTRAL BONES ARE NEAR-EQUAL
**1. FEMORAL-HEAD MORPHOLOGY AND CENTRE - TECHNICAL MEASUREMENTS**	
**RIGHT**	
Centre confidence	MODERATE
Fitted radius	20.31 mm
Sphere RMS	1.07 mm
Normalized RMS	5.29%
P95 residual	2.06 mm
Meaningful components	1
**LEFT**	
Centre confidence	MODERATE
Fitted radius	19.98 mm
Sphere RMS	1.28 mm
Normalized RMS	6.42%
P95 residual	2.59 mm
Meaningful components	1
**BILATERAL QC**	
Radius difference	0.33 mm
Radius difference	1.66%
Bilateral radius QC	PASS
**2. NATIVE HEAD-SOCKET SHELL AND CONGRUITY - TECHNICAL MEASUREMENTS**	
**RIGHT**	
Margin status	CONTINUOUS
Structure class	INTACT CONTACT MARGIN
Local congruity	CONGRUENT
Full-head coverage	39.73%
Superior-head coverage	56.17%
Shell area	3205.38 mm²
Meaningful components	1
Median head-to-shell gap	2.72 mm
P95 head-to-shell gap	5.99 mm
**LEFT**	
Margin status	SPLIT
Structure class	SPLIT FRACTURE
Local congruity	INCONGRUENT
Full-head coverage	18.29%
Superior-head coverage	28.42%
Shell area	2233.16 mm²

Pelvic CT Segmentation and Quantitative Anatomical Analysis

Technical Measurements - Page 2

Meaningful components	2
Median head-to-shell gap	5.47 mm
P95 head-to-shell gap	6.84 mm
**3. MIDSAGITTAL PLANE AND CENTRAL-BONE QC - TECHNICAL MEASUREMENTS**	
Overall plane QC	PASS — BOTH CENTRAL BONES ARE NEAR-EQUAL
**L5**	
RIGHT volume	24269.13 mm3
LEFT volume	25665.05 mm3
Bilateral difference	5.59%
Smaller/larger ratio	0.9456
Symmetry QC	NEAR-EQUAL
**Sacrum**	
RIGHT volume	90937.76 mm3
LEFT volume	87709.80 mm3
Bilateral difference	3.61%
Smaller/larger ratio	0.9645
Symmetry QC	NEAR-EQUAL
**6. REGISTERED EXPECTED NATIVE SOCKET AND HEAD DISPLACEMENT - TECHNICAL MEASUREMENTS**	
Abnormal side	LEFT
Total displacement	24.28 mm
Anterior component	-15.86 mm
Superior component	18.16 mm
Medial component	2.85 mm
Canonical direction	SUPERIOR + POSTERIOR
Position class	DISPLACED RELATIVE TO REGISTERED EXPECTED SOCKET
Local congruity	INCONGRUENT
Original-CT position confirmation	CONFIRMED
Direction report QC	SOURCE_REPORTED
**7. REGISTERED TEMPLATE DEFECT GEOMETRY - TECHNICAL MEASUREMENTS**	
Margin status	SPLIT
Defect class	SPLIT FRACTURE
Defect severity	LARGE
Defect direction	SUPERIOR–POSTERIOR
Geometric defect percentage	92.00%
Estimated defect area	2948.95 mm²
Central status	MEDIAL-WALL DEFECT
**8. ORIGINAL-CT SUPPORT CONFIRMATION - TECHNICAL MEASUREMENTS**	
CT status	CONFIRMED
Acetabular defect confirmation	CONFIRMED
Head-position confirmation	CONFIRMED
CT defect direction	SUPERIOR–POSTERIOR
CT central status	MEDIAL-WALL DEFECT

Pelvic CT Segmentation and Quantitative Anatomical Analysis

Technical Measurements - Page 3

Normal-template confirmation	100.00%
Total CT-defected support	81.08%
Median support offset	9.75 mm
P95 support offset	29.42 mm
Head outside expected socket	34.62%
P95 outside depth	20.58 mm
**9. REGISTERED EXPECTED-SOCKET ORIGINAL-CT RAY CONFIRMATION - TECHNICAL MEASUREMENTS**	
Abnormal side	LEFT
Internal rays tested	1027
Internal CT-supported rays	236
Accepted defect rays	23
Accepted defect clusters	3
Final conclusion	CT-CONFIRMED INTERNAL ACETABULAR DISCONTINUITY: ANTERIOR-SUPERIOR + CENTRAL
**10. CT-SUPPORTED SOCKET RECONSTRUCTION - TECHNICAL MEASUREMENTS**	
Adaptive status	CREATED
Pathway	REGISTERED CONTRALATERAL FULL-VOLUME TEMPLATE + MISSING-SECTOR DISPLACEMENT SWEEP + ACTUAL-HEAD SHELL RECOVERY
Head displacement	24.28 mm
Displacement direction	SUPERIOR + POSTERIOR
Position class	DISPLACED RELATIVE TO REGISTERED EXPECTED SOCKET
Local congruity	INCONGRUENT
CT status	CONFIRMED
Accepted candidate displaced fragments	1
**11. FINAL ACETABULAR SOCKET - TECHNICAL MEASUREMENTS**	
Abnormal side	LEFT
Medial-wall involvement	NO
Original-CT defect confirmation	CONFIRMED
CT central status	MEDIAL-WALL DEFECT
Adaptive pathway	REGISTERED CONTRALATERAL FULL-VOLUME TEMPLATE + MISSING-SECTOR DISPLACEMENT SWEEP + ACTUAL-HEAD SHELL RECOVERY
CT-matched candidate displaced fragments	1
**PUBIC SYMPHYSIS - TECHNICAL MEASUREMENTS**	
Clinical status	ABNORMAL
Screening classification	SUSPECTED TRAUMATIC SYMPHYSEAL DIASTASIS
Stable narrowest gap	21.26 mm
Selected pair gap	20.54 mm
Three-dimensional point distance	20.55 mm
AP shear	0.13 mm
SI shear	0.68 mm
Supporting local cells	5
Local gap IQR	1.35 mm

Pelvic CT Segmentation and Quantitative Anatomical Analysis

Technical Measurements - Page 4

**OBTURATOR RING - TECHNICAL MEASUREMENTS**	
**RIGHT**	
AP extent	63.78 mm
SI extent	58.32 mm
Area	1927.70 mm²
Perimeter	212.61 mm
Tracked rim	43.61%
Tracked rim outside 4-mm band	73.25%
Suspected discontinuities	2
Defect 1 bridge chord	51.31 mm
Defect 1 estimated missing rim arc	46.80 mm
Defect 2 bridge chord	56.34 mm
Defect 2 estimated missing rim arc	45.93 mm
**LEFT**	
AP extent	40.86 mm
SI extent	36.69 mm
Area	1658.20 mm²
Perimeter	170.19 mm
Tracked rim	100.00%
Tracked rim outside 4-mm band	69.44%
Suspected discontinuities	0
**SACROILIAC JOINTS - TECHNICAL MEASUREMENTS**	
Width classification	WIDER THAN AGE REFERENCE
Median cortical width	3.25 mm
P95 cortical width	7.75 mm
Maximum cortical width	10.25 mm
P95 axial projection	7.49 mm
P95 coronal projection	4.69 mm
CT-validated rays	675
Validation fraction	0.987
Bilateral trauma screen	SUSPECTED SIJ DIASTASIS ABSOLUTE AND ASYMMETRIC
Width classification	NARROWER THAN AGE REFERENCE
Median cortical width	0.25 mm
P95 cortical width	0.25 mm
Maximum cortical width	3.75 mm
P95 axial projection	0.25 mm
P95 coronal projection	0.25 mm
CT-validated rays	210
Validation fraction	1.000
Bilateral trauma screen	SUSPECTED SIJ DIASTASIS ABSOLUTE AND ASYMMETRIC
**FINAL INNOMINATE PARTITION - TECHNICAL MEASUREMENTS**	
**RIGHT**	

Pelvic CT Segmentation and Quantitative Anatomical Analysis

Technical Measurements - Page 5

Exact reconstruction	True
Unassigned voxels	0
Outside voxels	0
Overlap voxels	0
Acetabulum voxels	102189
Ilium voxels	181820
Pubis voxels	35148
Ischium voxels	35630
**LEFT**	
Exact reconstruction	True
Unassigned voxels	0
Outside voxels	0
Overlap voxels	0
Acetabulum voxels	95934
Ilium voxels	206129
Pubis voxels	36878
Ischium voxels	36106
**CURRENT PARTITION METHOD**	
Method	ROOT10 single-plane pubis/ischium split
Split mode	BOX ANCHOR FINITE20 ROOT CORRIDOR ILIUM FLOOR
Obturator used for split	False
Mirroring used for split	False
8-mm reclamation	False

Pelvic CT Segmentation and Quantitative Anatomical Analysis

References

Published comparators are interpreted according to their original measurement definitions; where the automated measurement differs, comparison is descriptive rather than a diagnostic cutoff.

[1] Hwang K, Wu X, Park CY. Width of pubic symphysis relating to age and sex in Koreans. J Orthop Surg Res. 2021;16(1):430. doi:10.1186/s13018-021-02561-9. PMID:34217332.

[2] Doro CJ, Forward DP, Kim H, Nascone JW, Sciadini MF, Hsieh AH, et al. Does 2.5 cm of symphyseal widening differentiate anteroposterior compression I from anteroposterior compression II pelvic ring injuries? J Orthop Trauma. 2010;24(10):610-615. doi:10.1097/BOT.0b013e3181cff42c. PMID:20871248.

[3] Gansslen A, Lindahl J, Krappinger D, Lindtner RA, Staresinic M. The myth of 2.5 cm symphyseal diastasis. Arch Orthop Trauma Surg. 2025;145(1):306. doi:10.1007/s00402-025-05904-x. PMID:40397156.

[4] Garras DN, Carothers JT, Olson SA. Single-leg-stance (flamingo) radiographs to assess pelvic instability: how much motion is normal? J Bone Joint Surg Am. 2008;90(10):2114-2118. doi:10.2106/JBJS.G.00277. PMID:18829908.

[5] Larson CM, Moreau-Gaudry A, Kelly BT, Byrd JWT, Tonetti J, Lavallee S, et al. Are normal hips being labeled as pathologic? A CT-based method for defining normal acetabular coverage. Clin Orthop Relat Res. 2015;473(4):1247-1254. doi:10.1007/s11999-014-4055-2. PMID:25407391.

[6] Zhang F, Zhang D, Huang Z, Wang Z, Cai X. Morphological asymmetry of pelvic rings: a study based on three-dimensional deviation analysis. Orthop Surg. 2022;14(5):967-976. doi:10.1111/os.13246. PMID:35377554.

[7] Duygu OB, Govsa F. Anatomical structures of the obturator foramen and its importance for pelvic interventions. Surg Radiol Anat. 2025;47(1):163. doi:10.1007/s00276-025-03661-5. PMID:40544414.

[8] Faflia CP, Prassopoulos PK, Daskalogiannaki ME, Gourtsoyiannis NC. Variation in the appearance of the normal sacroiliac joint on pelvic CT. Clin Radiol. 1998;53(10):742-746. doi:10.1016/S0009-9260(98)80316-4. PMID:9817091.

[9] Chien RS, Chen IJ, Lai CY, Chen JP, Yu YH. Critical distance of the sacroiliac joint for open reduction using screw fixation for traumatic sacroiliac joint diastasis: a retrospective study. J Orthop Surg Res. 2024;19(1):268. doi:10.1186/s13018-024-04759-z. PMID:38678298.

[10] Jamali AA, Mak W, Wang P, Tai L, Meehan JP, Lamba R. What is normal femoral head/neck anatomy? An analysis of radial CT reconstructions in adolescents. Clin Orthop Relat Res. 2013;471(11):3581-3587. doi:10.1007/s11999-013-3166-5. PMID:23922189.

[11] Hassan MM, Feroe AG, Douglass BW, Jimenez AE, Kuhns B, Mitchell CF, et al. Three-dimensional analysis of age and sex differences in femoral head asphericity in asymptomatic hips in the United States. World J Orthop. 2024;15(8):754-763. doi:10.5312/wjo.v15.i8.754. PMID:39165879.

[12] Rivas DJL, Gassmann JM, Goetz JE, Aitken HD, Davison JC, Miller A, et al. Weight-bearing CT as an approach to assess femoral-acetabular displacement during external rotation stress in the hip. J Hip Preserv Surg. 2025;12(2):93-104. doi:10.1093/jhps/hnaf001. PMID:40761572.

[13] Judet R, Judet J, Letournel E. Fractures of the acetabulum: classification and surgical approaches for open reduction. Preliminary report. J Bone Joint Surg Am. 1964;46:1615-1646. doi:10.2106/00004623-196446080-00001. PMID:14239854.

[14] Imani M, Buratto J, Dao T, Meijering E, Vogrin S, Kwok TCY, et al. Deep learning technique for automatic segmentation of proximal hip musculoskeletal tissues from CT scan images: a MrOS study. J Cachexia Sarcopenia Muscle. 2025;16(2):e13728. doi:10.1002/jcsm.13728. PMID:40022453.

[15] Ead MS, Duke KK, Jaremko JL, Westover L. Investigation of pelvic symmetry using CAD software. Med Biol Eng Comput. 2020;58(1):75-82. doi:10.1007/s11517-019-02068-w. PMID:31745838.

[16] Amar E, Druckmann I, Flusser G, Safran MR, Salai M, Rath E. The anterior inferior iliac spine: size, position, and location. An anthropometric and sex survey. Arthroscopy. 2013;29(5):874-881. doi:10.1016/j.arthro.2013.01.023. PMID:23523127.

[17] Rana SH, Farjoodi P, Haloman S, Dutton P, Hariri A, Ward SR, et al. Anatomic evaluation of the sacroiliac joint: a radiographic study with implications for procedures. Pain Physician. 2015;18(6):583-592. PMID:26606010.

[18] Hartel MJ, Petersik A, Schmidt A, Kendoff D, Nuchtern J, Rueger JM, et al. Determination of femoral neck angle and torsion angle utilizing a novel three-dimensional modeling and analytical technology based on CT datasets. PLoS One. 2016;11(3):e0149480. doi:10.1371/journal.pone.0149480.

[19] Mahmood SS, Mukka SS, Crnalic S, Wretenberg P, Sayed-Noor AS. Association between changes in global femoral offset after total hip arthroplasty and function, quality of life, and abductor muscle strength. Acta Orthop. 2016;87(1):36-41. doi:10.3109/17453674.2015.1091955.

[20] O'Brien S, Kernohan G. Perception of imposed leg length inequality in normal subjects. Hip Int. 2010;20(4):505-511. doi:10.1177/112070001002000414.

[21] Wang G, et al. Measurement of relative position of femoral head center, greater trochanter, and lesser trochanter. Ann Palliat Med. 2021;10(11):11524-11528. doi:10.21037/apm-21-2538.

Pelvic CT Segmentation and Quantitative Anatomical Analysis

Clinical Findings Summary

Case ID: ACETFX_0013_0000 | Age: Not supplied | Status: VALID

CONCLUSIVE FINDING

Automated CT analysis flags Left head-socket suspected dislocation; acetabular discontinuity of at least 5 mm; Left femoral-head displacement posterior + inferior; and 2 additional flagged finding(s). Femoral parameter assessment is flagged; see the dedicated evidence page.

OVERALL STATION ASSESSMENT

Flagged finding of at least 5 mm in the hip/acetabular station group(s).

Clinical station checklist

HIP JOINT AND ACETABULUM

Femoral-head morphology — NO FLAG

R VALID HEAD CENTRE — NORMAL-LIKE MORPHOLOGY / L VALID HEAD CENTRE — MORPHOLOGY CAUTION

Head-socket congruity — FLAGGED

R CONGRUENT / L SUSPECTED DISLOCATION (POSTERIOR + INFERIOR), 36.8 mm

Acetabular coverage — NO FLAG

R 45.2% / L 40.4%; no low-coverage flag

Acetabular continuity — FLAGGED

4 accepted cluster(s); maximum 3-D span 23.9 mm; 5 mm or larger discontinuity finding

Head position / dislocation — FLAGGED

LEFT 36.8 mm; POSTERIOR + INFERIOR

Joint-space / arthrosis screen — FLAGGED

R NARROWED — OSTEOARTHRITIS CANDIDATE (3.00 / 0.00 / 8.00 mm) / L NARROWED — OSTEOARTHRITIS CANDIDATE (1.00 / -2.00 / 6.50 mm)

Femur parameters — FLAGGED

Automated femoral analysis flags left 3D NSA above Hartel true-3D CT IQR; bilateral femoral-offset asymmetry ≥5 mm; limb-length discrepancy 27.2 mm (left shorter); left head-centre-to-lesser I/D

ANTERIOR PELVIC RING

Overall anterior ring screen — NO FLAG

No abnormality at all assessed anterior stations

Pubic symphysis — NO FLAG

Gap 1.4 mm; NO CT EVIDENCE OF SYMPHYSEAL DIASTASIS

Right obturator ring — NO FLAG

0 reportable rim discontinuity candidates

Left obturator ring — NO FLAG

0 reportable rim discontinuity candidates

POSTERIOR PELVIC RING

Right SIJ integrity — NO FLAG

median 2.2 mm; P95 4.5 mm; NORMAL WIDTH COMPATIBLE

Left SIJ integrity — NO FLAG

median 2.0 mm; P95 4.5 mm; NORMAL WIDTH COMPATIBLE

SCOPE: Joint-space narrowing is an arthrosis/coxarthrosis screen, not a stand-alone diagnosis. These station tests do not exclude isolated or minimally displaced iliac or sacral fractures; review the source CT.

SIJ references: normal CT mean 2.3 +/- 0.4 mm (<40 y) and 1.9 +/- 0.2 mm (>40 y) [8]; traumatic axial/coronal distance context [9]; anatomical extent/search-corridor context [17].

Detailed anatomical evidence and quantitative measurements follow on the next pages.

Pelvic CT Segmentation and Quantitative Anatomical Analysis

1. Femoral-Head Morphology and Centre

RIGHT FEMORAL HEAD: VALID HEAD CENTRE — NORMAL-LIKE MORPHOLOGY; CENTRE HIGH CONFIDENCE. LEFT FEMORAL HEAD: VALID HEAD CENTRE — MORPHOLOGY CAUTION; CENTRE MODERATE CONFIDENCE.

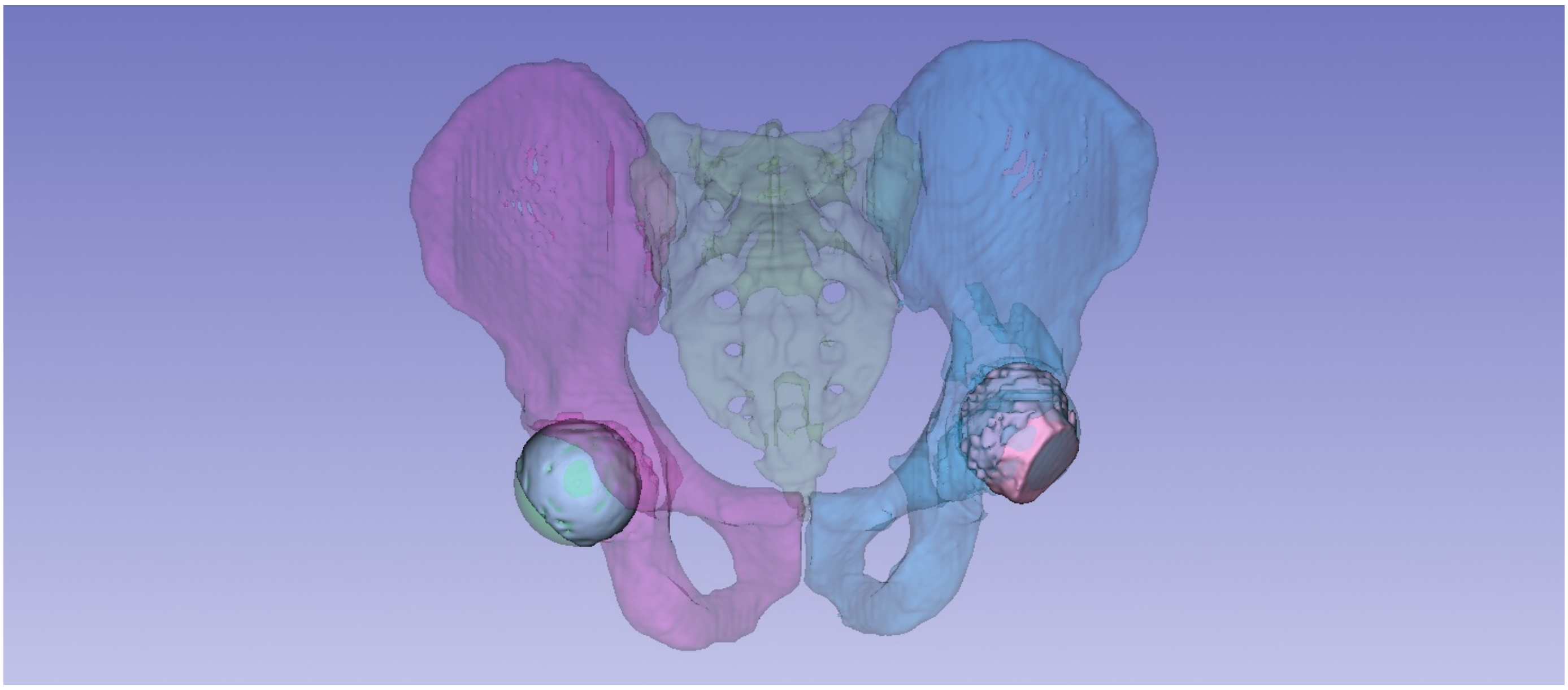

MEASUREMENT	RESULT	REFERENCE / COMPARATOR	INTERPRETATION
Right fitted radius	**24.5 mm**	Adolescent radial-CT comparator: male 24.53 +/- 1.74 mm; female 21.94 +/- 1.13 mm [10]	VALID HEAD CENTRE — NORMAL-LIKE MORPHOLOGY
Left fitted radius	**23.8 mm**	Adolescent radial-CT comparator: male 24.53 +/- 1.74 mm; female 21.94 +/- 1.13 mm [10]	VALID HEAD CENTRE — MORPHOLOGY CAUTION
Sphere-fit residual	**R/L P95 1.1 mm/4.0 mm**	Published 3-D sphere/asphericity analysis supports the measurement family; these residual limits are study-specific [11]	Quantitative morphology QC

Best-fit sphere radius and residuals are reported from the segmented femoral head. Published 3-D CT/asphericity work supports this measurement family, while the residual QC limits are study-specific [10,11].

Pelvic CT Segmentation and Quantitative Anatomical Analysis

Femur Subsegmentation and Quantitative Parameters

Automated femoral analysis flags left 3D NSA above Hartel true-3D CT IQR; bilateral femoral-offset asymmetry ≥5 mm; limb-length discrepancy 27.2 mm (left shorter); left head-centre-to-lesser L/D above the published interval.

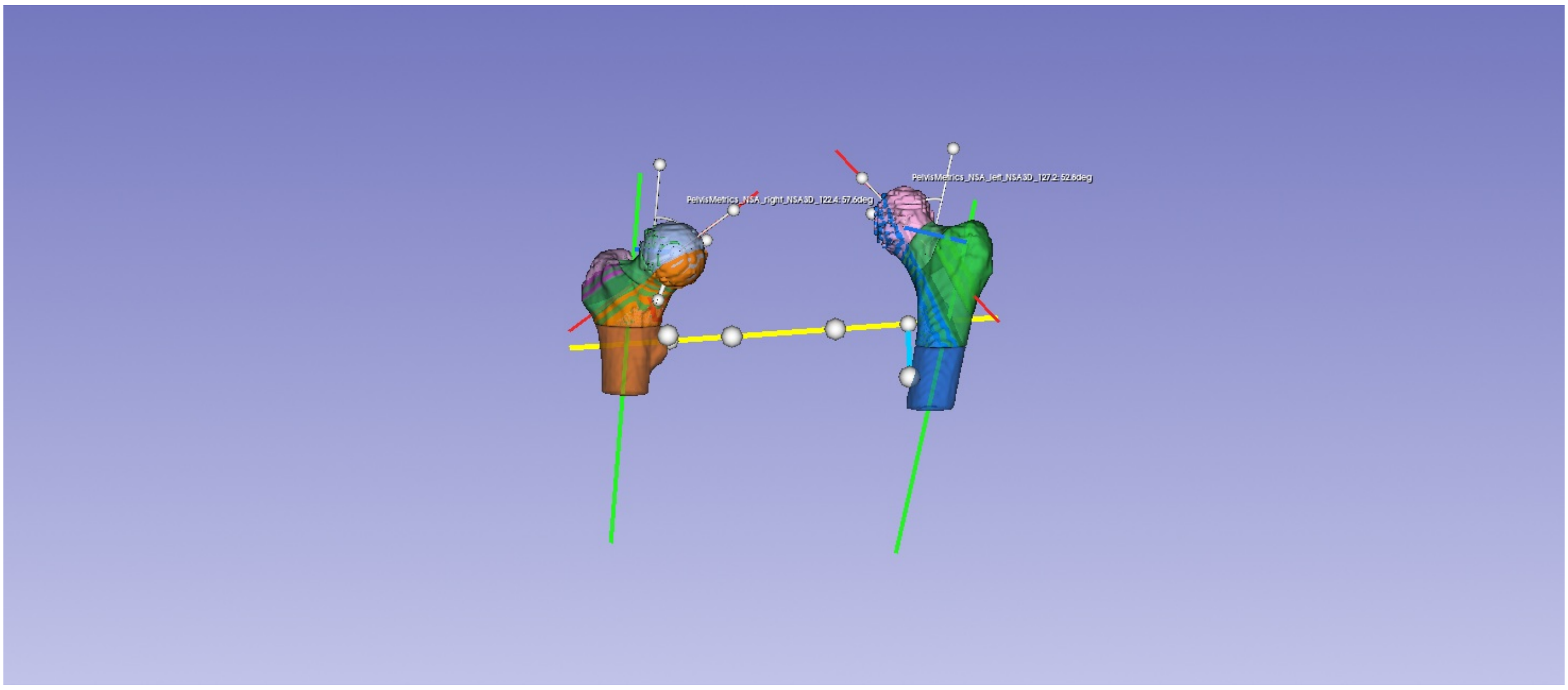

MEASUREMENT	RESULT	REFERENCE / COMPARATOR	INTERPRETATION
True-3D neck-shaft angle (R / L)	**122.4 / 127.17 deg**	Hartel CT IQR 117.9-125.6 deg [18]	WITHIN REFERENCE IQR / FLAGGED HIGH
NSA absolute bilateral difference	**4.8 deg**	Quantitative side comparison; not a stand-alone diagnosis [18]	FLAGGED
Femoral offset (R / L)	**30.49 / 47.25 mm**	Absolute native CT values are quantitative only [19]	FLAGGED ASYMMETRY
Offset absolute side difference	**16.8 mm**	>=5 mm reconstruction-asymmetry comparator [19]	FLAGGED
Limb-length discrepancy	**27.2 mm**	>=10 mm comparator [20]	FLAGGED LLD; Left shorter
Head-centre-to-lesser L/D — Right	**0.783**	AP-radiographic mean 0.84 +/- 0.08; screening interval 0.68-1.00 [21]	L/D is within the published screening interval.
Head-centre-to-lesser L/D — Left	**1.482**	AP-radiographic mean 0.84 +/- 0.08; screening interval 0.68-1.00 [21]	L/D is above the published screening interval; review the lesser point and head geometry.
Integrated femur assessment	**FLAGGED**	Automated quantitative screening; CT/source-segmentation confirmation required	Automated femoral analysis flags left 3D NSA above Hartel true-3D CT IQR; bilateral femoral-offset asymmetry ≥5 mm; limb-length discrepancy 27.2 mm (left shorter); left head-centre-to-lesser L/D above the published interval.

The displayed femur subsegments preserve the locked RAS geometry and existing lesser-trochanter result. NSA and offset use the corrected medullary-centre shaft axis; LLD uses bilateral lower-innominate reference geometry. L/D uses the femur-local AP projection (posterior component excluded). Published comparators are screening references, not stand-alone diagnoses [18-21].

Pelvic CT Segmentation and Quantitative Anatomical Analysis

2. Native Head-Socket Shell and Congruity

RIGHT MARGIN CONTINUOUS; INTACT CONTACT MARGIN; CONGRUENT. LEFT MARGIN CONTINUOUS; INTACT CONTACT MARGIN; SUSPECTED DISLOCATION, POSTERIOR + INFERIOR -- CT-confirmed 36.8 mm displacement relative to the registered expected socket (see section 6).

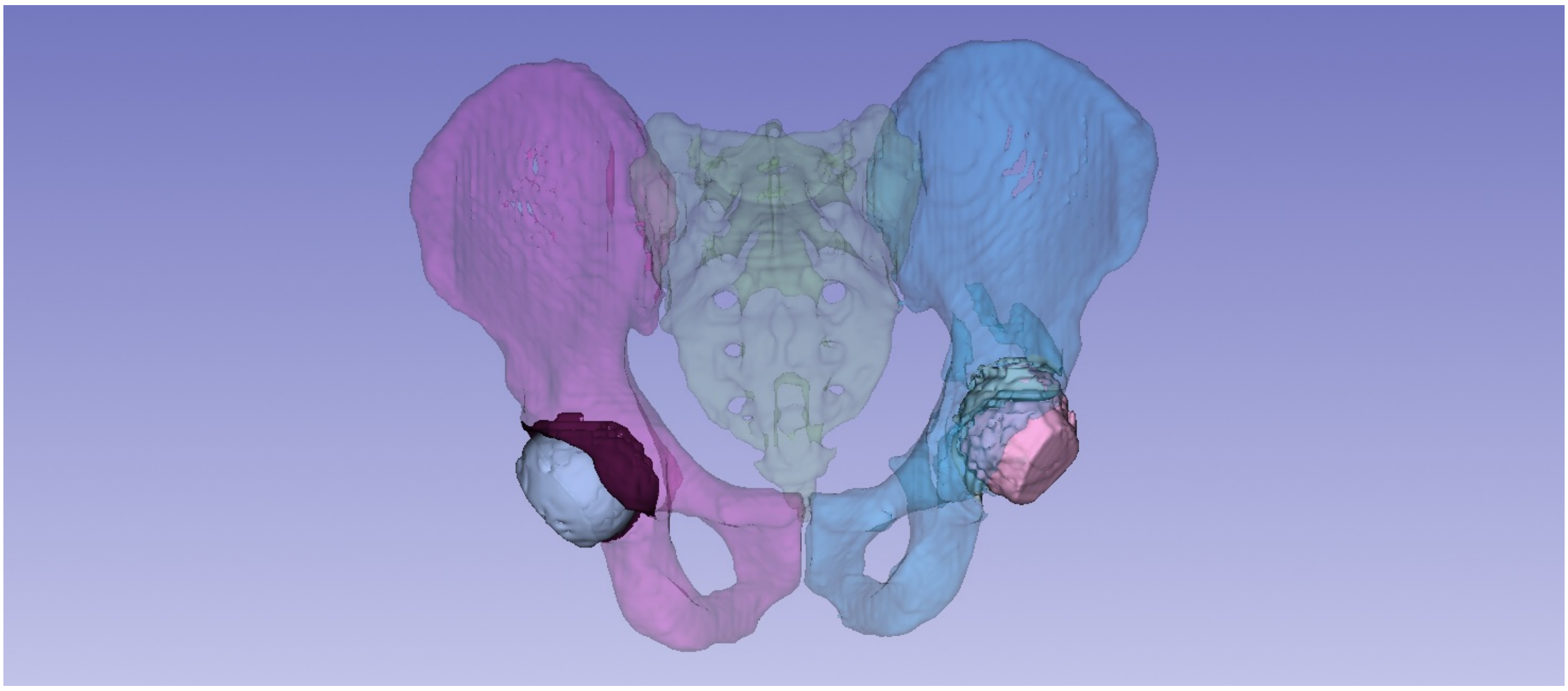

MEASUREMENT	RESULT	REFERENCE / COMPARATOR	INTERPRETATION
Right local gap (median / P95)	**2.4 mm / 5.4 mm**	Healthy-control whole-joint WBCT JSW 3.99 +/- 0.53 mm; different acquisition and geometry [12]	CONGRUENT
Left local gap (median / P95)	**0.4 mm / 5.3 mm**	Healthy-control whole-joint WBCT JSW 3.99 +/- 0.53 mm; different acquisition and geometry [12]	SUSPECTED DISLOCATION, POSTERIOR + INFERIOR (36.8 mm CT-confirmed)

Head-centred rays assess acetabular-shell continuity and local gap. The cited weight-bearing CT joint-space value is shown only as context because the acquisition and geometry differ [12].

Pelvic CT Segmentation and Quantitative Anatomical Analysis

3. Midsagittal Plane and Central-Bone QC

MIDSAGITTAL PLANE QC: PASS — BOTH CENTRAL BONES ARE NEAR-EQUAL; L5 3.90%; SACRUM 0.08%.

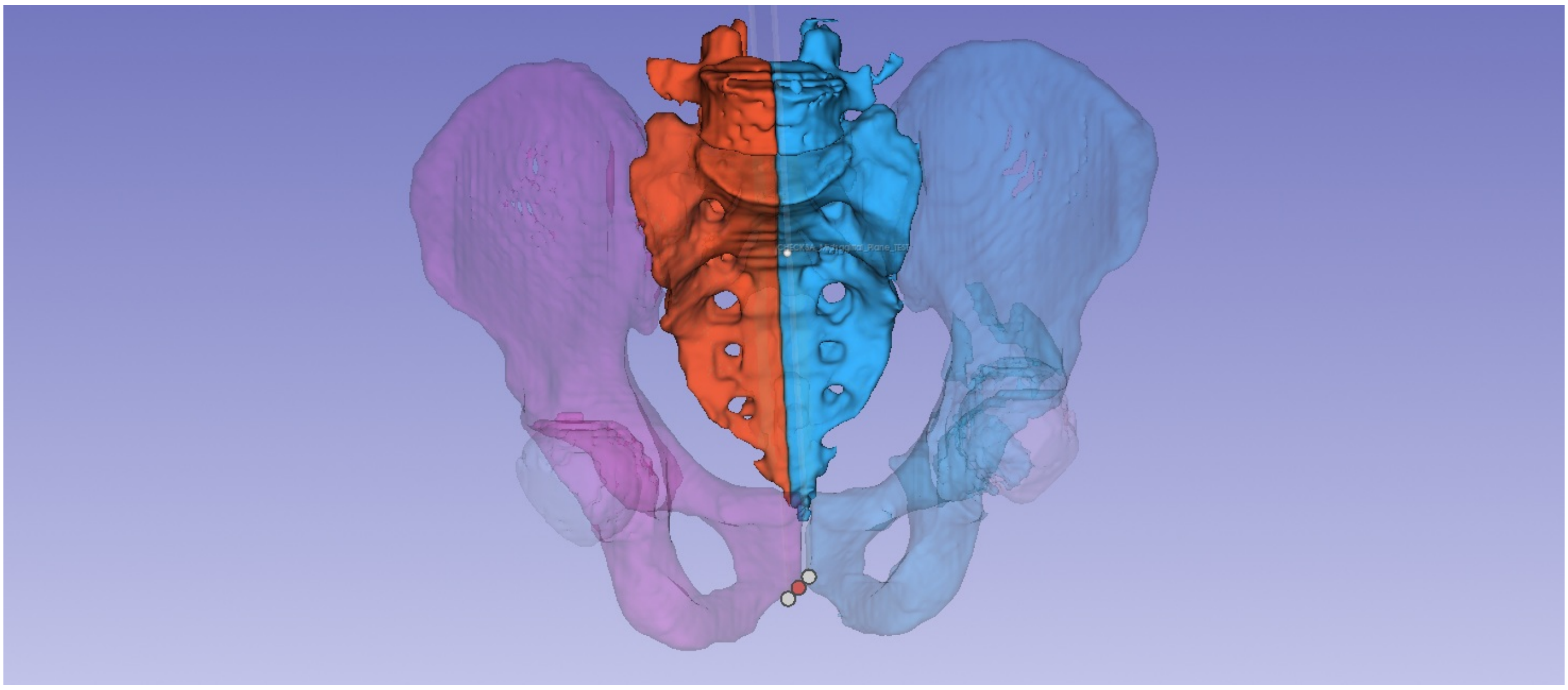

MEASUREMENT	RESULT	REFERENCE / COMPARATOR	INTERPRETATION
L5 bilateral half-volume difference	**3.90%**	Study-internal geometric QC	NEAR-EQUAL
Sacral bilateral half-volume difference	**0.08%**	Study-internal geometric QC	NEAR-EQUAL
Overall plane QC	**PASS — BOTH CENTRAL BONES ARE NEAR-EQUAL**	10% bilateral difference is a study-internal QC rule	Technical plane validation; not a clinical diagnostic cutoff

The pelvic midsagittal plane is accepted only after bilateral L5/sacral half-volume QC. The 10% difference rule is a study-internal geometric QC criterion, not a clinical threshold.

Pelvic CT Segmentation and Quantitative Anatomical Analysis

4. Global Femoral-Head Coverage

GLOBAL FEMORAL-HEAD COVERAGE: RIGHT 45.2%; LEFT 40.4%.

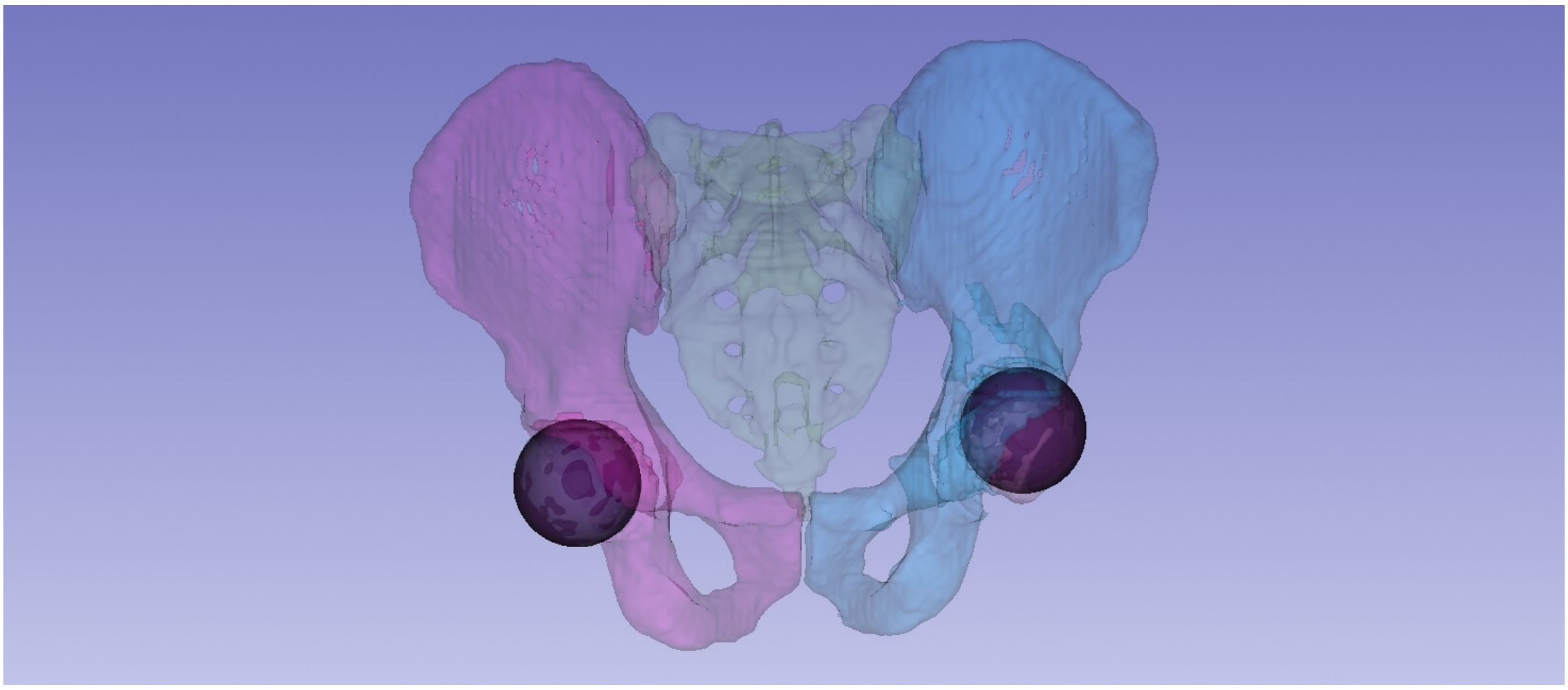

MEASUREMENT	RESULT	REFERENCE / COMPARATOR	INTERPRETATION
Right global coverage	**45.2%**	Asymptomatic CT mean 40 +/- 2%; calculated mean +/-2SD approximately 36-44% [5]	Z=+2.63 | ABOVE APPROXIMATE ASYMPTOMATIC MEAN+2SD — DESCRIPTIVE ONLY
Left global coverage	**40.4%**	Asymptomatic CT mean 40 +/- 2%; calculated mean +/-2SD approximately 36-44% [5]	Z=+0.18 | WITHIN APPROXIMATE ASYMPTOMATIC MEAN+/-2SD

Global curved-surface coverage is measured on a best-fit femoral-head sphere using radial intersection with the acetabular shell. This is an automated adaptation of Larson's method; 40 +/- 2% is the published comparator [5].

Pelvic CT Segmentation and Quantitative Anatomical Analysis

5. Native Original-CT Acetabular Discontinuity — Decisive Ray Test

FLAGGED: Left 4 accepted acetabular discontinuity cluster(s); maximum 3-D span 23.9 mm; 5 mm or larger.

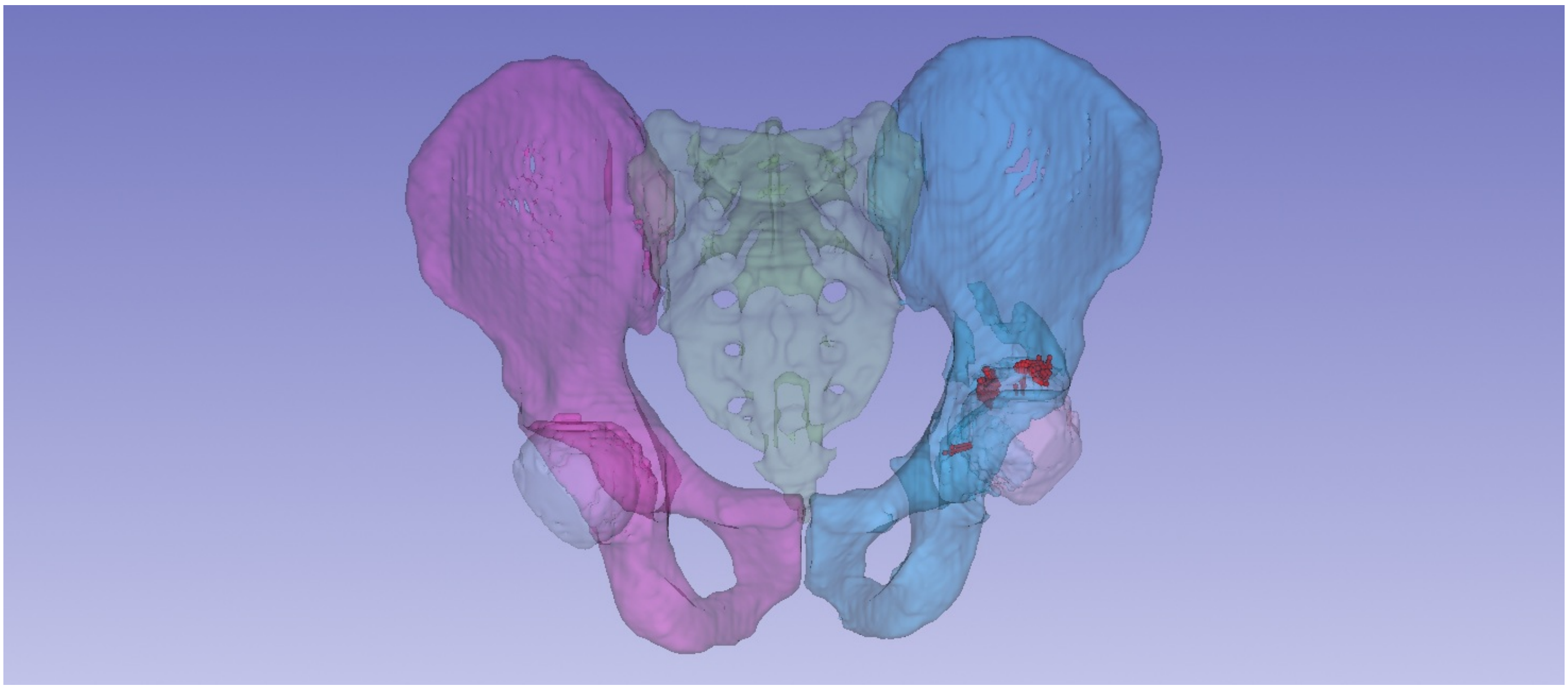

MEASUREMENT	RESULT	REFERENCE / COMPARATOR	INTERPRETATION
Right accepted internal discontinuity clusters	**0**	Original CT support inside the ipsilateral innominate; Check-2 shell supplies expected socket geometry only	NO INTERNAL ACETABULAR DISCONTINUITY DETECTED
Left accepted internal discontinuity clusters	**4**	Original CT support inside the ipsilateral innominate; Check-2 shell supplies expected socket geometry only	INTERNAL ACETABULAR DISCONTINUITY CANDIDATE
Left maximum radial opening	**6.82 mm**	Radial opening is measured along socket-centred rays; it is distinct from 3-D cluster span	Accepted discontinuity geometry
Accepted cluster total	**4**	Native original-CT ray analysis	ABNORMAL RECONSTRUCTION REQUIRED
Maximum radial opening	**6.82 mm**	10-mm route boundary is study-internal and requires cohort validation	ABNORMAL RECONSTRUCTION COMPLETE
Complete no-hit cluster	**YES**	A complete no-hit is handled separately from a measurable finite opening	AT LEAST ONE ACCEPTED CLUSTER IS A COMPLETE NO-HIT GAP; FINITE RADIAL OPENING IS NOT RELIABLY MEASURABLE.
Final acetabular route	**ABNORMAL RECONSTRUCTION COMPLETE**	Native ray + low-only coverage policy + route controller	FULL ABNORMAL CHAIN COMPLETE

Decisive native acetabular fracture-evidence step: the Check-2 shell supplies only the expected socket/angular geometry. Actual bone support is determined from the original CT inside the ipsilateral innominate. The peripheral rim band and inferior rays are excluded. Accepted internal clusters are spatially connected CT-supported outliers/no-hit regions with supported borders. Radial opening is measured along socket-centred rays and is distinct from 3-D cluster span. The 10-mm routing boundary is a study-internal threshold awaiting direct cohort validation.

Pelvic CT Segmentation and Quantitative Anatomical Analysis

6. Registered Expected Native Socket and Head Displacement

LEFT FEMORAL-HEAD DISPLACEMENT: 36.8 mm, POSTERIOR + INFERIOR; CT-CONFIRMED DISPLACED RELATIVE TO REGISTERED EXPECTED SOCKET.

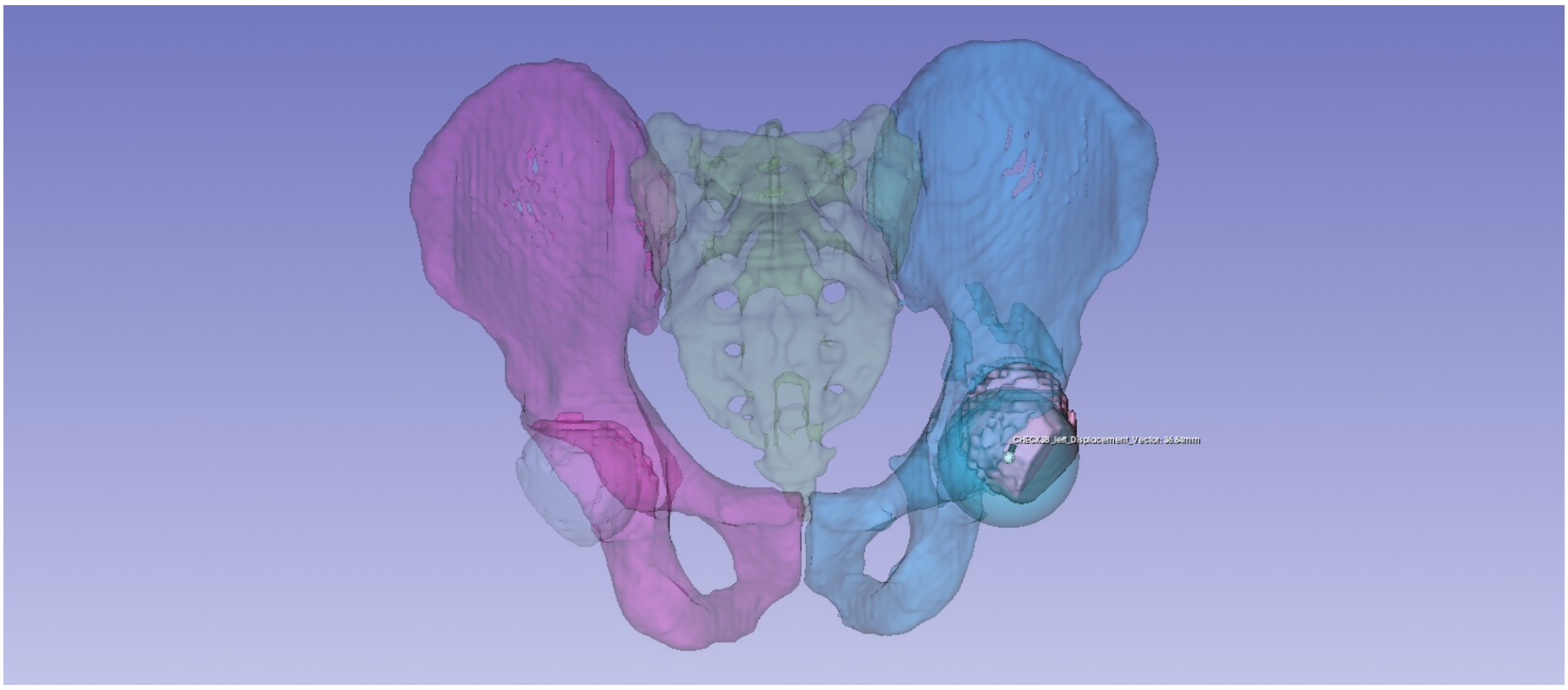

MEASUREMENT	RESULT	REFERENCE / COMPARATOR	INTERPRETATION
3-D displacement	**36.8 mm**	Healthy controls: 1.3 +/- 0.6 mm head-centre translation during external-rotation stress WBCT; not equivalent to routine trauma CT [12]	DISPLACED RELATIVE TO REGISTERED EXPECTED SOCKET
Dominant direction	**POSTERIOR + INFERIOR**	Direction is reported directly from locked Check3B; no ReportBuilder RAS recomputation.	CONGRUENT

Displacement is reported from the actual head centre relative to the registered expected native socket centre. Direction is taken directly from the locked source analysis.

Pelvic CT Segmentation and Quantitative Anatomical Analysis

7. Registered Template Defect Geometry

LARGE POSTERIOR–MEDIAL ACETABULAR DEFECT; NO CENTRAL PENETRATION.

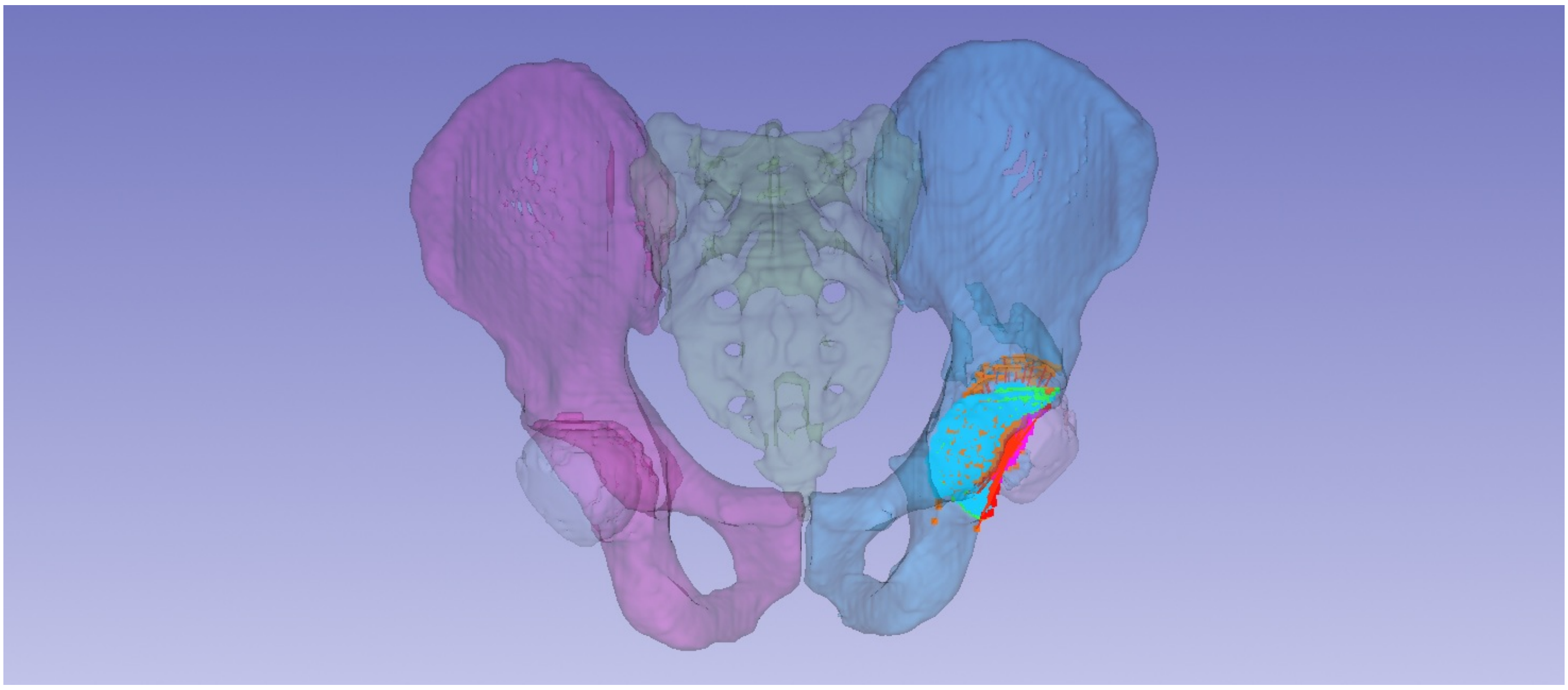

MEASUREMENT	RESULT	REFERENCE / COMPARATOR	INTERPRETATION
Defect class / severity	**ACETABULAR DEFECT / LARGE**	Letournel/Judet provides the anatomical fracture framework, not percentage cutoffs [13]	POSTERIOR–MEDIAL
Defected support	**52.3%**	No validated normal population range for this registered-template metric.	Study-internal technical rule.
Central / medial status	**NO CENTRAL PENETRATION**	central support/displacement cutoffs are internal trial thresholds.	Study-internal technical rule.

Expected, present, displaced, and missing acetabular support are reported by anatomical direction. Judet-Letournel is used only as fracture-anatomy background [13].

Pelvic CT Segmentation and Quantitative Anatomical Analysis

8. Original-CT Support Confirmation

ORIGINAL-CT CONFIRMATION: STATUS CONFIRMED; ACETABULAR DEFECT CONFIRMED; HEAD POSITION CONFIRMED.

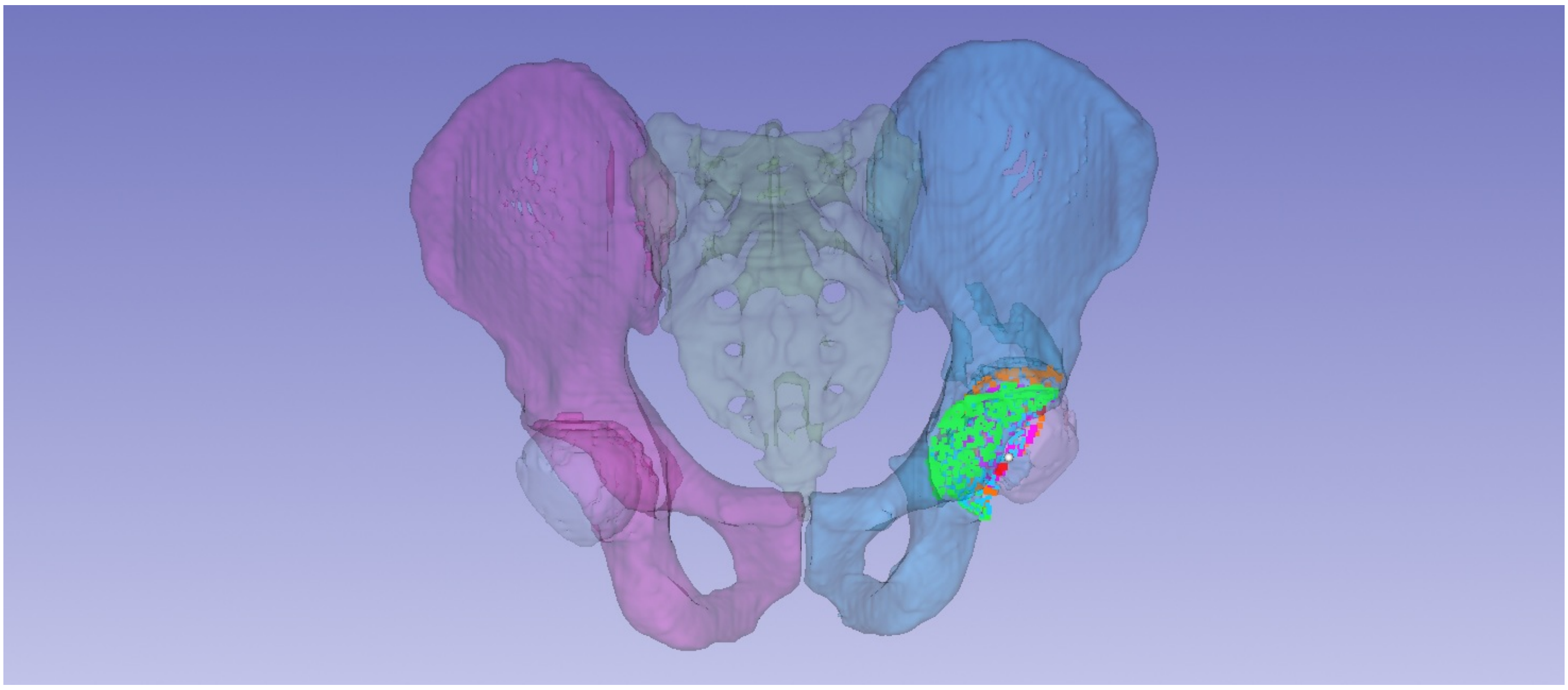

MEASUREMENT	RESULT	REFERENCE / COMPARATOR	INTERPRETATION
CT defect confirmation	**CONFIRMED**	Cortical >300 HU and cancellous 150-300 HU provide tissue context, but ray gates are algorithm-specific [14]	CONFIRMED
CT defected support	**18.8%**	No validated normal range for this CT-support fraction.	Study-internal technical rule.
Median support offset	**0.0 mm**	No validated clinical cutoff for the registered-template offset metric.	Study-internal technical rule.

Original scalar CT is sampled along the registered expected socket to assess actual bony support. HU and percentage gates are technical analysis parameters; published HU ranges are tissue context only [14].

Pelvic CT Segmentation and Quantitative Anatomical Analysis

9. Registered Expected-Socket Original-CT Ray Confirmation

FLAGGED: Left 1 accepted acetabular discontinuity cluster(s); maximum 3-D span 7.2 mm; 5 mm or larger.

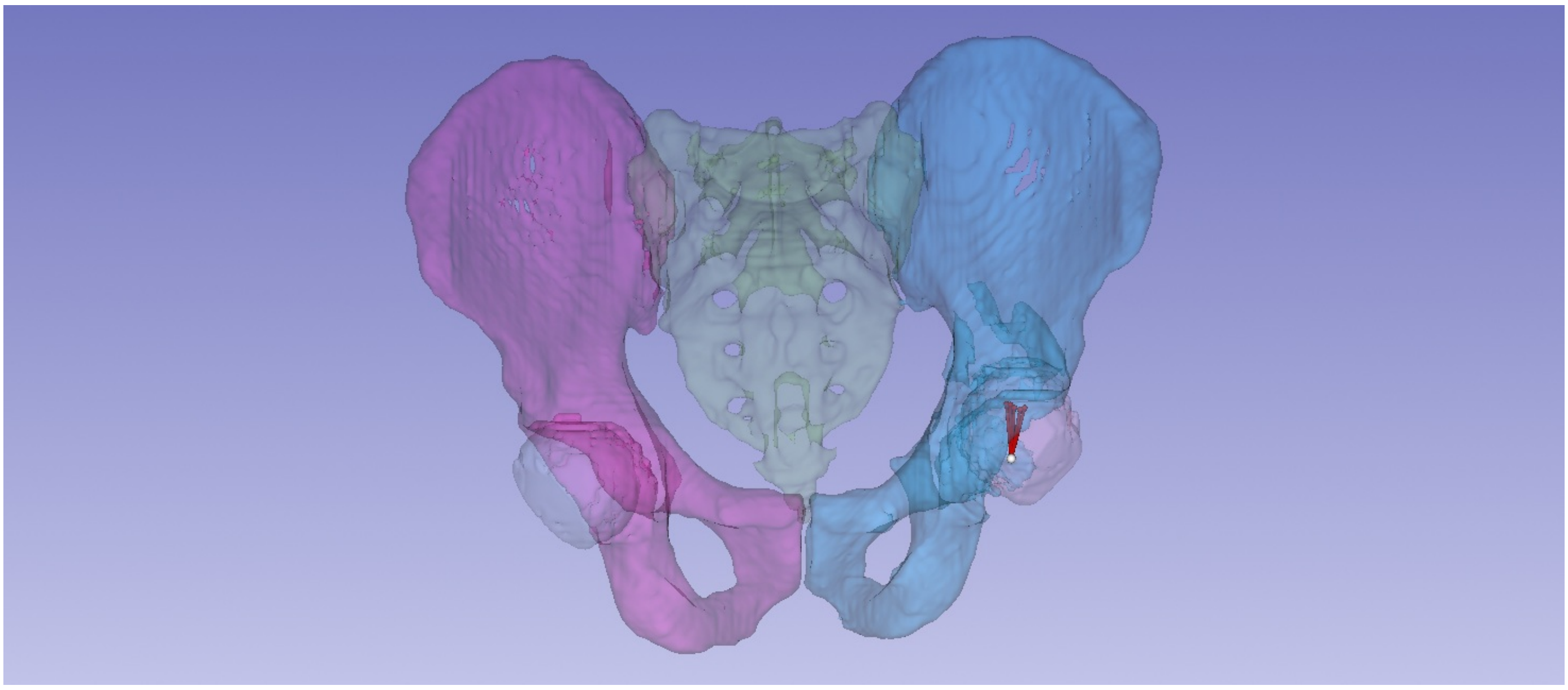

MEASUREMENT	RESULT	REFERENCE / COMPARATOR	INTERPRETATION
Abnormal side	**LEFT**	Registered expected native socket geometry	Original CT remains the bone-evidence source
Accepted CT discontinuity clusters	**1**	Expected/template geometry is never counted as bone	CT-CONFIRMED INTERNAL ACETABULAR DISCONTINUITY: POSTERIOR-SUPERIOR
Cluster 1 radial opening	**3.14 mm**	Measured against the registered expected native socket using original CT	POSTERIOR-SUPERIOR; MILD WITH PARTIAL NO-HIT GAP

For the registered abnormal reconstruction route, the contralateral socket is only an initial geometric prior and is rigidly registered to stable ipsilateral extra-acetabular anatomy. The registered expected socket defines where bone is expected; only original CT support inside the actual abnormal-side innominate can satisfy a ray. Template geometry is never counted as bone.

Pelvic CT Segmentation and Quantitative Anatomical Analysis

10. CT-Supported Socket Reconstruction

CT-supported socket reconstruction completed on LEFT; 1 CT-matched displaced fragment candidate(s) recovered.

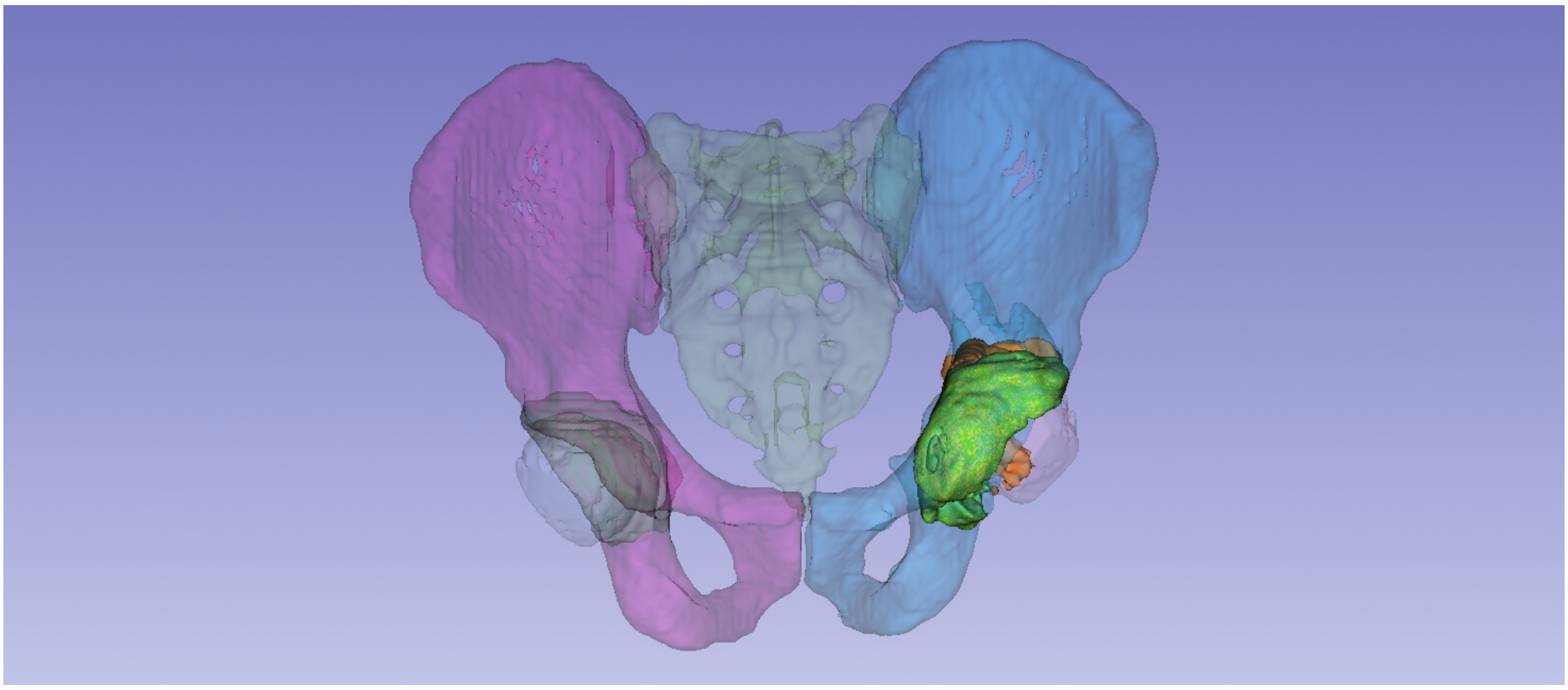

MEASUREMENT	RESULT	REFERENCE / COMPARATOR	INTERPRETATION
Adaptive status	**CREATED**	No population normal range; reconstruction step only.	Technical reconstruction
Recovered fragments	**1**	No validated clinical cutoff.	CT-matched candidate fragments

CT-supported displaced acetabular material is evaluated within the abnormal reconstruction route. This page reports reconstruction output rather than a population diagnostic cutoff.

Pelvic CT Segmentation and Quantitative Anatomical Analysis

11. Final Acetabular Socket

Final acetabular socket reconstruction completed on LEFT; original-CT acetabular defect confirmation: CONFIRMED; NO CENTRAL PENETRATION.

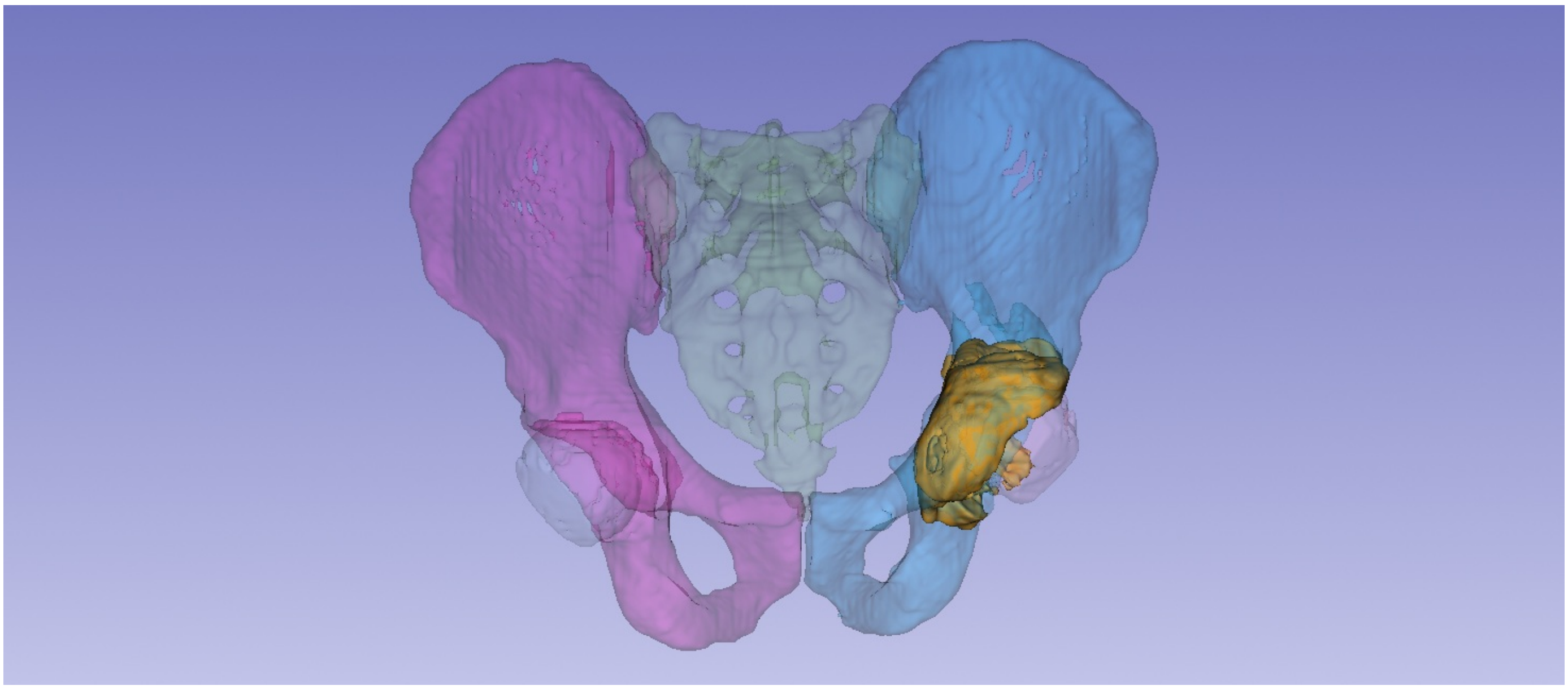

MEASUREMENT	RESULT	REFERENCE / COMPARATOR	INTERPRETATION
Reconstruction status	**COMPLETED**	Final patient-specific socket reconstruction stage	LEFT acetabulum
Original-CT defect confirmation	**CONFIRMED**	Original CT support confirmation from the completed reconstruction chain	NO CENTRAL PENETRATION
CT-matched displaced fragments	**1**	Candidate fragments recovered by the CT-supported reconstruction step	Patient-specific reconstruction output

Final patient-specific socket support is summarized against the registered expected anatomy. Contralateral anatomy is used as a patient-specific geometric prior, not perfect ground truth [15].

Pelvic CT Segmentation and Quantitative Anatomical Analysis

Pubic Symphysis

PUBIC SYMPHYSIS: REFERENCE-COMPATIBLE WIDTH; GAP 1.4 mm, AP/SI SHEAR -0.1 mm/0.1 mm.

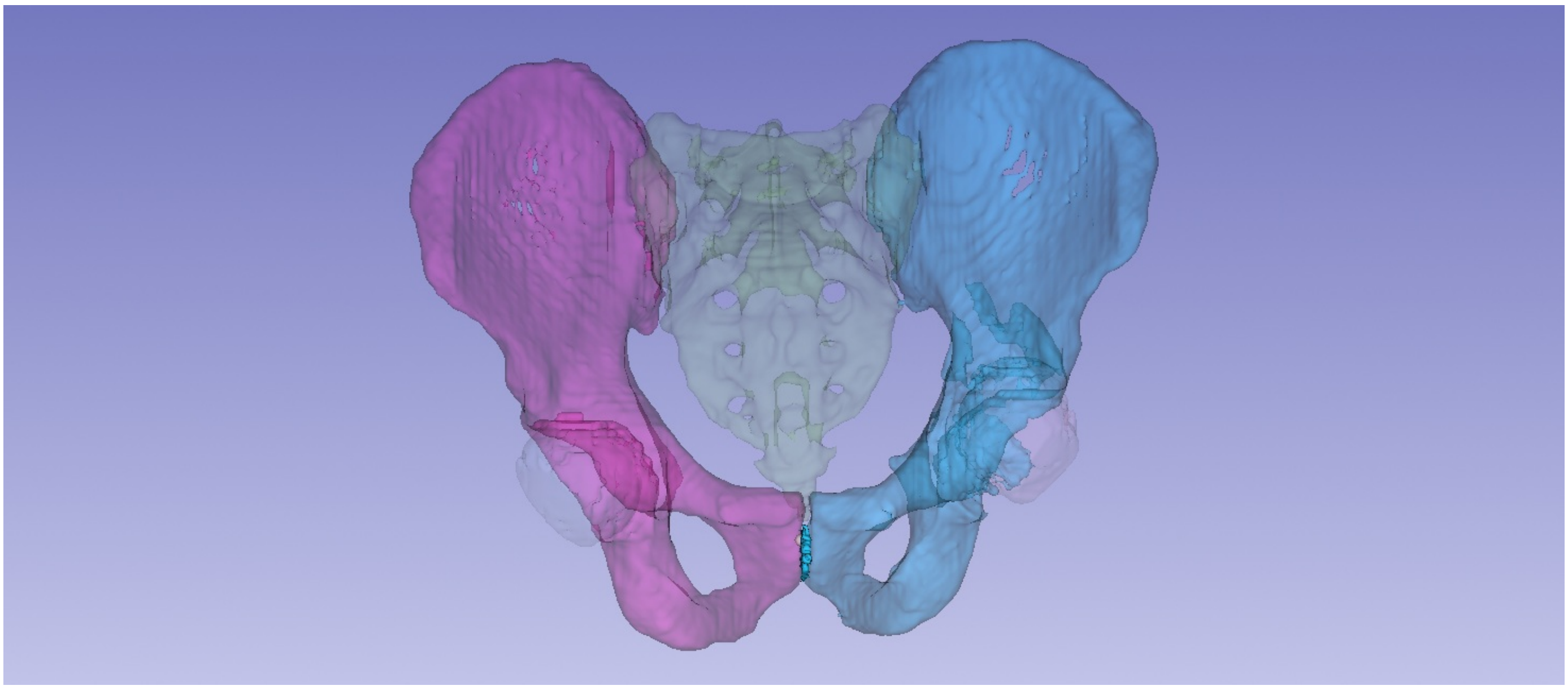

MEASUREMENT	RESULT	REFERENCE / COMPARATOR	INTERPRETATION
Narrowest symphyseal gap	**1.4 mm**	Manual axial-CT mean 3.1 +/- 1.5 mm; calculated mean +/-2SD approximately 0.1-6.1 mm [1]	NO CT EVIDENCE OF SYMPHYSEAL DIASTASIS
AP / SI shear	**-0.1 mm / 0.1 mm**	No validated static-CT cutoff for this automated measurement; the published 5-mm value refers to dynamic flamingo radiographs [4]	Descriptive only

Narrowest stable left-right symphyseal gap. Hwang et al. used a manual axial-CT measurement; the calculated 6.1-mm mean+2SD value is descriptive rather than diagnostic [1].

Pelvic CT Segmentation and Quantitative Anatomical Analysis

Obturator Ring

OBTURATOR RING: Right no reportable rim discontinuity; Left no reportable rim discontinuity.

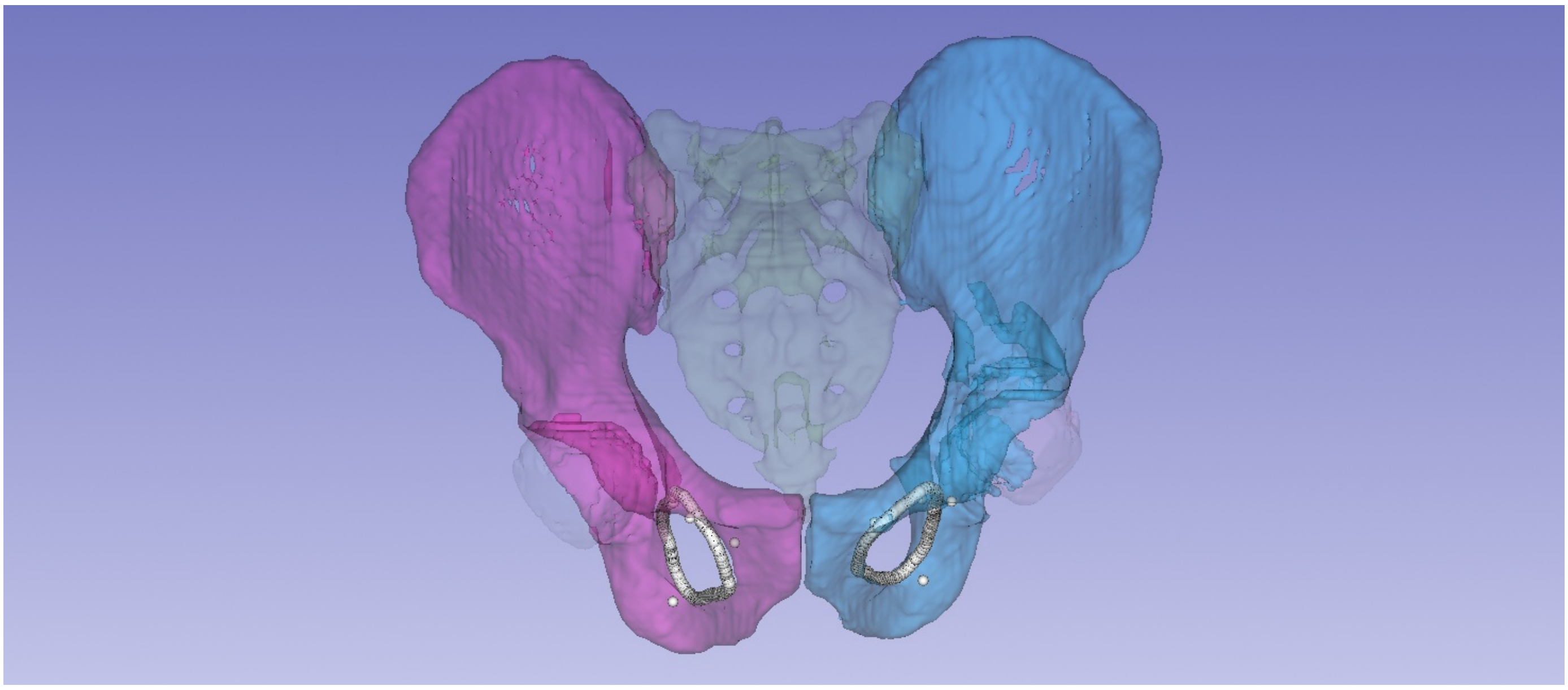

MEASUREMENT	RESULT	REFERENCE / COMPARATOR	INTERPRETATION
Right AP / SI extent	**49.0 mm / 40.9 mm**	Dry-bone mean AP 52.4 +/- 8.0 mm; SI 46.9 +/- 18.3 mm [7]	Descriptive dry-bone comparator only
Right rim discontinuity	**0**	4-mm registered pelvic asymmetry level is an alert comparator, not a fracture cutoff [6]	Visual confirmation required
Left AP / SI extent	**48.0 mm / 35.0 mm**	Dry-bone mean AP 51.7 +/- 8.7 mm; SI 46.6 +/- 16.9 mm [7]	Descriptive dry-bone comparator only
Left rim discontinuity	**0**	4-mm registered pelvic asymmetry level is an alert comparator, not a fracture cutoff [6]	Visual confirmation required

The inner obturator rim is tracked through 360 degrees. The 4-mm literature value is a registered pelvic-asymmetry comparator, not a fracture cutoff; dry-bone AP/SI dimensions are descriptive only [6,7].

Pelvic CT Segmentation and Quantitative Anatomical Analysis

Sacroiliac Joints

SACROILIAC JOINTS ASSESSED: NO LARGE DIASTASIS BY PROVISIONAL CT PROFILE SCREEN; P95 side difference axial 0.2 mm / coronal 0.0 mm; width normal width compatible.

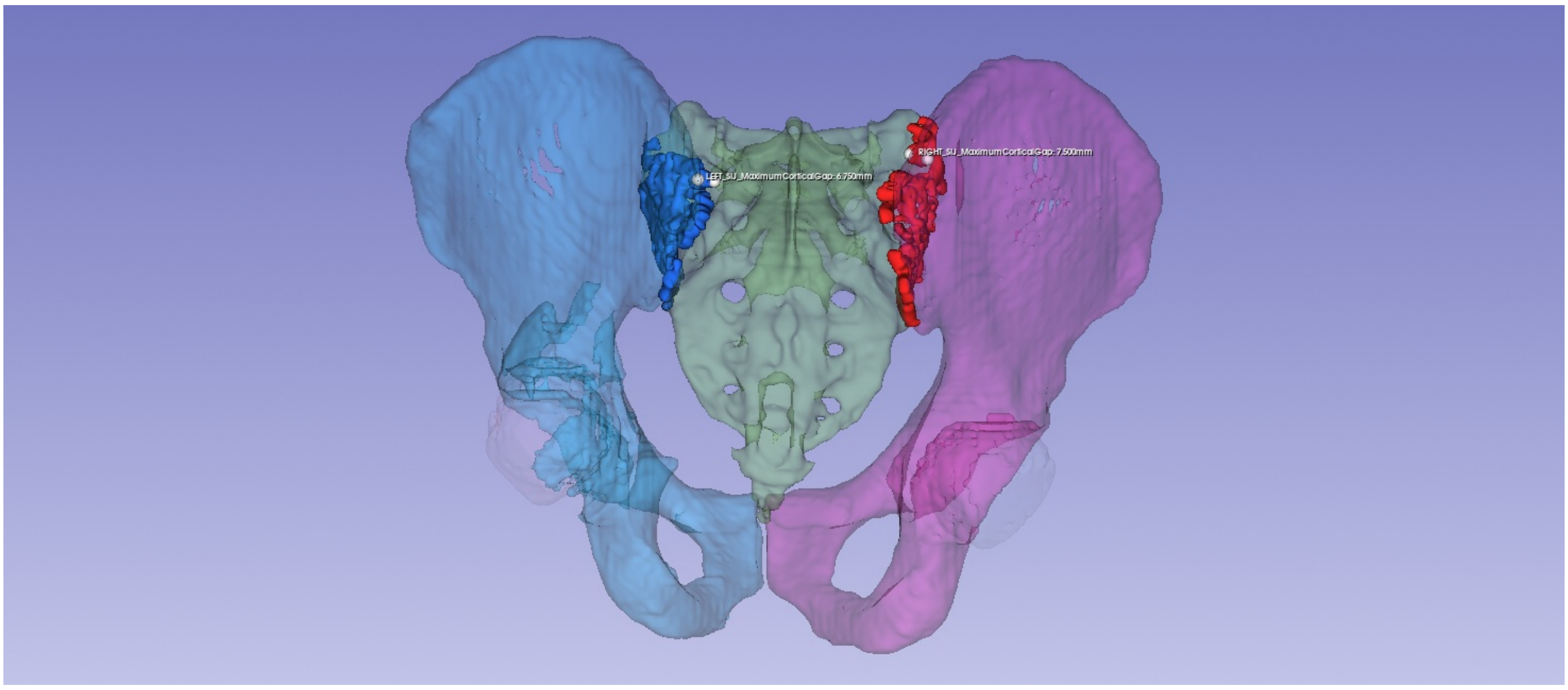

MEASUREMENT	RESULT	REFERENCE / COMPARATOR	INTERPRETATION
Right median cortical gap	**2.2 mm**	Published CT reference: 2.3 +/- 0.4 mm for patients <40 y and 1.9 +/- 0.2 mm for patients >40 y; patient age not supplied [8]	NORMAL WIDTH COMPATIBLE
Left median cortical gap	**2.0 mm**	Published CT reference: 2.3 +/- 0.4 mm for patients <40 y and 1.9 +/- 0.2 mm for patients >40 y; patient age not supplied [8]	NORMAL WIDTH COMPATIBLE
Axial / coronal side difference	**0.2 mm / 0.0 mm**	Study-specific open-reduction context: 3.39 / 3.71 mm; absolute 6.52 / 7.26 mm [9]	NO LARGE DIASTASIS BY PROVISIONAL CT PROFILE SCREEN

Opposing sacral and iliac cortices localize the SI joint, and original CT is sampled along local normals. Published normal widths and trauma cut-points are comparators because the automated 3-D construction differs from manual MPR measurements [8,9].

Pelvic CT Segmentation and Quantitative Anatomical Analysis

Final Innominate Partition

FINAL INNOMINATE PARTITION: EXACT BILATERAL RECONSTRUCTION. Current method: ROOT10 single-plane pubis/ ischium split.

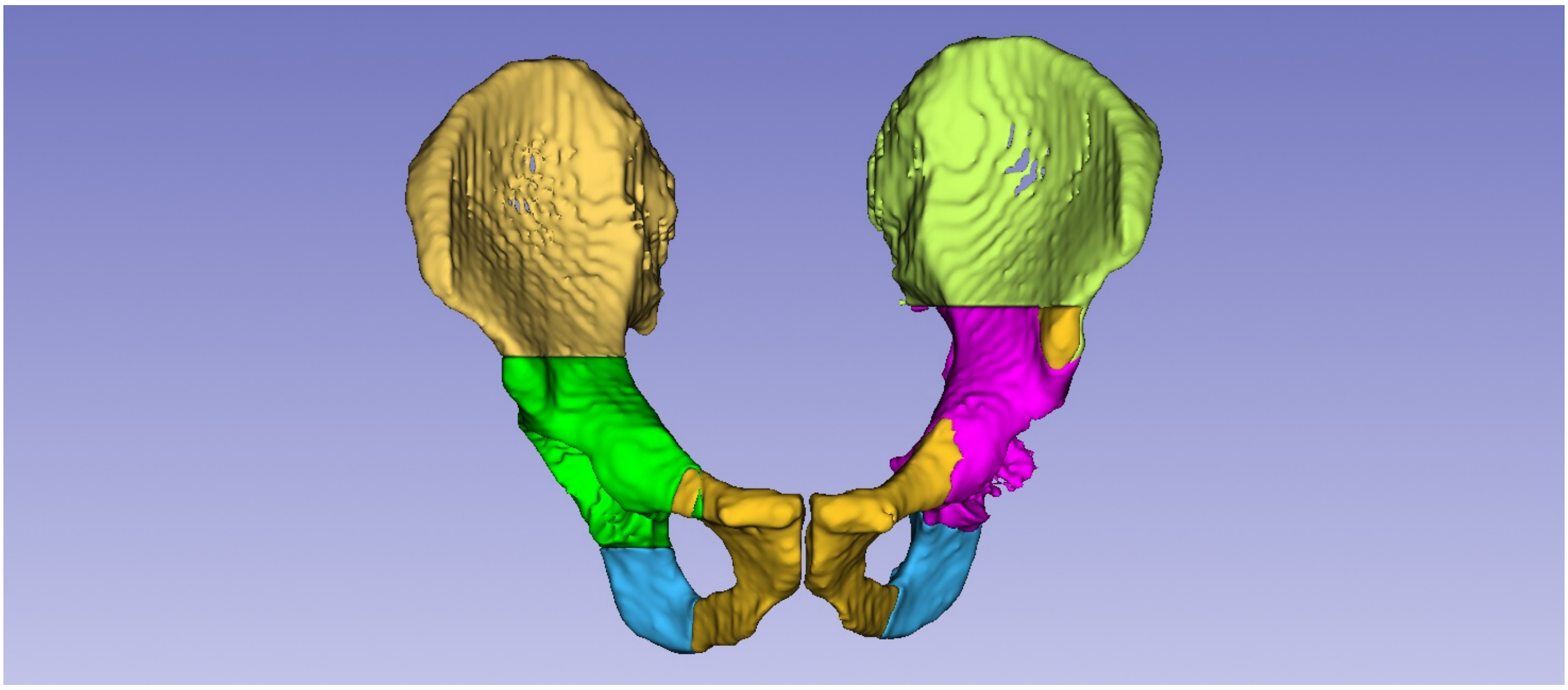

MEASUREMENT	RESULT	REFERENCE / COMPARATOR	INTERPRETATION
Right reconstruction	**PASS**	Technical requirement: zero overlap/unassigned/ outside voxels.	ROOT10 single-plane pubis/ ischium split
Left reconstruction	**PASS**	Technical requirement: zero overlap/unassigned/ outside voxels.	ROOT10 single-plane pubis/ ischium split

The innominate is reconstructed as mutually exclusive acetabulum, ilium, pubis, and ischium. Zero overlap, unassigned, and outside voxels are technical consistency requirements.

Pelvic CT Segmentation and Quantitative Anatomical Analysis

Technical Measurements - Page 1

**ROUTE / QUALITY CONTROL**	
Report status	VALID
Initial pathway	NORMAL
Final state	ABNORMAL RECONSTRUCTION COMPLETE
Midsagittal-plane QC	PASS — BOTH CENTRAL BONES ARE NEAR-EQUAL
**1. FEMORAL-HEAD MORPHOLOGY AND CENTRE - TECHNICAL MEASUREMENTS**	
**RIGHT**	
Centre confidence	HIGH
Fitted radius	24.54 mm
Sphere RMS	0.48 mm
Normalized RMS	1.95%
P95 residual	1.05 mm
Meaningful components	1
**LEFT**	
Centre confidence	MODERATE
Fitted radius	23.77 mm
Sphere RMS	2.03 mm
Normalized RMS	8.52%
P95 residual	3.99 mm
Meaningful components	1
**BILATERAL QC**	
Radius difference	0.77 mm
Radius difference	3.19%
Bilateral radius QC	PASS
**2. NATIVE HEAD-SOCKET SHELL AND CONGRUITY - TECHNICAL MEASUREMENTS**	
**RIGHT**	
Margin status	CONTINUOUS
Structure class	INTACT CONTACT MARGIN
Local congruity	CONGRUENT
Full-head coverage	44.68%
Superior-head coverage	62.25%
Shell area	5252.56 mm²
Meaningful components	1
Median head-to-shell gap	2.38 mm
P95 head-to-shell gap	5.44 mm
**LEFT**	
Margin status	CONTINUOUS
Structure class	INTACT CONTACT MARGIN
Local congruity	CONGRUENT
Full-head coverage	41.30%
Superior-head coverage	50.33%
Shell area	4676.94 mm²

Pelvic CT Segmentation and Quantitative Anatomical Analysis

Technical Measurements - Page 2

Meaningful components	1
Median head-to-shell gap	0.38 mm
P95 head-to-shell gap	5.35 mm
**3. MIDSAGITTAL PLANE AND CENTRAL-BONE QC - TECHNICAL MEASUREMENTS**	
Overall plane QC	PASS — BOTH CENTRAL BONES ARE NEAR-EQUAL
**L5**	
RIGHT volume	32812.58 mm3
LEFT volume	31557.61 mm3
Bilateral difference	3.90%
Smaller/larger ratio	0.9618
Symmetry QC	NEAR-EQUAL
**Sacrum**	
RIGHT volume	114310.15 mm3
LEFT volume	114397.83 mm3
Bilateral difference	0.08%
Smaller/larger ratio	0.9992
Symmetry QC	NEAR-EQUAL
**5. NATIVE ORIGINAL-CT ACETABULAR DISCONTINUITY — DECISIVE RAY TEST - TECHNICAL MEASUREMENTS**	
Accepted cluster total	4
Maximum radial opening	6.82 mm
Complete no-hit cluster	True
Radial opening routing gate	10.00 mm
Ray route	ABNORMAL RECONSTRUCTION REQUIRED
Routing basis	At least one accepted cluster is a complete no-hit gap; finite radial opening is not reliably measurable.
**RIGHT**	
Internal rays tested	1987
CT-supported internal rays	1987
Raw internal candidates	0
Accepted defect rays	0
Accepted defect clusters	0
Maximum side radial opening	**NA**
Complete no-hit cluster	False
Defect status	NO INTERNAL ACETABULAR DISCONTINUITY DETECTED
**LEFT**	
Internal rays tested	1716
CT-supported internal rays	1602
Raw internal candidates	94
Accepted defect rays	93
Accepted defect clusters	4
Maximum side radial opening	6.82 mm
Complete no-hit cluster	True

Pelvic CT Segmentation and Quantitative Anatomical Analysis

Technical Measurements - Page 3

Defect status	INTERNAL ACETABULAR DISCONTINUITY CANDIDATE
Cluster 1 location	ANTERIOR-SUPERIOR
Cluster 1 rays	33
Cluster 1 3-D span	14.05 mm
Cluster 1 radial opening	4.18 mm
Cluster 1 class	MILD WITH PARTIAL NO-HIT GAP
Cluster 2 location	POSTERIOR-SUPERIOR
Cluster 2 rays	53
Cluster 2 3-D span	23.87 mm
Cluster 2 radial opening	6.82 mm
Cluster 2 class	MODERATE WITH PARTIAL NO-HIT GAP
Cluster 3 location	POSTERIOR
Cluster 3 rays	4
Cluster 3 3-D span	6.26 mm
Cluster 3 radial opening	**NA**
Cluster 3 class	GAP / NO CT-SUPPORTED BONE
Cluster 4 location	ANTERIOR
Cluster 4 rays	3
Cluster 4 3-D span	4.20 mm
Cluster 4 radial opening	**NA**
Cluster 4 class	GAP / NO CT-SUPPORTED BONE
**6. REGISTERED EXPECTED NATIVE SOCKET AND HEAD DISPLACEMENT - TECHNICAL MEASUREMENTS**	
Abnormal side	LEFT
Total displacement	36.84 mm
Anterior component	-35.14 mm
Superior component	-9.50 mm
Medial component	-5.68 mm
Canonical direction	POSTERIOR + INFERIOR
Position class	DISPLACED RELATIVE TO REGISTERED EXPECTED SOCKET
Local congruity	CONGRUENT
Original-CT position confirmation	CONFIRMED
Direction report QC	SOURCE_REPORTED
**7. REGISTERED TEMPLATE DEFECT GEOMETRY - TECHNICAL MEASUREMENTS**	
Margin status	DEFECTED
Defect class	ACETABULAR DEFECT
Defect severity	LARGE
Defect direction	POSTERIOR–MEDIAL
Geometric defect percentage	52.33%
Estimated defect area	2748.84 mm^2
Central status	NO CENTRAL PENETRATION
**8. ORIGINAL-CT SUPPORT CONFIRMATION - TECHNICAL MEASUREMENTS**	
CT status	CONFIRMED

Pelvic CT Segmentation and Quantitative Anatomical Analysis

Technical Measurements - Page 4

Acetabular defect confirmation	CONFIRMED
Head-position confirmation	CONFIRMED
CT defect direction	POSTERIOR–MEDIAL
CT central status	NO CENTRAL PENETRATION
Normal-template confirmation	94.31%
Total CT-defected support	18.76%
Median support offset	0.00 mm
P95 support offset	29.00 mm
Head outside expected socket	16.31%
P95 outside depth	33.23 mm
**9. REGISTERED EXPECTED-SOCKET ORIGINAL-CT RAY CONFIRMATION - TECHNICAL MEASUREMENTS**	
Abnormal side	LEFT
Internal rays tested	1081
Internal CT-supported rays	1020
Accepted defect rays	6
Accepted defect clusters	1
Final conclusion	CT-CONFIRMED INTERNAL ACETABULAR DISCONTINUITY: POSTERIOR-SUPERIOR
**10. CT-SUPPORTED SOCKET RECONSTRUCTION - TECHNICAL MEASUREMENTS**	
Adaptive status	CREATED
Pathway	REGISTERED CONTRALATERAL FULL-VOLUME TEMPLATE + MISSING-SECTOR DISPLACEMENT SWEEP + ACTUAL-HEAD SHELL RECOVERY
Head displacement	36.84 mm
Displacement direction	POSTERIOR + INFERIOR
Position class	DISPLACED RELATIVE TO REGISTERED EXPECTED SOCKET
Local congruity	CONGRUENT
CT status	CONFIRMED
Accepted candidate displaced fragments	1
**11. FINAL ACETABULAR SOCKET - TECHNICAL MEASUREMENTS**	
Abnormal side	LEFT
Medial-wall involvement	NO
Original-CT defect confirmation	CONFIRMED
CT central status	NO CENTRAL PENETRATION
Adaptive pathway	REGISTERED CONTRALATERAL FULL-VOLUME TEMPLATE + MISSING-SECTOR DISPLACEMENT SWEEP + ACTUAL-HEAD SHELL RECOVERY
CT-matched candidate displaced fragments	1
**PUBIC SYMPHYSIS - TECHNICAL MEASUREMENTS**	
Clinical status	NORMAL
Screening classification	NO CT EVIDENCE OF SYMPHYSEAL DIASTASIS
Stable narrowest gap	1.43 mm
Selected pair gap	1.41 mm
Three-dimensional point distance	1.42 mm

Pelvic CT Segmentation and Quantitative Anatomical Analysis

Technical Measurements - Page 5

AP shear	-0.08 mm
SI shear	0.12 mm
Supporting local cells	9
Local gap IQR	0.08 mm
**OBTURATOR RING - TECHNICAL MEASUREMENTS**	
**RIGHT**	
AP extent	48.98 mm
SI extent	40.95 mm
Area	1659.90 mm²
Perimeter	162.97 mm
Tracked rim	100.00%
Tracked rim outside 4-mm band	79.17%
Suspected discontinuities	0
**LEFT**	
AP extent	47.99 mm
SI extent	35.02 mm
Area	1560.92 mm²
Perimeter	158.82 mm
Tracked rim	100.00%
Tracked rim outside 4-mm band	58.06%
Suspected discontinuities	0
**SACROILIAC JOINTS - TECHNICAL MEASUREMENTS**	
Width classification	NORMAL WIDTH COMPATIBLE
Median cortical width	2.25 mm
P95 cortical width	4.50 mm
Maximum cortical width	7.50 mm
P95 axial projection	4.45 mm
P95 coronal projection	4.04 mm
CT-validated rays	909
Validation fraction	0.998
Bilateral trauma screen	NO LARGE DIASTASIS BY PROVISIONAL CT PROFILE SCREEN
Width classification	NORMAL WIDTH COMPATIBLE
Median cortical width	2.00 mm
P95 cortical width	4.50 mm
Maximum cortical width	6.75 mm
P95 axial projection	4.22 mm
P95 coronal projection	4.00 mm
CT-validated rays	1018
Validation fraction	1.000
Bilateral trauma screen	NO LARGE DIASTASIS BY PROVISIONAL CT PROFILE SCREEN
**FINAL INNOMINATE PARTITION - TECHNICAL MEASUREMENTS**	
**RIGHT**	

Pelvic CT Segmentation and Quantitative Anatomical Analysis

Technical Measurements - Page 6

Exact reconstruction	True
Unassigned voxels	0
Outside voxels	0
Overlap voxels	0
Acetabulum voxels	257393
Ilium voxels	372811
Pubis voxels	73234
Ischium voxels	50697
**LEFT**	
Exact reconstruction	True
Unassigned voxels	0
Outside voxels	0
Overlap voxels	0
Acetabulum voxels	265359
Ilium voxels	329494
Pubis voxels	82189
Ischium voxels	66544
**CURRENT PARTITION METHOD**	
Method	ROOT10 single-plane pubis/ischium split
Split mode	BOX ANCHOR FINITE20 ROOT CORRIDOR ILIUM FLOOR
Obturator used for split	False
Mirroring used for split	False
8-mm reclamation	False

Pelvic CT Segmentation and Quantitative Anatomical Analysis

References

Published comparators are interpreted according to their original measurement definitions; where the automated measurement differs, comparison is descriptive rather than a diagnostic cutoff.

[1] Hwang K, Wu X, Park CY. Width of pubic symphysis relating to age and sex in Koreans. J Orthop Surg Res. 2021;16(1):430. doi:10.1186/s13018-021-02561-9. PMID:34217332.

[2] Doro CJ, Forward DP, Kim H, Nascone JW, Sciadini MF, Hsieh AH, et al. Does 2.5 cm of symphyseal widening differentiate anteroposterior compression I from anteroposterior compression II pelvic ring injuries? J Orthop Trauma. 2010;24(10):610-615. doi:10.1097/BOT.0b013e3181cff42c. PMID:20871248.

[3] Gansslen A, Lindahl J, Krappinger D, Lindtner RA, Staresinic M. The myth of 2.5 cm symphyseal diastasis. Arch Orthop Trauma Surg. 2025;145(1):306. doi:10.1007/s00402-025-05904-x. PMID:40397156.

[4] Garras DN, Carothers JT, Olson SA. Single-leg-stance (flamingo) radiographs to assess pelvic instability: how much motion is normal? J Bone Joint Surg Am. 2008;90(10):2114-2118. doi:10.2106/JBJS.G.00277. PMID:18829908.

[5] Larson CM, Moreau-Gaudry A, Kelly BT, Byrd JWT, Tonetti J, Lavallee S, et al. Are normal hips being labeled as pathologic? A CT-based method for defining normal acetabular coverage. Clin Orthop Relat Res. 2015;473(4):1247-1254. doi:10.1007/s11999-014-4055-2. PMID:25407391.

[6] Zhang F, Zhang D, Huang Z, Wang Z, Cai X. Morphological asymmetry of pelvic rings: a study based on three-dimensional deviation analysis. Orthop Surg. 2022;14(5):967-976. doi:10.1111/os.13246. PMID:35377554.

[7] Duygu OB, Govsa F. Anatomical structures of the obturator foramen and its importance for pelvic interventions. Surg Radiol Anat. 2025;47(1):163. doi:10.1007/s00276-025-03661-5. PMID:40544414.

[8] Faflia CP, Prassopoulos PK, Daskalogiannaki ME, Gourtsoyiannis NC. Variation in the appearance of the normal sacroiliac joint on pelvic CT. Clin Radiol. 1998;53(10):742-746. doi:10.1016/S0009-9260(98)80316-4. PMID:9817091.

[9] Chien RS, Chen IJ, Lai CY, Chen JP, Yu YH. Critical distance of the sacroiliac joint for open reduction using screw fixation for traumatic sacroiliac joint diastasis: a retrospective study. J Orthop Surg Res. 2024;19(1):268. doi:10.1186/s13018-024-04759-z. PMID:38678298.

[10] Jamali AA, Mak W, Wang P, Tai L, Meehan JP, Lamba R. What is normal femoral head/neck anatomy? An analysis of radial CT reconstructions in adolescents. Clin Orthop Relat Res. 2013;471(11):3581-3587. doi:10.1007/s11999-013-3166-5. PMID:23922189.

[11] Hassan MM, Feroe AG, Douglass BW, Jimenez AE, Kuhns B, Mitchell CF, et al. Three-dimensional analysis of age and sex differences in femoral head asphericity in asymptomatic hips in the United States. World J Orthop. 2024;15(8):754-763. doi:10.5312/wjo.v15.i8.754. PMID:39165879.

[12] Rivas DJL, Gassmann JM, Goetz JE, Aitken HD, Davison JC, Miller A, et al. Weight-bearing CT as an approach to assess femoral-acetabular displacement during external rotation stress in the hip. J Hip Preserv Surg. 2025;12(2):93-104. doi:10.1093/jhps/hnaf001. PMID:40761572.

[13] Judet R, Judet J, Letournel E. Fractures of the acetabulum: classification and surgical approaches for open reduction. Preliminary report. J Bone Joint Surg Am. 1964;46:1615-1646. doi:10.2106/00004623-196446080-00001. PMID:14239854.

[14] Imani M, Buratto J, Dao T, Meijering E, Vogrin S, Kwok TCY, et al. Deep learning technique for automatic segmentation of proximal hip musculoskeletal tissues from CT scan images: a MrOS study. J Cachexia Sarcopenia Muscle. 2025;16(2):e13728. doi:10.1002/jcsm.13728. PMID:40022453.

[15] Ead MS, Duke KK, Jaremko JL, Westover L. Investigation of pelvic symmetry using CAD software. Med Biol Eng Comput. 2020;58(1):75-82. doi:10.1007/s11517-019-02068-w. PMID:31745838.

[16] Amar E, Druckmann I, Flusser G, Safran MR, Salai M, Rath E. The anterior inferior iliac spine: size, position, and location. An anthropometric and sex survey. Arthroscopy. 2013;29(5):874-881. doi:10.1016/j.arthro.2013.01.023. PMID:23523127.

[17] Rana SH, Farjoodi P, Haloman S, Dutton P, Hariri A, Ward SR, et al. Anatomic evaluation of the sacroiliac joint: a radiographic study with implications for procedures. Pain Physician. 2015;18(6):583-592. PMID:26606010.

[18] Hartel MJ, Petersik A, Schmidt A, Kendoff D, Nuchtern J, Rueger JM, et al. Determination of femoral neck angle and torsion angle utilizing a novel three-dimensional modeling and analytical technology based on CT datasets. PLoS One. 2016;11(3):e0149480. doi:10.1371/journal.pone.0149480.

[19] Mahmood SS, Mukka SS, Crnalic S, Wretenberg P, Sayed-Noor AS. Association between changes in global femoral offset after total hip arthroplasty and function, quality of life, and abductor muscle strength. Acta Orthop. 2016;87(1):36-41. doi:10.3109/17453674.2015.1091955.

[20] O'Brien S, Kernohan G. Perception of imposed leg length inequality in normal subjects. Hip Int. 2010;20(4):505-511. doi:10.1177/112070001002000414.

[21] Wang G, et al. Measurement of relative position of femoral head center, greater trochanter, and lesser trochanter. Ann Palliat Med. 2021;10(11):11524-11528. doi:10.21037/apm-21-2538.

Pelvic CT Segmentation and Quantitative Anatomical Analysis

Clinical Findings Summary

Case ID: FEMUR_0007_0000 | Age: Not supplied | Status: VALID

CONCLUSIVE FINDING

Automated CT analysis flags joint-space narrowing on Right and Left; femoral parameter assessment flagged. In addition, 1 small (<5 mm) candidate finding(s) require confirmation. Femoral parameter assessment is flagged; see the dedicated evidence page.

OVERALL STATION ASSESSMENT

Flagged finding of at least 5 mm in the hip/acetabular station group(s).

Clinical station checklist

HIP JOINT AND ACETABULUM

Femoral-head morphology — NO FLAG
R VALID HEAD CENTRE — NORMAL-LIKE MORPHOLOGY / L VALID HEAD CENTRE — NORMAL-LIKE MORPHOLOGY

Head-socket congruity — NO FLAG
R CONGRUENT / L CONGRUENT

Acetabular coverage — NO FLAG
R 44.4% / L 44.6%; no low-coverage flag

Acetabular continuity — CAUTION
2 accepted cluster(s); maximum 3-D span 4.2 mm; small finding may be irrelevant/artifactual; confirm on source CT

Head position / dislocation — NO FLAG
No displacement pathway activated; bilateral native assessment

Joint-space / arthrosis screen — FLAGGED
R NARROWED — OSTEOARTHRITIS CANDIDATE (2.50 / -0.50 / 5.50 mm) / L NARROWED — OSTEOARTHRITIS CANDIDATE (2.50 / -0.50 / 6.50 mm)

Femur parameters — FLAGGED
Automated femoral analysis flags right 3D NSA below Hartel true-3D CT IQR; left 3D NSA below Hartel true-3D CT IQR; bilateral femoral-offset asymmetry ≥5 mm; limb-length discrepancy 14.0 mm (right

ANTERIOR PELVIC RING

Overall anterior ring screen — NO FLAG
No abnormality at all assessed anterior stations

Pubic symphysis — NO FLAG
Gap 1.6 mm; NO CT EVIDENCE OF SYMPHYSEAL DIASTASIS

Right obturator ring — NO FLAG
0 reportable rim discontinuity candidates

Left obturator ring — NO FLAG
0 reportable rim discontinuity candidates

POSTERIOR PELVIC RING

Right SIJ integrity — NO FLAG
median 1.0 mm; P95 5.5 mm; NARROWER THAN AGE REFERENCE

Left SIJ integrity — NO FLAG
median 0.8 mm; P95 5.0 mm; NARROWER THAN AGE REFERENCE

SCOPE: Joint-space narrowing is an arthrosis/coxarthrosis screen, not a stand-alone diagnosis. These station tests do not exclude isolated or minimally displaced iliac or sacral fractures; review the source CT.

SIJ references: normal CT mean 2.3 +/- 0.4 mm (<40 y) and 1.9 +/- 0.2 mm (>40 y) [8]; traumatic axial/coronal distance context [9]; anatomical extent/search-corridor context [17].

Detailed anatomical evidence and quantitative measurements follow on the next pages.

Pelvic CT Segmentation and Quantitative Anatomical Analysis

1. Femoral-Head Morphology and Centre

RIGHT FEMORAL HEAD: VALID HEAD CENTRE — NORMAL-LIKE MORPHOLOGY; CENTRE HIGH CONFIDENCE. LEFT FEMORAL HEAD: VALID HEAD CENTRE — NORMAL-LIKE MORPHOLOGY; CENTRE HIGH CONFIDENCE.

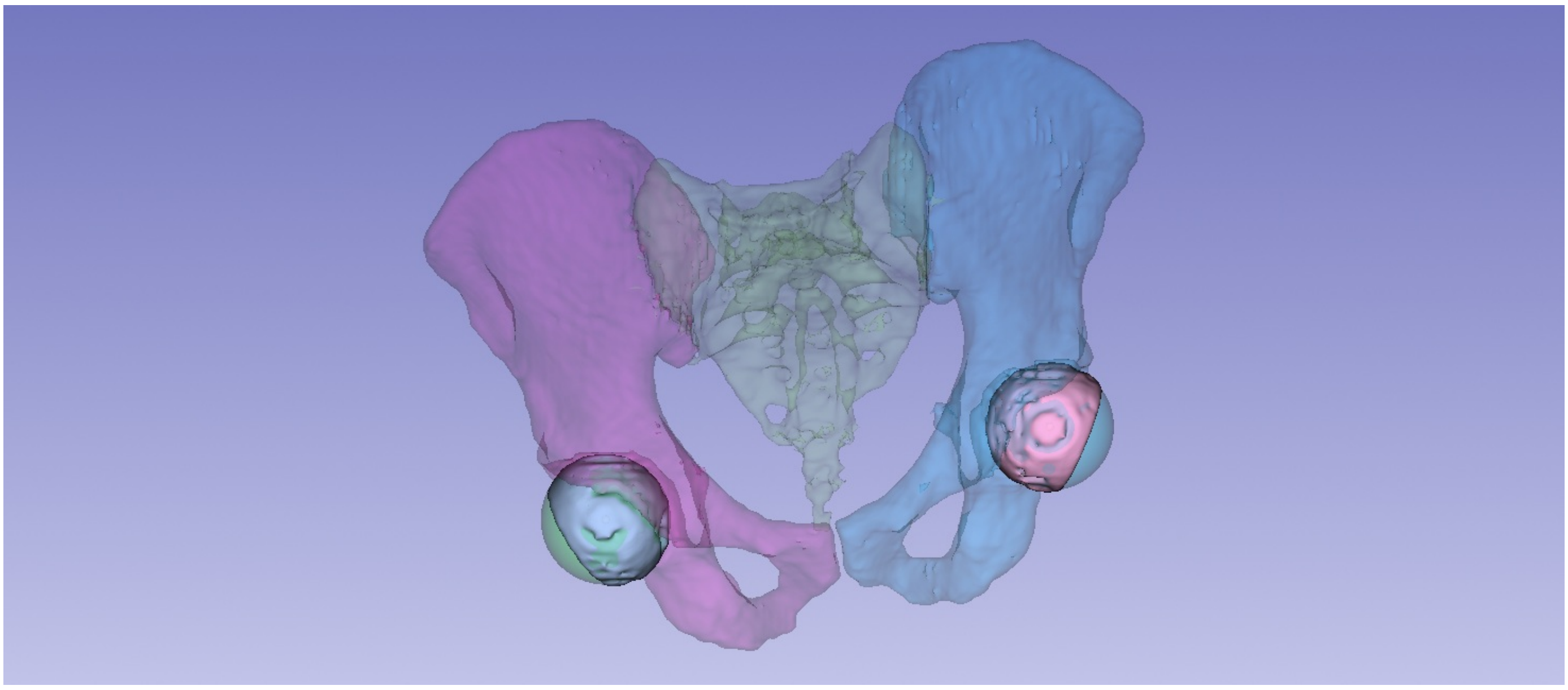

MEASUREMENT	RESULT	REFERENCE / COMPARATOR	INTERPRETATION
Right fitted radius	**25.2 mm**	Adolescent radial-CT comparator: male 24.53 +/- 1.74 mm; female 21.94 +/- 1.13 mm [10]	VALID HEAD CENTRE — NORMAL-LIKE MORPHOLOGY
Left fitted radius	**24.8 mm**	Adolescent radial-CT comparator: male 24.53 +/- 1.74 mm; female 21.94 +/- 1.13 mm [10]	VALID HEAD CENTRE — NORMAL-LIKE MORPHOLOGY
Sphere-fit residual	**R/L P95 1.6 mm/1.4 mm**	Published 3-D sphere/asphericity analysis supports the measurement family; these residual limits are study-specific [11]	Quantitative morphology QC

Best-fit sphere radius and residuals are reported from the segmented femoral head. Published 3-D CT/asphericity work supports this measurement family, while the residual QC limits are study-specific [10,11].

Pelvic CT Segmentation and Quantitative Anatomical Analysis

Femur Subsegmentation and Quantitative Parameters

Automated femoral analysis flags right 3D NSA below Hartel true-3D CT IQR; left 3D NSA below Hartel true-3D CT IQR; bilateral femoral-offset asymmetry ≥5 mm; limb-length discrepancy 14.0 mm (right shorter); right head-centre-to-lesser L/D above the published interval.

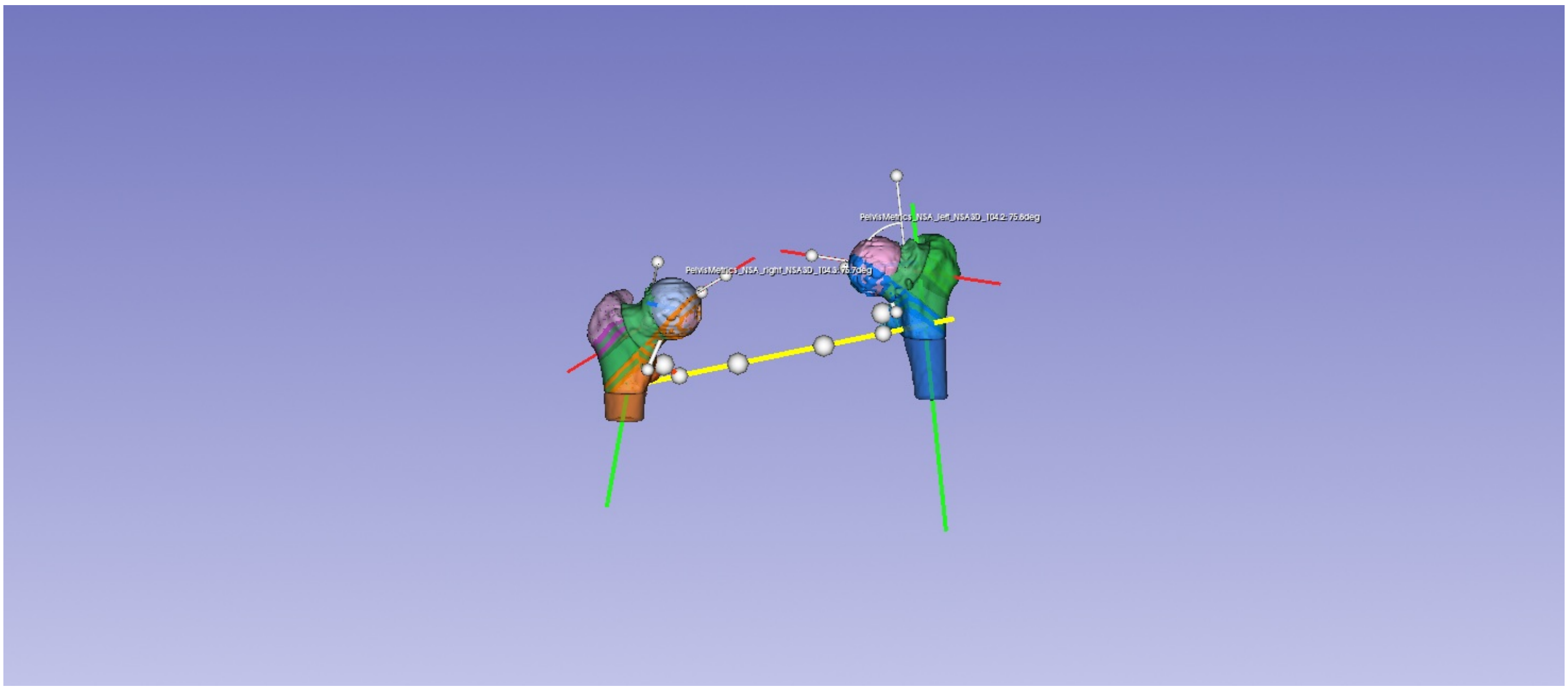

MEASUREMENT	RESULT	REFERENCE / COMPARATOR	INTERPRETATION
True-3D neck-shaft angle (R / L)	**104.3 / 104.2 deg**	Hartel CT IQR 117.9-125.6 deg [18]	FLAGGED LOW / FLAGGED LOW
NSA absolute bilateral difference	**0.1 deg**	Quantitative side comparison; not a stand-alone diagnosis [18]	FLAGGED
Femoral offset (R / L)	**24.04 / 36.43 mm**	Absolute native CT values are quantitative only [19]	FLAGGED ASYMMETRY
Offset absolute side difference	**12.4 mm**	>=5 mm reconstruction-asymmetry comparator [19]	FLAGGED
Limb-length discrepancy	**14.0 mm**	>=10 mm comparator [20]	FLAGGED LLD; Right shorter
Head-centre-to-lesser L/D — Right	**1.148**	AP-radiographic mean 0.84 +/- 0.08; screening interval 0.68-1.00 [21]	L/D is above the published screening interval; review the lesser point and head geometry.
Head-centre-to-lesser L/D — Left	**0.883**	AP-radiographic mean 0.84 +/- 0.08; screening interval 0.68-1.00 [21]	L/D is within the published screening interval.
Integrated femur assessment	**FLAGGED**	Automated quantitative screening; CT/source-segmentation confirmation required	Automated femoral analysis flags right 3D NSA below Hartel true-3D CT IQR; left 3D NSA below Hartel true-3D CT IQR; bilateral femoral-offset asymmetry ≥5 mm; limb-length discrepancy 14.0 mm (right shorter); right head-centre-to-lesser L/D above the published interval.

The displayed femur subsegments preserve the locked RAS geometry and existing lesser-trochanter result. NSA and offset use the corrected medullary-centre shaft axis; LLD uses bilateral lower-innominate reference geometry. L/D uses the femur-local AP projection (posterior component excluded). Published comparators are screening references, not stand-alone diagnoses [18-21].

Pelvic CT Segmentation and Quantitative Anatomical Analysis

2. Native Head-Socket Shell and Congruity

RIGHT MARGIN CONTINUOUS; INTACT CONTACT MARGIN; CONGRUENT. LEFT MARGIN CONTINUOUS; INTACT CONTACT MARGIN; CONGRUENT.

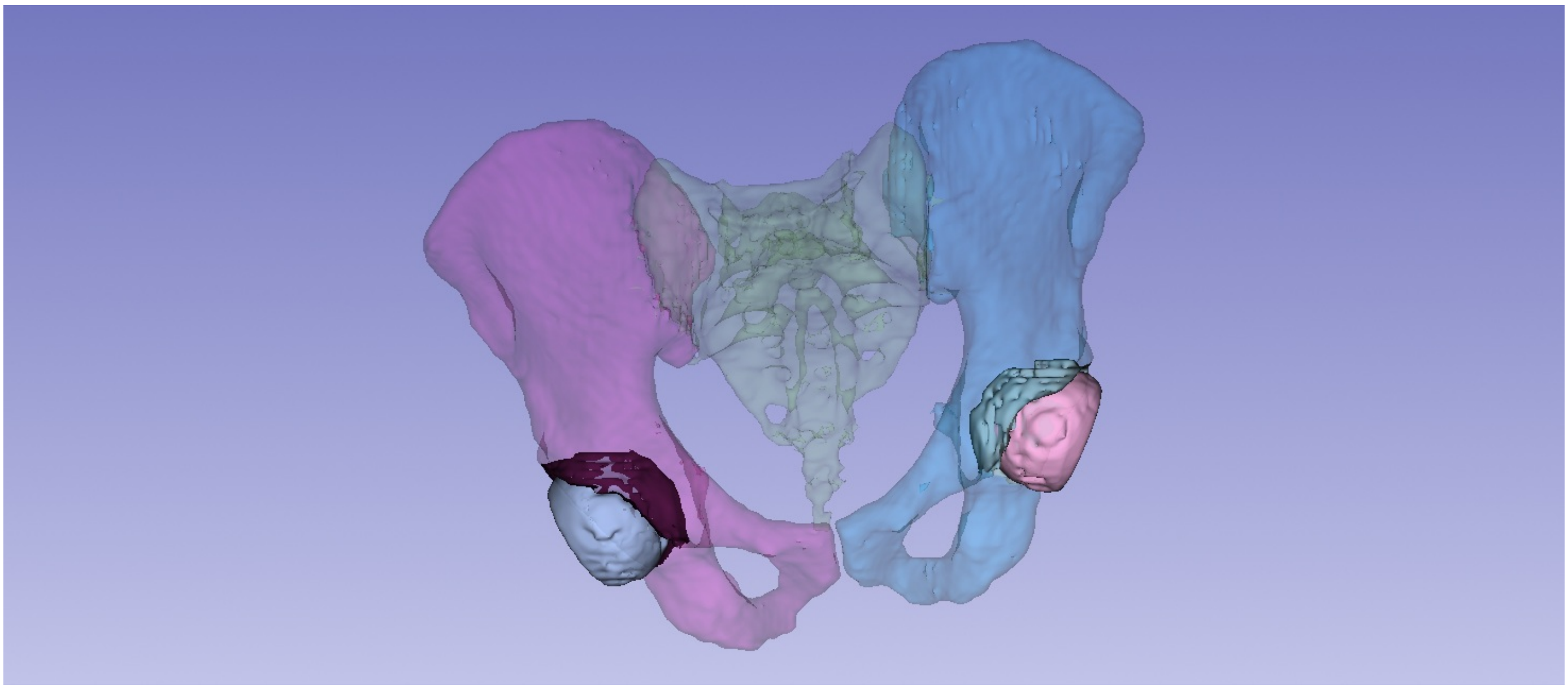

MEASUREMENT	RESULT	REFERENCE / COMPARATOR	INTERPRETATION
Right local gap (median / P95)	**2.1 mm / 5.1 mm**	Healthy-control whole-joint WBCT JSW 3.99 +/- 0.53 mm; different acquisition and geometry [12]	CONGRUENT
Left local gap (median / P95)	**2.0 mm / 6.0 mm**	Healthy-control whole-joint WBCT JSW 3.99 +/- 0.53 mm; different acquisition and geometry [12]	CONGRUENT

Head-centred rays assess acetabular-shell continuity and local gap. The cited weight-bearing CT joint-space value is shown only as context because the acquisition and geometry differ [12].

Pelvic CT Segmentation and Quantitative Anatomical Analysis

3. Midsagittal Plane and Central-Bone QC

MIDSAGITTAL PLANE QC: PASS — BOTH CENTRAL BONES ARE NEAR-EQUAL; L5 1.12%; SACRUM 0.98%.

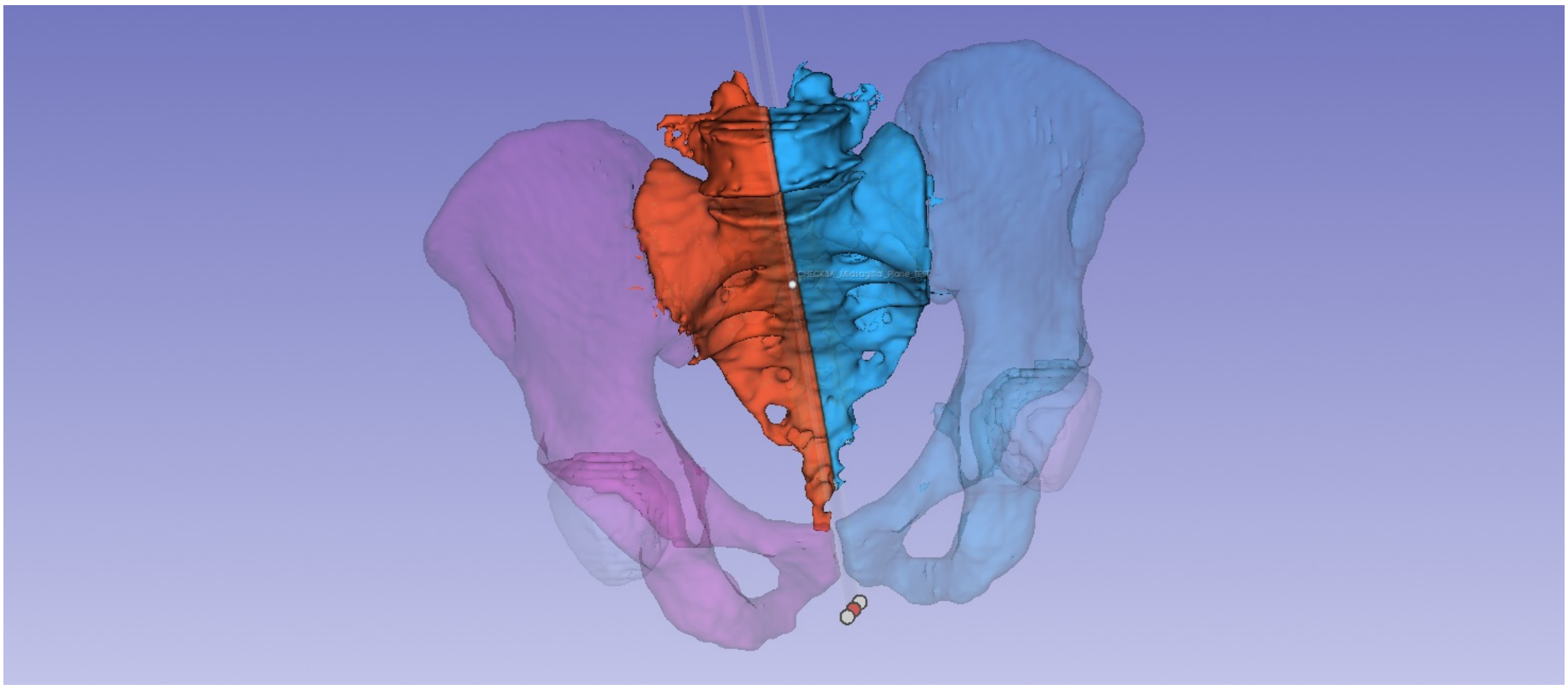

MEASUREMENT	RESULT	REFERENCE / COMPARATOR	INTERPRETATION
L5 bilateral half-volume difference	**1.12%**	Study-internal geometric QC	NEAR-EQUAL
Sacral bilateral half-volume difference	**0.98%**	Study-internal geometric QC	NEAR-EQUAL
Overall plane QC	**PASS — BOTH CENTRAL BONES ARE NEAR-EQUAL**	10% bilateral difference is a study-internal QC rule	Technical plane validation; not a clinical diagnostic cutoff

The pelvic midsagittal plane is accepted only after bilateral L5/sacral half-volume QC. The 10% difference rule is a study-internal geometric QC criterion, not a clinical threshold.

Pelvic CT Segmentation and Quantitative Anatomical Analysis

4. Global Femoral-Head Coverage

GLOBAL FEMORAL-HEAD COVERAGE: RIGHT 44.4%; LEFT 44.6%.

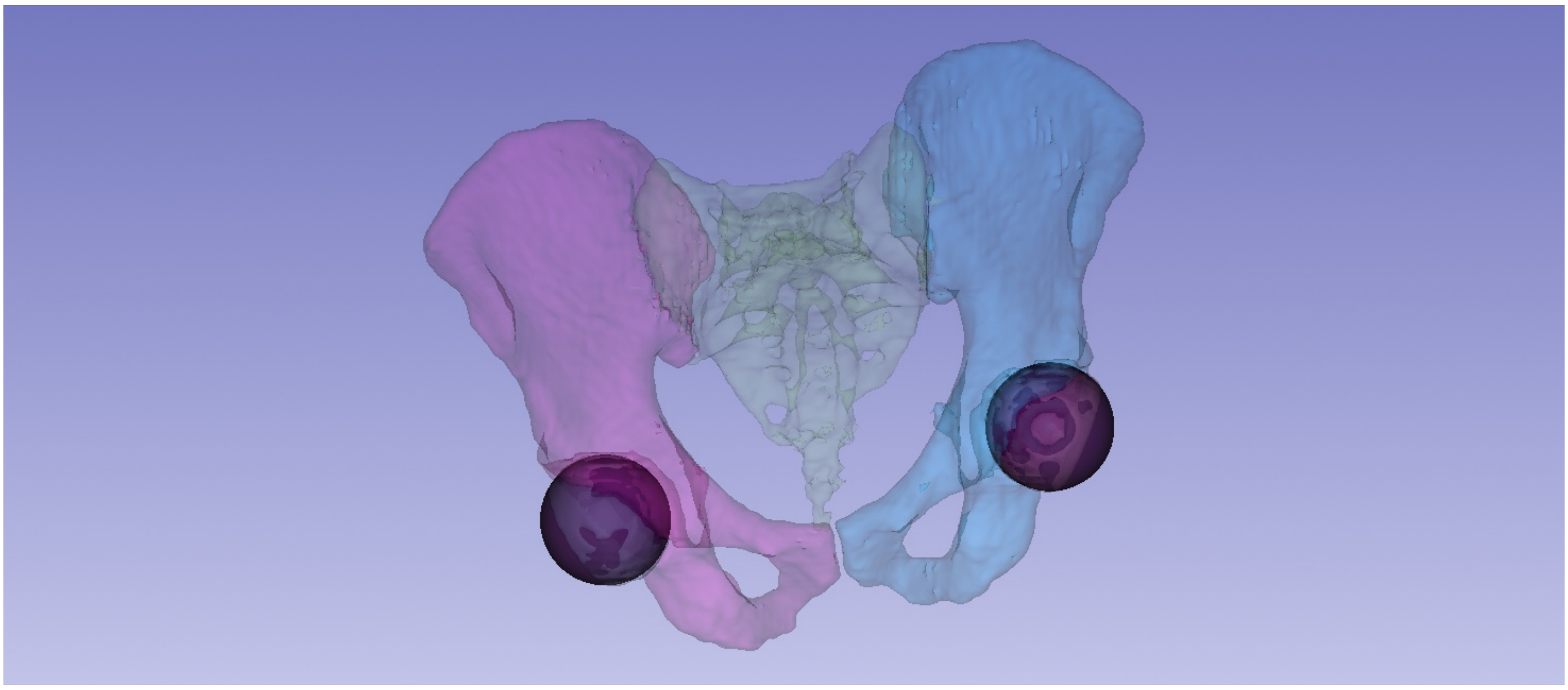

MEASUREMENT	RESULT	REFERENCE / COMPARATOR	INTERPRETATION
Right global coverage	**44.4%**	Asymptomatic CT mean 40 +/- 2%; calculated mean +/-2SD approximately 36-44% [5]	Z=+2.22 | ABOVE APPROXIMATE ASYMPTOMATIC MEAN+2SD — DESCRIPTIVE ONLY
Left global coverage	**44.6%**	Asymptomatic CT mean 40 +/- 2%; calculated mean +/-2SD approximately 36-44% [5]	Z=+2.30 | ABOVE APPROXIMATE ASYMPTOMATIC MEAN+2SD — DESCRIPTIVE ONLY

Global curved-surface coverage is measured on a best-fit femoral-head sphere using radial intersection with the acetabular shell. This is an automated adaptation of Larson's method; 40 +/- 2% is the published comparator [5].

Pelvic CT Segmentation and Quantitative Anatomical Analysis

5. Native Original-CT Acetabular Discontinuity — Decisive Ray Test

CAUTION: Right 1 accepted acetabular discontinuity cluster(s); maximum 3-D span 4.2 mm; small finding may be irrelevant/ artifactual; source-CT confirmation required. CAUTION: Left 1 accepted acetabular discontinuity cluster(s); maximum 3-D span 3.8 mm; small finding may be irrelevant/artifactual; source-CT confirmation required.

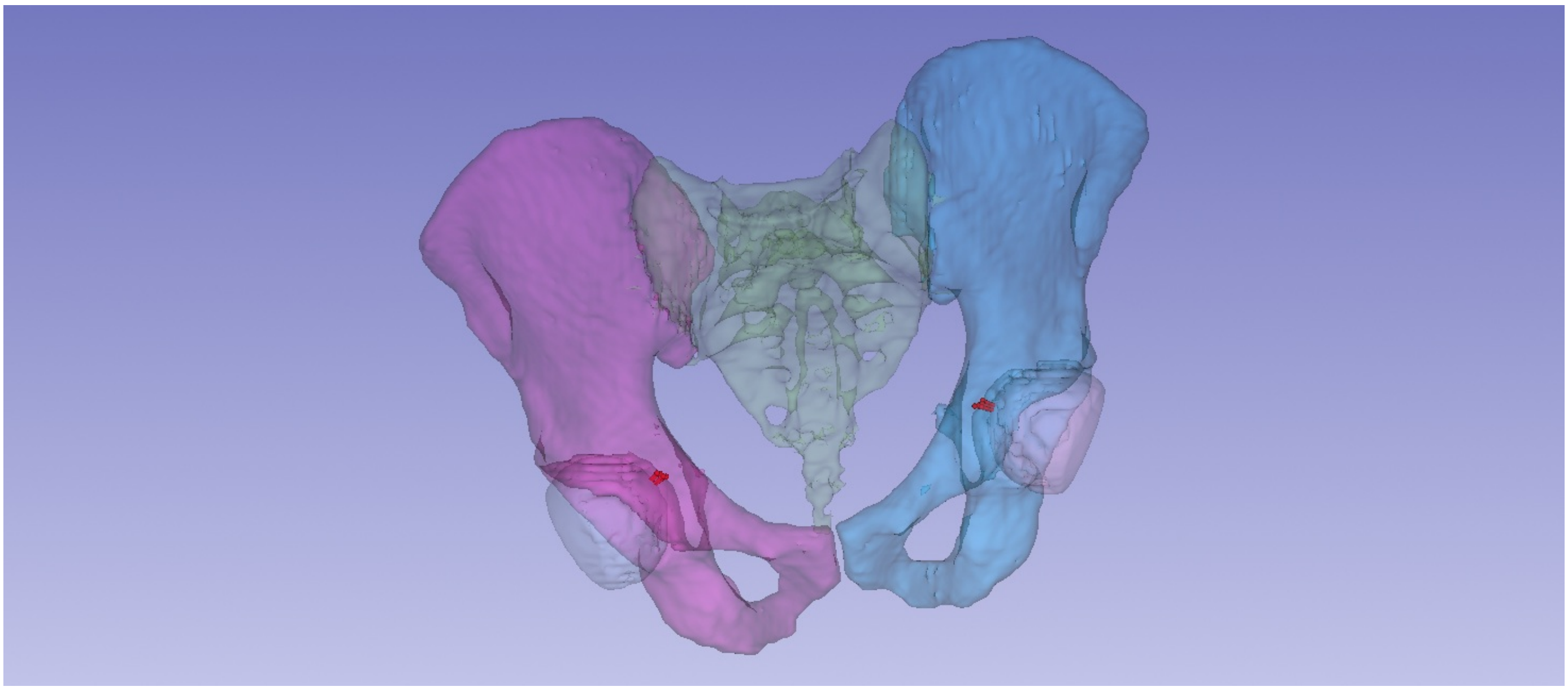

MEASUREMENT	RESULT	REFERENCE / COMPARATOR	INTERPRETATION
Right accepted internal discontinuity clusters	**1**	Original CT support inside the ipsilateral innominate; Check-2 shell supplies expected socket geometry only	INTERNAL ACETABULAR DISCONTINUITY CANDIDATE
Right maximum radial opening	**3.39 mm**	Radial opening is measured along socket-centred rays; it is distinct from 3-D cluster span	Accepted discontinuity geometry
Left accepted internal discontinuity clusters	**1**	Original CT support inside the ipsilateral innominate; Check-2 shell supplies expected socket geometry only	INTERNAL ACETABULAR DISCONTINUITY CANDIDATE
Left maximum radial opening	**5.07 mm**	Radial opening is measured along socket-centred rays; it is distinct from 3-D cluster span	Accepted discontinuity geometry
Accepted cluster total	**2**	Native original-CT ray analysis	NATIVE MILD DEFECT
Maximum radial opening	**5.07 mm**	10-mm route boundary is study-internal and requires cohort validation	NATIVE MILD DEFECT
Complete no-hit cluster	**NO**	A complete no-hit is handled separately from a measurable finite opening	MAXIMUM NATIVE RADIAL OPENING 5.07 MM IS < 10.00 MM.
Final acetabular route	**NATIVE MILD DEFECT**	Native ray + low-only coverage policy + route controller	NATIVE ACETABULAR BOX COMPLETE; NO MIRROR DEFECT RECONSTRUCTION

Decisive native acetabular fracture-evidence step: the Check-2 shell supplies only the expected socket/angular geometry. Actual bone support is determined from the original CT inside the ipsilateral innominate. The peripheral rim band and inferior rays are excluded. Accepted internal clusters are spatially connected CT-supported outliers/no-hit regions with supported borders. Radial opening is measured along socket-centred rays and is distinct from 3-D cluster span. The 10-mm routing boundary is a study-internal threshold awaiting direct cohort validation.

Pelvic CT Segmentation and Quantitative Anatomical Analysis

Pubic Symphysis

PUBIC SYMPHYSIS: REFERENCE-COMPATIBLE WIDTH; GAP 1.6 mm, AP/SI SHEAR 0.3 mm/-0.4 mm.

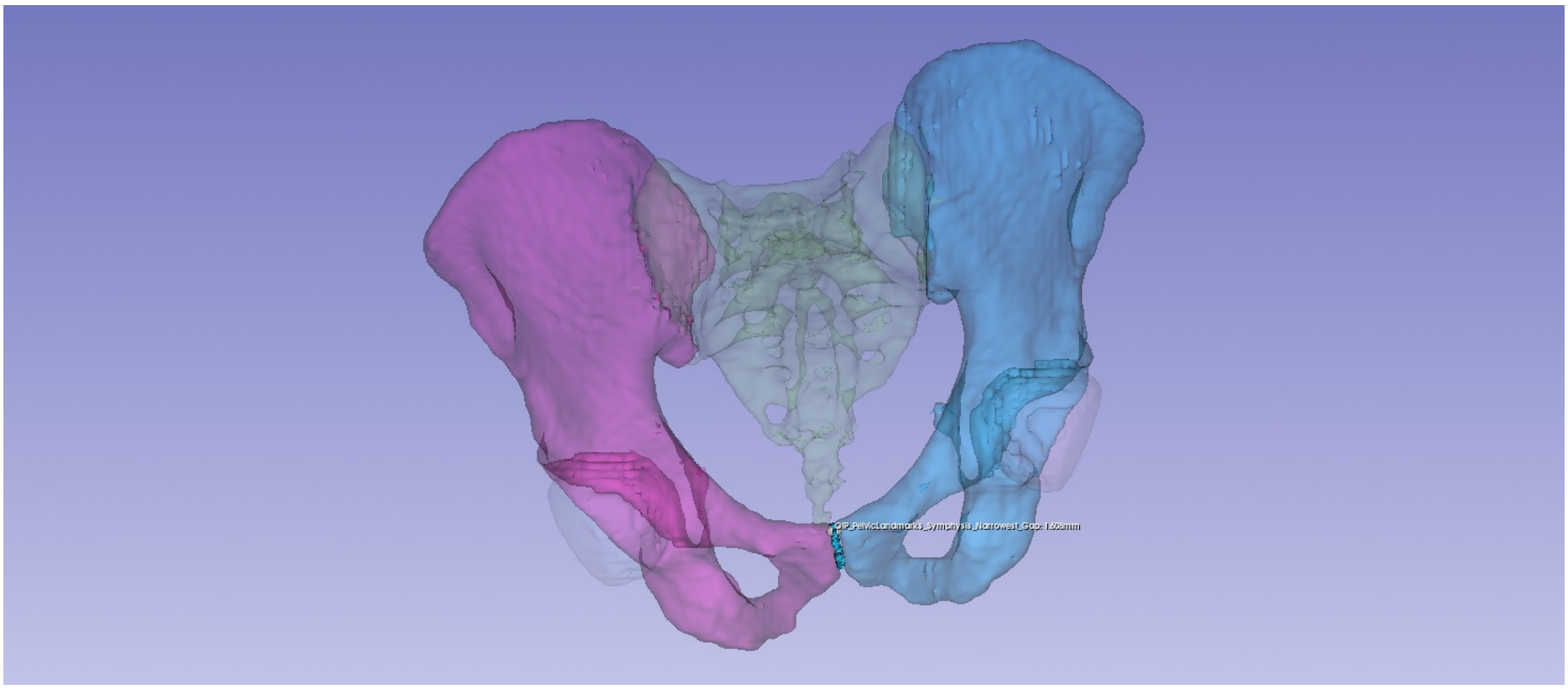

MEASUREMENT	RESULT	REFERENCE / COMPARATOR	INTERPRETATION
Narrowest symphyseal gap	**1.6 mm**	Manual axial-CT mean 3.1 +/- 1.5 mm; calculated mean +/-2SD approximately 0.1-6.1 mm [1]	NO CT EVIDENCE OF SYMPHYSEAL DIASTASIS
AP / SI shear	**0.3 mm / -0.4 mm**	No validated static-CT cutoff for this automated measurement; the published 5-mm value refers to dynamic flamingo radiographs [4]	Descriptive only

Narrowest stable left-right symphyseal gap. Hwang et al. used a manual axial-CT measurement; the calculated 6.1-mm mean+2SD value is descriptive rather than diagnostic [1].

Pelvic CT Segmentation and Quantitative Anatomical Analysis

Obturator Ring

OBTURATOR RING: Right no reportable rim discontinuity; Left no reportable rim discontinuity.

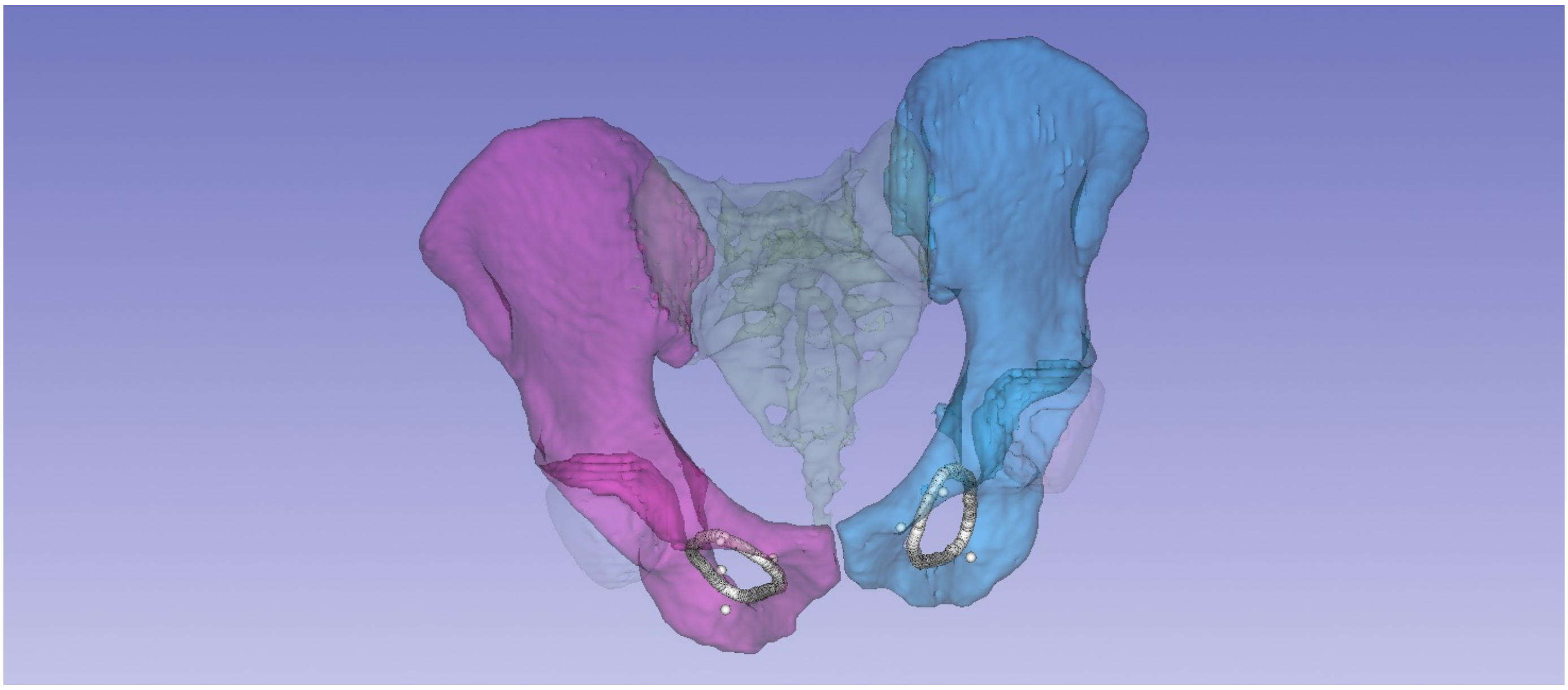

MEASUREMENT	RESULT	REFERENCE / COMPARATOR	INTERPRETATION
Right AP / SI extent	**52.5 mm / 23.6 mm**	Dry-bone mean AP 52.4 +/- 8.0 mm; SI 46.9 +/- 18.3 mm [7]	Descriptive dry-bone comparator only
Right rim discontinuity	**0**	4-mm registered pelvic asymmetry level is an alert comparator, not a fracture cutoff [6]	Visual confirmation required
Left AP / SI extent	**50.1 mm / 36.1 mm**	Dry-bone mean AP 51.7 +/- 8.7 mm; SI 46.6 +/- 16.9 mm [7]	Descriptive dry-bone comparator only
Left rim discontinuity	**0**	4-mm registered pelvic asymmetry level is an alert comparator, not a fracture cutoff [6]	Visual confirmation required

The inner obturator rim is tracked through 360 degrees. The 4-mm literature value is a registered pelvic-asymmetry comparator, not a fracture cutoff; dry-bone AP/SI dimensions are descriptive only [6,7].

Pelvic CT Segmentation and Quantitative Anatomical Analysis

Sacroiliac Joints

SACROILIAC JOINTS ASSESSED: NO LARGE DIASTASIS BY PROVISIONAL CT PROFILE SCREEN; P95 side difference axial 0.3 mm / coronal 0.2 mm; width narrower than age reference.

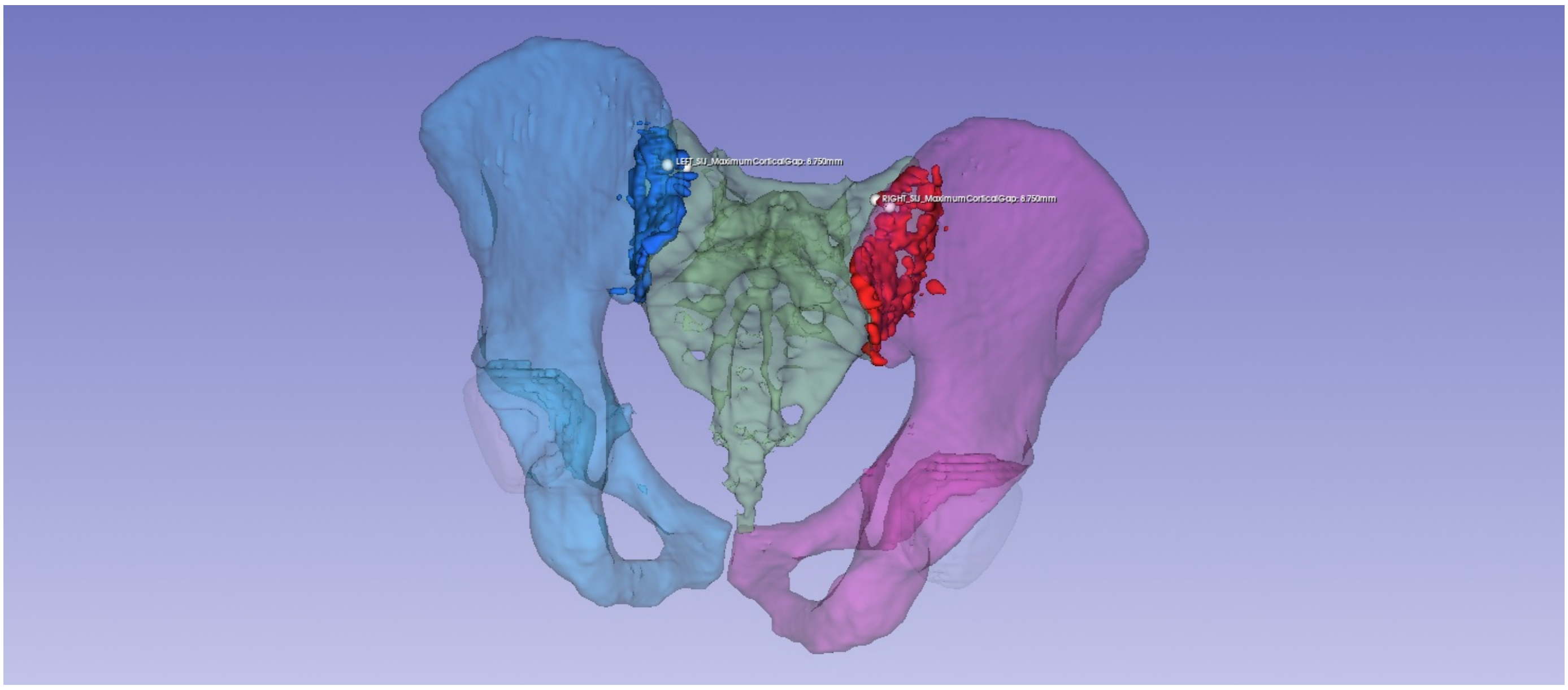

MEASUREMENT	RESULT	REFERENCE / COMPARATOR	INTERPRETATION
Right median cortical gap	**1.0 mm**	Published CT reference: 2.3 +/- 0.4 mm for patients <40 y and 1.9 +/- 0.2 mm for patients >40 y; patient age not supplied [8]	NARROWER THAN AGE REFERENCE
Left median cortical gap	**0.8 mm**	Published CT reference: 2.3 +/- 0.4 mm for patients <40 y and 1.9 +/- 0.2 mm for patients >40 y; patient age not supplied [8]	NARROWER THAN AGE REFERENCE
Axial / coronal side difference	**0.3 mm / 0.2 mm**	Study-specific open-reduction context: 3.39 / 3.71 mm; absolute 6.52 / 7.26 mm [9]	NO LARGE DIASTASIS BY PROVISIONAL CT PROFILE SCREEN

Opposing sacral and iliac cortices localize the SI joint, and original CT is sampled along local normals. Published normal widths and trauma cut-points are comparators because the automated 3-D construction differs from manual MPR measurements [8,9].

Pelvic CT Segmentation and Quantitative Anatomical Analysis

Final Innominate Partition

FINAL INNOMINATE PARTITION: EXACT BILATERAL RECONSTRUCTION. Current method: ROOT10 single-plane pubis/ ischium split.

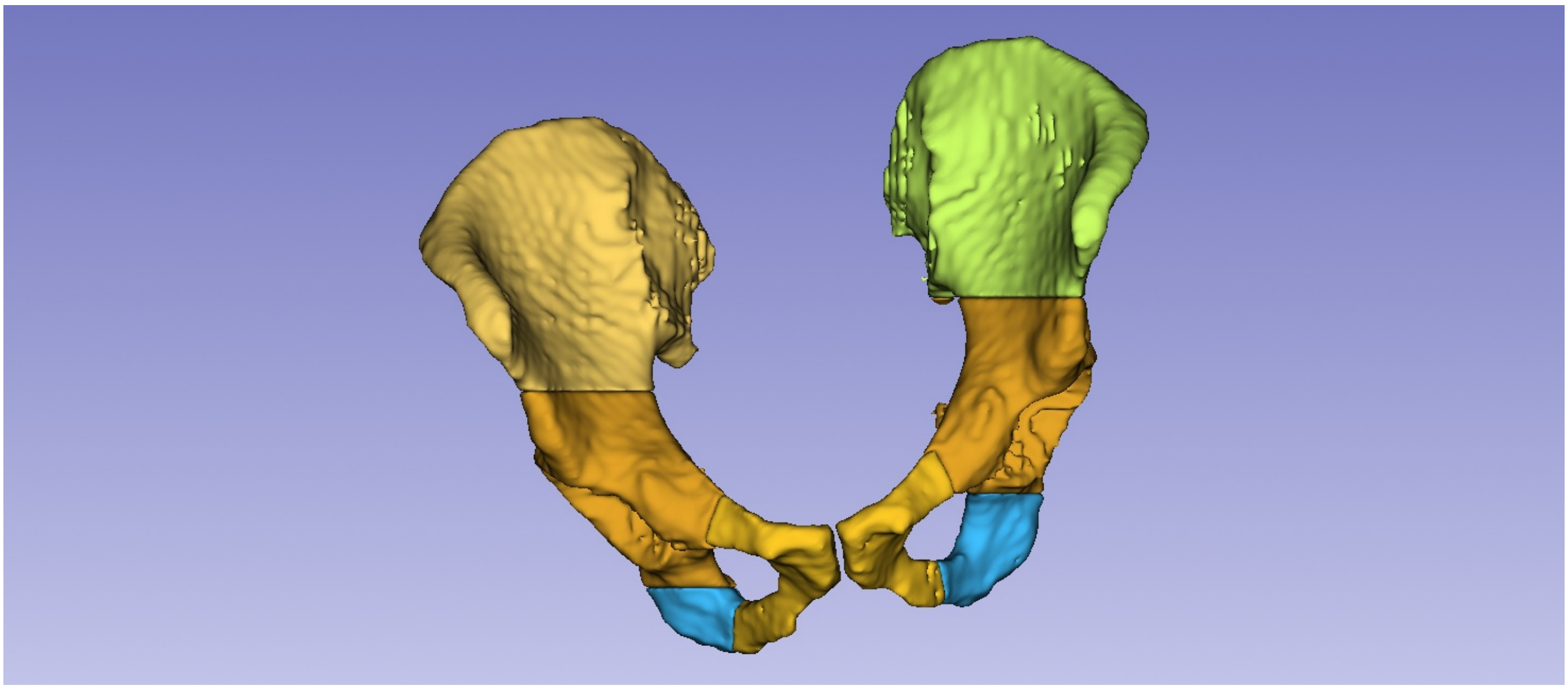

MEASUREMENT	RESULT	REFERENCE / COMPARATOR	INTERPRETATION
Right reconstruction	**PASS**	Technical requirement: zero overlap/unassigned/ outside voxels.	ROOT10 single-plane pubis/ ischium split
Left reconstruction	**PASS**	Technical requirement: zero overlap/unassigned/ outside voxels.	ROOT10 single-plane pubis/ ischium split

The innominate is reconstructed as mutually exclusive acetabulum, ilium, pubis, and ischium. Zero overlap, unassigned, and outside voxels are technical consistency requirements.

Pelvic CT Segmentation and Quantitative Anatomical Analysis

Technical Measurements - Page 1

**ROUTE / QUALITY CONTROL**	
Report status	VALID
Initial pathway	NORMAL
Final state	NATIVE MILD DEFECT
Midsagittal-plane QC	PASS — BOTH CENTRAL BONES ARE NEAR-EQUAL
Route notes	Registered mirror-based abnormal reconstruction outputs are not applicable to this route.
**1. FEMORAL-HEAD MORPHOLOGY AND CENTRE - TECHNICAL MEASUREMENTS**	
**RIGHT**	
Centre confidence	HIGH
Fitted radius	25.21 mm
Sphere RMS	0.79 mm
Normalized RMS	3.15%
P95 residual	1.59 mm
Meaningful components	1
**LEFT**	
Centre confidence	HIGH
Fitted radius	24.79 mm
Sphere RMS	0.64 mm
Normalized RMS	2.58%
P95 residual	1.42 mm
Meaningful components	1
**BILATERAL QC**	
Radius difference	0.42 mm
Radius difference	1.70%
Bilateral radius QC	PASS
**2. NATIVE HEAD-SOCKET SHELL AND CONGRUITY - TECHNICAL MEASUREMENTS**	
**RIGHT**	
Margin status	CONTINUOUS
Structure class	INTACT CONTACT MARGIN
Local congruity	CONGRUENT
Full-head coverage	44.33%
Superior-head coverage	63.30%
Shell area	5174.86 mm^2
Meaningful components	1
Median head-to-shell gap	2.06 mm
P95 head-to-shell gap	5.08 mm
**LEFT**	
Margin status	CONTINUOUS
Structure class	INTACT CONTACT MARGIN
Local congruity	CONGRUENT
Full-head coverage	44.33%

Pelvic CT Segmentation and Quantitative Anatomical Analysis

Technical Measurements - Page 2

Superior-head coverage	64.18%
Shell area	5250.54 mm²
Meaningful components	1
Median head-to-shell gap	1.98 mm
P95 head-to-shell gap	6.00 mm
**3. MIDSAGITTAL PLANE AND CENTRAL-BONE QC - TECHNICAL MEASUREMENTS**	
Overall plane QC	PASS — BOTH CENTRAL BONES ARE NEAR-EQUAL
**L5**	
RIGHT volume	33549.46 mm3
LEFT volume	33928.54 mm3
Bilateral difference	1.12%
Smaller/larger ratio	0.9888
Symmetry QC	NEAR-EQUAL
**Sacrum**	
RIGHT volume	111484.15 mm3
LEFT volume	112586.61 mm3
Bilateral difference	0.98%
Smaller/larger ratio	0.9902
Symmetry QC	NEAR-EQUAL
**5. NATIVE ORIGINAL-CT ACETABULAR DISCONTINUITY — DECISIVE RAY TEST - TECHNICAL MEASUREMENTS**	
Accepted cluster total	2
Maximum radial opening	5.07 mm
Complete no-hit cluster	False
Radial opening routing gate	10.00 mm
Ray route	NATIVE MILD DEFECT
Routing basis	Maximum native radial opening 5.07 mm is < 10.00 mm.
**RIGHT**	
Internal rays tested	2028
CT-supported internal rays	2027
Raw internal candidates	5
Accepted defect rays	4
Accepted defect clusters	1
Maximum side radial opening	3.39 mm
Complete no-hit cluster	False
Defect status	INTERNAL ACETABULAR DISCONTINUITY CANDIDATE
Cluster 1 location	ANTERIOR-SUPERIOR
Cluster 1 rays	4
Cluster 1 3-D span	4.15 mm
Cluster 1 radial opening	3.39 mm
Cluster 1 class	MILD WITH PARTIAL NO-HIT GAP
**LEFT**	
Internal rays tested	2043

Pelvic CT Segmentation and Quantitative Anatomical Analysis

Technical Measurements - Page 3

CT-supported internal rays	2040
Raw internal candidates	5
Accepted defect rays	5
Accepted defect clusters	1
Maximum side radial opening	5.07 mm
Complete no-hit cluster	False
Defect status	INTERNAL ACETABULAR DISCONTINUITY CANDIDATE
Cluster 1 location	ANTERIOR-SUPERIOR
Cluster 1 rays	5
Cluster 1 3-D span	3.78 mm
Cluster 1 radial opening	5.07 mm
Cluster 1 class	MODERATE WITH PARTIAL NO-HIT GAP
**PUBIC SYMPHYSIS - TECHNICAL MEASUREMENTS**	
Clinical status	NORMAL
Screening classification	NO CT EVIDENCE OF SYMPHYSEAL DIASTASIS
Stable narrowest gap	1.63 mm
Selected pair gap	1.51 mm
Three-dimensional point distance	1.61 mm
AP shear	0.31 mm
SI shear	-0.44 mm
Supporting local cells	9
Local gap IQR	0.02 mm
**OBTURATOR RING - TECHNICAL MEASUREMENTS**	
**RIGHT**	
AP extent	52.45 mm
SI extent	23.61 mm
Area	1502.65 mm^2
Perimeter	156.80 mm
Tracked rim	100.00%
Tracked rim outside 4-mm band	70.83%
Suspected discontinuities	0
**LEFT**	
AP extent	50.14 mm
SI extent	36.12 mm
Area	1536.46 mm^2
Perimeter	158.35 mm
Tracked rim	100.00%
Tracked rim outside 4-mm band	67.78%
Suspected discontinuities	0
**SACROILIAC JOINTS - TECHNICAL MEASUREMENTS**	
Width classification	NARROWER THAN AGE REFERENCE
Median cortical width	1.00 mm

Pelvic CT Segmentation and Quantitative Anatomical Analysis

Technical Measurements - Page 4

P95 cortical width	5.50 mm
Maximum cortical width	8.75 mm
P95 axial projection	5.21 mm
P95 coronal projection	4.59 mm
CT-validated rays	1143
Validation fraction	0.998
Bilateral trauma screen	NO LARGE DIASTASIS BY PROVISIONAL CT PROFILE SCREEN
Width classification	NARROWER THAN AGE REFERENCE
Median cortical width	0.75 mm
P95 cortical width	5.00 mm
Maximum cortical width	8.75 mm
P95 axial projection	4.86 mm
P95 coronal projection	4.38 mm
CT-validated rays	1026
Validation fraction	1.000
Bilateral trauma screen	NO LARGE DIASTASIS BY PROVISIONAL CT PROFILE SCREEN
**FINAL INNOMINATE PARTITION - TECHNICAL MEASUREMENTS**	
**RIGHT**	
Exact reconstruction	True
Unassigned voxels	0
Outside voxels	0
Overlap voxels	0
Acetabulum voxels	214422
Ilium voxels	294305
Pubis voxels	38370
Ischium voxels	23296
**LEFT**	
Exact reconstruction	True
Unassigned voxels	0
Outside voxels	0
Overlap voxels	0
Acetabulum voxels	204693
Ilium voxels	266187
Pubis voxels	33042
Ischium voxels	41724
**CURRENT PARTITION METHOD**	
Method	ROOT10 single-plane pubis/ischium split
Split mode	BOX ANCHOR FINITE20 ROOT CORRIDOR ILIUM FLOOR
Obturator used for split	False
Mirroring used for split	False
8-mm reclamation	False

Pelvic CT Segmentation and Quantitative Anatomical Analysis

References

Published comparators are interpreted according to their original measurement definitions; where the automated measurement differs, comparison is descriptive rather than a diagnostic cutoff.

[1] Hwang K, Wu X, Park CY. Width of pubic symphysis relating to age and sex in Koreans. J Orthop Surg Res. 2021;16(1):430. doi:10.1186/s13018-021-02561-9. PMID:34217332.

[2] Doro CJ, Forward DP, Kim H, Nascone JW, Sciadini MF, Hsieh AH, et al. Does 2.5 cm of symphyseal widening differentiate anteroposterior compression I from anteroposterior compression II pelvic ring injuries? J Orthop Trauma. 2010;24(10):610-615. doi:10.1097/BOT.0b013e3181cff42c. PMID:20871248.

[3] Gansslen A, Lindahl J, Krappinger D, Lindtner RA, Staresinic M. The myth of 2.5 cm symphyseal diastasis. Arch Orthop Trauma Surg. 2025;145(1):306. doi:10.1007/s00402-025-05904-x. PMID:40397156.

[4] Garras DN, Carothers JT, Olson SA. Single-leg-stance (flamingo) radiographs to assess pelvic instability: how much motion is normal? J Bone Joint Surg Am. 2008;90(10):2114-2118. doi:10.2106/JBJS.G.00277. PMID:18829908.

[5] Larson CM, Moreau-Gaudry A, Kelly BT, Byrd JWT, Tonetti J, Lavallee S, et al. Are normal hips being labeled as pathologic? A CT-based method for defining normal acetabular coverage. Clin Orthop Relat Res. 2015;473(4):1247-1254. doi:10.1007/s11999-014-4055-2. PMID:25407391.

[6] Zhang F, Zhang D, Huang Z, Wang Z, Cai X. Morphological asymmetry of pelvic rings: a study based on three-dimensional deviation analysis. Orthop Surg. 2022;14(5):967-976. doi:10.1111/os.13246. PMID:35377554.

[7] Duygu OB, Govsa F. Anatomical structures of the obturator foramen and its importance for pelvic interventions. Surg Radiol Anat. 2025;47(1):163. doi:10.1007/s00276-025-03661-5. PMID:40544414.

[8] Faflia CP, Prassopoulos PK, Daskalogiannaki ME, Gourtsoyiannis NC. Variation in the appearance of the normal sacroiliac joint on pelvic CT. Clin Radiol. 1998;53(10):742-746. doi:10.1016/S0009-9260(98)80316-4. PMID:9817091.

[9] Chien RS, Chen IJ, Lai CY, Chen JP, Yu YH. Critical distance of the sacroiliac joint for open reduction using screw fixation for traumatic sacroiliac joint diastasis: a retrospective study. J Orthop Surg Res. 2024;19(1):268. doi:10.1186/s13018-024-04759-z. PMID:38678298.

[10] Jamali AA, Mak W, Wang P, Tai L, Meehan JP, Lamba R. What is normal femoral head/neck anatomy? An analysis of radial CT reconstructions in adolescents. Clin Orthop Relat Res. 2013;471(11):3581-3587. doi:10.1007/s11999-013-3166-5. PMID:23922189.

[11] Hassan MM, Feroe AG, Douglass BW, Jimenez AE, Kuhns B, Mitchell CF, et al. Three-dimensional analysis of age and sex differences in femoral head asphericity in asymptomatic hips in the United States. World J Orthop. 2024;15(8):754-763. doi:10.5312/wjo.v15.i8.754. PMID:39165879.

[12] Rivas DJL, Gassmann JM, Goetz JE, Aitken HD, Davison JC, Miller A, et al. Weight-bearing CT as an approach to assess femoral-acetabular displacement during external rotation stress in the hip. J Hip Preserv Surg. 2025;12(2):93-104. doi:10.1093/jhps/hnaf001. PMID:40761572.

[13] Judet R, Judet J, Letournel E. Fractures of the acetabulum: classification and surgical approaches for open reduction. Preliminary report. J Bone Joint Surg Am. 1964;46:1615-1646. doi:10.2106/00004623-196446080-00001. PMID:14239854.

[14] Imani M, Buratto J, Dao T, Meijering E, Vogrin S, Kwok TCY, et al. Deep learning technique for automatic segmentation of proximal hip musculoskeletal tissues from CT scan images: a MrOS study. J Cachexia Sarcopenia Muscle. 2025;16(2):e13728. doi:10.1002/jcsm.13728. PMID:40022453.

[15] Ead MS, Duke KK, Jaremko JL, Westover L. Investigation of pelvic symmetry using CAD software. Med Biol Eng Comput. 2020;58(1):75-82. doi:10.1007/s11517-019-02068-w. PMID:31745838.

[16] Amar E, Druckmann I, Flusser G, Safran MR, Salai M, Rath E. The anterior inferior iliac spine: size, position, and location. An anthropometric and sex survey. Arthroscopy. 2013;29(5):874-881. doi:10.1016/j.arthro.2013.01.023. PMID:23523127.

[17] Rana SH, Farjoodi P, Haloman S, Dutton P, Hariri A, Ward SR, et al. Anatomic evaluation of the sacroiliac joint: a radiographic study with implications for procedures. Pain Physician. 2015;18(6):583-592. PMID:26606010.

[18] Hartel MJ, Petersik A, Schmidt A, Kendoff D, Nuchtern J, Rueger JM, et al. Determination of femoral neck angle and torsion angle utilizing a novel three-dimensional modeling and analytical technology based on CT datasets. PLoS One. 2016;11(3):e0149480. doi:10.1371/journal.pone.0149480.

[19] Mahmood SS, Mukka SS, Crnalic S, Wretenberg P, Sayed-Noor AS. Association between changes in global femoral offset after total hip arthroplasty and function, quality of life, and abductor muscle strength. Acta Orthop. 2016;87(1):36-41. doi:10.3109/17453674.2015.1091955.

[20] O'Brien S, Kernohan G. Perception of imposed leg length inequality in normal subjects. Hip Int. 2010;20(4):505-511. doi:10.1177/112070001002000414.

[21] Wang G, et al. Measurement of relative position of femoral head center, greater trochanter, and lesser trochanter. Ann Palliat Med. 2021;10(11):11524-11528. doi:10.21037/apm-21-2538.

Pelvic CT Segmentation and Quantitative Anatomical Analysis

Clinical Findings Summary

Case ID: PRFX_0006_0000 | Age: Not supplied | Status: VALID

CONCLUSIVE FINDING

Automated CT analysis flags joint-space narrowing on Left; femoral parameter assessment flagged; symphyseal widening/shear; and 1 additional flagged finding(s). Femoral parameter assessment is flagged; see the dedicated evidence page.

OVERALL STATION ASSESSMENT

Flagged finding of at least 5 mm in the hip/acetabular, anterior-ring station group(s).

Clinical station checklist

HIP JOINT AND ACETABULUM

Femoral-head morphology — NO FLAG

R VALID HEAD CENTRE — NORMAL-LIKE MORPHOLOGY / L VALID HEAD CENTRE — NORMAL-LIKE MORPHOLOGY

Head-socket congruity — NO FLAG

R CONGRUENT / L CONGRUENT

Acetabular coverage — NO FLAG

R 50.1% / L 44.0%; no low-coverage flag

Acetabular continuity — NO FLAG

0 accepted discontinuity clusters; native original CT

Head position / dislocation — NO FLAG

No displacement pathway activated; bilateral native assessment

Joint-space / arthrosis screen — FLAGGED

R WIDENED — SUBLUXATION CANDIDATE (3.50 / 2.50 / 8.00 mm) / L MIXED REGIONAL NARROWING AND WIDENING (3.00 / 0.00 / 9.50 mm)

Femur parameters — FLAGGED

Automated femoral analysis flags left 3D NSA below Hartel true-3D CT IQR; bilateral femoral-offset asymmetry ≥5 mm; right head-centre-to-lesser L/D above the published interval; left head-centre-to-lesser

ANTERIOR PELVIC RING

Overall anterior ring screen — FLAGGED

Discontinuity finding of at least 5 mm at an anterior-ring station

Pubic symphysis — FLAGGED

Gap 7.1 mm; BORDERLINE WIDENING DIASTASIS NOT CONFIRMED

Right obturator ring — NO FLAG

0 reportable rim discontinuity candidates

Left obturator ring — FLAGGED

2 candidate(s); maximum bridge chord 40.0 mm; 5 mm or larger discontinuity

POSTERIOR PELVIC RING

Right SIJ integrity — NO FLAG

median 3.8 mm; P95 5.8 mm; WIDER THAN AGE REFERENCE

Left SIJ integrity — NO FLAG

median 5.0 mm; P95 8.0 mm; WIDER THAN AGE REFERENCE

SCOPE: Joint-space narrowing is an arthrosis/coxarthrosis screen, not a stand-alone diagnosis. These station tests do not exclude isolated or minimally displaced iliac or sacral fractures; review the source CT.

SIJ references: normal CT mean 2.3 +/- 0.4 mm (<40 y) and 1.9 +/- 0.2 mm (>40 y) [8]; traumatic axial/coronal distance context [9]; anatomical extent/search-corridor context [17].

Detailed anatomical evidence and quantitative measurements follow on the next pages.

Pelvic CT Segmentation and Quantitative Anatomical Analysis

1. Femoral-Head Morphology and Centre

RIGHT FEMORAL HEAD: VALID HEAD CENTRE — NORMAL-LIKE MORPHOLOGY; CENTRE HIGH CONFIDENCE. LEFT FEMORAL HEAD: VALID HEAD CENTRE — NORMAL-LIKE MORPHOLOGY; CENTRE HIGH CONFIDENCE.

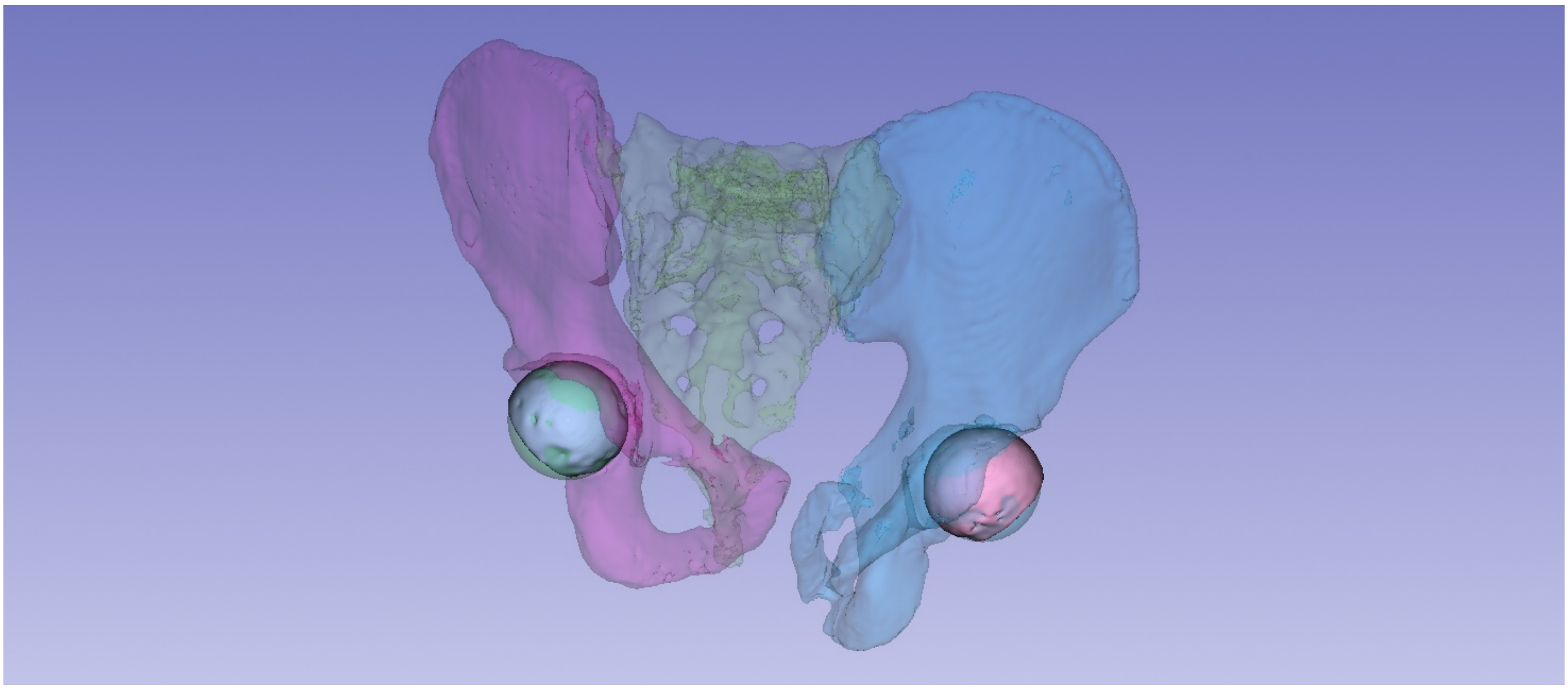

MEASUREMENT	RESULT	REFERENCE / COMPARATOR	INTERPRETATION
Right fitted radius	**23.4 mm**	Adolescent radial-CT comparator: male 24.53 +/- 1.74 mm; female 21.94 +/- 1.13 mm [10]	VALID HEAD CENTRE — NORMAL-LIKE MORPHOLOGY
Left fitted radius	**22.8 mm**	Adolescent radial-CT comparator: male 24.53 +/- 1.74 mm; female 21.94 +/- 1.13 mm [10]	VALID HEAD CENTRE — NORMAL-LIKE MORPHOLOGY
Sphere-fit residual	**R/L P95 1.1 mm/1.6 mm**	Published 3-D sphere/asphericity analysis supports the measurement family; these residual limits are study-specific [11]	Quantitative morphology QC

Best-fit sphere radius and residuals are reported from the segmented femoral head. Published 3-D CT/asphericity work supports this measurement family, while the residual QC limits are study-specific [10,11].

Pelvic CT Segmentation and Quantitative Anatomical Analysis

Femur Subsegmentation and Quantitative Parameters

Automated femoral analysis flags left 3D NSA below Hartel true-3D CT IQR; bilateral femoral-offset asymmetry ≥5 mm; right head-centre-to-lesser L/D above the published interval; left head-centre-to-lesser L/D above the published interval.

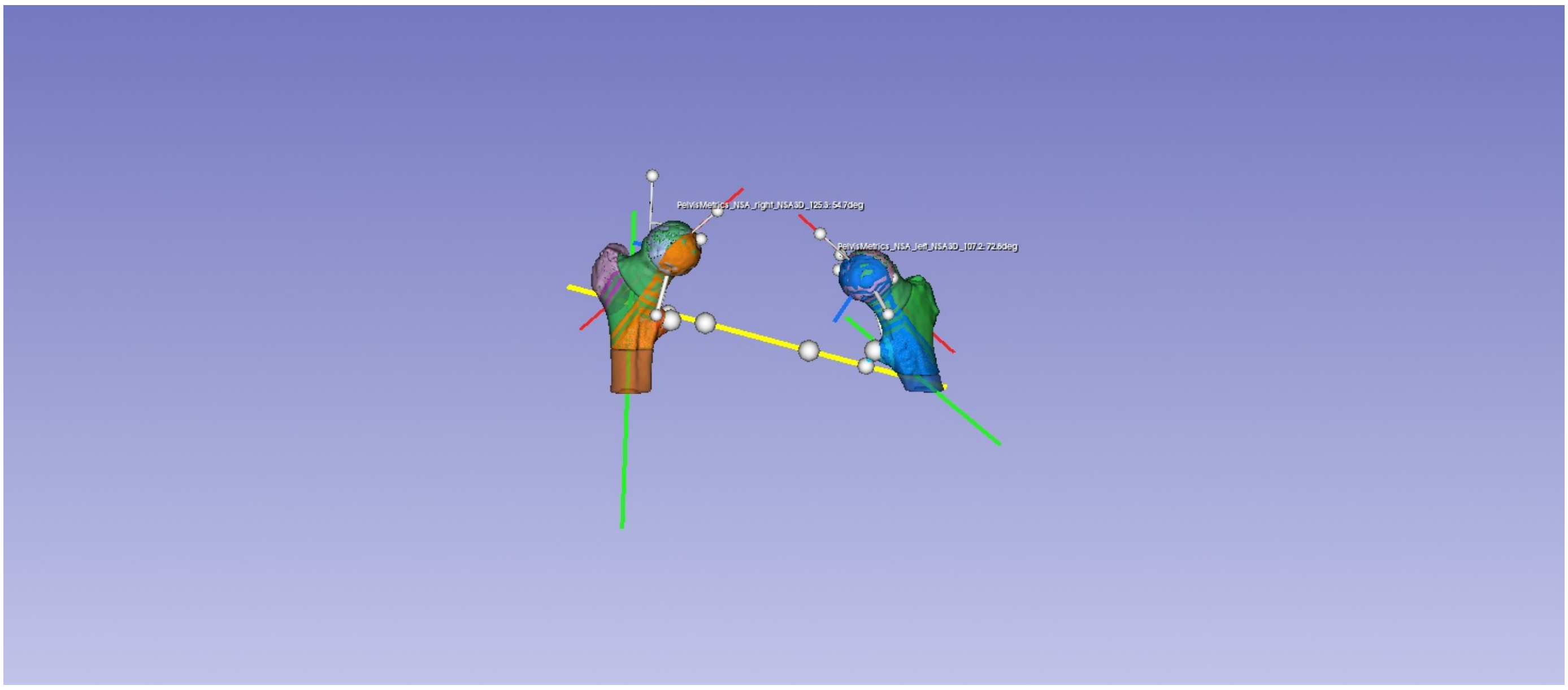

MEASUREMENT	RESULT	REFERENCE / COMPARATOR	INTERPRETATION
True-3D neck-shaft angle (R / L)	**125.35 / 107.23 deg**	Hartel CT IQR 117.9-125.6 deg [18]	WITHIN REFERENCE IQR / FLAGGED LOW
NSA absolute bilateral difference	**18.1 deg**	Quantitative side comparison; not a stand-alone diagnosis [18]	FLAGGED
Femoral offset (R / L)	**34.45 / 47.29 mm**	Absolute native CT values are quantitative only [19]	FLAGGED ASYMMETRY
Offset absolute side difference	**12.8 mm**	>=5 mm reconstruction-asymmetry comparator [19]	FLAGGED
Limb-length discrepancy	**7.8 mm**	>=10 mm comparator [20]	WITHIN 10MM COMPARATOR; Left shorter
Head-centre-to-lesser L/D — Right	**1.267**	AP-radiographic mean 0.84 +/- 0.08; screening interval 0.68-1.00 [21]	L/D is above the published screening interval; review the lesser point and head geometry.
Head-centre-to-lesser L/D — Left	**1.027**	AP-radiographic mean 0.84 +/- 0.08; screening interval 0.68-1.00 [21]	L/D is above the published screening interval; review the lesser point and head geometry.
Integrated femur assessment	**FLAGGED**	Automated quantitative screening; CT/source-segmentation confirmation required	Automated femoral analysis flags left 3D NSA below Hartel true-3D CT IQR; bilateral femoral-offset asymmetry ≥5 mm; right head-centre-to-lesser L/D above the published interval; left head-centre-to-lesser L/D above the published interval.

The displayed femur subsegments preserve the locked RAS geometry and existing lesser-trochanter result. NSA and offset use the corrected medullary-centre shaft axis; LLD uses bilateral lower-innominate reference geometry. L/D uses the femur-local AP projection (posterior component excluded). Published comparators are screening references, not stand-alone diagnoses [18-21].

Pelvic CT Segmentation and Quantitative Anatomical Analysis

2. Native Head-Socket Shell and Congruity

RIGHT MARGIN CONTINUOUS; INTACT CONTACT MARGIN; CONGRUENT. LEFT MARGIN CONTINUOUS; INTACT CONTACT MARGIN; CONGRUENT.

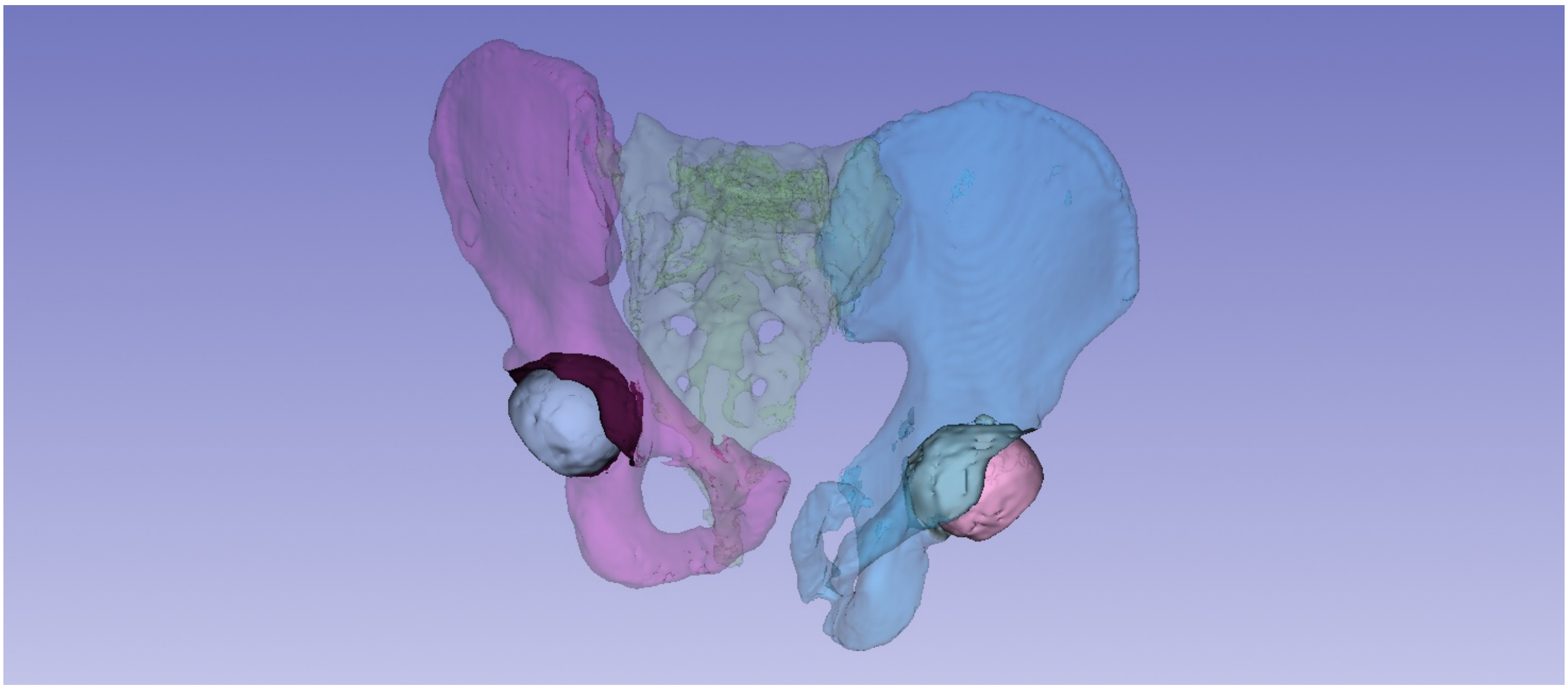

MEASUREMENT	RESULT	REFERENCE / COMPARATOR	INTERPRETATION
Right local gap (median / P95)	**3.0 mm / 5.5 mm**	Healthy-control whole-joint WBCT JSW 3.99 +/- 0.53 mm; different acquisition and geometry [12]	CONGRUENT
Left local gap (median / P95)	**2.8 mm / 6.2 mm**	Healthy-control whole-joint WBCT JSW 3.99 +/- 0.53 mm; different acquisition and geometry [12]	CONGRUENT

Head-centred rays assess acetabular-shell continuity and local gap. The cited weight-bearing CT joint-space value is shown only as context because the acquisition and geometry differ [12].

Pelvic CT Segmentation and Quantitative Anatomical Analysis

3. Midsagittal Plane and Central-Bone QC

MIDSAGITTAL PLANE QC: PASS — BOTH CENTRAL BONES ARE NEAR-EQUAL; L5 8.74%; SACRUM 9.35%.

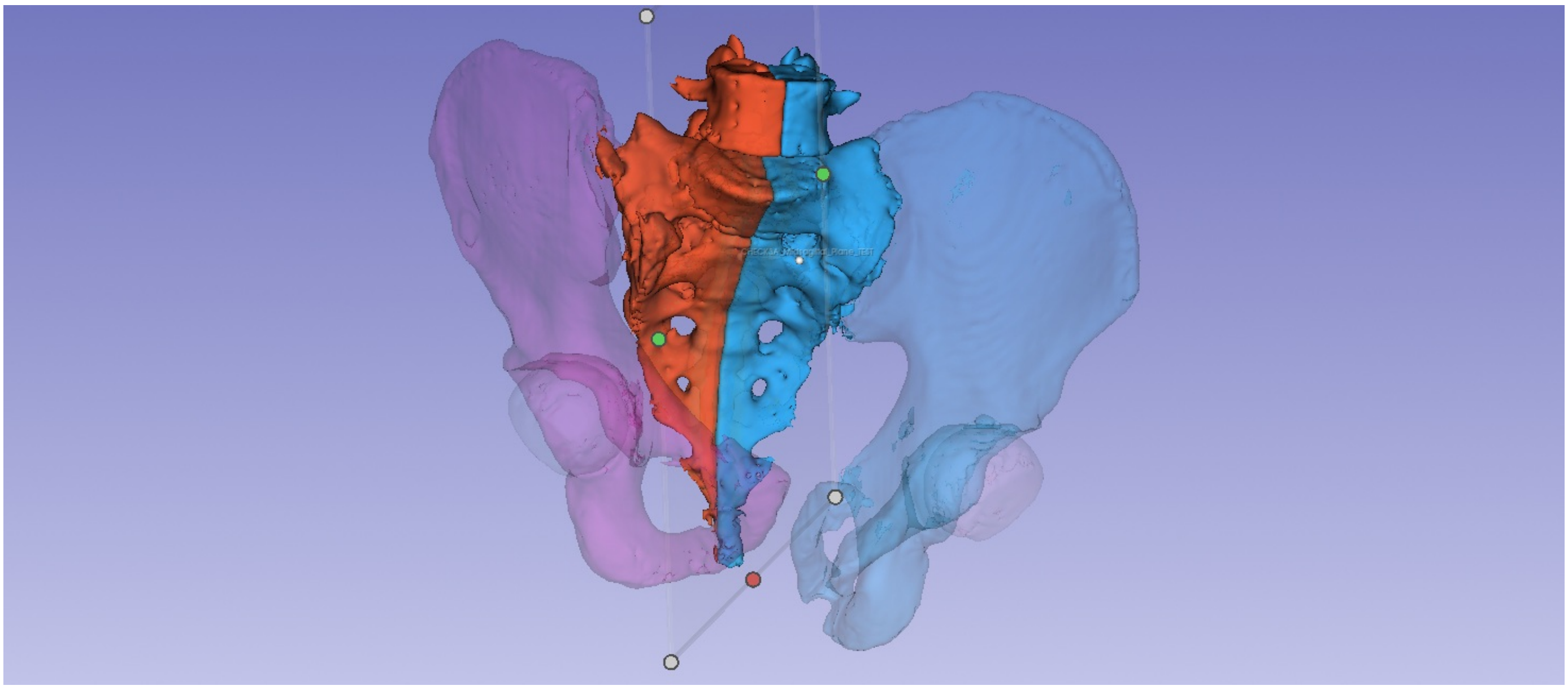

MEASUREMENT	RESULT	REFERENCE / COMPARATOR	INTERPRETATION
L5 bilateral half-volume difference	**8.74%**	Study-internal geometric QC	NEAR-EQUAL
Sacral bilateral half-volume difference	**9.35%**	Study-internal geometric QC	NEAR-EQUAL
Overall plane QC	**PASS — BOTH CENTRAL BONES ARE NEAR-EQUAL**	10% bilateral difference is a study-internal QC rule	Technical plane validation; not a clinical diagnostic cutoff

The pelvic midsagittal plane is accepted only after bilateral L5/sacral half-volume QC. The 10% difference rule is a study-internal geometric QC criterion, not a clinical threshold.

Pelvic CT Segmentation and Quantitative Anatomical Analysis

4. Global Femoral-Head Coverage

GLOBAL FEMORAL-HEAD COVERAGE: RIGHT 50.1%; LEFT 44.0%.

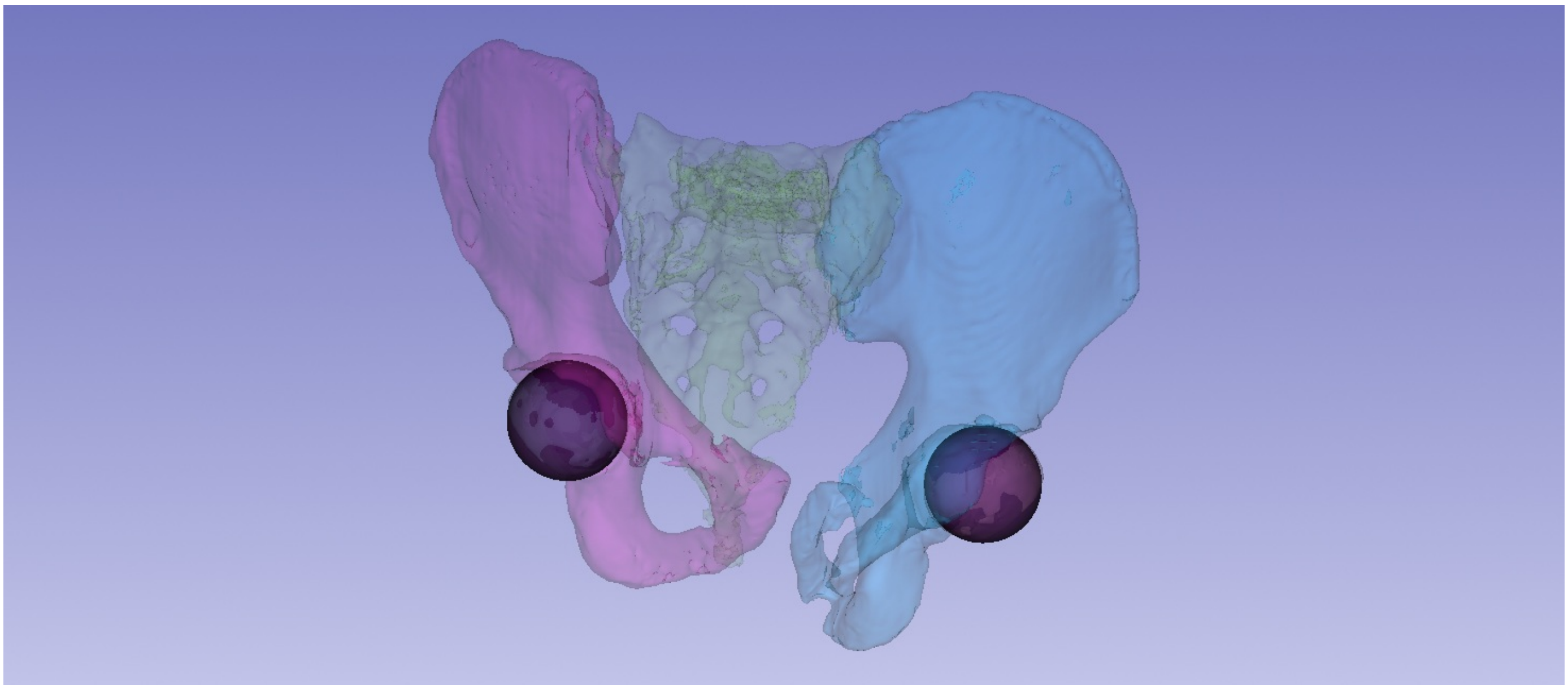

MEASUREMENT	RESULT	REFERENCE / COMPARATOR	INTERPRETATION
Right global coverage	**50.1%**	Asymptomatic CT mean 40 +/- 2%; calculated mean +/-2SD approximately 36-44% [5]	Z=+5.06 | ABOVE APPROXIMATE ASYMPTOMATIC MEAN+2SD — DESCRIPTIVE ONLY
Left global coverage	**44.0%**	Asymptomatic CT mean 40 +/- 2%; calculated mean +/-2SD approximately 36-44% [5]	Z=+2.01 | ABOVE APPROXIMATE ASYMPTOMATIC MEAN+2SD — DESCRIPTIVE ONLY

Global curved-surface coverage is measured on a best-fit femoral-head sphere using radial intersection with the acetabular shell. This is an automated adaptation of Larson's method; 40 +/- 2% is the published comparator [5].

Pelvic CT Segmentation and Quantitative Anatomical Analysis

5. Native Original-CT Acetabular Discontinuity — Decisive Ray Test

No internal acetabular discontinuity detected on native original-CT ray analysis.

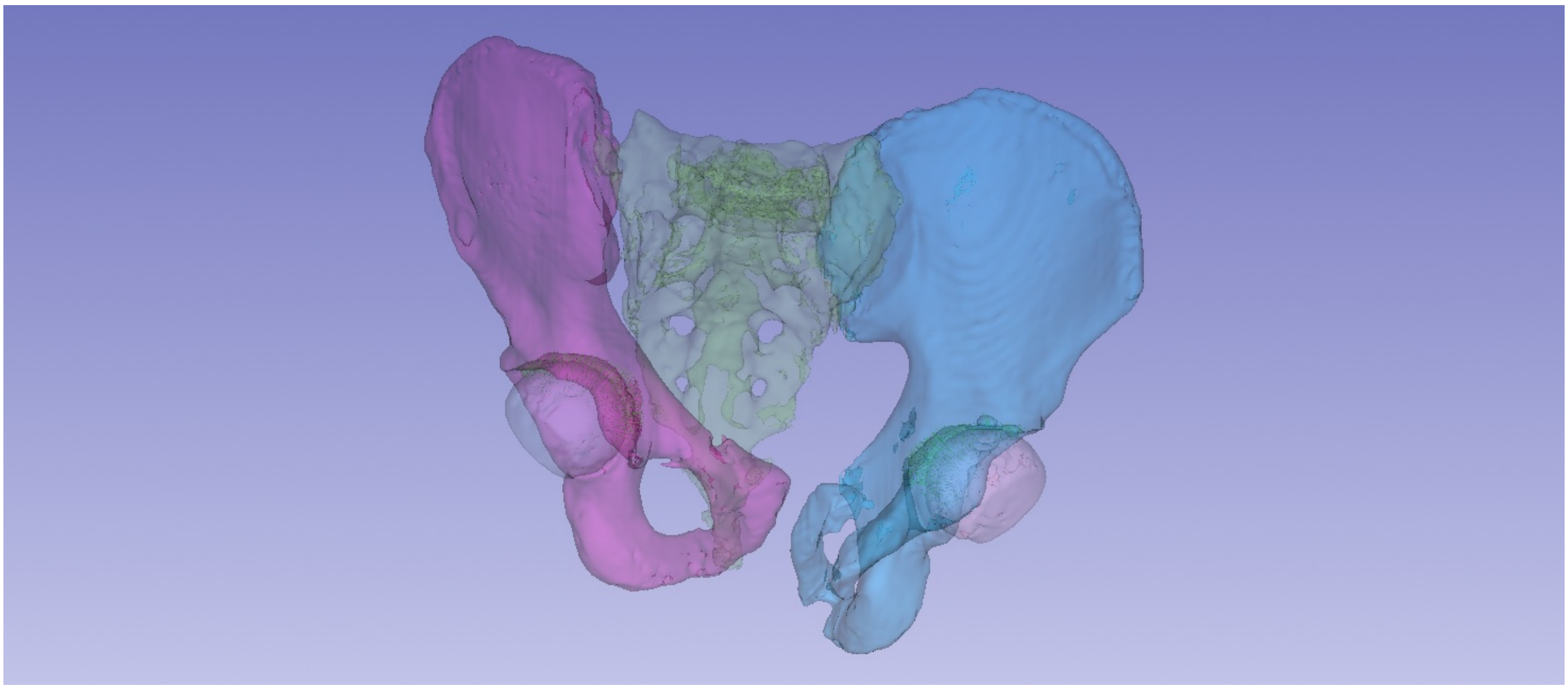

MEASUREMENT	RESULT	REFERENCE / COMPARATOR	INTERPRETATION
Right accepted internal discontinuity clusters	**0**	Original CT support inside the ipsilateral innominate; Check-2 shell supplies expected socket geometry only	NO INTERNAL ACETABULAR DISCONTINUITY DETECTED
Left accepted internal discontinuity clusters	**0**	Original CT support inside the ipsilateral innominate; Check-2 shell supplies expected socket geometry only	NO INTERNAL ACETABULAR DISCONTINUITY DETECTED
Accepted cluster total	**0**	Native original-CT ray analysis	NO ACCEPTED CLUSTER
Maximum radial opening	**NA**	10-mm route boundary is study-internal and requires cohort validation	NORMAL NATIVE
Complete no-hit cluster	**NO**	A complete no-hit is handled separately from a measurable finite opening	NO ACCEPTED NATIVE INTERNAL DISCONTINUITY CLUSTER.
Final acetabular route	**NORMAL NATIVE**	Native ray + low-only coverage policy + route controller	NATIVE ACETABULAR BOX COMPLETE

Decisive native acetabular fracture-evidence step: the Check-2 shell supplies only the expected socket/angular geometry. Actual bone support is determined from the original CT inside the ipsilateral innominate. The peripheral rim band and inferior rays are excluded. Accepted internal clusters are spatially connected CT-supported outliers/no-hit regions with supported borders. Radial opening is measured along socket-centred rays and is distinct from 3-D cluster span. The 10-mm routing boundary is a study-internal threshold awaiting direct cohort validation.

Pelvic CT Segmentation and Quantitative Anatomical Analysis

Pubic Symphysis

PUBIC SYMPHYSIS: BORDERLINE WIDENING DIASTASIS NOT CONFIRMED; GAP 7.1 mm, AP/SI SHEAR -0.1 mm/1.1 mm.

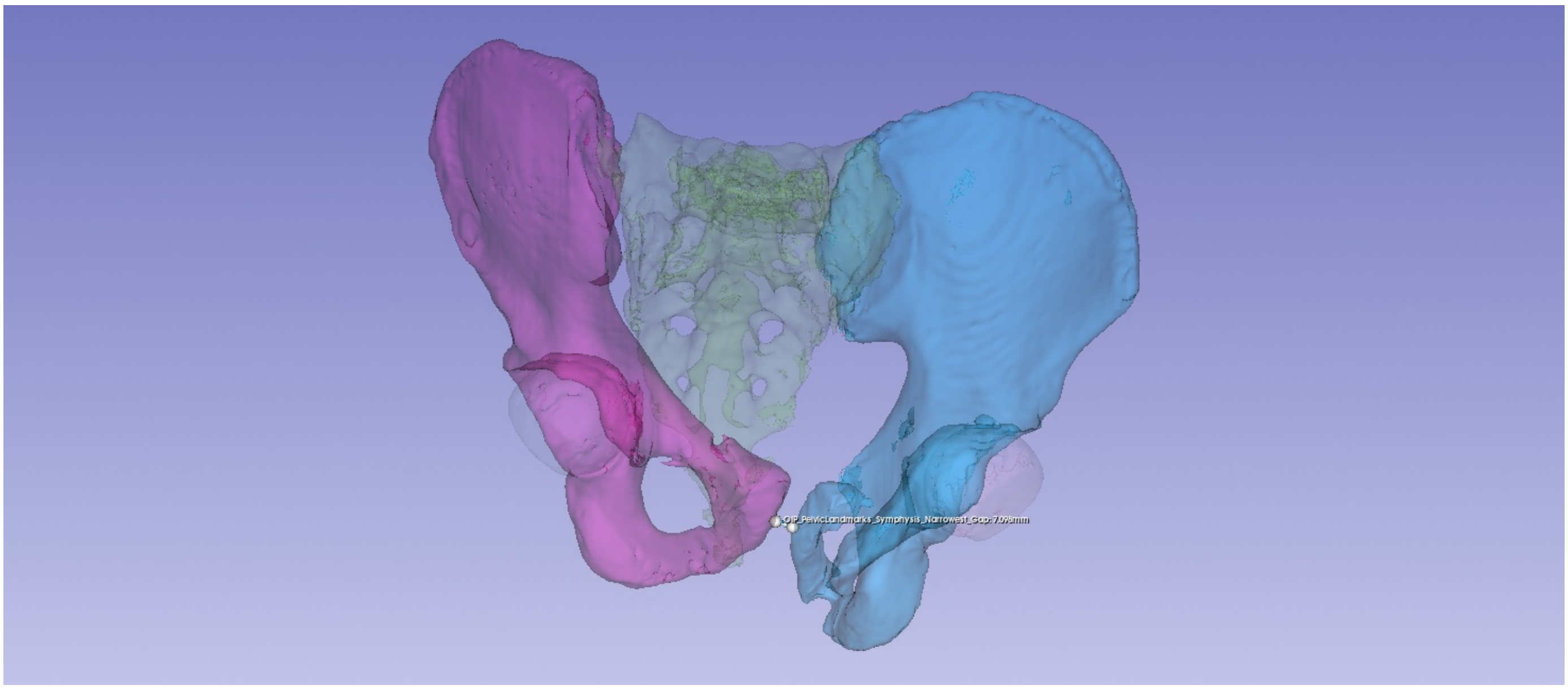

MEASUREMENT	RESULT	REFERENCE / COMPARATOR	INTERPRETATION
Narrowest symphyseal gap	**7.1 mm**	Manual axial-CT mean 3.1 +/- 1.5 mm; calculated mean +/-2SD approximately 0.1-6.1 mm [1]	BORDERLINE WIDENING DIASTASIS NOT CONFIRMED
AP / SI shear	**-0.1 mm / 1.1 mm**	No validated static-CT cutoff for this automated measurement; the published 5-mm value refers to dynamic flamingo radiographs [4]	Descriptive only

Narrowest stable left-right symphyseal gap. Hwang et al. used a manual axial-CT measurement; the calculated 6.1-mm mean+2SD value is descriptive rather than diagnostic [1].

Pelvic CT Segmentation and Quantitative Anatomical Analysis

Obturator Ring

OBTURATOR RING: Right no reportable rim discontinuity; FLAGGED: Left 2 rim candidate(s), maximum bridge chord 40.0 mm; 5 mm or larger.

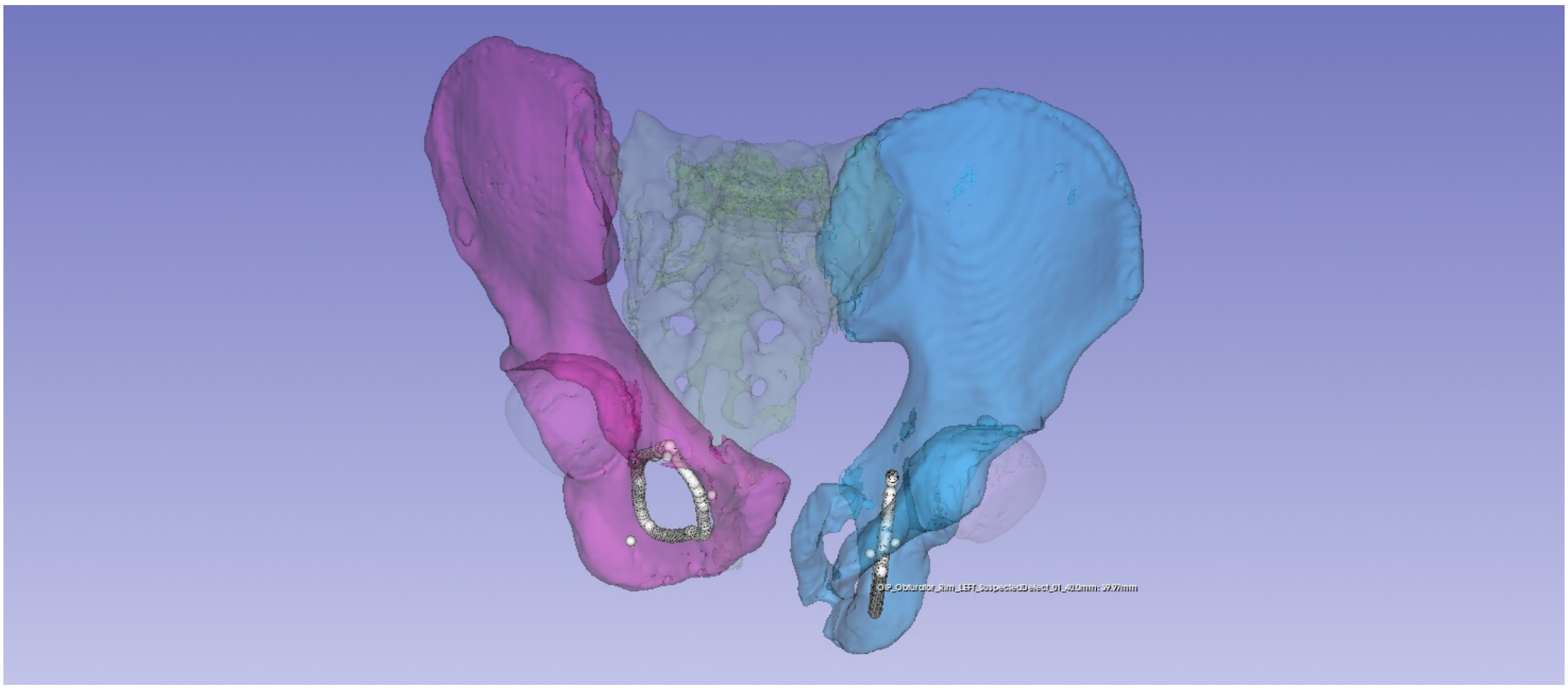

MEASUREMENT	RESULT	REFERENCE / COMPARATOR	INTERPRETATION
Right AP / SI extent	**43.0 mm / 35.4 mm**	Dry-bone mean AP 52.4 +/- 8.0 mm; SI 46.9 +/- 18.3 mm [7]	Descriptive dry-bone comparator only
Right rim discontinuity	**0**	4-mm registered pelvic asymmetry level is an alert comparator, not a fracture cutoff [6]	Visual confirmation required
Left AP / SI extent	**48.8 mm / 52.7 mm**	Dry-bone mean AP 51.7 +/- 8.7 mm; SI 46.6 +/- 16.9 mm [7]	Descriptive dry-bone comparator only
Left rim discontinuity	**2**	4-mm registered pelvic asymmetry level is an alert comparator, not a fracture cutoff [6]	Visual confirmation required

The inner obturator rim is tracked through 360 degrees. The 4-mm literature value is a registered pelvic-asymmetry comparator, not a fracture cutoff; dry-bone AP/SI dimensions are descriptive only [6,7].

Pelvic CT Segmentation and Quantitative Anatomical Analysis

Sacroiliac Joints

SACROILIAC JOINTS ASSESSED: SUSPECTED BILATERAL OR SYMMETRIC SIJ WIDENING; P95 side difference axial 2.4 mm / coronal 0.7 mm; width wider than age reference.

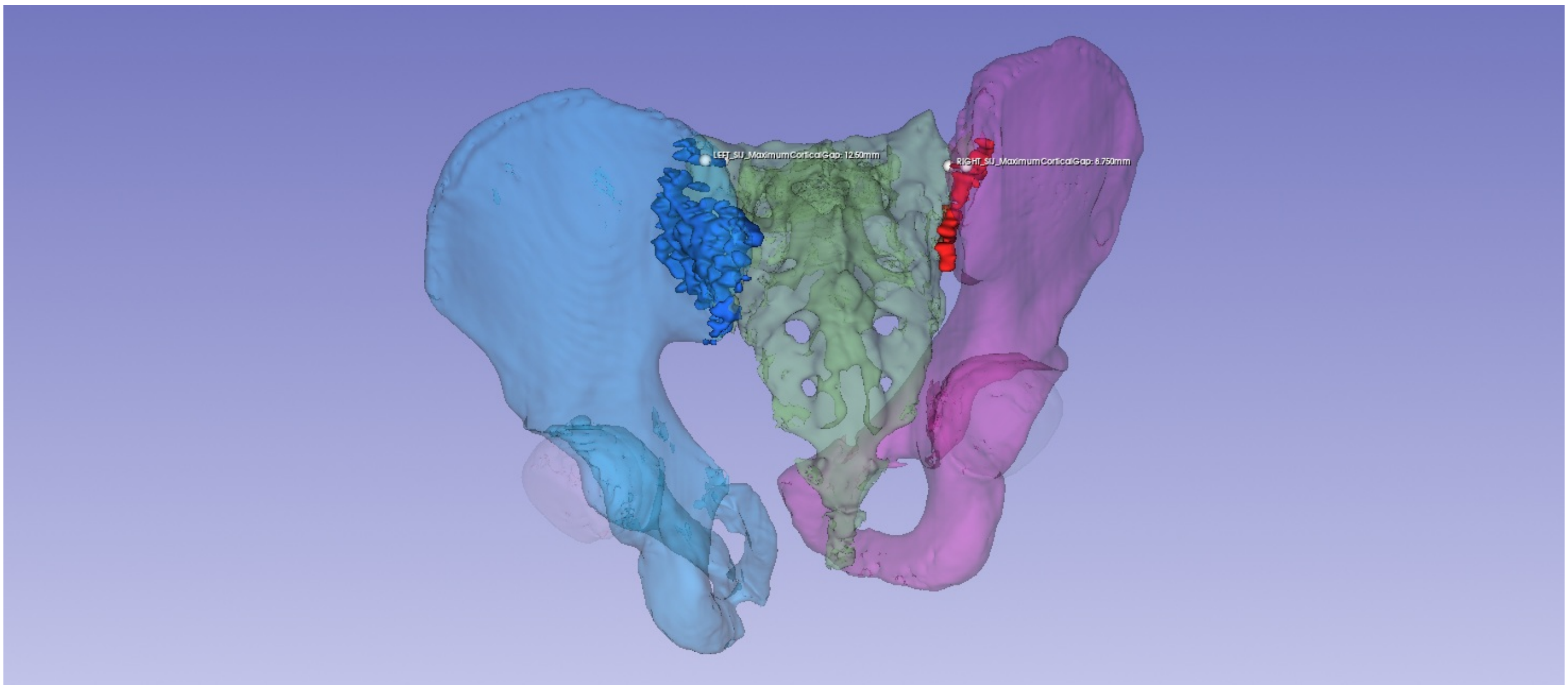

MEASUREMENT	RESULT	REFERENCE / COMPARATOR	INTERPRETATION
Right median cortical gap	**3.8 mm**	Published CT reference: 2.3 +/- 0.4 mm for patients <40 y and 1.9 +/- 0.2 mm for patients >40 y; patient age not supplied [8]	WIDER THAN AGE REFERENCE
Left median cortical gap	**5.0 mm**	Published CT reference: 2.3 +/- 0.4 mm for patients <40 y and 1.9 +/- 0.2 mm for patients >40 y; patient age not supplied [8]	WIDER THAN AGE REFERENCE
Axial / coronal side difference	**2.4 mm / 0.7 mm**	Study-specific open-reduction context: 3.39 / 3.71 mm; absolute 6.52 / 7.26 mm [9]	SUSPECTED BILATERAL OR SYMMETRIC SIJ WIDENING

Opposing sacral and iliac cortices localize the SI joint, and original CT is sampled along local normals. Published normal widths and trauma cut-points are comparators because the automated 3-D construction differs from manual MPR measurements [8,9].

Pelvic CT Segmentation and Quantitative Anatomical Analysis

Final Innominate Partition

FINAL INNOMINATE PARTITION: EXACT BILATERAL RECONSTRUCTION. Current method: ROOT10 single-plane pubis/ischium split.

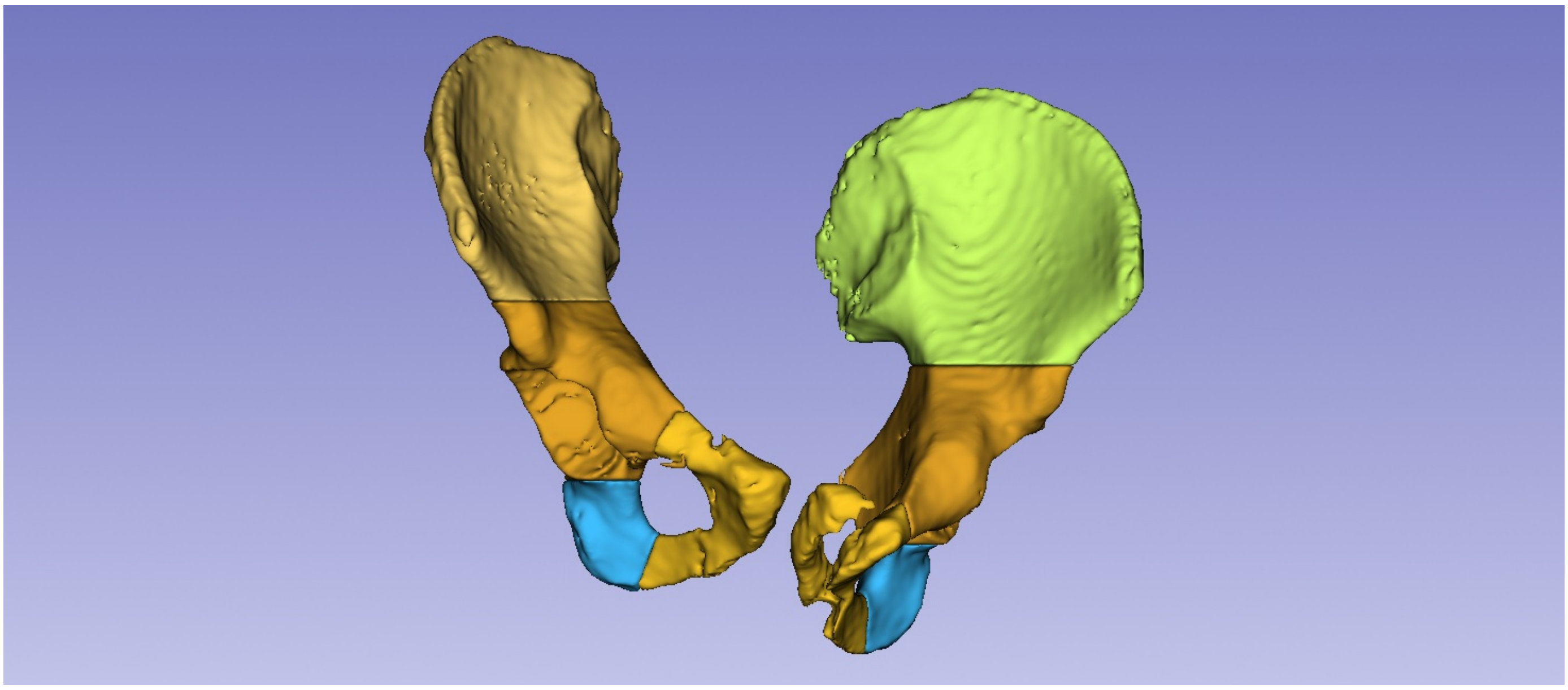

MEASUREMENT	RESULT	REFERENCE / COMPARATOR	INTERPRETATION
Right reconstruction	**PASS**	Technical requirement: zero overlap/unassigned/outside voxels.	ROOT10 single-plane pubis/ischium split
Left reconstruction	**PASS**	Technical requirement: zero overlap/unassigned/outside voxels.	ROOT10 single-plane pubis/ischium split

The innominate is reconstructed as mutually exclusive acetabulum, ilium, pubis, and ischium. Zero overlap, unassigned, and outside voxels are technical consistency requirements.

Pelvic CT Segmentation and Quantitative Anatomical Analysis

Technical Measurements - Page 1

**ROUTE / QUALITY CONTROL**	
Report status	VALID
Initial pathway	NORMAL
Final state	NORMAL NATIVE
Midsagittal-plane QC	PASS — BOTH CENTRAL BONES ARE NEAR-EQUAL
Route notes	Registered mirror-based abnormal reconstruction outputs are not applicable to this route.
**1. FEMORAL-HEAD MORPHOLOGY AND CENTRE - TECHNICAL MEASUREMENTS**	
**RIGHT**	
Centre confidence	HIGH
Fitted radius	23.40 mm
Sphere RMS	0.51 mm
Normalized RMS	2.20%
P95 residual	1.09 mm
Meaningful components	1
**LEFT**	
Centre confidence	HIGH
Fitted radius	22.79 mm
Sphere RMS	0.74 mm
Normalized RMS	3.26%
P95 residual	1.59 mm
Meaningful components	1
**BILATERAL QC**	
Radius difference	0.62 mm
Radius difference	2.67%
Bilateral radius QC	PASS
**2. NATIVE HEAD-SOCKET SHELL AND CONGRUITY - TECHNICAL MEASUREMENTS**	
**RIGHT**	
Margin status	CONTINUOUS
Structure class	INTACT CONTACT MARGIN
Local congruity	CONGRUENT
Full-head coverage	49.38%
Superior-head coverage	68.60%
Shell area	5219.84 mm²
Meaningful components	1
Median head-to-shell gap	3.05 mm
P95 head-to-shell gap	5.49 mm
**LEFT**	
Margin status	CONTINUOUS
Structure class	INTACT CONTACT MARGIN
Local congruity	CONGRUENT
Full-head coverage	42.43%

Pelvic CT Segmentation and Quantitative Anatomical Analysis

Technical Measurements - Page 2

Superior-head coverage	59.72%
Shell area	4742.26 mm²
Meaningful components	1
Median head-to-shell gap	2.76 mm
P95 head-to-shell gap	6.17 mm
**3. MIDSAGITTAL PLANE AND CENTRAL-BONE QC - TECHNICAL MEASUREMENTS**	
Overall plane QC	PASS — BOTH CENTRAL BONES ARE NEAR-EQUAL
**L5**	
RIGHT volume	27864.50 mm3
LEFT volume	25531.11 mm3
Bilateral difference	8.74%
Smaller/larger ratio	0.9163
Symmetry QC	NEAR-EQUAL
**Sacrum**	
RIGHT volume	90312.01 mm3
LEFT volume	99173.01 mm3
Bilateral difference	9.35%
Smaller/larger ratio	0.9107
Symmetry QC	NEAR-EQUAL
**5. NATIVE ORIGINAL-CT ACETABULAR DISCONTINUITY — DECISIVE RAY TEST - TECHNICAL MEASUREMENTS**	
Accepted cluster total	0
Maximum radial opening	**NA**
Complete no-hit cluster	False
Radial opening routing gate	10.00 mm
Ray route	NO ACCEPTED CLUSTER
Routing basis	No accepted native internal discontinuity cluster.
**RIGHT**	
Internal rays tested	2233
CT-supported internal rays	2232
Raw internal candidates	0
Accepted defect rays	0
Accepted defect clusters	0
Maximum side radial opening	**NA**
Complete no-hit cluster	False
Defect status	NO INTERNAL ACETABULAR DISCONTINUITY DETECTED
**LEFT**	
Internal rays tested	2015
CT-supported internal rays	2015
Raw internal candidates	0
Accepted defect rays	0
Accepted defect clusters	0
Maximum side radial opening	**NA**

Pelvic CT Segmentation and Quantitative Anatomical Analysis

Technical Measurements - Page 3

Complete no-hit cluster	False
Defect status	NO INTERNAL ACETABULAR DISCONTINUITY DETECTED
**PUBIC SYMPHYSIS - TECHNICAL MEASUREMENTS**	
Clinical status	ABNORMAL
Screening classification	BORDERLINE WIDENING DIASTASIS NOT CONFIRMED
Stable narrowest gap	7.15 mm
Selected pair gap	7.01 mm
Three-dimensional point distance	7.10 mm
AP shear	-0.07 mm
SI shear	1.13 mm
Supporting local cells	25
Local gap IQR	0.12 mm
**OBTURATOR RING - TECHNICAL MEASUREMENTS**	
**RIGHT**	
AP extent	42.98 mm
SI extent	35.44 mm
Area	1324.90 mm²
Perimeter	150.48 mm
Tracked rim	100.00%
Tracked rim outside 4-mm band	75.56%
Suspected discontinuities	0
**LEFT**	
AP extent	48.84 mm
SI extent	52.71 mm
Area	1390.27 mm²
Perimeter	173.91 mm
Tracked rim	66.11%
Tracked rim outside 4-mm band	77.73%
Suspected discontinuities	2
Defect 1 bridge chord	39.97 mm
Defect 1 estimated missing rim arc	46.97 mm
Defect 2 bridge chord	7.75 mm
Defect 2 estimated missing rim arc	5.07 mm
**SACROILIAC JOINTS - TECHNICAL MEASUREMENTS**	
Width classification	WIDER THAN AGE REFERENCE
Median cortical width	3.75 mm
P95 cortical width	5.75 mm
Maximum cortical width	8.75 mm
P95 axial projection	5.32 mm
P95 coronal projection	5.54 mm
CT-validated rays	178
Validation fraction	0.978

Pelvic CT Segmentation and Quantitative Anatomical Analysis

Technical Measurements - Page 4

Bilateral trauma screen	SUSPECTED BILATERAL OR SYMMETRIC SIJ WIDENING
Width classification	WIDER THAN AGE REFERENCE
Median cortical width	5.00 mm
P95 cortical width	8.00 mm
Maximum cortical width	12.50 mm
P95 axial projection	7.71 mm
P95 coronal projection	6.23 mm
CT-validated rays	772
Validation fraction	0.995
Bilateral trauma screen	SUSPECTED BILATERAL OR SYMMETRIC SIJ WIDENING
**FINAL INNOMINATE PARTITION - TECHNICAL MEASUREMENTS**	
**RIGHT**	
Exact reconstruction	True
Unassigned voxels	0
Outside voxels	0
Overlap voxels	0
Acetabulum voxels	182179
Ilium voxels	224747
Pubis voxels	45242
Ischium voxels	40361
**LEFT**	
Exact reconstruction	True
Unassigned voxels	0
Outside voxels	0
Overlap voxels	0
Acetabulum voxels	185084
Ilium voxels	236615
Pubis voxels	39105
Ischium voxels	37399
**CURRENT PARTITION METHOD**	
Method	ROOT10 single-plane pubis/ischium split
Split mode	BOX ANCHOR FINITE20 ROOT CORRIDOR ILIUM FLOOR
Obturator used for split	False
Mirroring used for split	False
8-mm reclamation	False

Pelvic CT Segmentation and Quantitative Anatomical Analysis

References

Published comparators are interpreted according to their original measurement definitions; where the automated measurement differs, comparison is descriptive rather than a diagnostic cutoff.

[1] Hwang K, Wu X, Park CY. Width of pubic symphysis relating to age and sex in Koreans. J Orthop Surg Res. 2021;16(1):430. doi:10.1186/s13018-021-02561-9. PMID:34217332.

[2] Doro CJ, Forward DP, Kim H, Nascone JW, Sciadini MF, Hsieh AH, et al. Does 2.5 cm of symphyseal widening differentiate anteroposterior compression I from anteroposterior compression II pelvic ring injuries? J Orthop Trauma. 2010;24(10):610-615. doi:10.1097/BOT.0b013e3181cff42c. PMID:20871248.

[3] Gansslen A, Lindahl J, Krappinger D, Lindtner RA, Staresinic M. The myth of 2.5 cm symphyseal diastasis. Arch Orthop Trauma Surg. 2025;145(1):306. doi:10.1007/s00402-025-05904-x. PMID:40397156.

[4] Garras DN, Carothers JT, Olson SA. Single-leg-stance (flamingo) radiographs to assess pelvic instability: how much motion is normal? J Bone Joint Surg Am. 2008;90(10):2114-2118. doi:10.2106/JBJS.G.00277. PMID:18829908.

[5] Larson CM, Moreau-Gaudry A, Kelly BT, Byrd JWT, Tonetti J, Lavallee S, et al. Are normal hips being labeled as pathologic? A CT-based method for defining normal acetabular coverage. Clin Orthop Relat Res. 2015;473(4):1247-1254. doi:10.1007/s11999-014-4055-2. PMID:25407391.

[6] Zhang F, Zhang D, Huang Z, Wang Z, Cai X. Morphological asymmetry of pelvic rings: a study based on three-dimensional deviation analysis. Orthop Surg. 2022;14(5):967-976. doi:10.1111/os.13246. PMID:35377554.

[7] Duygu OB, Govsa F. Anatomical structures of the obturator foramen and its importance for pelvic interventions. Surg Radiol Anat. 2025;47(1):163. doi:10.1007/s00276-025-03661-5. PMID:40544414.

[8] Faflia CP, Prassopoulos PK, Daskalogiannaki ME, Gourtsoyiannis NC. Variation in the appearance of the normal sacroiliac joint on pelvic CT. Clin Radiol. 1998;53(10):742-746. doi:10.1016/S0009-9260(98)80316-4. PMID:9817091.

[9] Chien RS, Chen IJ, Lai CY, Chen JP, Yu YH. Critical distance of the sacroiliac joint for open reduction using screw fixation for traumatic sacroiliac joint diastasis: a retrospective study. J Orthop Surg Res. 2024;19(1):268. doi:10.1186/s13018-024-04759-z. PMID:38678298.

[10] Jamali AA, Mak W, Wang P, Tai L, Meehan JP, Lamba R. What is normal femoral head/neck anatomy? An analysis of radial CT reconstructions in adolescents. Clin Orthop Relat Res. 2013;471(11):3581-3587. doi:10.1007/s11999-013-3166-5. PMID:23922189.

[11] Hassan MM, Feroe AG, Douglass BW, Jimenez AE, Kuhns B, Mitchell CF, et al. Three-dimensional analysis of age and sex differences in femoral head asphericity in asymptomatic hips in the United States. World J Orthop. 2024;15(8):754-763. doi:10.5312/wjo.v15.i8.754. PMID:39165879.

[12] Rivas DJL, Gassmann JM, Goetz JE, Aitken HD, Davison JC, Miller A, et al. Weight-bearing CT as an approach to assess femoral-acetabular displacement during external rotation stress in the hip. J Hip Preserv Surg. 2025;12(2):93-104. doi:10.1093/jhps/hnaf001. PMID:40761572.

[13] Judet R, Judet J, Letournel E. Fractures of the acetabulum: classification and surgical approaches for open reduction. Preliminary report. J Bone Joint Surg Am. 1964;46:1615-1646. doi:10.2106/00004623-196446080-00001. PMID:14239854.

[14] Imani M, Buratto J, Dao T, Meijering E, Vogrin S, Kwok TCY, et al. Deep learning technique for automatic segmentation of proximal hip musculoskeletal tissues from CT scan images: a MrOS study. J Cachexia Sarcopenia Muscle. 2025;16(2):e13728. doi:10.1002/jcsm.13728. PMID:40022453.

[15] Ead MS, Duke KK, Jaremko JL, Westover L. Investigation of pelvic symmetry using CAD software. Med Biol Eng Comput. 2020;58(1):75-82. doi:10.1007/s11517-019-02068-w. PMID:31745838.

[16] Amar E, Druckmann I, Flusser G, Safran MR, Salai M, Rath E. The anterior inferior iliac spine: size, position, and location. An anthropometric and sex survey. Arthroscopy. 2013;29(5):874-881. doi:10.1016/j.arthro.2013.01.023. PMID:23523127.

[17] Rana SH, Farjoodi P, Haloman S, Dutton P, Hariri A, Ward SR, et al. Anatomic evaluation of the sacroiliac joint: a radiographic study with implications for procedures. Pain Physician. 2015;18(6):583-592. PMID:26606010.

[18] Hartel MJ, Petersik A, Schmidt A, Kendoff D, Nuchtern J, Rueger JM, et al. Determination of femoral neck angle and torsion angle utilizing a novel three-dimensional modeling and analytical technology based on CT datasets. PLoS One. 2016;11(3):e0149480. doi:10.1371/journal.pone.0149480.

[19] Mahmood SS, Mukka SS, Crnalic S, Wretenberg P, Sayed-Noor AS. Association between changes in global femoral offset after total hip arthroplasty and function, quality of life, and abductor muscle strength. Acta Orthop. 2016;87(1):36-41. doi:10.3109/17453674.2015.1091955.

[20] O'Brien S, Kernohan G. Perception of imposed leg length inequality in normal subjects. Hip Int. 2010;20(4):505-511. doi:10.1177/112070001002000414.

[21] Wang G, et al. Measurement of relative position of femoral head center, greater trochanter, and lesser trochanter. Ann Palliat Med. 2021;10(11):11524-11528. doi:10.21037/apm-21-2538.

Pelvic CT Segmentation and Quantitative Anatomical Analysis

Clinical Findings Summary

Case ID: PRFX_0010_0000 | Age: Not supplied | Status: VALID

CONCLUSIVE FINDING

Automated CT analysis flags joint-space narrowing on Right and Left; femoral parameter assessment flagged; Right obturator-rim discontinuity of at least 5 mm. In addition, 1 small (<5 mm) candidate finding(s) require confirmation. Femoral parameter assessment is flagged; see the dedicated evidence page.

OVERALL STATION ASSESSMENT

Flagged finding of at least 5 mm in the hip/acetabular, anterior-ring station group(s).

Clinical station checklist

HIP JOINT AND ACETABULUM

Femoral-head morphology — NO FLAG
R VALID HEAD CENTRE — NORMAL-LIKE MORPHOLOGY / L VALID HEAD CENTRE — NORMAL-LIKE MORPHOLOGY

Head-socket congruity — NO FLAG
R CONGRUENT / L CONGRUENT

Acetabular coverage — NO FLAG
R 43.9% / L 46.0%; no low-coverage flag

Acetabular continuity — CAUTION
1 accepted cluster(s); maximum 3-D span 4.2 mm; small finding may be irrelevant/artifactual; confirm on source CT

Head position / dislocation — NO FLAG
No displacement pathway activated; bilateral native assessment

Joint-space / arthrosis screen — FLAGGED
R NARROWED — OSTEOARTHRITIS CANDIDATE (2.50 / -0.50 / 7.00 mm) / L NARROWED — OSTEOARTHRITIS CANDIDATE (2.00 / -0.50 / 7.50 mm)

Femur parameters — FLAGGED
Automated femoral analysis flags right 3D NSA above Hartel true-3D CT IQR; left 3D NSA above Hartel true-3D CT IQR; limb-length discrepancy 14.3 mm (right shorter); right head-centre-to-lesser I/D

ANTERIOR PELVIC RING

Overall anterior ring screen — FLAGGED
Discontinuity finding of at least 5 mm at an anterior-ring station

Pubic symphysis — NO FLAG
Gap 6.0 mm; NO CT EVIDENCE OF SYMPHYSEAL DIASTASIS

Right obturator ring — FLAGGED
2 candidate(s); maximum bridge chord 79.3 mm; 5 mm or larger discontinuity

Left obturator ring — NO FLAG
0 reportable rim discontinuity candidates

POSTERIOR PELVIC RING

Right SIJ integrity — NO FLAG
median 0.2 mm; P95 0.2 mm; NARROWER THAN AGE REFERENCE

Left SIJ integrity — NO FLAG
median 4.0 mm; P95 7.0 mm; WIDER THAN AGE REFERENCE

SCOPE: Joint-space narrowing is an arthrosis/coxarthrosis screen, not a stand-alone diagnosis. These station tests do not exclude isolated or minimally displaced iliac or sacral fractures; review the source CT.

SIJ references: normal CT mean 2.3 +/- 0.4 mm (<40 y) and 1.9 +/- 0.2 mm (>40 y) [8]; traumatic axial/coronal distance context [9]; anatomical extent/search-corridor context [17].

Detailed anatomical evidence and quantitative measurements follow on the next pages.

Pelvic CT Segmentation and Quantitative Anatomical Analysis

1. Femoral-Head Morphology and Centre

RIGHT FEMORAL HEAD: VALID HEAD CENTRE — NORMAL-LIKE MORPHOLOGY; CENTRE HIGH CONFIDENCE. LEFT FEMORAL HEAD: VALID HEAD CENTRE — NORMAL-LIKE MORPHOLOGY; CENTRE HIGH CONFIDENCE.

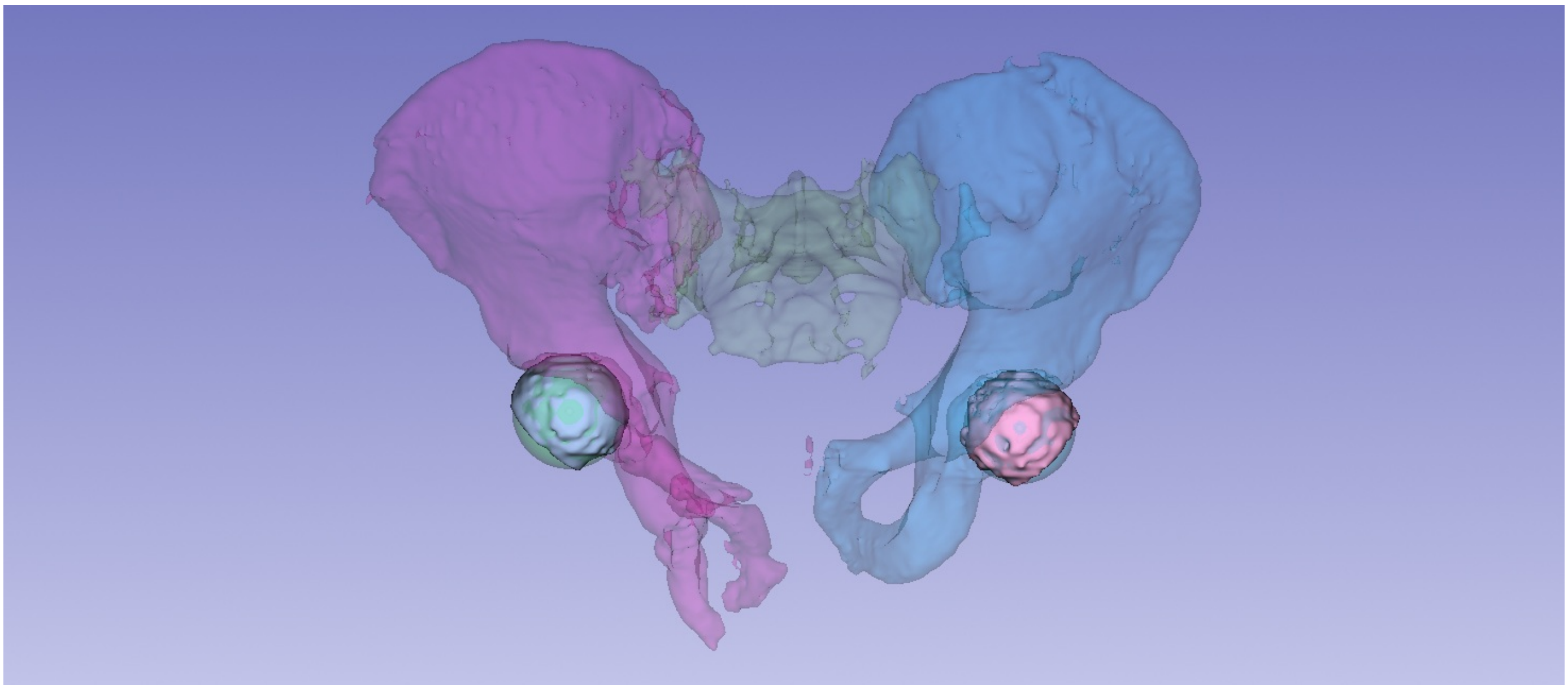

MEASUREMENT	RESULT	REFERENCE / COMPARATOR	INTERPRETATION
Right fitted radius	**22.7 mm**	Adolescent radial-CT comparator: male 24.53 +/- 1.74 mm; female 21.94 +/- 1.13 mm [10]	VALID HEAD CENTRE — NORMAL-LIKE MORPHOLOGY
Left fitted radius	**22.2 mm**	Adolescent radial-CT comparator: male 24.53 +/- 1.74 mm; female 21.94 +/- 1.13 mm [10]	VALID HEAD CENTRE — NORMAL-LIKE MORPHOLOGY
Sphere-fit residual	**R/L P95 1.4 mm/1.9 mm**	Published 3-D sphere/asphericity analysis supports the measurement family; these residual limits are study-specific [11]	Quantitative morphology QC

Best-fit sphere radius and residuals are reported from the segmented femoral head. Published 3-D CT/asphericity work supports this measurement family, while the residual QC limits are study-specific [10,11].

Pelvic CT Segmentation and Quantitative Anatomical Analysis

Femur Subsegmentation and Quantitative Parameters

Automated femoral analysis flags right 3D NSA above Hartel true-3D CT IQR; left 3D NSA above Hartel true-3D CT IQR; limb-length discrepancy 14.3 mm (right shorter); right head-centre-to-lesser L/D above the published interval; left head-centre-to-lesser L/D above the published interval.

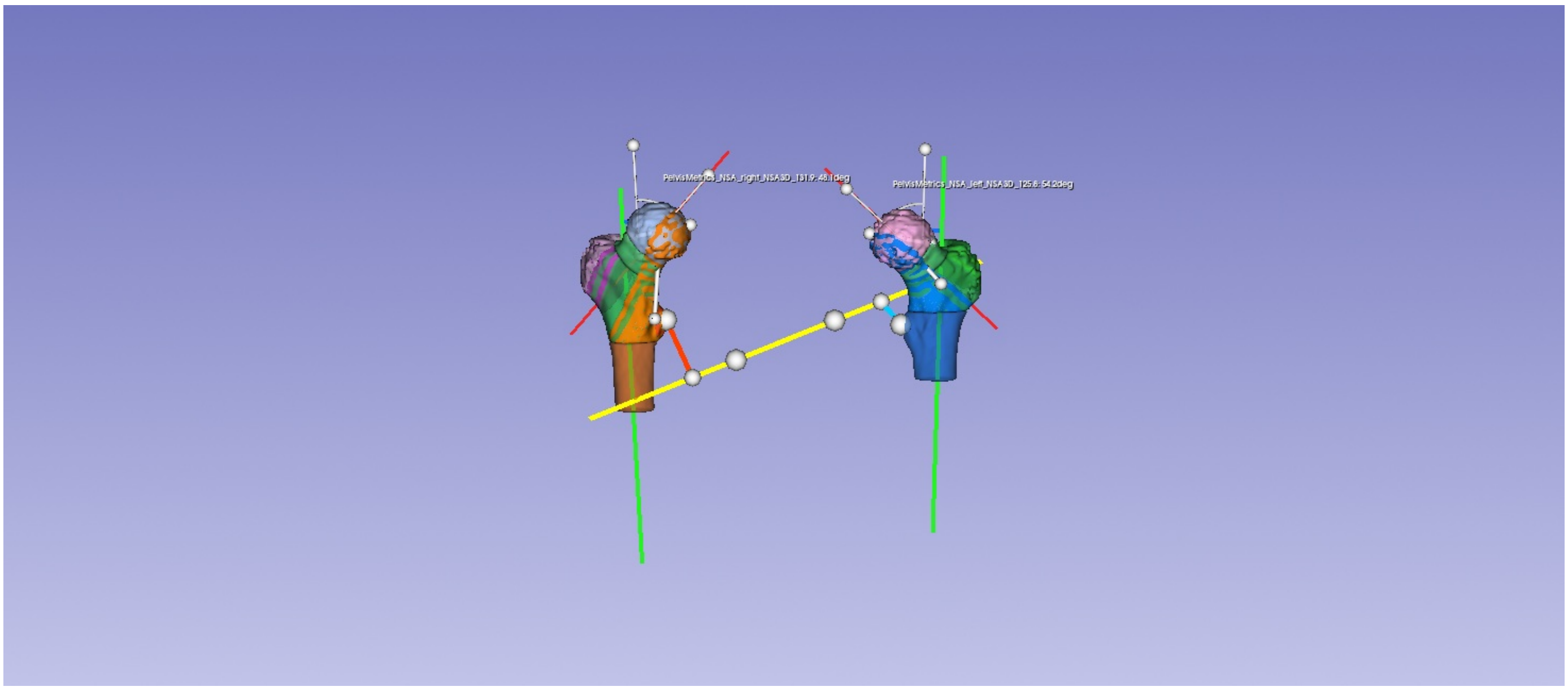

MEASUREMENT	RESULT	REFERENCE / COMPARATOR	INTERPRETATION
True-3D neck-shaft angle (R / L)	**131.87 / 125.78 deg**	Hartel CT IQR 117.9-125.6 deg [18]	FLAGGED HIGH / FLAGGED HIGH
NSA absolute bilateral difference	**6.1 deg**	Quantitative side comparison; not a stand-alone diagnosis [18]	FLAGGED
Femoral offset (R / L)	**27.1 / 30.53 mm**	Absolute native CT values are quantitative only [19]	SYMMETRIC BY COMPARATOR
Offset absolute side difference	**3.4 mm**	>=5 mm reconstruction-asymmetry comparator [19]	BELOW COMPARATOR
Limb-length discrepancy	**14.3 mm**	>=10 mm comparator [20]	FLAGGED LLD; Right shorter
Head-centre-to-lesser L/D — Right	**1.413**	AP-radiographic mean 0.84 +/- 0.08; screening interval 0.68-1.00 [21]	L/D is above the published screening interval; review the lesser point and head geometry.
Head-centre-to-lesser L/D — Left	**1.000**	AP-radiographic mean 0.84 +/- 0.08; screening interval 0.68-1.00 [21]	L/D is above the published screening interval; review the lesser point and head geometry.
Integrated femur assessment	**FLAGGED**	Automated quantitative screening; CT/source-segmentation confirmation required	Automated femoral analysis flags right 3D NSA above Hartel true-3D CT IQR; left 3D NSA above Hartel true-3D CT IQR; limb-length discrepancy 14.3 mm (right shorter); right head-centre-to-lesser L/D above the published interval; left head-centre-to-lesser L/D above the published interval.

The displayed femur subsegments preserve the locked RAS geometry and existing lesser-trochanter result. NSA and offset use the corrected medullary-centre shaft axis; LLD uses bilateral lower-innominate reference geometry. L/D uses the femur-local AP projection (posterior component excluded). Published comparators are screening references, not stand-alone diagnoses [18-21].

Pelvic CT Segmentation and Quantitative Anatomical Analysis

2. Native Head-Socket Shell and Congruity

RIGHT MARGIN CONTINUOUS; INTACT CONTACT MARGIN; CONGRUENT. LEFT MARGIN CONTINUOUS; INTACT CONTACT MARGIN; CONGRUENT.

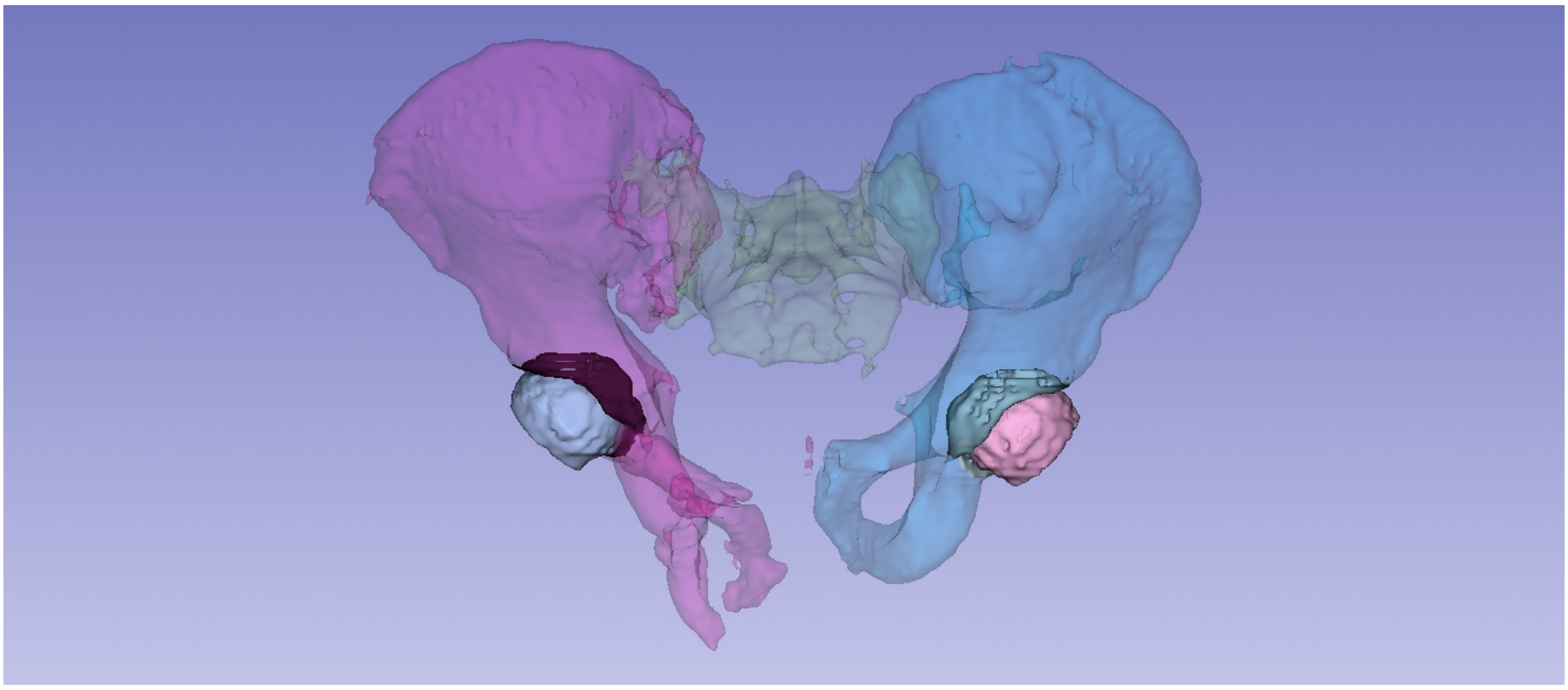

MEASUREMENT	RESULT	REFERENCE / COMPARATOR	INTERPRETATION
Right local gap (median / P95)	**2.4 mm / 4.9 mm**	Healthy-control whole-joint WBCT JSW 3.99 +/- 0.53 mm; different acquisition and geometry [12]	CONGRUENT
Left local gap (median / P95)	**2.0 mm / 6.1 mm**	Healthy-control whole-joint WBCT JSW 3.99 +/- 0.53 mm; different acquisition and geometry [12]	CONGRUENT

Head-centred rays assess acetabular-shell continuity and local gap. The cited weight-bearing CT joint-space value is shown only as context because the acquisition and geometry differ [12].

Pelvic CT Segmentation and Quantitative Anatomical Analysis

3. Midsagittal Plane and Central-Bone QC

MIDSAGITTAL PLANE QC: PASS — PLANE AVAILABLE; CENTRAL-BONE ASYMMETRY IS REPORT-ONLY; L5 18.56%; SACRUM 22.84%.

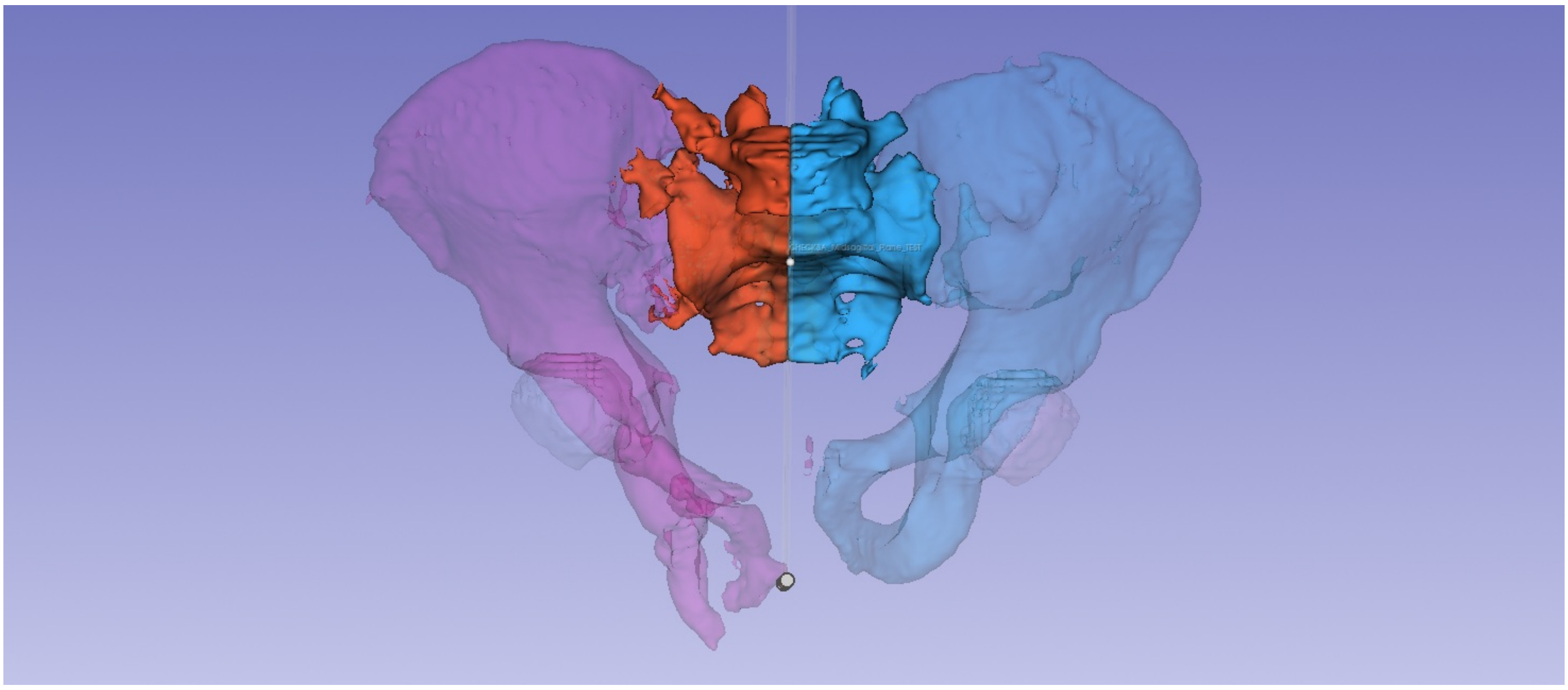

MEASUREMENT	RESULT	REFERENCE / COMPARATOR	INTERPRETATION
L5 bilateral half-volume difference	**18.56%**	Study-internal geometric QC	NOT NEAR-EQUAL
Sacral bilateral half-volume difference	**22.84%**	Study-internal geometric QC	NOT NEAR-EQUAL
Overall plane QC	**PASS — PLANE AVAILABLE; CENTRAL-BONE ASYMMETRY IS REPORT-ONLY**	10% bilateral difference is a study-internal QC rule	Technical plane validation; not a clinical diagnostic cutoff

The pelvic midsagittal plane is accepted only after bilateral L5/sacral half-volume QC. The 10% difference rule is a study-internal geometric QC criterion, not a clinical threshold.

Pelvic CT Segmentation and Quantitative Anatomical Analysis

4. Global Femoral-Head Coverage

GLOBAL FEMORAL-HEAD COVERAGE: RIGHT 43.9%; LEFT 46.0%.

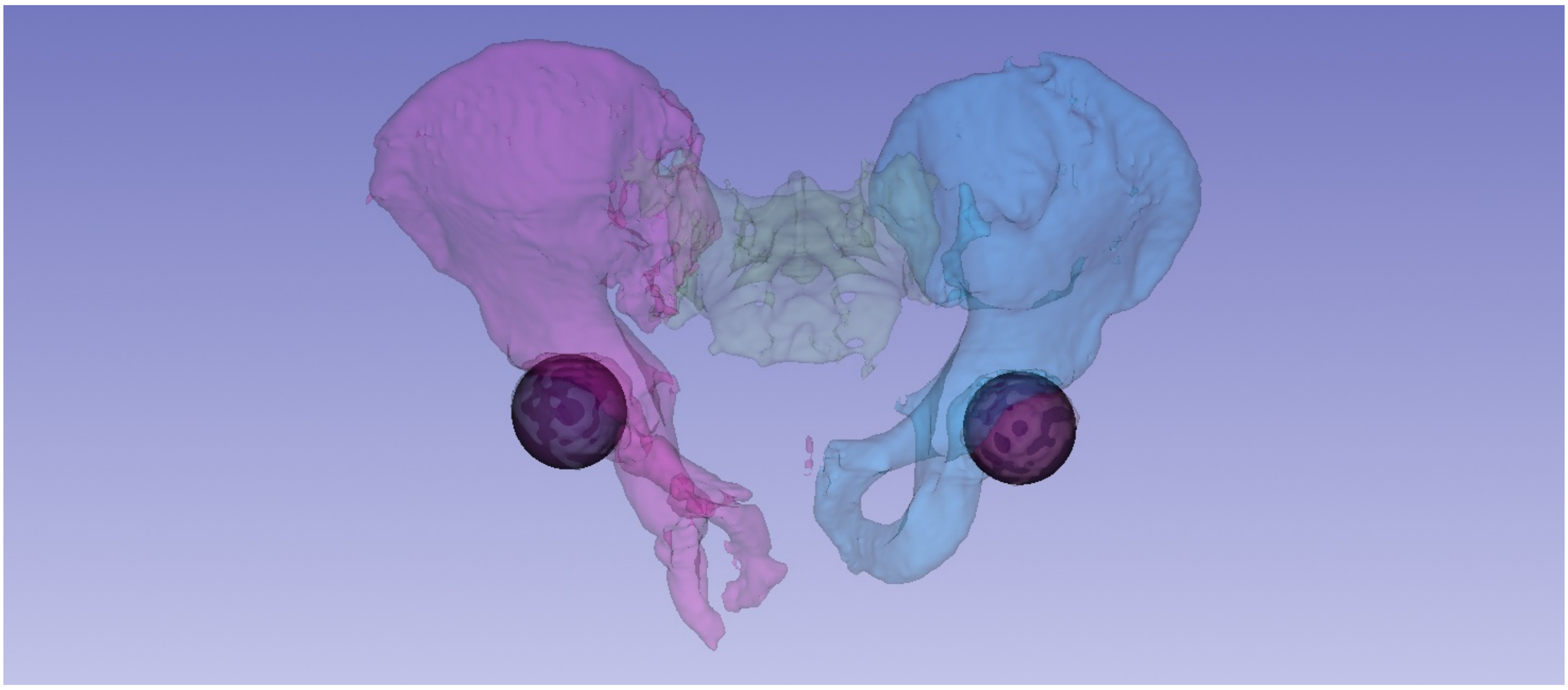

MEASUREMENT	RESULT	REFERENCE / COMPARATOR	INTERPRETATION
Right global coverage	**43.9%**	Asymptomatic CT mean 40 +/- 2%; calculated mean +/-2SD approximately 36-44% [5]	Z=+1.96 | WITHIN APPROXIMATE ASYMPTOMATIC MEAN+/-2SD
Left global coverage	**46.0%**	Asymptomatic CT mean 40 +/- 2%; calculated mean +/-2SD approximately 36-44% [5]	Z=+2.97 | ABOVE APPROXIMATE ASYMPTOMATIC MEAN+2SD — DESCRIPTIVE ONLY

Global curved-surface coverage is measured on a best-fit femoral-head sphere using radial intersection with the acetabular shell. This is an automated adaptation of Larson's method; 40 +/- 2% is the published comparator [5].

Pelvic CT Segmentation and Quantitative Anatomical Analysis

5. Native Original-CT Acetabular Discontinuity — Decisive Ray Test

CAUTION: Left 1 accepted acetabular discontinuity cluster(s); maximum 3-D span 4.2 mm; small finding may be irrelevant/artifactual; source-CT confirmation required.

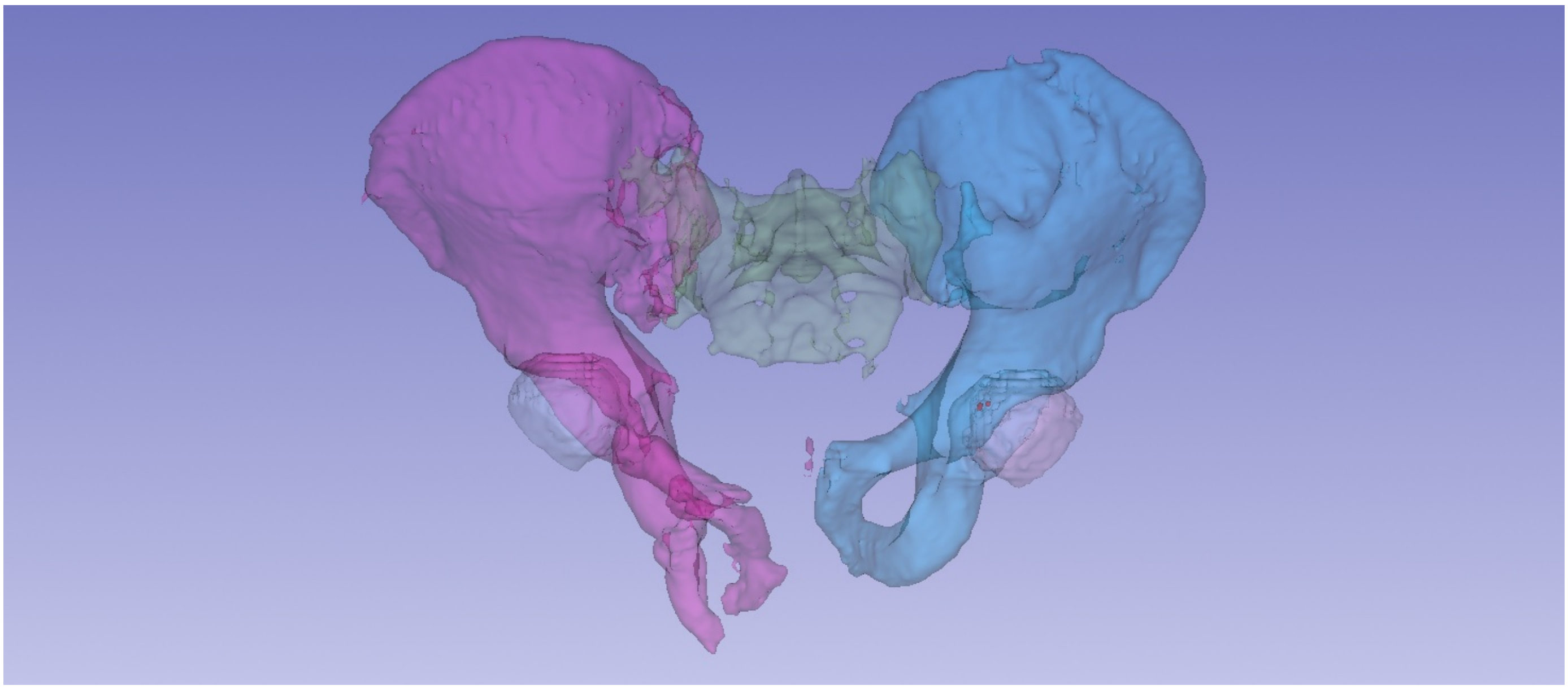

MEASUREMENT	RESULT	REFERENCE / COMPARATOR	INTERPRETATION
Right accepted internal discontinuity clusters	**0**	Original CT support inside the ipsilateral innominate; Check-2 shell supplies expected socket geometry only	NO INTERNAL ACETABULAR DISCONTINUITY DETECTED
Left accepted internal discontinuity clusters	**1**	Original CT support inside the ipsilateral innominate; Check-2 shell supplies expected socket geometry only	INTERNAL ACETABULAR DISCONTINUITY CANDIDATE
Left maximum radial opening	**7.43 mm**	Radial opening is measured along socket-centred rays; it is distinct from 3-D cluster span	Accepted discontinuity geometry
Accepted cluster total	**1**	Native original-CT ray analysis	NATIVE MILD DEFECT
Maximum radial opening	**7.43 mm**	10-mm route boundary is study-internal and requires cohort validation	NATIVE MILD DEFECT
Complete no-hit cluster	**NO**	A complete no-hit is handled separately from a measurable finite opening	MAXIMUM NATIVE RADIAL OPENING 7.43 MM IS < 10.00 MM.
Final acetabular route	**NATIVE MILD DEFECT**	Native ray + low-only coverage policy + route controller	NATIVE ACETABULAR BOX COMPLETE; NO MIRROR DEFECT RECONSTRUCTION

Decisive native acetabular fracture-evidence step: the Check-2 shell supplies only the expected socket/angular geometry. Actual bone support is determined from the original CT inside the ipsilateral innominate. The peripheral rim band and inferior rays are excluded. Accepted internal clusters are spatially connected CT-supported outliers/no-hit regions with supported borders. Radial opening is measured along socket-centred rays and is distinct from 3-D cluster span. The 10-mm routing boundary is a study-internal threshold awaiting direct cohort validation.

Pelvic CT Segmentation and Quantitative Anatomical Analysis

Pubic Symphysis

PUBIC SYMPHYSIS: REFERENCE-COMPATIBLE WIDTH; GAP 6.0 mm, AP/SI SHEAR 2.7 mm/-1.0 mm.

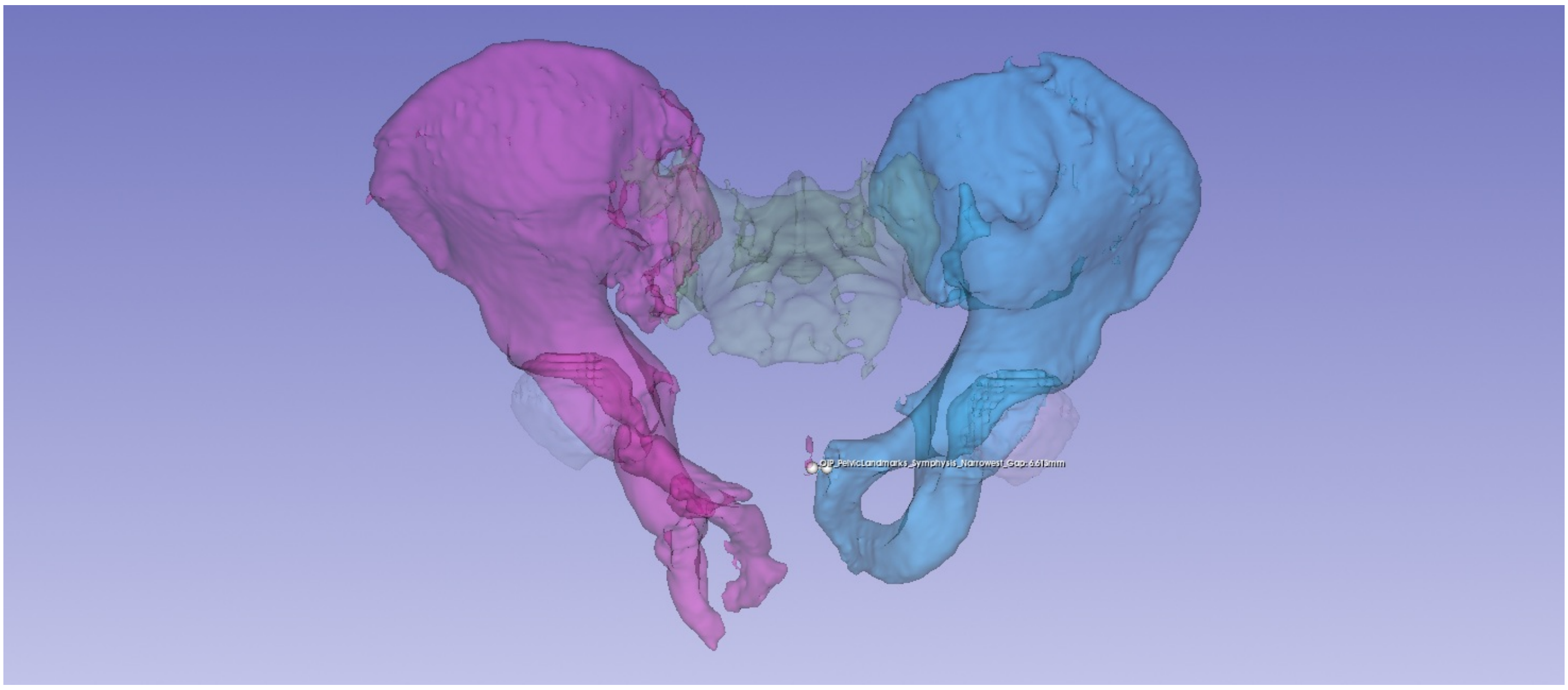

MEASUREMENT	RESULT	REFERENCE / COMPARATOR	INTERPRETATION
Narrowest symphyseal gap	**6.0 mm**	Manual axial-CT mean 3.1 +/- 1.5 mm; calculated mean +/-2SD approximately 0.1-6.1 mm [1]	NO CT EVIDENCE OF SYMPHYSEAL DIASTASIS
AP / SI shear	**2.7 mm / -1.0 mm**	No validated static-CT cutoff for this automated measurement; the published 5-mm value refers to dynamic flamingo radiographs [4]	Descriptive only

Narrowest stable left-right symphyseal gap. Hwang et al. used a manual axial-CT measurement; the calculated 6.1-mm mean+2SD value is descriptive rather than diagnostic [1].

Pelvic CT Segmentation and Quantitative Anatomical Analysis

Obturator Ring

OBTURATOR RING: FLAGGED: Right 2 rim candidate(s), maximum bridge chord 79.3 mm; 5 mm or larger; Left no reportable rim discontinuity.

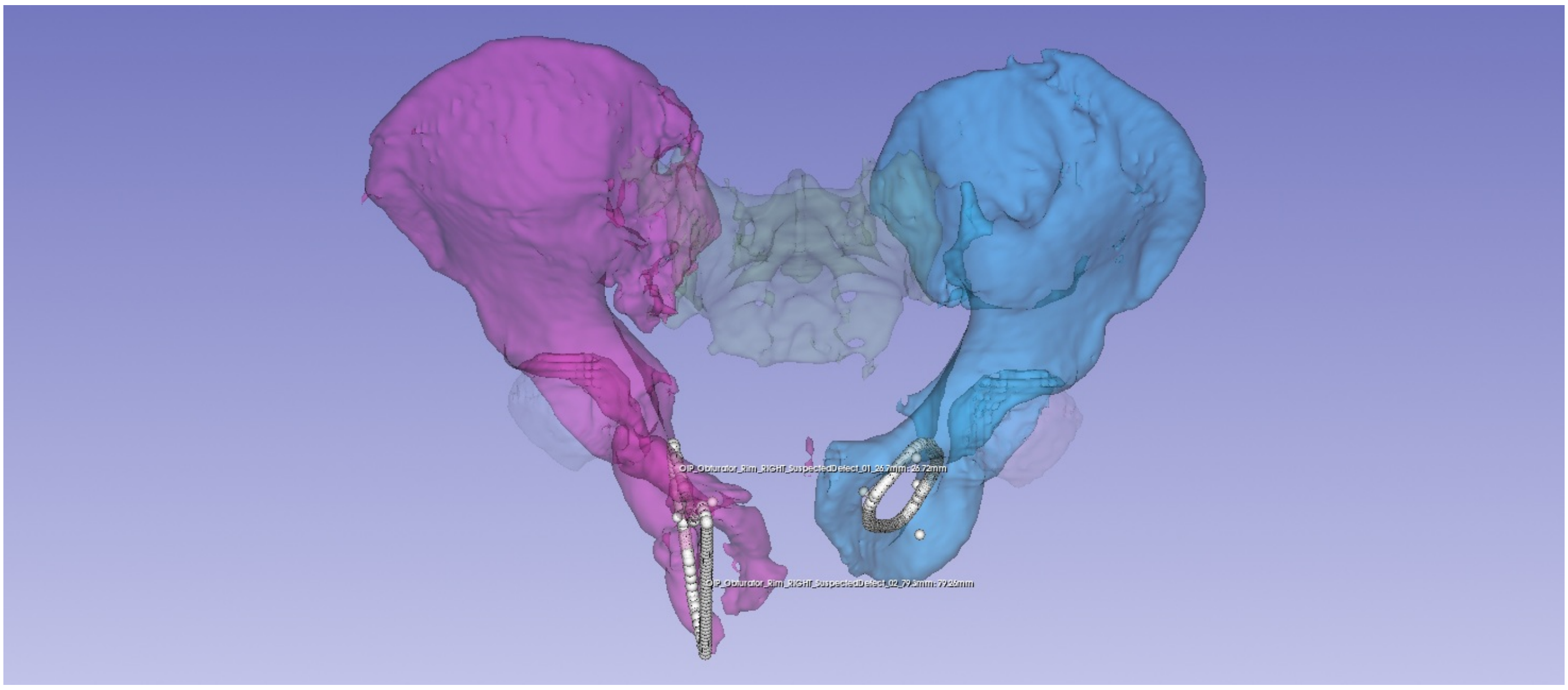

MEASUREMENT	RESULT	REFERENCE / COMPARATOR	INTERPRETATION
Right AP / SI extent	**69.9 mm / 81.4 mm**	Dry-bone mean AP 52.4 +/- 8.0 mm; SI 46.9 +/- 18.3 mm [7]	Descriptive dry-bone comparator only
Right rim discontinuity	**2**	4-mm registered pelvic asymmetry level is an alert comparator, not a fracture cutoff [6]	Visual confirmation required
Left AP / SI extent	**47.0 mm / 32.6 mm**	Dry-bone mean AP 51.7 +/- 8.7 mm; SI 46.6 +/- 16.9 mm [7]	Descriptive dry-bone comparator only
Left rim discontinuity	**0**	4-mm registered pelvic asymmetry level is an alert comparator, not a fracture cutoff [6]	Visual confirmation required

The inner obturator rim is tracked through 360 degrees. The 4-mm literature value is a registered pelvic-asymmetry comparator, not a fracture cutoff; dry-bone AP/SI dimensions are descriptive only [6,7].

Pelvic CT Segmentation and Quantitative Anatomical Analysis

Sacroiliac Joints

SACROILIAC JOINTS ASSESSED: SUSPECTED SIJ DIASTASIS ABSOLUTE AND ASYMMETRIC; P95 side difference axial 6.7 mm / coronal 5.9 mm; width narrower than age reference/wider than age reference.

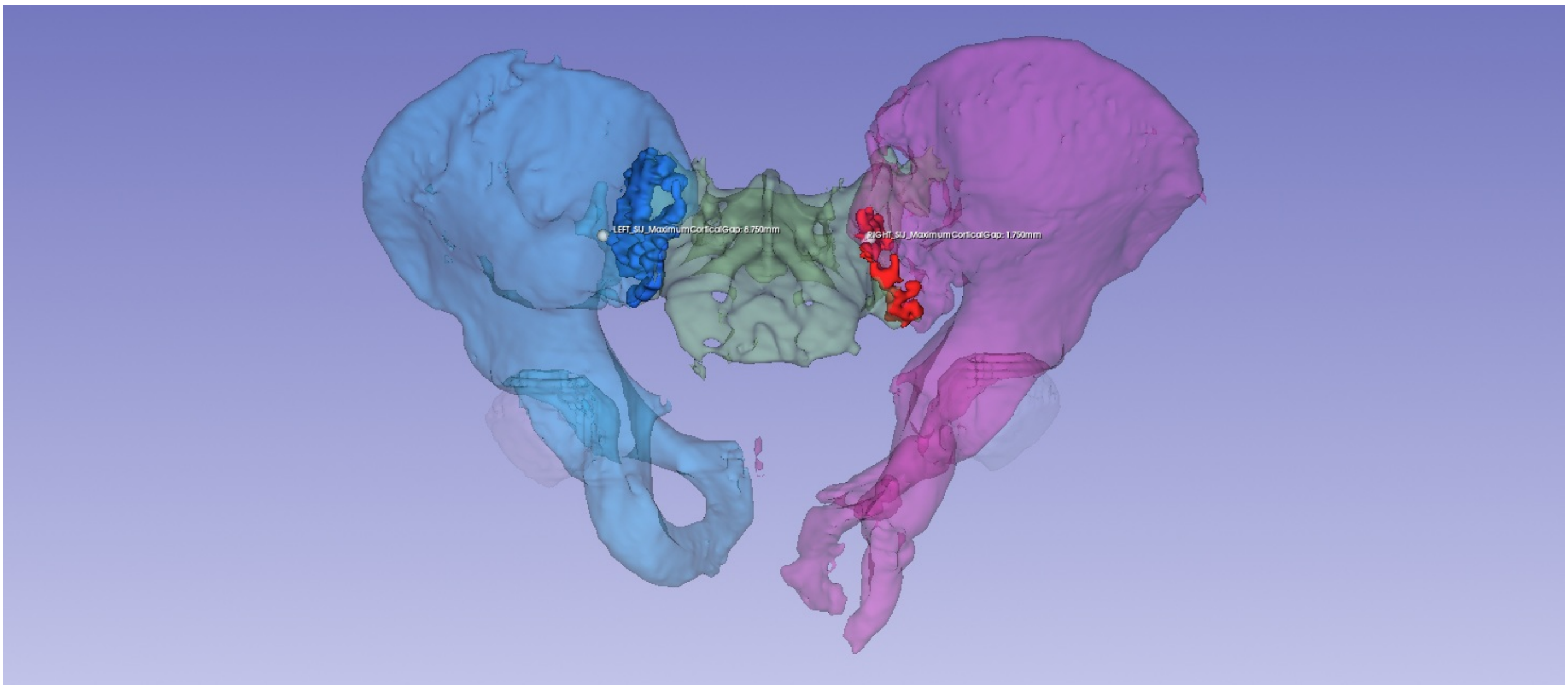

MEASUREMENT	RESULT	REFERENCE / COMPARATOR	INTERPRETATION
Right median cortical gap	**0.2 mm**	Published CT reference: 2.3 +/- 0.4 mm for patients <40 y and 1.9 +/- 0.2 mm for patients >40 y; patient age not supplied [8]	NARROWER THAN AGE REFERENCE
Left median cortical gap	**4.0 mm**	Published CT reference: 2.3 +/- 0.4 mm for patients <40 y and 1.9 +/- 0.2 mm for patients >40 y; patient age not supplied [8]	WIDER THAN AGE REFERENCE
Axial / coronal side difference	**6.7 mm / 5.9 mm**	Study-specific open-reduction context: 3.39 / 3.71 mm; absolute 6.52 / 7.26 mm [9]	SUSPECTED SIJ DIASTASIS ABSOLUTE AND ASYMMETRIC

Opposing sacral and iliac cortices localize the SI joint, and original CT is sampled along local normals. Published normal widths and trauma cut-points are comparators because the automated 3-D construction differs from manual MPR measurements [8,9].

Pelvic CT Segmentation and Quantitative Anatomical Analysis

Final Innominate Partition

FINAL INNOMINATE PARTITION: EXACT BILATERAL RECONSTRUCTION. Current method: ROOT10 single-plane pubis/ ischium split.

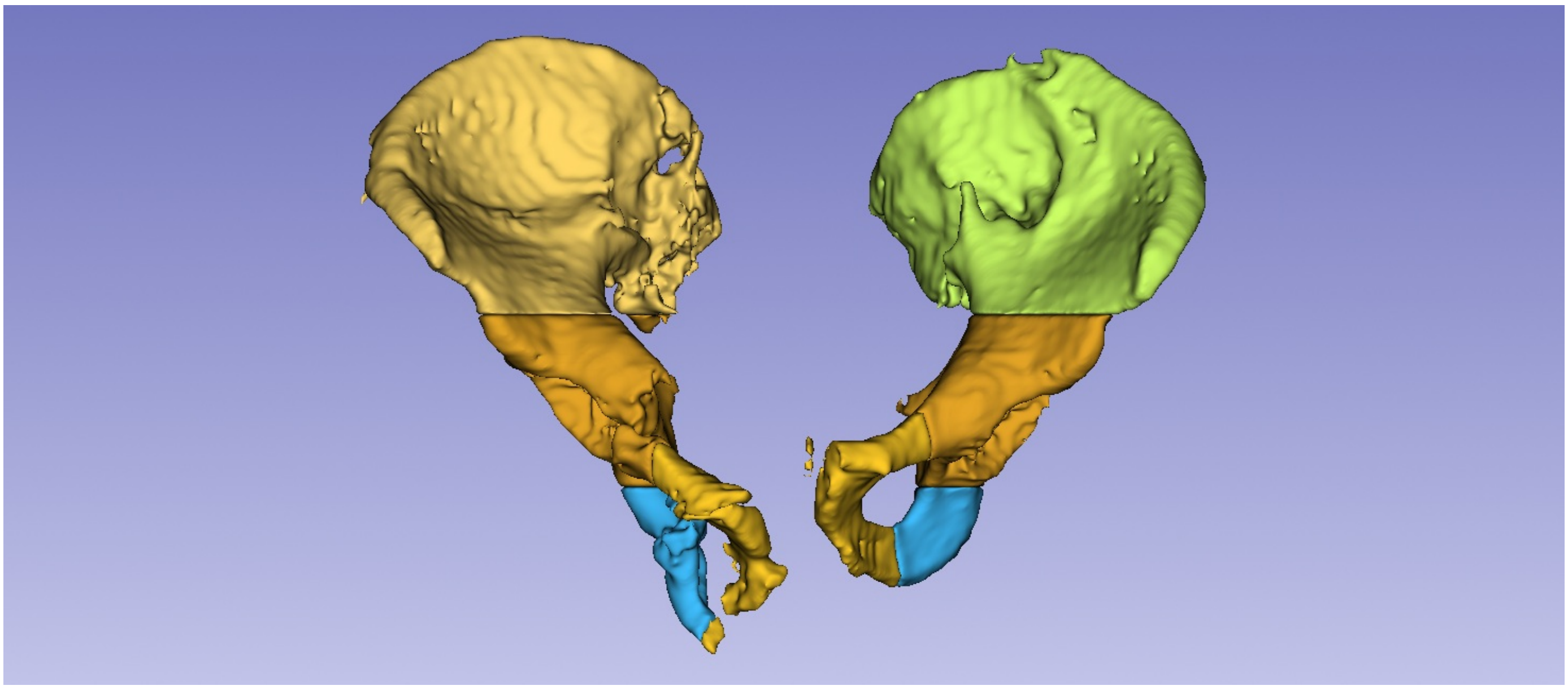

MEASUREMENT	RESULT	REFERENCE / COMPARATOR	INTERPRETATION
Right reconstruction	**PASS**	Technical requirement: zero overlap/unassigned/ outside voxels.	ROOT10 single-plane pubis/ ischium split
Left reconstruction	**PASS**	Technical requirement: zero overlap/unassigned/ outside voxels.	ROOT10 single-plane pubis/ ischium split

The innominate is reconstructed as mutually exclusive acetabulum, ilium, pubis, and ischium. Zero overlap, unassigned, and outside voxels are technical consistency requirements.

Pelvic CT Segmentation and Quantitative Anatomical Analysis

Technical Measurements - Page 1

**ROUTE / QUALITY CONTROL**	
Report status	VALID
Initial pathway	NORMAL
Final state	NATIVE MILD DEFECT
Midsagittal-plane QC	PASS — PLANE AVAILABLE; CENTRAL-BONE ASYMMETRY IS REPORT-ONLY
Route notes	Registered mirror-based abnormal reconstruction outputs are not applicable to this route.
**1. FEMORAL-HEAD MORPHOLOGY AND CENTRE - TECHNICAL MEASUREMENTS**	
**RIGHT**	
Centre confidence	HIGH
Fitted radius	22.69 mm
Sphere RMS	0.72 mm
Normalized RMS	3.18%
P95 residual	1.45 mm
Meaningful components	1
**LEFT**	
Centre confidence	HIGH
Fitted radius	22.19 mm
Sphere RMS	0.94 mm
Normalized RMS	4.24%
P95 residual	1.93 mm
Meaningful components	1
**BILATERAL QC**	
Radius difference	0.50 mm
Radius difference	2.22%
Bilateral radius QC	PASS
**2. NATIVE HEAD-SOCKET SHELL AND CONGRUITY - TECHNICAL MEASUREMENTS**	
**RIGHT**	
Margin status	CONTINUOUS
Structure class	INTACT CONTACT MARGIN
Local congruity	CONGRUENT
Full-head coverage	43.84%
Superior-head coverage	61.87%
Shell area	4395.70 mm²
Meaningful components	1
Median head-to-shell gap	2.39 mm
P95 head-to-shell gap	4.90 mm
**LEFT**	
Margin status	CONTINUOUS
Structure class	INTACT CONTACT MARGIN
Local congruity	CONGRUENT
Full-head coverage	44.96%

Pelvic CT Segmentation and Quantitative Anatomical Analysis

Technical Measurements - Page 2

Superior-head coverage	62.53%
Shell area	4615.53 mm²
Meaningful components	1
Median head-to-shell gap	2.04 mm
P95 head-to-shell gap	6.14 mm
**3. MIDSAGITTAL PLANE AND CENTRAL-BONE QC - TECHNICAL MEASUREMENTS**	
Overall plane QC	PASS — PLANE AVAILABLE; CENTRAL-BONE ASYMMETRY IS REPORT-ONLY
**L5**	
RIGHT volume	25771.76 mm3
LEFT volume	31044.50 mm3
Bilateral difference	18.56%
Smaller/larger ratio	0.8302
Symmetry QC	NOT NEAR-EQUAL
**Sacrum**	
RIGHT volume	63093.26 mm3
LEFT volume	79358.94 mm3
Bilateral difference	22.84%
Smaller/larger ratio	0.7950
Symmetry QC	NOT NEAR-EQUAL
**5. NATIVE ORIGINAL-CT ACETABULAR DISCONTINUITY — DECISIVE RAY TEST - TECHNICAL MEASUREMENTS**	
Accepted cluster total	1
Maximum radial opening	7.43 mm
Complete no-hit cluster	False
Radial opening routing gate	10.00 mm
Ray route	NATIVE MILD DEFECT
Routing basis	Maximum native radial opening 7.43 mm is < 10.00 mm.
**RIGHT**	
Internal rays tested	1935
CT-supported internal rays	1931
Raw internal candidates	4
Accepted defect rays	0
Accepted defect clusters	0
Maximum side radial opening	**NA**
Complete no-hit cluster	False
Defect status	NO INTERNAL ACETABULAR DISCONTINUITY DETECTED
**LEFT**	
Internal rays tested	1993
CT-supported internal rays	1982
Raw internal candidates	6
Accepted defect rays	3
Accepted defect clusters	1
Maximum side radial opening	7.43 mm

Pelvic CT Segmentation and Quantitative Anatomical Analysis

Technical Measurements - Page 3

Complete no-hit cluster	False
Defect status	INTERNAL ACETABULAR DISCONTINUITY CANDIDATE
Cluster 1 location	POSTERIOR
Cluster 1 rays	3
Cluster 1 3-D span	4.17 mm
Cluster 1 radial opening	7.43 mm
Cluster 1 class	MODERATE WITH PARTIAL NO-HIT GAP
**PUBIC SYMPHYSIS - TECHNICAL MEASUREMENTS**	
Clinical status	NORMAL
Screening classification	NO CT EVIDENCE OF SYMPHYSEAL DIASTASIS
Stable narrowest gap	6.00 mm
Selected pair gap	5.94 mm
Three-dimensional point distance	6.61 mm
AP shear	2.74 mm
SI shear	-1.00 mm
Supporting local cells	25
Local gap IQR	0.56 mm
**OBTURATOR RING - TECHNICAL MEASUREMENTS**	
**RIGHT**	
AP extent	69.95 mm
SI extent	81.39 mm
Area	2534.45 mm^2
Perimeter	256.03 mm
Tracked rim	64.44%
Tracked rim outside 4-mm band	79.74%
Suspected discontinuities	2
Defect 1 bridge chord	26.72 mm
Defect 1 estimated missing rim arc	6.77 mm
Defect 2 bridge chord	79.26 mm
Defect 2 estimated missing rim arc	79.88 mm
**LEFT**	
AP extent	47.00 mm
SI extent	32.57 mm
Area	1507.32 mm^2
Perimeter	152.59 mm
Tracked rim	100.00%
Tracked rim outside 4-mm band	76.67%
Suspected discontinuities	0
**SACROILIAC JOINTS - TECHNICAL MEASUREMENTS**	
Width classification	NARROWER THAN AGE REFERENCE
Median cortical width	0.25 mm
P95 cortical width	0.25 mm

Pelvic CT Segmentation and Quantitative Anatomical Analysis

Technical Measurements - Page 4

Maximum cortical width	1.75 mm
P95 axial projection	0.25 mm
P95 coronal projection	0.25 mm
CT-validated rays	356
Validation fraction	1.000
Bilateral trauma screen	SUSPECTED SIJ DIASTASIS ABSOLUTE AND ASYMMETRIC
Width classification	WIDER THAN AGE REFERENCE
Median cortical width	4.00 mm
P95 cortical width	7.00 mm
Maximum cortical width	8.75 mm
P95 axial projection	6.97 mm
P95 coronal projection	6.11 mm
CT-validated rays	855
Validation fraction	1.000
Bilateral trauma screen	SUSPECTED SIJ DIASTASIS ABSOLUTE AND ASYMMETRIC
**FINAL INNOMINATE PARTITION - TECHNICAL MEASUREMENTS**	
**RIGHT**	
Exact reconstruction	True
Unassigned voxels	0
Outside voxels	0
Overlap voxels	0
Acetabulum voxels	145579
Ilium voxels	287490
Pubis voxels	24986
Ischium voxels	30544
**LEFT**	
Exact reconstruction	True
Unassigned voxels	0
Outside voxels	0
Overlap voxels	0
Acetabulum voxels	155267
Ilium voxels	309947
Pubis voxels	31348
Ischium voxels	28844
**CURRENT PARTITION METHOD**	
Method	ROOT10 single-plane pubis/ischium split
Split mode	BOX ANCHOR FINITE20 ROOT CORRIDOR ILIUM FLOOR
Obturator used for split	False
Mirroring used for split	False
8-mm reclamation	False

Pelvic CT Segmentation and Quantitative Anatomical Analysis

References

Published comparators are interpreted according to their original measurement definitions; where the automated measurement differs, comparison is descriptive rather than a diagnostic cutoff.

[1] Hwang K, Wu X, Park CY. Width of pubic symphysis relating to age and sex in Koreans. J Orthop Surg Res. 2021;16(1):430. doi:10.1186/s13018-021-02561-9. PMID:34217332.

[2] Doro CJ, Forward DP, Kim H, Nascone JW, Sciadini MF, Hsieh AH, et al. Does 2.5 cm of symphyseal widening differentiate anteroposterior compression I from anteroposterior compression II pelvic ring injuries? J Orthop Trauma. 2010;24(10):610-615. doi:10.1097/BOT.0b013e3181cff42c. PMID:20871248.

[3] Gansslen A, Lindahl J, Krappinger D, Lindtner RA, Staresinic M. The myth of 2.5 cm symphyseal diastasis. Arch Orthop Trauma Surg. 2025;145(1):306. doi:10.1007/s00402-025-05904-x. PMID:40397156.

[4] Garras DN, Carothers JT, Olson SA. Single-leg-stance (flamingo) radiographs to assess pelvic instability: how much motion is normal? J Bone Joint Surg Am. 2008;90(10):2114-2118. doi:10.2106/JBJS.G.00277. PMID:18829908.

[5] Larson CM, Moreau-Gaudry A, Kelly BT, Byrd JWT, Tonetti J, Lavallee S, et al. Are normal hips being labeled as pathologic? A CT-based method for defining normal acetabular coverage. Clin Orthop Relat Res. 2015;473(4):1247-1254. doi:10.1007/s11999-014-4055-2. PMID:25407391.

[6] Zhang F, Zhang D, Huang Z, Wang Z, Cai X. Morphological asymmetry of pelvic rings: a study based on three-dimensional deviation analysis. Orthop Surg. 2022;14(5):967-976. doi:10.1111/os.13246. PMID:35377554.

[7] Duygu OB, Govsa F. Anatomical structures of the obturator foramen and its importance for pelvic interventions. Surg Radiol Anat. 2025;47(1):163. doi:10.1007/s00276-025-03661-5. PMID:40544414.

[8] Faflia CP, Prassopoulos PK, Daskalogiannaki ME, Gourtsoyiannis NC. Variation in the appearance of the normal sacroiliac joint on pelvic CT. Clin Radiol. 1998;53(10):742-746. doi:10.1016/S0009-9260(98)80316-4. PMID:9817091.

[9] Chien RS, Chen IJ, Lai CY, Chen JP, Yu YH. Critical distance of the sacroiliac joint for open reduction using screw fixation for traumatic sacroiliac joint diastasis: a retrospective study. J Orthop Surg Res. 2024;19(1):268. doi:10.1186/s13018-024-04759-z. PMID:38678298.

[10] Jamali AA, Mak W, Wang P, Tai L, Meehan JP, Lamba R. What is normal femoral head/neck anatomy? An analysis of radial CT reconstructions in adolescents. Clin Orthop Relat Res. 2013;471(11):3581-3587. doi:10.1007/s11999-013-3166-5. PMID:23922189.

[11] Hassan MM, Feroe AG, Douglass BW, Jimenez AE, Kuhns B, Mitchell CF, et al. Three-dimensional analysis of age and sex differences in femoral head asphericity in asymptomatic hips in the United States. World J Orthop. 2024;15(8):754-763. doi:10.5312/wjo.v15.i8.754. PMID:39165879.

[12] Rivas DJL, Gassmann JM, Goetz JE, Aitken HD, Davison JC, Miller A, et al. Weight-bearing CT as an approach to assess femoral-acetabular displacement during external rotation stress in the hip. J Hip Preserv Surg. 2025;12(2):93-104. doi:10.1093/jhps/hnaf001. PMID:40761572.

[13] Judet R, Judet J, Letournel E. Fractures of the acetabulum: classification and surgical approaches for open reduction. Preliminary report. J Bone Joint Surg Am. 1964;46:1615-1646. doi:10.2106/00004623-196446080-00001. PMID:14239854.

[14] Imani M, Buratto J, Dao T, Meijering E, Vogrin S, Kwok TCY, et al. Deep learning technique for automatic segmentation of proximal hip musculoskeletal tissues from CT scan images: a MrOS study. J Cachexia Sarcopenia Muscle. 2025;16(2):e13728. doi:10.1002/jcsm.13728. PMID:40022453.

[15] Ead MS, Duke KK, Jaremko JL, Westover L. Investigation of pelvic symmetry using CAD software. Med Biol Eng Comput. 2020;58(1):75-82. doi:10.1007/s11517-019-02068-w. PMID:31745838.

[16] Amar E, Druckmann I, Flusser G, Safran MR, Salai M, Rath E. The anterior inferior iliac spine: size, position, and location. An anthropometric and sex survey. Arthroscopy. 2013;29(5):874-881. doi:10.1016/j.arthro.2013.01.023. PMID:23523127.

[17] Rana SH, Farjoodi P, Haloman S, Dutton P, Hariri A, Ward SR, et al. Anatomic evaluation of the sacroiliac joint: a radiographic study with implications for procedures. Pain Physician. 2015;18(6):583-592. PMID:26606010.

[18] Hartel MJ, Petersik A, Schmidt A, Kendoff D, Nuchtern J, Rueger JM, et al. Determination of femoral neck angle and torsion angle utilizing a novel three-dimensional modeling and analytical technology based on CT datasets. PLoS One. 2016;11(3):e0149480. doi:10.1371/journal.pone.0149480.

[19] Mahmood SS, Mukka SS, Crnalic S, Wretenberg P, Sayed-Noor AS. Association between changes in global femoral offset after total hip arthroplasty and function, quality of life, and abductor muscle strength. Acta Orthop. 2016;87(1):36-41. doi:10.3109/17453674.2015.1091955.

[20] O'Brien S, Kernohan G. Perception of imposed leg length inequality in normal subjects. Hip Int. 2010;20(4):505-511. doi:10.1177/112070001002000414.

[21] Wang G, et al. Measurement of relative position of femoral head center, greater trochanter, and lesser trochanter. Ann Palliat Med. 2021;10(11):11524-11528. doi:10.21037/apm-21-2538.
