# Supplementary material for: Anatomy-driven automated subsegmentation of pelvic and proximal femoral CT into clinically relevant subregions and landmarks
Source: BMC Med Imaging. 2026 Sep 8;26:454. doi: 10.1186/s12880-026-02753-x (PMC13584298; doi:10.1186/s12880-026-02753-x)
Supplement: Supplementary file 2 [file 12880_2026_2753_MOESM1_ESM.docx]

**Supplementary Methods: Anatomy-Based Pelvic CT Subsegmentation Workflow**

The segmentation workflow was implemented as a structured multistage pipeline within a custom 3D Slicer environment, enabling dedicated scripted Python modules for automated pelvic CT processing. The workflow began with manual screening by an experienced orthopedic surgeon to exclude cases with morphological abnormalities, fractures, implants, major imaging artifacts, or incomplete anatomical coverage, with eligible scans required to include the full pelvis and proximal femora from at least the L5 level superiorly to below the lesser trochanters inferiorly. The eligible cases then underwent preprocessing with Pelvis Batch Manager, a scripted batch module that applied TotalSegmentator to detect the major parent bony structures required for downstream processing, including L5, the sacrum, both hemipelves, and both femora.

The PBM-derived masks were then used in the automated inclusion-exclusion quality-control step, in which cases were classified according to predefined thresholds for voxel spacing, pelvic positioning, and bone quality (in-plane voxel size >1.0 mm, slice spacing >1.25 mm, coronal tilt >5°, axial rotation >5°, marked left-right hemipelvis volume asymmetry, and L5 mean trabecular attenuation <160 HU) (Figure 1).

Figure 1. Pelvic CT eligibility and quality-control screening. Representative CT examples show excluded cases and an eligible normal case after assessment of coverage, resolution, bone density, pelvic orientation, and morphology.

An end-to-end segmentation pipeline for the pelvis and proximal femur was implemented as a custom 3D Slicer module (SegmentAll). Because sacral output from the initial TotalSegmentator pass was inadequate in most cases, the CT volume was cropped to the relevant pelvic region, and the sacrum was subsequently redefined from the remaining osseous voxels not already assigned to the other masked parent structures (Figure 2).

Figure 2. Sacral mask refinement after initial parent-structure detection. The sacrum was redefined from remaining pelvic osseous voxels after masking the other parent structures.

**Acetabular segmentation**

Femoral head detection was based on study-defined best axial and coronal proximal femoral core masks, from which the most representative circular contour was identified in perpendicular planes. This best-fit circle then served as the geometric reference for robust localization and extraction of the femoral head mask (Figure 3).

Figure 3. Femoral-head detection using best-fit circular core masks. Axial and coronal proximal femoral core masks were used to identify a representative circle for femoral-head extraction.

The acetabulum was localized by scaling the search geometry to the femoral head, using the detected head center and radius to define a proportionate confinement volume. True acetabular extraction was then restricted to the corresponding hemipelvis osseous voxels with femoral exclusion, converting detection into anatomically constrained geometric inference.

The acetabular roof, defined as the superior weight-bearing acetabular region, was then extracted from the isolated true acetabular mask after femoral exclusion, with roof isolation constrained to the anatomically superior acetabular component above the true superior extent of the femoral head [1] (Figure 4).

Figure 4. True acetabular extraction and acetabular roof isolation. Acetabular extraction was constrained by femoral-head geometry, hemipelvis voxels, femoral exclusion, and superior roof localization.

Further acetabular subsegmentation was performed by transforming the isolated true acetabular mask into a standardized roof-centered geometric reference frame, defined by a reproducible mid-roof point and orthogonal anterior-posterior and medial-lateral axes; within this frame, voxels were assigned according to their true positional relationship to the intersecting axes, thereby enabling anatomically oriented partitioning rather than arbitrary shape-based division.

Within this roof-centered frame, the remaining non-roof acetabular volume was partitioned into anatomically oriented sectors. The anterior sector was retained as Anterior Wall (Anterior Column footprint) and the posterior sector as Posterior Wall (Posterior Column footprint), corresponding to the anterior and posterior rim-based acetabular components, respectively [1,2]

The medial sector was retained as the Quadrilateral Plate (floor segment), corresponding to the relatively flat medial acetabular surface forming the acetabular floor directly medial to the socket [3,4] (Figure 5).

Figure 5. Roof-centered acetabular wall and quadrilateral-plate partitioning. The isolated acetabulum was partitioned in a roof-centered frame into anterior wall, posterior wall, and quadrilateral plate components.

**Landmarks** were detected as study-specific extreme-point or prominence-based reference anchors derived directly from the relevant parent masks and selected to match their anatomical descriptions, with the symphysis pubis additionally defined as the most anterior midline point between the right and left hemipelves; together, these landmark detections provided anatomically meaningful guide points for standardized pelvic orientation (Figure 6).

Figure 6. Pelvic landmark detection for standardized orientation. Extreme-point and prominence-based landmarks provided guide points for reproducible pelvic orientation.

# Iliac, Columnar, and Pubic-Ischial Subsegmentation

The ilium was extracted as the superior osseous component of the hemipelvis above the acetabular level, after exclusion of the acetabular domain.

ASIS and PSIS were first identified from the iliac mask as the most anterior and most posterior iliac reference points, respectively. Upper_Ilium and Lower_Ilium were then defined as study-specific superior and inferior iliac subregions generated by an ASIS-PSIS-based dividing plane, anatomically aligned with the distinction between the superior iliac ala and the inferior iliac body [5]

The infracetabular region, representing the combined ischial and pubic subacetabular domain, was obtained by subtracting the acetabular and iliac masks from the hemipelvis mask.

Anatomically, the anterior column comprises the anterior half of the iliac crest, the iliac spines, the anterior half of the acetabulum, and the pubis, whereas the posterior column comprises the ischium, the ischial spine, the posterior half of the acetabulum, and the dense bone of the sciatic notch [6] (Figure 7).

Figure 7. Hemipelvic column split using crest-apex and mid-roof references.

The hemipelvis was therefore divided into anterior and posterior column domains by a plane extending between the iliac crest apex and the mid-roof point, and further resolved into anterior and posterior iliac, acetabular, and infracetabular subcomponents (Figure 8).

Figure 8.Iliac, acetabular, and infracetabular columnar domains. Anterior and posterior column definitions were mapped to iliac, acetabular, and infracetabular subcomponents..

The ilium was extracted as the superior osseous component of the hemipelvis above the acetabular level after exclusion of the acetabular domain. ASIS and PSIS were first identified from the iliac mask as the most anterior and most posterior iliac reference points, respectively. Upper_Ilium and Lower_Ilium were then defined as study-specific superior and inferior iliac subregions generated by an ASIS–PSIS-based dividing plane, anatomically aligned with the distinction between the superior iliac ala and the inferior iliac body [5].

Sciatic buttress was defined as the thick and compact osseous structure formed where the acetabular columns coalesce in the supra-acetabular region and course posteriorly, transmitting load from the axial skeleton via the sacroiliac joint; in the pipeline, it was segmented from the overlap of Lower_Ilium and Posterior_Column_Ilium after exclusion of the oblique supra-acetabular reference line projected from the mid-roof point along the acetabular roof anterior-posterior axis with cranial angulation until contact with the upper ilium [7,8] (Figure 9).

Figure 9. Iliac subdivision and sciatic-buttress identification.The ilium is divided into upper and lower regions aligned with the superior iliac ala and inferior iliac body, with the sciatic buttress identified within the supra-acetabular load-transfer region.

The final pubic-ischial split defined the extra-acetabular pubic and ischial segments by dividing the residual infracetabular domain into the inferior pubic ramus, ischial spine, and ischial body, after which the anterior pubic component was merged with the inferior pubic ramus to form the pubis, while the ischial body was retained as the ischium [5] (Figure 10).

# Figure 10. Final pelvic component map after pubis–ischium separation.The infracetabular domain is resolved into distinct pubic and ischial components within the final pelvic subsegmentation tree.

# Proximal Femoral Subsegmentation

The proximal femur comprises the femoral head, femoral neck, and the greater and lesser trochanters [9]

In the pipeline, the distal limit of the upper proximal femoral working domain was anchored to a computed lesser-trochanter level. The primary lesser estimate was defined as the most prominent posteromedial landmark used to localize the lesser-trochanter level, and when that estimate was unreliable because of femoral malrotation, it was corrected using the published relation between lesser-trochanter-to-head-center distance and femoral head diameter; Wang et al. reported that "The L/D ratio was 0.84 ± 0.08." [10]

The final split was then performed from the corrected lesser-trochanter level, and the subtrochanteric region was defined as the proximal femoral segment extending 5 cm distal to the lesser trochanter [11]

The proximal femur was further divided by the intertrochanteric line into a head-neck region and an intertrochanteric region; in the pipeline, this boundary was defined as the straight line connecting the most medial point of the lesser trochanter to the most superior point of the greater trochanter [12]

The intertrochanteric region was then defined in the pipeline as the residual proximal femoral compartment after subtraction of the femoral head and the segmented femoral neck [13]

The femoral neck was defined as the narrowed osseous bridge connecting the femoral head to the femoral shaft, and in the pipeline was identified as the non-head component of the segmented head-neck region after subtraction of the femoral head [14]

The greater trochanter was defined as the lateral proximal femoral prominence, and in the pipeline was derived from the lateral compartment of the proximal femoral mask after exclusion of the femoral head or ball mask and retention of the dominant connected component [13]

The calcar was defined as the inferomedial cortical buttress at the femoral neck-shaft junction; in the pipeline, its study-specific lower inferomedial segment was derived by merging the three medial partitions of the proximal femoral working mask, thereby isolating the inferomedial proximal femoral compartment [15] (Figure 11).

Figure 11. Proximal femoral subsegmentation. The proximal femur was subdivided into head, neck, intertrochanteric, subtrochanteric, trochanteric, and calcar-related compartments.

# Sacral Zonal Subdivision

The sacrum was divided into right and left halves along its mediolateral extent to permit side-specific zonal subdivision [16]

The zonal framework followed the Denis concept, with Zone I lateral to the neural foramina, Zone II transforaminal, and Zone III medial to the foramina and involving the central sacral canal [16]

To simulate this arrangement, each sacral half was partitioned in the pipeline into three study-specific mediolateral compartments: a lateral 40% segment, a middle 40% segment, and a medial terminal 20% segment.

This proportional model showed near-complete agreement across the 100 validation cases but remains a study-derived geometric approximation that requires full-dataset morphometric analysis to define more precise zonal proportions (Figure 12).

Figure 12. Sacral zonal subdivision. Each sacral half was partitioned into study-specific mediolateral compartments approximating the Denis zonal framework.

# References

**1.** Lawrence DA, Menn K, Baumgaertner M, Haims AH. Acetabular fractures: anatomic and clinical considerations. American Journal of Roentgenology. 2013;201(3):W425-W436. doi:10.2214/AJR.12.10470.

**2.** Scheinfeld MH, Dym AA, Spektor M, Avery LL, Dym RJ, Amanatullah DF. Acetabular fractures: what radiologists should know and how 3D CT can aid classification. Radiographics. 2015;35(2):555-577. doi:10.1148/rg.352140098.

**3.** White G, Kanakaris NK, Faour O, Valverde JA, Martin MA, Giannoudis PV. Quadrilateral plate fractures of the acetabulum: an update. Injury. 2013;44(2):159-167. doi:10.1016/j.injury.2012.10.025.

**4.** ElNahal WA, Abdel Karim M, Khaled SA, Abdelazeem AH, Abdelazeem H. Quadrilateral plate fractures of the acetabulum: Proposition for a novel classification system. Injury. 2018;49(2):296-301. doi:10.1016/j.injury.2017.11.041.

**5.** Figueroa C, Jozsa F, Le PH. Anatomy, Bony Pelvis and Lower Limb: Pelvis Bones. In: StatPearls [Internet]. Treasure Island (FL): StatPearls Publishing; updated July 30, 2023.

**6.** Sharma SK, Mathur H. Surgical Anatomy of Acetabulum and Biomechanics. In: Suárez-Ahedo C, Olivos-Meza A, Rijke AM, editors. Essentials in Hip and Ankle. London: IntechOpen; 2020. doi:10.5772/intechopen.92330.

**7.** Dreizin D, LeBedis CA, Nascone JW. Imaging acetabular fractures. Radiologic Clinics of North America. 2019;57(4):823-841.

**8.** Durkee NJ, Jacobson J, Jamadar D, Karunakar MA, Morag Y, Hayes C. Classification of common acetabular fractures: radiographic and CT appearances. American Journal of Roentgenology. 2006;187(4):915-925. doi:10.2214/AJR.05.1269.

**9.** Agarwal J, Sharan A, Pant MK. The proximal end of the femur: exploring its clinical significance, implications in orthopedics. International Journal of Academic Medicine and Pharmacy. 2023;5(5):1175-1178. doi:10.47009/jamp.2023.5.5.231.

**10.** Wang G, Guo A, Zhang Y, Qiang H, Yu H, Diao N, Ma L, Zhang Y, Zhao E. Measurement of the relative position of the femoral head center, greater trochanter, and lesser trochanter in the proximal femur. Annals of Palliative Medicine. 2021;10(12):12889-12898. doi:10.21037/apm-21-3137.

**11.** Garrison I, Vosseller JT. Subtrochanteric femur fractures: current review of management. EFORT Open Reviews. 2021;6(2):145-151. doi:10.1302/2058-5241.6.200048.

**12.** Gupta M, Jindal P, Walia JPS, Kaur H. Morphometric analysis of the proximal femur with its clinical correlation in eastern Uttar Pradesh region. Cureus. 2022;14(10):e29868. doi:10.7759/cureus.29868.

**13.** Attum B, Pilson H. Intertrochanteric Femur Fracture. StatPearls [Internet]. Treasure Island (FL): StatPearls Publishing; 2026 Jan-. Last updated July 31, 2023.

**14.** Kazley JM, Bagchi K. Femoral Neck Fractures. StatPearls [Internet]. Treasure Island (FL): StatPearls Publishing; 2026 Jan-. Last updated May 8, 2023.

**15.** Stiles RG, Lavernia CJ, Resnick D, Convery FR. The calcar femorale. An anatomic, radiologic, and surgical study. Investigative Radiology. 1990;25(12):1311-1315.

**16.** Denis F, Davis S, Comfort T. Sacral fractures: an important problem. Retrospective analysis of 236 cases. Clinical Orthopaedics and Related Research. 1988;(227):67-81.
