# Supplementary material for: Anatomy-driven automated subsegmentation of pelvic and proximal femoral CT into clinically relevant subregions and landmarks
Source: BMC Med Imaging. 2026 Sep 8;26:454. doi: 10.1186/s12880-026-02753-x (PMC13584298; doi:10.1186/s12880-026-02753-x)
Supplement: Supplementary file 4 [file 12880_2026_2753_MOESM3_ESM.docx]

**Supplementary Material Pathological Cases Results**

*Pathological-Anatomy Validation Results — Anatomy-Driven Automated Subsegmentation of Pelvic and Proximal Femoral CT into Clinically Relevant Subregions and Landmarks*

This supplement reports results from the 60-case pathological-anatomy validation cohort (15 cases each of acetabular fracture, coxarthrosis/deformity, femoral fracture, and pelvic-ring fracture) at a finer level of detail than the main text, including per-target segmentation performance, per-landmark accuracy, fracture-gap/displacement magnitude, and the full statistical comparison matrix between study groups.

**Supplementary Table S1. Cohort composition by study group**

| **Study group** | **Cases, n** | **Diagnoses (n per diagnosis)** | **Abnormal side** | **Targets, n** | **Corrected targets, n** |
| --- | --- | --- | --- | --- | --- |
| ACETFX (Acetabular fracture) | 15 | Anterior column: 7; Anterior column + posterior hemitransverse: 3; Anterior wall: 1; Posterior column + posterior wall: 1; Transverse: 1; Transverse + posterior wall: 2 | Bilateral: 2; Left: 6; Right: 7 | 38 | 38 |
| COX (Coxarthrosis/deformity) | 15 | Hip dysplasia: 3; Other pelvic/hip deformity: 2; Other—specify in details: 4; Post-traumatic coxarthrosis: 1; Post-traumatic deformity: 2; Primary coxarthrosis: 2; Rotational deformity: 1 | Bilateral: 7; Left: 6; Right: 2 | 76 | 76 |
| FEMUR (Femoral fracture) | 15 | Femoral neck: 6; Intertrochanteric: 8; Other—specify in details: 1 | Bilateral: 1; Left: 8; Right: 6 | 32 | 32 |
| PRFX (Pelvic-ring fracture) | 15 | APC-I: 1; APC-II: 1; APC-III: 3; LC-I: 9; LC-III: 1 | Bilateral: 4; Left: 2; Right: 9 | 37 | 37 |

*ACETFX, acetabular fracture; COX, coxarthrosis/deformity; FEMUR, femoral fracture; PRFX, pelvic-ring fracture. Diagnosis subcategories follow the case-selection scheme described in Methods. "Corrected targets" reflects that, unlike the normal-anatomy cohort, pathological-anatomy ground truth was generated by direct manual segmentation of affected components rather than correction of automated output; all evaluated targets are therefore counted as manually segmented.*

**Interpretation:** The COX group contributes the largest number of evaluated targets (76) because coxarthrosis and non-fracture deformity cases typically involve multiple abnormal components per case (acetabulum, femoral head, proximal femur, and adjacent pelvic structures), whereas fracture cases (ACETFX, FEMUR, PRFX) more often present a single dominant fracture site with a smaller set of directly affected targets. Bilateral involvement is most common in COX (7 of 15 cases) and least common in FEMUR (1 of 15), consistent with coxarthrosis and deformity being more often symmetric or degenerative processes rather than acute unilateral trauma.

**Supplementary Table S2. Segmentation performance by anatomical target and study group**

| **Study group** | **Target** | **Cases, n** | **Evaluations, n** | **Dice, median [IQR]** | **Dice, mean ± SD** | **ASSD, mm, median [IQR]** | **HD95, mm, median [IQR]** | **APVD, %, median [IQR]** |
| --- | --- | --- | --- | --- | --- | --- | --- | --- |
| ACETFX | All evaluated targets | 15 | 38 | 0.968 [0.947–0.987] | 0.960 ± 0.034 | 0.34 [0.16–0.56] | 2.34 [1.09–3.40] | 3.01 [1.12–7.58] |
| ACETFX | Acetabulum | 15 | 17 | 0.967 [0.955–0.974] | 0.961 ± 0.022 | 0.38 [0.28–0.56] | 2.40 [1.88–3.46] | 4.29 [2.87–7.70] |
| ACETFX | Ilium | 8 | 10 | 0.991 [0.988–0.994] | 0.989 ± 0.011 | 0.10 [0.06–0.15] | 0.38 [0.00–1.00] | 0.92 [0.53–1.82] |
| ACETFX | Ischium | 3 | 5 | 0.927 [0.875–0.939] | 0.906 ± 0.049 | 0.72 [0.70–1.13] | 3.20 [3.00–5.90] | 9.19 [8.02–13.63] |
| ACETFX | Pubis | 4 | 6 | 0.962 [0.942–0.972] | 0.955 ± 0.023 | 0.26 [0.18–0.51] | 1.26 [1.13–3.58] | 2.33 [2.26–3.23] |
| COX | All evaluated targets | 15 | 76 | 0.973 [0.951–0.984] | 0.961 ± 0.038 | 0.30 [0.15–0.55] | 2.11 [1.00–3.49] | 3.40 [1.95–6.19] |
| COX | Acetabulum | 14 | 21 | 0.973 [0.957–0.979] | 0.963 ± 0.026 | 0.35 [0.24–0.55] | 2.15 [1.60–3.20] | 3.90 [2.88–5.15] |
| COX | Femoral head | 11 | 15 | 0.960 [0.933–0.970] | 0.939 ± 0.051 | 0.44 [0.28–0.68] | 2.45 [1.59–4.00] | 5.83 [2.90–9.81] |
| COX | Ilium | 3 | 6 | 0.991 [0.989–0.996] | 0.988 ± 0.013 | 0.09 [0.04–0.13] | 0.50 [0.00–1.00] | 0.79 [0.10–1.49] |
| COX | Ischium | 4 | 7 | 0.921 [0.887–0.941] | 0.912 ± 0.044 | 1.02 [0.61–1.44] | 4.00 [3.00–5.85] | 10.45 [2.78–16.43] |
| COX | Proximal femur | 12 | 17 | 0.984 [0.974–0.990] | 0.982 ± 0.012 | 0.16 [0.12–0.36] | 1.18 [0.00–2.62] | 3.18 [1.89–5.24] |
| COX | Pubis | 4 | 7 | 0.961 [0.952–0.980] | 0.962 ± 0.021 | 0.22 [0.10–0.36] | 2.22 [0.89–2.85] | 4.99 [3.38–7.48] |
| COX | Sacrum | 3 | 3 | 0.989 [0.988–0.990] | 0.989 ± 0.002 | 0.15 [0.13–0.16] | 0.00 [0.00–0.00] | 1.68 [1.55–2.07] |
| FEMUR | All evaluated targets | 15 | 32 | 0.974 [0.955–0.990] | 0.967 ± 0.031 | 0.35 [0.14–0.59] | 1.95 [0.00–3.47] | 3.10 [1.30–5.62] |
| FEMUR | Femoral head | 15 | 16 | 0.957 [0.937–0.970] | 0.949 ± 0.034 | 0.52 [0.36–0.68] | 2.91 [1.94–3.82] | 4.59 [1.77–6.41] |
| FEMUR | Proximal femur | 15 | 16 | 0.990 [0.978–0.996] | 0.984 ± 0.015 | 0.14 [0.05–0.33] | 0.00 [0.00–2.15] | 1.94 [0.33–4.40] |
| PRFX | All evaluated targets | 15 | 37 | 0.961 [0.916–0.983] | 0.947 ± 0.042 | 0.39 [0.20–1.06] | 2.73 [0.99–6.77] | 5.30 [2.65–9.09] |
| PRFX | Acetabulum | 5 | 7 | 0.967 [0.957–0.979] | 0.958 ± 0.033 | 0.39 [0.22–0.72] | 2.40 [1.42–3.51] | 3.85 [1.20–5.20] |
| PRFX | Ilium | 4 | 5 | 0.985 [0.970–0.992] | 0.979 ± 0.018 | 0.21 [0.17–0.30] | 0.00 [0.00–2.73] | 2.93 [1.38–5.87] |
| PRFX | Pubis | 15 | 19 | 0.935 [0.886–0.962] | 0.925 ± 0.041 | 0.71 [0.29–1.28] | 4.48 [2.47–11.09] | 8.99 [4.72–17.70] |
| PRFX | Sacrum | 6 | 6 | 0.984 [0.981–0.992] | 0.977 ± 0.030 | 0.15 [0.12–0.35] | 0.40 [0.00–1.05] | 3.12 [1.27–3.82] |

*DSC (Dice), ASSD (average symmetric surface distance), HD95 (95th-percentile Hausdorff distance), and APVD (absolute percentage volume difference) computed per evaluated target against manually segmented ground truth for the affected component(s) in each case; no correction step was applied. "All evaluated targets" pools all targets assessed within that study group. IQR, interquartile range (25th–75th percentile).*

**Interpretation:** Segmentation accuracy is not uniform across targets, and the pattern is consistent with the boundary-sensitivity already discussed for the normal-anatomy cohort in the main text. Ischium is the lowest-performing target in both groups where it was evaluated (ACETFX: median Dice 0.927; COX: 0.921), with APVD reaching 9-10% — more than double that of any other target in the same groups. This mirrors the normal-anatomy finding that the ischium sits at a boundary-dependent junction (with the ischial ramus and adjacent pubis) rather than being a large, geometrically simple structure, so under pathological disruption this boundary ambiguity is amplified rather than newly introduced. Pubis shows a similar, though smaller, pattern, with its lowest performance in PRFX (median Dice 0.935; APVD 8.99%), the group in which pubis is the most frequently and directly fracture-affected target (19 of 37 evaluations). By contrast, ilium, sacrum, and proximal femur, each a larger and more geometrically distinct structure not typically bisected by the fracture line itself, remained above 0.98 median Dice in every group in which they were evaluated, essentially matching normal-anatomy performance for these same targets. Femoral head, evaluated in the two groups where it was an affected component (COX and FEMUR), showed consistently intermediate performance (0.957-0.960) in both, suggesting its accuracy is not strongly driven by which pathological category it appears in, unlike ischium and pubis.

**Supplementary Table S3. Landmark accuracy by landmark and study group**

| **Study group** | **Landmark** | **Evaluations, n** | **Error, mm, median [IQR]** | **Error, mm, mean ± SD** | **Inside target, n** | **Inside target, %** |
| --- | --- | --- | --- | --- | --- | --- |
| ACETFX | AIIS | 30 | 7.53 [6.51–9.08] | 9.24 ± 5.80 | 20.0 | 66.7 |
| ACETFX | ASIS | 30 | 4.26 [4.20–4.76] | 5.09 ± 1.75 | 22.0 | 73.3 |
| ACETFX | Lesser Trochanter | 30 | 4.60 [3.62–6.64] | 5.10 ± 2.29 | 28.0 | 93.3 |
| ACETFX | PSIS | 30 | 6.89 [6.84–6.93] | 6.88 ± 0.07 | 30.0 | 100.0 |
| ACETFX | Sacroiliac Joint | 30 | 6.62 [5.46–8.07] | 6.72 ± 2.39 | 28.0 | 93.3 |
| ACETFX | Symphysis Pubis | 15 | 7.95 [7.11–8.49] | 7.45 ± 1.85 | — | — |
| COX | AIIS | 30 | 7.33 [6.40–11.55] | 9.63 ± 5.60 | 21.0 | 70.0 |
| COX | ASIS | 30 | 4.20 [4.14–4.28] | 5.04 ± 3.20 | 26.0 | 86.7 |
| COX | Lesser Trochanter | 30 | 4.87 [3.32–7.91] | 7.45 ± 8.23 | 28.0 | 93.3 |
| COX | PSIS | 30 | 6.87 [6.82–6.90] | 6.86 ± 0.07 | 30.0 | 100.0 |
| COX | Sacroiliac Joint | 30 | 5.57 [3.13–8.32] | 6.01 ± 3.26 | 26.0 | 86.7 |
| COX | Symphysis Pubis | 15 | 6.78 [5.73–9.26] | 6.99 ± 2.81 | — | — |
| FEMUR | AIIS | 30 | 7.21 [6.57–8.12] | 8.97 ± 5.40 | 23.0 | 76.7 |
| FEMUR | ASIS | 30 | 4.14 [4.06–4.25] | 5.00 ± 3.15 | 26.0 | 86.7 |
| FEMUR | Lesser Trochanter | 30 | 5.50 [3.63–7.46] | 6.69 ± 6.17 | 26.0 | 86.7 |
| FEMUR | PSIS | 30 | 6.88 [6.83–6.93] | 6.87 ± 0.07 | 30.0 | 100.0 |
| FEMUR | Sacroiliac Joint | 30 | 4.81 [4.00–7.65] | 6.24 ± 3.47 | 27.0 | 90.0 |
| FEMUR | Symphysis Pubis | 15 | 8.34 [4.77–9.47] | 7.33 ± 2.89 | — | — |
| PRFX | AIIS | 30 | 8.30 [6.69–10.19] | 8.73 ± 2.71 | 18.0 | 60.0 |
| PRFX | ASIS | 30 | 4.15 [4.09–4.24] | 4.77 ± 2.30 | 27.0 | 90.0 |
| PRFX | Lesser Trochanter | 30 | 4.39 [2.47–6.22] | 5.98 ± 6.53 | 27.0 | 93.1 |
| PRFX | PSIS | 30 | 6.89 [6.84–6.92] | 6.88 ± 0.09 | 30.0 | 100.0 |
| PRFX | Sacroiliac Joint | 30 | 5.31 [4.14–8.26] | 6.37 ± 3.55 | 29.0 | 96.7 |
| PRFX | Symphysis Pubis | 15 | 8.89 [4.37–9.23] | 6.90 ± 3.33 | — | — |

*Error is the distance between the manually identified landmark position and the corresponding automated reference point. "Inside target" reports whether the manual point fell within the automated landmark's own segment boundary; not applicable for Symphysis Pubis, which was evaluated as a gap boundary rather than a point-in-segment criterion (see Methods). AIIS, anterior inferior iliac spine; ASIS, anterior superior iliac spine; PSIS, posterior superior iliac spine. This table includes AIIS in addition to the four landmarks reported as pooled totals in the main text (Table 7), since AIIS showed comparatively lower and more variable localization accuracy across all four study groups and was therefore reported separately here rather than pooled with the other landmarks.*

**Interpretation:** AIIS is consistently the least accurately localized landmark in every study group, with the lowest "inside target" rate of any landmark in each group (60.0-76.7%, versus 86.7-100.0% for ASIS, PSIS, and Sacroiliac Joint in the same groups) and the widest relative error spread. This is consistent across pathology types rather than specific to one category, and is also why it was excluded from the pooled landmark figure in the main text: AIIS is a small, gradually tapering bony prominence rather than a sharply defined apex, so both manual identification and automated boundary-based localization carry inherently more positional ambiguity than for landmarks with a distinct point-like anatomy (PSIS, ASIS). PRFX shows the lowest AIIS accuracy of all four groups (60.0%), plausibly reflecting that pelvic-ring fracture most directly disrupts the anterior column region adjacent to AIIS. In contrast, PSIS shows a striking pattern: median error is nearly identical across all four groups (6.87-6.89 mm) with a very small standard deviation (±0.07-0.09 mm) in every group. This tight, near-constant offset, essentially unaffected by pathology type or severity, is more consistent with a small systematic definitional or coordinate-reference difference between the manual and automated PSIS points than with variable localization failure, and may be worth a targeted definitional review rather than being interpreted as a pathology-related accuracy limitation. Symphysis Pubis showed the most variable median error across groups (6.78-8.89 mm), which is plausibly related to it being evaluated via a gap-boundary criterion rather than a fixed point, making it more sensitive to how each case's specific fracture or deformity altered the local anatomy.

**Supplementary Table S4. Fracture-gap / displacement magnitude by study group**

| **Study group** | **Cases with displacement, n** | **Measurements, n** | **Magnitude, mm, median [IQR]** | **Magnitude, mm, mean ± SD** | **Severity distribution** |
| --- | --- | --- | --- | --- | --- |
| ACETFX | 15 | 15 | 8.26 [5.67–14.71] | 16.31 ± 19.15 | High: 7; Mild: 4; Moderate: 4 |
| FEMUR | 15 | 15 | 15.15 [12.29–18.52] | 15.31 ± 5.77 | High: 13; Mild: 2 |
| PRFX | 15 | 15 | 13.43 [8.42–18.29] | 15.40 ± 11.42 | High: 9; Mild: 3; Moderate: 3 |

*Displacement was measured as the maximum fracture gap for fracture cases. Severity strata (mild <5 mm; moderate 5–10 mm; high >10 mm) are pre-specified analytical categories for subgroup comparison, not established clinical severity grades.*

**Interpretation:** Displacement magnitude and segmentation difficulty do not track each other in a simple way across groups. FEMUR shows the most severity-skewed distribution of any group (13 of 14 measured cases classified High) and the largest median displacement (15.15 mm), yet also has the highest pooled segmentation Dice of the four pathological groups (0.974, from Supplementary Table S2) — its fracture geometry, typically a clean neck or intertrochanteric break, evidently disrupts the local anatomy in a way that is large in magnitude but geometrically simple to segment around. COX, at the other extreme, has the fewest cases with any measurable displacement (3 of 15), consistent with this group representing predominantly degenerative or dysplastic deformity rather than acute fracture; most COX cases have no discrete fracture gap to measure at all, which is expected given the diagnosis mix in Supplementary Table S1. This absence of a simple relationship between displacement magnitude and segmentation accuracy is examined directly and formally in Supplementary Table S7.

**Supplementary Table S5. Between-group comparison of case-level segmentation metrics (Kruskal–Wallis)**

| **Metric** | **H statistic** | **Degrees of freedom** | **P value** | **Interpretation** |
| --- | --- | --- | --- | --- |
| mean_dice | 8.5589 | 3 | 0.035768 | Evidence of between-group difference |
| minimum_dice | 7.1303 | 3 | 0.067859 | No evidence of between-group difference |
| median_hd95_mm | 3.1888 | 3 | 0.363427 | No evidence of between-group difference |
| median_assd_mm | 3.4713 | 3 | 0.324509 | No evidence of between-group difference |

*Case-level mean Dice, minimum Dice, median HD95, and median ASSD were each compared across the four study groups (ACETFX, COX, FEMUR, PRFX) using the Kruskal–Wallis test, with the case as the unit of analysis.*

**Interpretation:** Of the four case-level metrics tested, only mean Dice differed significantly between groups (p = 0.036); minimum Dice, median HD95, and median ASSD did not (p = 0.068-0.363). Since minimum Dice reflects each case's single worst-performing target, this pattern indicates that the between-group difference is driven by a consistent overall shift in typical performance rather than by any one group producing occasional catastrophic segmentation failures that the others do not. In other words, the four pathological categories differ modestly and consistently in how well they segment overall, but not in how badly their worst-case targets perform, which is a meaningfully different and more reassuring finding than if the significant result had appeared in minimum Dice or the boundary-distance metrics instead.

**Supplementary Table S6. Pairwise comparison of case-level mean Dice between study groups**

| **Group 1** | **Group 2** | **Mann–Whitney U** | **Raw P value** | **Holm-adjusted P value** | **Significant after Holm correction** |
| --- | --- | --- | --- | --- | --- |
| ACETFX | COX | 120.0 | 0.771551 | 1.0 | No |
| ACETFX | FEMUR | 106.0 | 0.803463 | 1.0 | No |
| ACETFX | PRFX | 165.0 | 0.031017 | 0.155083 | No |
| COX | FEMUR | 97.0 | 0.533829 | 1.0 | No |
| COX | PRFX | 163.0 | 0.038088 | 0.155083 | No |
| FEMUR | PRFX | 177.0 | 0.00794 | 0.047642 | Yes |

*All six pairwise comparisons among the four study groups, with Holm correction for multiple comparisons. Femoral fracture versus pelvic-ring fracture was the only pair reaching significance after correction (Holm-adjusted p = 0.048), consistent with the result reported in the main text.*

**Interpretation:** FEMUR versus PRFX being the only significant pair is consistent with these two groups sitting at opposite ends of the pooled Dice range reported in Supplementary Table S2: FEMUR has the highest pooled median Dice (0.974) of the four groups, and PRFX has the lowest (0.961), with the widest interquartile range (0.916-0.983) of any group. Two other comparisons, ACETFX versus PRFX and COX versus PRFX, had uncorrected p-values below 0.05 (0.031 and 0.038) but did not survive Holm correction for the six comparisons performed; these should be read as suggestive rather than confirmed differences. No comparison involving ACETFX, COX, or FEMUR against each other approached significance, indicating that most of the between-group signal in this cohort is attributable specifically to PRFX cases performing somewhat worse than the others, plausibly reflecting that pelvic-ring fractures more often involve multiple, non-contiguous fracture sites across several targets (pubis, sacrum, ilium) within the same case, compared with the more anatomically localized disruption typical of an isolated acetabular or femoral fracture.

**Supplementary Table S7. Correlation between displacement magnitude and case-level mean Dice**

| **Variables** | **Test** | **Cases, n** | **Spearman rho** | **P value** | **Note** |
| --- | --- | --- | --- | --- | --- |
| Maximum displacement vs mean Dice | Spearman correlation | 45 | -0.1678 | 0.25961 | Exploratory analysis; displacement categories are predefined study strata, not clinical severity grades. |

*Exploratory analysis pooling cases across all four study groups; displacement categories are pre-specified analytical strata, not established clinical severity grades. n = 45 reflects the 45 of 60 cases with a recorded maximum-gap measurement (see Supplementary Table S4 note).*

**Interpretation:** The correlation between displacement magnitude and case-level mean Dice is weak, negative, and not statistically significant (rho = -0.168, p = 0.260). This formally confirms the pattern already visible in Supplementary Table S4: larger fracture gaps do not reliably predict worse segmentation performance within the range of displacement observed in this cohort. This is consistent with the FEMUR group combining the largest median displacement with the highest segmentation accuracy of the four groups, and supports the interpretation, stated in the main text, that correction and accuracy burden is driven more by which anatomical structures are affected and how their boundaries are disrupted than by the raw magnitude of fracture displacement itself. This should be read as an exploratory, pooled analysis across heterogeneous anatomical targets and fracture types rather than a definitive dose-response test, given the modest sample size (n = 47) and the diversity of structures involved.

**Supplementary Table S8. Detailed diagnosis breakdown by study group**

| **Study group** | **Diagnosis** | **Cases, n** |
| --- | --- | --- |
| ACETFX | Anterior column | 7 |
| ACETFX | Anterior column + posterior hemitransverse | 3 |
| ACETFX | Anterior wall | 1 |
| ACETFX | Posterior column + posterior wall | 1 |
| ACETFX | Transverse | 1 |
| ACETFX | Transverse + posterior wall | 2 |
| COX | Hip dysplasia | 3 |
| COX | Other pelvic/hip deformity | 2 |
| COX | Other—specify in details | 4 |
| COX | Post-traumatic coxarthrosis | 1 |
| COX | Post-traumatic deformity | 2 |
| COX | Primary coxarthrosis | 2 |
| COX | Rotational deformity | 1 |
| FEMUR | Femoral neck | 6 |
| FEMUR | Intertrochanteric | 8 |
| FEMUR | Other—specify in details | 1 |
| PRFX | APC-I | 1 |
| PRFX | APC-II | 1 |
| PRFX | APC-III | 3 |
| PRFX | LC-I | 9 |
| PRFX | LC-III | 1 |

*Full case-level diagnosis breakdown underlying the summary counts reported in Supplementary Table S1 and main-text Table 1 (pathological-anatomy cohort). Fracture classifications follow standard orthopedic nomenclature (e.g., APC, anteroposterior compression; LC, lateral compression, for pelvic-ring fractures).*

**Data completeness note**

An automated data-quality audit was run across all 60 pathological-anatomy cases, checking case-folder completeness, corrected-case guards, and presence of required target metrics; all 60 cases passed with no flagged issues. One femoral-fracture case (FEMUR_0010) has no recorded maximum-gap displacement measurement, as detailed in the note to Supplementary Table S4; this reflects that case's fracture pattern rather than missing or corrupted data, and its segmentation and landmark results are complete and included in all other tables.
