# Supplementary material for: Anatomy-driven automated subsegmentation of pelvic and proximal femoral CT into clinically relevant subregions and landmarks
Source: BMC Med Imaging. 2026 Sep 8;26:454. doi: 10.1186/s12880-026-02753-x (PMC13584298; doi:10.1186/s12880-026-02753-x)
Supplement: Supplementary file 5 [file 12880_2026_2753_MOESM4_ESM.docx]

**Supplementary Methods: Pathological-Case Validation and Adaptive Segmentation Logic**

The pathological pathway is the validation and adaptation layer of the pipeline. For every case, it tests whether the segmented femoral head, acetabulum, proximal femur, pubic symphysis, sacroiliac joints, and obturator rings are geometrically and radiographically consistent with an intact, congruent pelvis. Where a region fails that test, the pipeline does not simply raise a flag and stop: it switches the underlying segmentation logic for that region from a fixed anatomical model to an adaptive, evidence-driven reconstruction that uses the patient's own uninvolved anatomy — the contralateral hip, the native but displaced bone fragments, and the original CT density — as the reference, so a final, anatomically labeled segmentation is still produced when the true anatomy is fractured, dislocated, or discontinuous. This dual role, abnormality flagging together with adaptive re-segmentation, is the core function of the pathological pathway. Every figure below is a real evidence-page screenshot extracted from one of five processed cases (PRFX_0010, PRFX_0006, FEMUR_0007, ACETFX_0013, ACETFX_0004), not a mockup; the right hemipelvis renders pink/magenta and the left renders blue throughout.

# **Targeted Output Segments**

The pathological pathway publishes the following segments, models, and measurement sets in addition to the standard anatomical segmentation:

| **Output** | **Description** |
| --- | --- |
| right_acetabulum / left_acetabulum | Final published acetabular segmentation per side: native head-anchored box on the reference side; box union adaptive fragment-recovery mask on the reconstructed side. |
| right_acetabular_box / left_acetabular_box | Native, head-center-anchored bounding geometry (3x/2x radius envelope, ASIS-calibrated), built identically and independently for both sides. |
| {side}_acetabulum_adaptive_native / _adaptive_recovered / _adaptive_fragment_NN / _adaptive_final | Individually labeled native-bone and recovered-fragment components produced by the adaptive fracture-recovery segmentation, plus their merged mask. |
| CHECK3B_{side}_Virtual_Expected_Socket_Center / _Registered_Expected_Acetabular_Shell | The simulated (mirrored and stable-bone registered) reference socket used as the expected-geometry comparator when the native socket is disrupted. |
| CHECK3B_{side}_Displacement_Vector / Actual_Head_Center | Femoral head-center position and its displacement vector relative to the registered expected socket. |
| OIP_FINAL_SOCKET_RAY_{side}_* / OIP_ABNORMAL_VIRTUAL_RAY_{side}_* | Supported and defect ray models from the native-CT and registered-socket discontinuity tests, marking confirmed defect clusters by location. |
| femur_parameters (per side) | Neck-shaft angle, femoral offset, limb-length discrepancy, and head-center-to-lesser-trochanter ratio, with an integrated flagged/not-flagged status. |
| Symphysis_Pubis_Gap / Narrowest_Point markups | Narrowest stable symphyseal gap, 3-D point distance, and AP/SI shear at the pubic symphysis. |
| {side}_SIJ_joint / {SIDE}_SIJ_MaximumCorticalGap | Sacroiliac joint cortical-gap profile and maximum gap per side, plus the bilateral trauma and vertical-shear screens. |
| OIP_Obturator_Rim_{side}_* / FourHitPoints_PerSide | 360-degree obturator rim contour, dimensions, and any suspected rim-discontinuity segments per side. |
| PelvisMetrics_LCEA_* / PelvisMetrics_AI_* / femoral-coverage models / Larson coverage report | Coverage and radiographic-analog outputs: global coverage, LCEA, OIP acetabular index, and DeFroda/Cheng femoral-head coverage. |

# **Core Concept: How the Segmentation Logic Changes From the Uncomplicated Case**

In an uncomplicated hip, each anatomical structure is extracted with a fixed geometric formula applied to the patient's own segmented anatomy. In a pathological case, that fixed formula breaks down wherever bone is missing, displaced, or discontinuous, so three structures switch to a fundamentally different, adaptive segmentation strategy.

## **Femoral-Head/Socket Simulation**

When the true acetabular socket on the affected side cannot be trusted as a geometric reference, the pipeline builds a simulated socket instead of segmenting the disrupted anatomy directly. A sphere is fit to the reference (unaffected) side's acetabular shell, reflected across the patient's own midsagittal plane, and then corrected by a rigid registration of the two sides' stable, uninvolved bone — deliberately excluding the fracture site from the registration so the injury itself cannot bias the prediction. The result is a patient-specific simulated socket center that behaves as the expected, pre-injury geometry: it is the fixed point every subsequent displacement, defect, and penetration measurement on the affected side is measured against, and it is what allows the pipeline to quantify "how far" and "which direction" the head has moved, rather than only recognizing that something is wrong.

**Figure 1.** Femoral-head/socket simulation and displacement quantification (Case ACETFX_0013). Left femoral-head displacement: 36.8 mm, posterior + inferior, CT-confirmed relative to the registered expected socket built from the mirrored and stable-bone-registered right acetabular shell.

## **Adaptive Acetabulum**

Where the femoral head is displaced or the local socket is not congruent, the acetabulum on the affected side is no longer built from a single fixed box. The pipeline instead identifies which parts of the simulated socket surface are not locally supported by native bone ("missing shell"), then searches two recovery zones — a corridor swept along the head's displacement vector, and a contact zone around the head's current position — for bone fragments that could plausibly have come from those missing regions. Each candidate fragment is tested by translating it back along the displacement vector and checking how much of the missing surface it would explain; fragments that pass are kept and merged with the native bone as individually labeled components. The final acetabulum on that side is therefore reconstructed, not merely bounded: displaced bone is reassigned to its inferred native position instead of being excluded or accepted as a single undifferentiated mass.

**Figure 2.** Adaptive acetabulum: CT-supported fragment-recovery reconstruction (Case ACETFX_0013). CT-supported socket reconstruction completed on the left side; one CT-matched displaced fragment candidate (green) was recovered and merged with native bone.

## **Obturator Foramen Ring-Disruption Detection**

The obturator ring is not segmented from a fixed template at all; it is tracked. The pipeline follows the inner obturator rim continuously through 360 degrees per side and compares the tracked contour to an expected envelope. Where the tracked rim departs from that envelope over a sustained arc, the ring is no longer treated as a single closed structure: the departure is reported as a suspected rim discontinuity, characterized by the gap it spans (bridge-chord length), the arc of rim it removes, and its angular position. Because the obturator ring is formed jointly by the inferior pubic ramus and the ischium, a break detected this way is a direct structural signal for ramus fracture even before the adjacent bone segments themselves are individually classified as abnormal — the ring-tracking method turns a continuity check into the pipeline's earliest anterior-pelvic-ring fracture detector.

**Figure 3.** Obturator foramen ring-disruption detection (Case PRFX_0010). Right obturator ring flagged: 2 rim discontinuity candidates tracked around the 360-degree contour, maximum bridge chord 79.3 mm; left ring shows no reportable discontinuity.

## **Route Selection Itself Is Adaptive**

The same principle extends to which reconstruction path a case takes at all. A case with a mild native-bone opening keeps its own native geometry with no mirroring; only a case with a marked or unmeasurable opening, or a confirmed unilateral coverage deficit, is escalated into the full simulate-and-recover chain above; and a case with bilateral or conflicting severe findings is deliberately held as indeterminate rather than forced through a reconstruction the pipeline cannot support with a clean reference side. The choice of segmentation strategy is therefore itself part of the pathology-detection logic, not a separate step downstream of it.

# **Flagged Abnormality-Detection Methods**

Every screening method in the pipeline, the anatomical region it covers, and the criterion that raises a flag. Real examples for every row are given in the illustrative figures that follow (Figures 4-16).

| **Region** | **Method** | **Flag criterion** | **Ref.** |
| --- | --- | --- | --- |
| Hip articulation | Check 1 — femoral-head sphere fit / center confidence | Implausible radius (outside 15-35 mm) or combined RMS >10% and P95 residual >5 mm -> LOW confidence / severe morphology caution (Fig. 4) | [10,11] |
| Hip articulation | Check 2 — head-corresponding acetabular margin / local congruity | Margin not continuous, or median gap >4 mm / P95 gap >7 mm -> BORDERLINE/PARTIAL/INCONGRUENT; coverage <15% (full) or <25% (superior) -> INCOMPLETE shell (Fig. 5) | - |
| Hip articulation | Pre-ray pathway routing | Full-head coverage <36% (Larson-derived), or Check-1/Check-2 structural failure -> side routed ABNORMAL (Fig. 15) | [5] |
| Hip articulation | Native-CT ray-discontinuity test | Clustered rays with no CT bone support or >=3 mm / 4-sigma local deviation -> defect cluster; graded mild (<=5 mm) / moderate (<10 mm) / marked (>=10 mm) (Fig. 6) | - |
| Hip articulation | Post-ray routing gate | Any cluster >=10 mm opening or complete no-hit -> ABNORMAL RECONSTRUCTION REQUIRED (Fig. 6) | - |
| Hip articulation | Head-socket displacement / congruity | Total displacement from simulated socket >3 mm -> DISPLACED (not congruent); direction reported only if >=3 mm and >=20% of largest component (Fig. 1, 5) | - |
| Hip articulation | Central / medial-wall penetration | Central-missing rays >10%, central-displaced >20%, or wall shift >3 mm -> wall disrupted; head beyond expected floor by >3 mm -> penetration; combined with >=5 mm medial displacement -> central fracture-dislocation (Fig. 7) | [13] |
| Hip articulation | Registered-template defect quantification | Displaced+missing ray fraction >3% -> defect present; graded small (<=7%) / moderate (<=20%) / large (>20%); component structure -> split fracture / detached fragment (Fig. 7, 8) | - |
| Hip articulation | Original-CT support confirmation | Independent CT-density confirmation of the registered-template defect and head position (Fig. 9) | [14] |
| Hip articulation | CT-confirmed defect mapping (registered socket) | Native-CT ray test re-anchored to the registered socket; accepted cluster -> CT-confirmed internal acetabular discontinuity (Fig. 10) | - |
| Hip articulation | Final acetabular zone assembly | Native box union adaptive fragment-recovery mask on the reconstructed side, with mandatory containment QC (Fig. 11) | - |
| Proximal femur | Neck-shaft angle (true 3-D, bilateral) | Compared to CT IQR 117.9-125.6 deg; absolute bilateral difference reported as quantitative flag (Fig. 12) | [17] |
| Proximal femur | Femoral offset asymmetry | Absolute bilateral difference >=5 mm -> FLAGGED (reconstruction-asymmetry comparator) (Fig. 12) | [18] |
| Proximal femur | Limb-length discrepancy | Absolute discrepancy >=10 mm -> FLAGGED (Fig. 12) | [19] |
| Proximal femur | Head-center-to-lesser-trochanter L/D ratio | Outside 0.68-1.00 screening interval (mean 0.84 +/- 0.08) -> FLAGGED (Fig. 12) | [20] |
| Proximal femur | Head asphericity / sphere-fit residual | Normalized RMS >10% or P95 residual >5 mm -> morphology caution (Fig. 4) | [11] |
| Anterior ring | Pubic symphysis diastasis | Narrowest stable gap beyond ~6.1 mm derived boundary (mean 3.1 +/- 1.5 mm reference); 25-mm context for APC-II-pattern widening (Fig. 13) | [1,2,3] |
| Anterior ring | Symphyseal AP/SI shear | No validated static-CT cutoff; reported descriptively against 5-mm dynamic (flamingo-view) motion (Fig. 13) | [4] |
| Anterior ring | Obturator ring discontinuity | Sustained rim departure beyond a 4-mm envelope -> suspected discontinuity (bridge-chord, arc, angular span reported); ramus identity requires visual confirmation (Fig. 3) | [6,7] |
| Posterior ring | Sacroiliac joint width | Median gap beyond age-adaptive comparator (<40y: 1.5-3.1 mm; >=40y: 1.5-2.3 mm) -> width flag (Fig. 14) | [8] |
| Posterior ring | SIJ axial/coronal asymmetry | Reported against open-reduction context (typical 3.39/3.71 mm; absolute 6.52/7.26 mm) (Fig. 14) | [9] |
| Posterior ring | Vertical shear / translation | Vertical alignment offset screen per side; folded into the SIJ-integrity station (Fig. 14) | - |
| Coverage | Larson global femoral-head coverage | Undercoverage below ~36% (mean 40 +/- 2%, approx. -2SD) contributes to routing; overcoverage/asymmetry descriptive only (Fig. 15) | [5] |
| Coverage | LCEA (center-edge angle) | <20 deg undercoverage / 20-25 deg borderline / 25-40 deg typical / >40 deg overcoverage (conventional comparator) (Fig. 16) | [21] |
| Coverage | OIP acetabular index | Informational Sharp-like angle; no clinical cutoff applied (Fig. 16) | - |
| Coverage | Femoral-head coverage (surface / axial-topographic) | Axial topographic coverage <~60% -> reduced-coverage flag (controls 70.1 +/- 5.7%, dysplasia 44.7 +/- 10.1%) (Fig. 16) | [22,23] |

# **Illustrative Findings by Region**

Figures 1-3 (femoral-head/socket simulation, adaptive acetabulum, obturator ring disruption) are given above in the Core Concept section. The remaining flagged-method categories are illustrated below, each from a real processed case.

**Figure 4.** Femoral-head morphology and center-fit quality, Check 1 (Case ACETFX_0013). Right: valid head centre, normal-like morphology, high confidence. Left: valid head centre, morphology caution, moderate confidence.

**Figure 5.** Head-corresponding acetabular margin and local congruity, Check 2 (Case ACETFX_0013). Right margin continuous and congruent. Left margin continuous but suspected dislocation, posterior + inferior, CT-confirmed 36.8 mm displacement relative to the registered expected socket.

**Figure 6.** Native-CT ray-discontinuity test, the shared decisive ray test (Case ACETFX_0013). Flagged: left side, 4 accepted acetabular discontinuity clusters, maximum 3-D span 23.9 mm.

**Figure 7.** Registered-template defect quantification and central/medial-wall subcheck (Case ACETFX_0013). Large posterior-medial acetabular defect, 52.3% defected support against the registered template; no central penetration.

**Figure 8.** Registered-template defect quantification, a more severe example (Case ACETFX_0004). Large superior-posterior split fracture with medial-wall defect; 92.0% defected support against the registered template — the dense orange ray fan marks every ray cast from the registered socket surface toward native bone, converging on the displaced/missing region highlighted in red.

**Figure 9.** Original-CT support confirmation (Case ACETFX_0013). Status confirmed: acetabular defect confirmed, head position confirmed, 18.8% CT-defected support against the registered template.

**Figure 10.** CT-confirmed defect mapping against the registered expected socket (Case ACETFX_0013). Flagged: left side, 1 accepted acetabular discontinuity cluster confirmed directly from original CT density, maximum 3-D span 7.2 mm.

**Figure 11.** Final acetabular zone assembly (Case ACETFX_0013). Final acetabular socket reconstruction completed on the left side; original-CT acetabular defect confirmed; no central penetration.

**Figure 12.** Proximal femoral abnormality screening (Case ACETFX_0013). Left 3-D neck-shaft angle 127.2 deg, above the Hartel CT IQR (117.9-125.6 deg); bilateral femoral-offset asymmetry >=5 mm; limb-length discrepancy 27.2 mm (left shorter); left head-center-to-lesser-trochanter L/D ratio above the published interval.

**Figure 13.** Pubic symphysis diastasis screening (Case ACETFX_0004). Suspected traumatic symphyseal diastasis: gap 21.3 mm, AP/SI shear 0.1 mm / 0.7 mm.

**Figure 14.** Sacroiliac joint disruption and asymmetry screening (Case PRFX_0010). Suspected SIJ diastasis, absolute and asymmetric: right median cortical gap 0.2 mm (narrower than age reference), left 4.0 mm (wider than age reference); P95 side difference axial 6.7 mm / coronal 5.9 mm.

**Figure 15.** Global femoral-head coverage, Larson-adapted method (Case ACETFX_0013). Right 45.2%, left 40.4%, against the 40 +/- 2% asymptomatic comparator.

# **References**

**1.** Hwang K, Wu X, Park CY. Width of pubic symphysis relating to age and sex in Koreans. J Orthop Surg Res. 2021;16(1):430. doi:10.1186/s13018-021-02561-9. PMID:34217332.

**2.** Doro CJ, Forward DP, Kim H, Nascone JW, Sciadini MF, Hsieh AH, et al. Does 2.5 cm of symphyseal widening differentiate anteroposterior compression I from anteroposterior compression II pelvic ring injuries? J Orthop Trauma. 2010;24(10):610-615. doi:10.1097/BOT.0b013e3181cff42c. PMID:20871248.

**3.** Gansslen A, Lindahl J, Krappinger D, Lindtner RA, Staresinic M. The myth of 2.5 cm symphyseal diastasis. Arch Orthop Trauma Surg. 2025;145(1):306. doi:10.1007/s00402-025-05904-x. PMID:40397156.

**4.** Garras DN, Carothers JT, Olson SA. Single-leg-stance (flamingo) radiographs to assess pelvic instability: how much motion is normal? J Bone Joint Surg Am. 2008;90(10):2114-2118. doi:10.2106/JBJS.G.00277. PMID:18829908.

**5.** Larson CM, Moreau-Gaudry A, Kelly BT, Byrd JWT, Tonetti J, Lavallee S, et al. Are normal hips being labeled as pathologic? A CT-based method for defining normal acetabular coverage. Clin Orthop Relat Res. 2015;473(4):1247-1254. doi:10.1007/s11999-014-4055-2. PMID:25407391.

**6.** Zhang F, Zhang D, Huang Z, Wang Z, Cai X. Morphological asymmetry of pelvic rings: a study based on three-dimensional deviation analysis. Orthop Surg. 2022;14(5):967-976. doi:10.1111/os.13246. PMID:35377554.

**7.** Duygu OB, Govsa F. Anatomical structures of the obturator foramen and its importance for pelvic interventions. Surg Radiol Anat. 2025;47(1):163. doi:10.1007/s00276-025-03661-5. PMID:40544414.

**8.** Faflia CP, Prassopoulos PK, Daskalogiannaki ME, Gourtsoyiannis NC. Variation in the appearance of the normal sacroiliac joint on pelvic CT. Clin Radiol. 1998;53(10):742-746. doi:10.1016/S0009-9260(98)80316-4. PMID:9817091.

**9.** Chien RS, Chen IJ, Lai CY, Chen JP, Yu YH. Critical distance of the sacroiliac joint for open reduction using screw fixation for traumatic sacroiliac joint diastasis: a retrospective study. J Orthop Surg Res. 2024;19(1):268. doi:10.1186/s13018-024-04759-z. PMID:38678298.

**10.** Jamali AA, Mak W, Wang P, Tai L, Meehan JP, Lamba R. What is normal femoral head/neck anatomy? An analysis of radial CT reconstructions in adolescents. Clin Orthop Relat Res. 2013;471(11):3581-3587. doi:10.1007/s11999-013-3166-5. PMID:23922189.

**11.** Hassan MM, Feroe AG, Douglass BW, Jimenez AE, Kuhns B, Mitchell CF, et al. Three-dimensional analysis of age and sex differences in femoral head asphericity in asymptomatic hips in the United States. World J Orthop. 2024;15(8):754-763. doi:10.5312/wjo.v15.i8.754. PMID:39165879.

**12.** Rivas DJL, Gassmann JM, Goetz JE, Aitken HD, Davison JC, Miller A, et al. Weight-bearing CT as an approach to assess femoral-acetabular displacement during external rotation stress in the hip. J Hip Preserv Surg. 2025;12(2):93-104. doi:10.1093/jhps/hnaf001. PMID:40761572.

**13.** Judet R, Judet J, Letournel E. Fractures of the acetabulum: classification and surgical approaches for open reduction. Preliminary report. J Bone Joint Surg Am. 1964;46:1615-1646. doi:10.2106/00004623-196446080-00001. PMID:14239854.

**14.** Imani M, Buratto J, Dao T, Meijering E, Vogrin S, Kwok TCY, et al. Deep learning technique for automatic segmentation of proximal hip musculoskeletal tissues from CT scan images: a MrOS study. J Cachexia Sarcopenia Muscle. 2025;16(2):e13728. doi:10.1002/jcsm.13728. PMID:40022453.

**15.** Ead MS, Duke KK, Jaremko JL, Westover L. Investigation of pelvic symmetry using CAD software. Med Biol Eng Comput. 2020;58(1):75-82. doi:10.1007/s11517-019-02068-w. PMID:31745838.

**16.** Rana SH, Farjoodi P, Haloman S, Dutton P, Hariri A, Ward SR, et al. Anatomic evaluation of the sacroiliac joint: a radiographic study with implications for procedures. Pain Physician. 2015;18(6):583-592. PMID:26606010.

**17.** Hartel MJ, Petersik A, Schmidt A, Kendoff D, Nuchtern J, Rueger JM, et al. Determination of femoral neck angle and torsion angle utilizing a novel three-dimensional modeling and analytical technology based on CT datasets. PLoS One. 2016;11(3):e0149480. doi:10.1371/journal.pone.0149480.

**18.** Mahmood SS, Mukka SS, Crnalic S, Wretenberg P, Sayed-Noor AS. Association between changes in global femoral offset after total hip arthroplasty and function, quality of life, and abductor muscle strength. Acta Orthop. 2016;87(1):36-41. doi:10.3109/17453674.2015.1091955.

**19.** O'Brien S, Kernohan G. Perception of imposed leg length inequality in normal subjects. Hip Int. 2010;20(4):505-511. doi:10.1177/112070001002000414.

**20.** Wang G, Guo A, Zhang Y, Qiang H, Yu H, Diao N, Ma L, Zhang Y, Zhao E. Measurement of the relative position of the femoral head center, greater trochanter, and lesser trochanter in the proximal femur. Ann Palliat Med. 2021;10(12):12889-12898. doi:10.21037/apm-21-3137.

**21.** Wiberg G. Studies on dysplastic acetabula and congenital subluxation of the hip joint: with special reference to the complication of osteoarthritis. Acta Chir Scand. 1939;83(Suppl 58):1-135.

**22.** DeFroda SF, Alter TD, Lambers F, Malloy P, Clapp IM, Chahla J, Nho SJ. Quantification of acetabular coverage on 3-dimensional reconstructed computed tomography scan bone models in patients with femoroacetabular impingement syndrome: a descriptive study. Orthop J Sports Med. 2021;9(11):23259671211049457. doi:10.1177/23259671211049457.

**23.** Cheng H, Liu L, Yu W, Zhang H, Luo D, Zheng G. Comparison of 2.5D and 3D quantification of femoral head coverage in normal control subjects and patients with hip dysplasia. PLOS ONE. 2015;10(11):e0143498. doi:10.1371/journal.pone.0143498. PMID:26599869.
